# Expanding the biotechnology potential of lactobacilli through comparative genomics of 213 strains and associated genera

**DOI:** 10.1038/ncomms9322

**Published:** 2015-09-29

**Authors:** Zhihong Sun, Hugh M. B. Harris, Angela McCann, Chenyi Guo, Silvia Argimón, Wenyi Zhang, Xianwei Yang, Ian B Jeffery, Jakki C. Cooney, Todd F. Kagawa, Wenjun Liu, Yuqin Song, Elisa Salvetti, Agnieszka Wrobel, Pia Rasinkangas, Julian Parkhill, Mary C. Rea, Orla O'Sullivan, Jarmo Ritari, François P. Douillard, R. Paul Ross, Ruifu Yang, Alexandra E. Briner, Giovanna E. Felis, Willem M. de Vos, Rodolphe Barrangou, Todd R. Klaenhammer, Page W. Caufield, Yujun Cui, Heping Zhang, Paul W. O'Toole

**Affiliations:** 1 Key Laboratory of Dairy Biotechnology and Engineering, Education Ministry of China, Inner Mongolia Agricultural University, Hohhot, Inner Mongolia 010018, China; 2 School of Microbiology, Alimentary Pharmabiotic Centre, University College Cork, Cork T12 Y337, Ireland; 3 State Key Laboratory of Pathogen and Biosecurity, Beijing Institute of Microbiology and Epidemiology, Beijing 100071, China; 4 College of Dentistry, New York University, New York City, New York 10010, USA; 5 Department Life Sciences & MSSI, University of Limerick, V94 T9PX Limerick, Ireland; 6 Department of Biotechnology, University of Verona, Verona 37134, Italy; 7 Department of Veterinary Biosciences, University of Helsinki, Helsinki 00014, Finland; 8 Wellcome Trust Sanger Centre, Hinxton, CB10 1SA, UK; 9 Department of Biotechnology, Teagasc, Moorepark, Fermoy Co. Cork P61 C996, Ireland; 10 Department of Food, Bioprocessing and Nutrition Sciences, North Carolina State University, Raleigh, North Carolina 27695, USA; 11 Laboratory of Microbiology, Wageningen University, Wageningen, 6703HB, The Netherlands

## Abstract

Lactobacilli are a diverse group of species that occupy diverse nutrient-rich niches associated with humans, animals, plants and food. They are used widely in biotechnology and food preservation, and are being explored as therapeutics. Exploiting lactobacilli has been complicated by metabolic diversity, unclear species identity and uncertain relationships between them and other commercially important lactic acid bacteria. The capacity for biotransformations catalysed by lactobacilli is an untapped biotechnology resource. Here we report the genome sequences of 213 *Lactobacillus* strains and associated genera, and their encoded genetic catalogue for modifying carbohydrates and proteins. In addition, we describe broad and diverse presence of novel CRISPR-Cas immune systems in lactobacilli that may be exploited for genome editing. We rationalize the phylogenomic distribution of host interaction factors and bacteriocins that affect their natural and industrial environments, and mechanisms to withstand stress during technological processes. We present a robust phylogenomic framework of existing species and for classifying new species.

## Abstract (web summary)

*Lactobacillus* is a lactic acid bacteria and has a wide range of application from use in probiotic food production to biotherapeutics. Here, the authors sequence and compare the genomes of 213 different *Lactobacillus* strains and related genera, and provide new insight into phylogenomic organization and adaptive immunity elements in this bacteria family.

## Graphical abstract

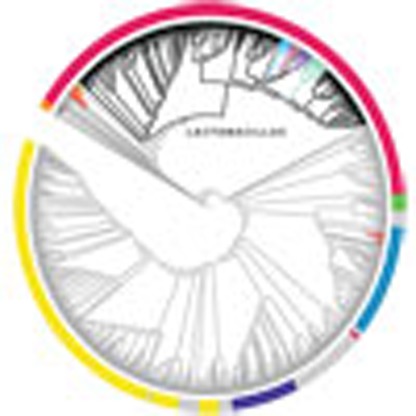

The genus *Lactobacillus* comprises over 200 formally recognized species and subspecies that have been isolated from a wide range of sources^1^. Their ability to ferment raw materials including milk, meat and plants has resulted in their industrial and artisanal use. Hence, many *Lactobacillus* species have a long history of human usage^2^, including recognition as Generally Recognized as Safe or a Qualified Presumption of Safety by the Food and Drug Administration and European Food Safety Authority, respectively^3^. Some strains are marketed as probiotics, meaning they may be beneficial to the consumer beyond basic nutritional value^4^,^5^. Products containing lactobacilli dominate the global probiotics market^6^. In addition to fermentative and preservative properties, some lactobacilli produce exopolysaccharides that contribute to the texture of foods^7^, and to intestinal survival of probiotic species^8^. Furthermore, lactobacilli are under development as delivery systems for vaccines^9^ and therapeutics^10^. In recent years, the relevance of lactobacilli to the chemical industry has considerably increased because of their capacity to produce enantiomers of lactic acid used for bioplastics as well as 1,3-propanediol (a starting ingredient used for biomedicines, cosmetics, adhesives, plastics and textiles)^11^. Thus, lactobacilli are among the microbes most commonly used for producing lactate from raw carbohydrates and synthetic media^12^.

The lactobacilli were originally grouped taxonomically according to their major carbohydrate metabolism, as homofermentative (metabolic group A), facultatively heterofermentative (group B) or obligately heterofermentative lactobacilli (group C)^13^. The accumulation of 16S rRNA gene sequences^14^ and a handful of genome sequences led to the realization that taxonomic and phylogenetic groupings of the lactobacilli were not concordant^15^,^16^,^17^,^18^, that the genus is unusually diverse (as recently reviewed ref. 1) and that a revised genome-based re-classification of the genus was warranted^19^.

To provide an extensive resource for comparing, grouping and functionally exploiting the lactobacilli, we describe here the sequences of 175 *Lactobacillus* genomes and 26 genomes from 8 other genera historically associated with or grouped within the lactobacilli. We complement this analysis by the inclusion of 12 genome sequences from two genera that were already publically available. In all but one case, we sequenced genomes of Type Strains sourced from international culture collections (Supplementary Table 1), to provide taxonomic rigour and to avoid the problems associated with the genome sequence of a non-type strain unintentionally becoming the *de facto* genetic reference for that species, even when it contravened the published type-strain phenotype for that species^20^. This phenomenon has added to confusion on strain identification. Three non-type strain *Leuconostoc* genomes were downloaded from National Center for Biotechnology Information (NCBI; JB16, KM20 and 4,882) and one *Pediococcus* non-type strain was sequenced (AS1.2696). The data underline the extraordinary level of genomic difference across species currently assigned to a single genus, and they provide the definitive resource for mining *Lactobacillus* genes involved in modifying carbohydrates, proteins and other macromolecules, as well as novel Clustered Regularly Interspaced Short Palindromic Repeats (CRISPR)-CRISPR-associated proteins (Cas) systems.

## Results

### A genus more diverse than a family

The genomes of the lactobacilli range in size from 1.23 Mb (*Lactobacillus sanfranciscensis*) to four times larger (4.91 Mb; *L. parakefiri*) as shown in Supplementary Table 1 and Supplementary Fig. 1. The GC content also varies considerably, from 31.93 to 57.02% (Supplementary Fig. 1). The core genome of the 213 strains comprises only 73 genes, the majority of which encode essential proteins for cell growth and replication (Supplementary Table 2 and Supplementary Data, data set 1). Owing to the draft nature of the genomes, this core gene number would increase if the genomes were closed. The genus *Lactobacillus* and associated lactic acid bacteria (LAB) genera have a large open pan-genome whose size increases continuously with the number of added genomes, and contains 44,668 gene families (Supplementary Fig. 2). Exclusion of draft genome assemblies at different fragmentation levels, namely greater than 20, 50, 100, 200, 300, 400 and 500 contigs, does not lead to largely altered predictions for the pan-genome curves. Core genome curves were also generated using the same fragmentation levels and these curves are similar, especially for higher fragmentation levels. The core gene curves do show, however, that contig numbers have an effect on the core genome size (Supplementary Fig. 2). Although niche associations and described sources for *Lactobacillus* strains and species are not all equally robust, there was a clear trend for the genomes of species isolated from animals to be smaller, consistent with genome decay in a nutrient-rich environment^18^ (Supplementary Fig. 3).

ANI (average nucleotide identity) is the average identity value calculated from a pair-wise comparison of homologous sequences between two genomes and is frequently used in the definition of species^21^,^22^. The frequency distribution of pair-wise ANI values for *Lactobacillus* species differs substantially from the distribution of values for genus and family, overlapping with values for order and class (Supplementary Fig. 4). TNI (total nucleotide identity) is an improved method that determines the proportion of matched nucleotide sequences between pairs of genomes, providing a higher discriminatory power for the high-level taxonomy units in this data set^23^. The TNI calculations indicate that the genomic diversity of the genus *Lactobacillus* is intermediate between that of the majority of the currently approved taxonomic units for orders and families (http://www.bacterio.net/), and the mean value of TNI between all species in this genus is 13.97% (Supplementary Fig. 4). Thus, although *Lactobacillus* has traditionally been defined as a genus, its genetic diversity is larger than that of a typical family.

### A paraphyletic genus intermixed with five other genera

**Figure 1:**
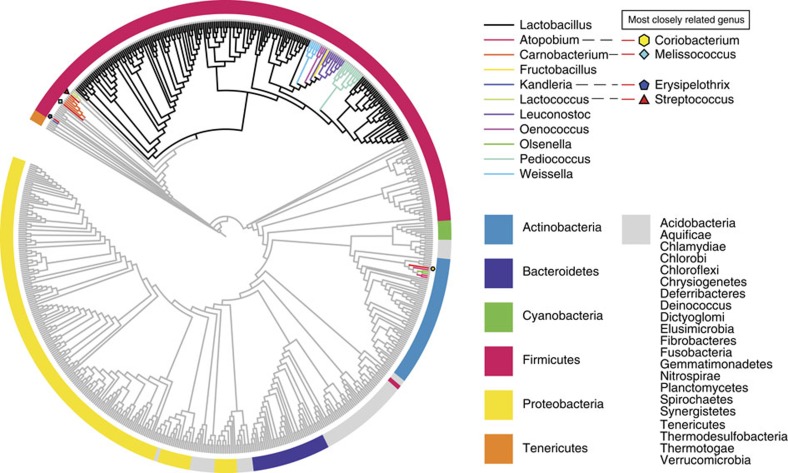
Cladogram of 452 genera. Cladogram of 452 genera from 26 phyla with the 213 genomes analysed in this study, based on the amino-acid sequences of 16 marker genes. The tree was built using the maximum likelihood method but visualized by removing the branch length information. The coloured branches indicate different genera sequenced in this research; grey branches indicate members of genera whose genomes were previously sequenced. The colours in the outer circle represent the phyla that are indicated in the legend, and the different shapes near branch-tips indicate the position of genera that are most closely related to *Atopobium*, *Carnobacterium*, *Kandleria* and *Lactococcus*, separately.

**Figure 2:**
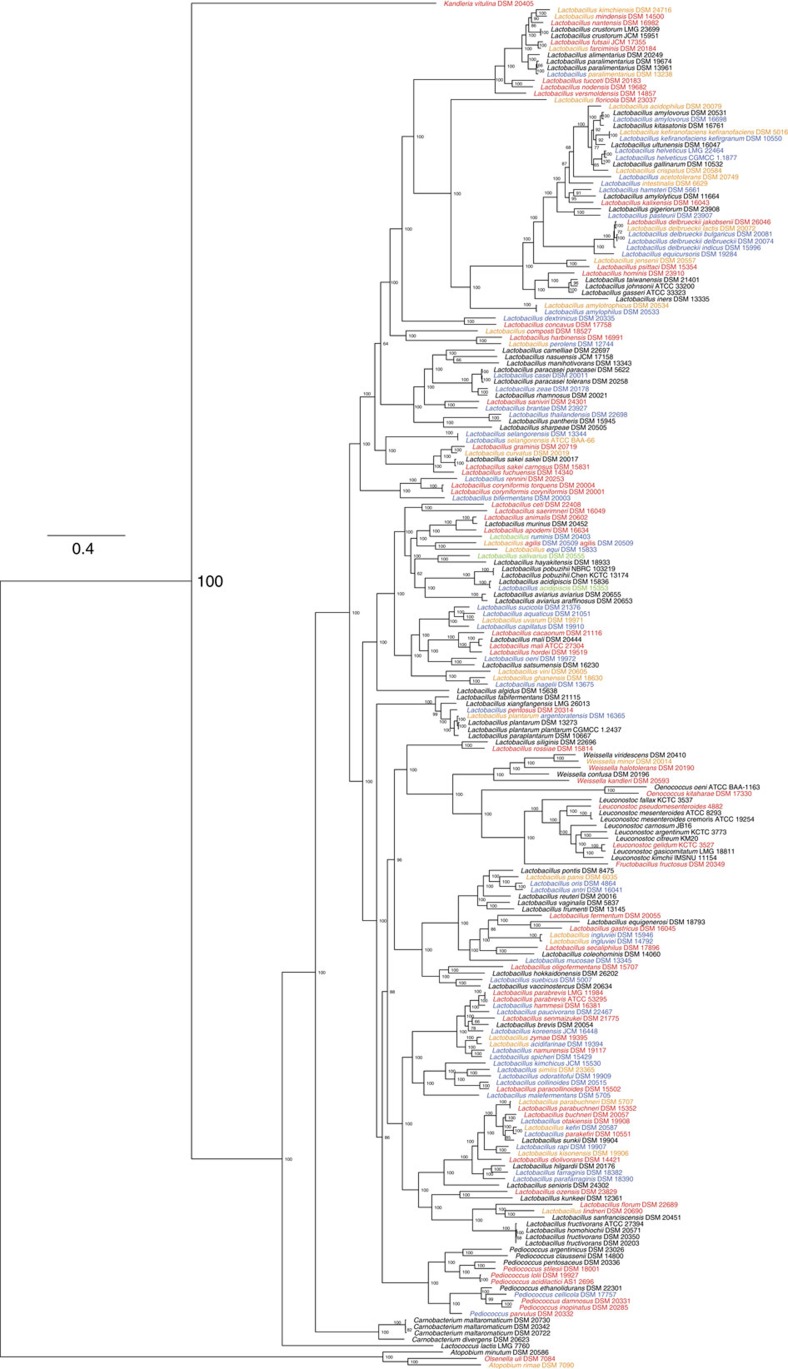
Maximum likelihood phylogeny derived from 73 core genes across 213 strains. The phylogeny was estimated using the PROTCATWAG model in RAxML and rooted using the branch leading to *Atopobium minutum* DSM 20586, *Olsenella uli* DSM 7084 and *Atopobium rimae* DSM 7090 as the outgroup. Bootstrapping was carried out using 100 replicates and values are indicated on the nodes. Colours on taxon labels indicated the presence of CRISPR-Cas systems using blue, red and green for Type I, II and III systems, respectively. Undefined systems are represented in orange. Colour combinations were used when multiple systems from different families were concurrently detected in bacterial genomes.

In light of the extraordinary genomic diversity of the genus *Lactobacillus* and its polyphyletic nature, we set out to provide the most comprehensive phylogenetic study of the genus to date, thereby removing ambiguities in uncertain classifications and further validating existing taxonomic relationships. We constructed a phylogenetic tree with the lactobacilli and representative genomes of 452 selected genera from 26 phyla (Supplementary Table 3) using 16 proteins common to all taxa (see Methods section for details and selection criteria; see Supplementary Table 4 for the protein list and Supplementary Data, data set 2 for sequences). The phylogeny revealed that *Lactobacillus* is paraphyletic and that all species of *Lactobacillus* descend from a common ancestor (Fig. 1; this tree with taxon names and branch lengths is presented in Supplementary Fig. 5). However, five other genera, *Pediococcus*, *Weissella*, *Leuconostoc*, *Oenococcus* and *Fructobacillus*, are grouped within the lactobacilli as sub-clades. This phylogenomic arrangement was confirmed by a maximum likelihood tree constructed from the 73 core proteins shared by the 213 genomes of the lactobacilli and 10 associated genera (Fig. 2). This tree is supported by high bootstrap values, which supports the 73 core proteins as being reflective of the evolutionary history of the lactobacilli and associated genera. The genera *Pediococcus*, *Leuconostoc* and *Oenococcus* have long been recognized as phylogroups within the genus *Lactobacillus* based on both 16S rRNA gene sequence typing and extensive phylogenomic analysis^1^,^18^. Our results provide unequivocal evidence that the genera *Fructobacillus* and *Weissella* are members of the *Lactobacillus* clade, with *Fructobacillus* located between *Leuconostoc* and *Oenococcus* and the genus *Weissella* located as a sister branch (Fig. 2). As the *Lactobacillus* clade includes species from six different genera (*Lactobacillus*, *Pediococcus*, *Weissella*, *Leuconostoc*, *Oenococcus* and *Fructobacillus*), we propose to name these six genera as constituting the *Lactobacillus* Genus Complex. Interestingly, the *Carnobacteria* are external to the *Streptococcus*/*Lactococcus* branch in the 16-core phylogeny of 26 phyla (Fig. 1), but they are internal to this branch in the 73-core tree of the *Lactobacillus* Genus Complex and associated genera (Fig. 2). The lower bootstrap value of 51% (*L. lactis*) for the 16-core tree, which was built from an alignment of 3,863 bp, suggests that there was not enough phylogenetic signal to resolve this branch to a high degree of confidence. In contrast, the 73-core tree, which was built from an alignment of 30,780 bp, has a bootstrap value of 100% for this branch. This places greater confidence in the latter tree topology and hence it was used in all downstream analyses.

As a complement to the maximum likelihood tree of the *Lactobacillus* Genus Complex and associated genera based on 73 core proteins (Fig. 2), we built another tree (Supplementary Fig. 6) omitting *Atopobium*, *Olsenella*, *Kandleria* and *Carnobacterium* genomes and retaining the position of the most recent common ancestor (MRCA) according to the tree of bacteria (Fig. 1). In agreement with previous observations based on 28 LAB genomes^17^, this tree shows that the *Lactobacillus* Genus Complex splits into two main branches after diverging from the MRCA. Branch 1 contains the type species of the genus *Lactobacillus*, *L. delbrueckii*, and a large number of type strains that were isolated from dairy products. Branch 2 contains more species (*n*=127) than branch 1 (*n*=77), and all five of the other genera in the *Lactobacillus* Genus Complex.

### A broad repertoire of carbohydrate-active enzymes (CAZymes)

With interest in their applications in fermentations, some of the earliest classifications of lactobacilli were based on their carbohydrate utilization patterns^13^. Glycolysis occurs in obligately homofermentative (group A) and facultatively heterofermentative (group B) lactobacilli, and has been traditionally linked to the presence of 1,6-biphosphate aldolase^24^. A full set of glycolysis genes were predicted in 49% of the species analysed (Supplementary Fig. 7) and gene duplication is common, although not particularly associated with a group or niche. All *Lactobacillus*, *Leuconostoc*, *Weissella*, *Fructobacillus* and *Oenococcus* species lacking phosphofructokinase (Pfk) formed a distinct monophyletic group. This group included the historically defined *L. reuteri*, *L. brevis*, *L. buchneri*, *L. collinoides*, *L. vaccinostercus* and *L. fructivorans* groups. Most species (75%) within this Pfk-negative clade also lacked 1,6-biphosphate aldolase, although this gene was consistently present in the *Weissella* clade as well as in some leuconostocs and species from the *L. reuteri* and *L. fructivorans* groups. Importantly, most species (87%) within the Pfk-lacking group were classified as obligatively heterofermentative^1^, with the rest being facultatively heterofermentative. The reason for the link between *pfk* gene loss and heterofermentative metabolism needs functional genomic investigation. The average phylogenetic distance (number of nodes to root) of facultatively heterofermentative lactobacilli (as defined in Supplementary Fig. 7) to the MRCA (Supplementary Fig. 6) is considerably lower than that of obligately heterofermentative or obligately homofermentative species (Supplementary Fig. 8), suggesting that the *Lactobacillus* MRCA was facultatively heterofermentative. The obligatively heterofermentative species also form a distinct cluster that may be explained by several evolutionary scenarios that require further investigation.

**Figure 3:**
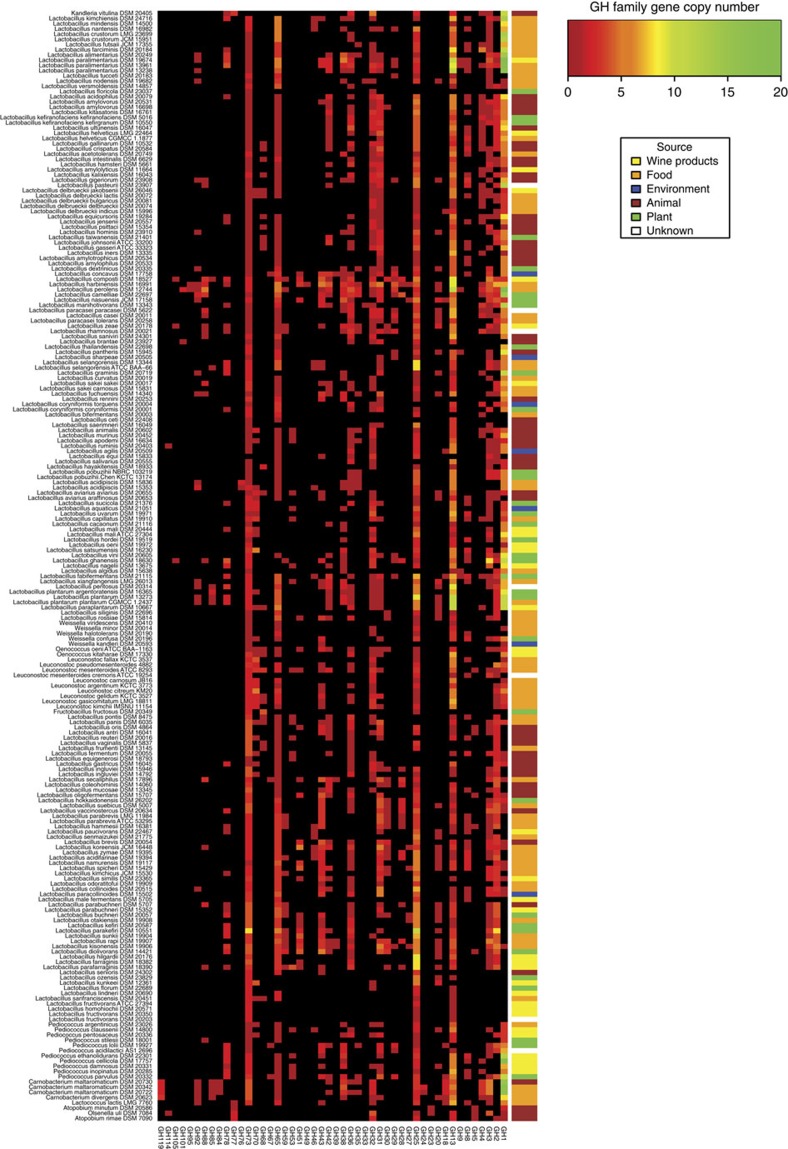
Heat map illustrating the distribution and abundance of glycoside hydrolase (GH) family genes across the *Lactobacillus* Genus Complex and associated genera. Gene copy number of each of the 48 represented GH families is indicated by the colour key ranging from black (absent) to green. Strains are graphed in the same order left to right as they appear top to bottom in the phylogeny (Fig. 2) with the isolation source of each strain indicated by the colour bar at the top of the heat map.

Biotransformation of carbohydrates by bacteria can be exploited for transforming raw materials, for optimizing growth and for producing valuable metabolites. The 213 genomes collectively encode 48 of the 133 families of glycoside hydrolases (GHs) in the CAZy database (http://www.cazy.org), many of which represent unrecognized and unexploited enzymes for biotechnology (Fig. 3). Chitin is the second most abundant natural polysaccharide after cellulose. Among 115 LAB species previously tested, only *Carnobacterium* spp. were able to hydrolyse α chitin^25^. In this study, three new *Carnobacterium* genomes, along with strains of *L. delbrueckii*, *L. nasuensis*, *L. agilis*, *L. fabifermentans* and *Pediococus*, provide the genetic information to exploit that activity. The GH39 genes are β-xylosidases that are present in the *L. rapi*/*L. kisonensis* branch as well as two singleton species, *L. concavus* and *L. secaliphilus*. GH49 (dextranase) and GH95 (α-fucosidase) are harboured only in the *L. harbinensis*/*L. perolens* branch with GH49 being absent from the latter species. Dextranases are considered to be the most efficient means for hydrolysing undesirable dextrans at sugar mills^26^. Microbial mannanases hydrolyse complex plant polysaccharides and they have applications in the paper and pulp industry, for food and feed technology, coffee extraction, oil drilling and detergent production; the corresponding GH76 is found only in the two *L. acidipiscis* strains. GH101 is found only in *L. brantae*, isolated from goose faeces, and *L. perolens*, which is from a beverage production environment. This GH is an endo-α-N-acetylgalactosaminidase, which is thought to play a role in the degradation and utilization of mucins by probiotic bifidobacteria^27^. Although this explains its presence in the goose intestine, its association with beverage production may be due to limited hygiene.

We identified two GH families not previously associated with the *Lactobacillus* genus complex. GH67 displays α-glucuronidase activity^28^ and is involved in the breakdown of xylan; such enzymes have an application in the pulp industry for bio-bleaching, in the paper industry, as food additives in poultry and in wheat flour for improving dough handling^29^. GH95 fucosidases can cleave and remove specific fucosyl residues^30^. Fucose residues are present in oligosaccharides in milk and on erythrocyte surface antigens. Some GH types appeared to be common across the genome data set, if not universal, and these are described in Supplementary Information.

Analysis of the 213 genomes reveals they encode representatives of 22 of the 95 families of glycosyltransferases (GT) in the CAZy database with a high level of GT-encoding diversity and a number of surprising findings (Supplementary Fig. 9). Glycogen is one of five main carbohydrate storage forms used by bacteria, and a previous analysis of 1,202 diverse bacteria concluded that bacteria that can synthesize glycogen occupy more diverse niches^31^. GT5 and GT35 are glycogen synthase and glycogen phosphorlyase, respectively. These GTs are encoded by the *L. casei* clade, which includes two species that are currently exploited heavily as probiotics, *L. casei* and *L. rhamnosus*, as well as the *L. plantarum* group, some members of the *L. salivarius* group (such as *L. salivarius* itself) and a number of singletons. It is not clear if the ability to synthesize glycogen contributes to the biological fitness of these species. Strikingly, among the sequenced genomes only *L. gasseri* encodes GT11 (galactoside α-1,2-L-fucosyltransferase), whereas only *L. delbrueckii* DSM15996 encodes GT92 (N-glycan core α-1,6-fucoside β-1,4-galactosyltransferase). Surface fucose is common in pathogens, including *Helicobacter pylori*, where it is linked to antigenic mimicry (with Lewis blood group antigens), immune avoidance and adhesion^32^. According to the CAZy database, the GT11 fucosyltransferase is uncommon in LAB; it is present in *Akkermansia muciniphila*, in a minority of commensal *Bacteroides*, in three *Roseburia* species and in several Proteobacteria. Interestingly, GT92 is not described in any prokaryotic organisms in CAZy, but the current study identified the characteristic GT92 domain in *L. delbreuckii*. The production of surface fucose-containing moieties by certain *L. gasseri* and *L. delbreuckii* strains merits biological evaluation.

### Interaction factors on the *Lactobacillus* cell surface

**Figure 4:**
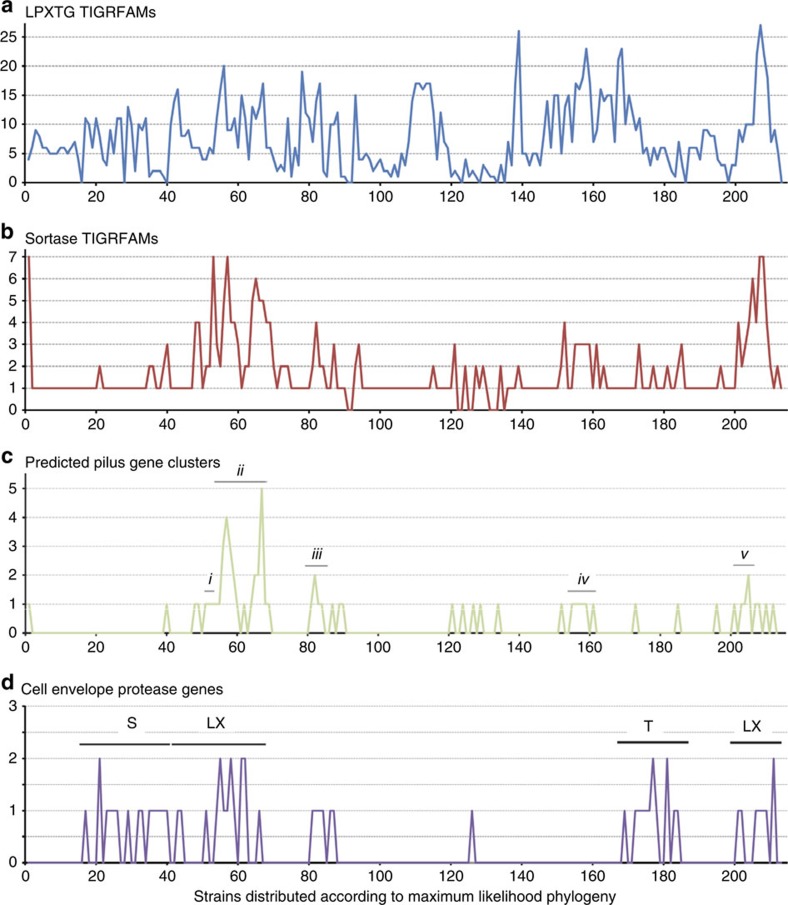
Differential abundance of genes encoding LPXTG proteins, sortases, pili and cell envelope proteases (a–d, respectively). The *y* axis indicates the number of genes/clusters detected. Strains are graphed in the same order left to right as they appear top to bottom in the phylogeny (Fig. 2). In **c**, each black bar indicates strains belonging to the same lineages. (**c**) Labels: *i*, the *L. composti* clade; *ii*, the *L. casei/rhamnosus* clade; *iii*, the *L. ruminis* clade; *iv*, the *L. brevis/parabrevis* clade; *v*, the *Pediococcus ethanolidurans* clade. (**d**) Labels: LX, LPXTG-sortase-dependent anchor (including derivatives); S, S-layer type anchor; T, truncated protein.

Surface proteins of lactobacilli include key interaction receptors for probiotics and enzymes for growth in milk. A major class of surface proteins in Gram-positive bacteria are those anchored by sortase enzymes that recognize a highly conserved LPXTG sequence motif^33^. We identified 1,628 predicted LPXTG-containing proteins and 357 sortase enzymes in the 213 genomes (Supplementary Table 5). The number of sortases and LPXTG proteins greatly varies between species (Fig. 4), with 0 to 27 LPXTG proteins found. The highest number of LPXTG proteins (27) occurred in the milk isolate *Carnobacterium maltaromaticum* DSM 20342. Other species of the genus *Carnobacterium* also showed a large LPXTG protein repertoire, suggesting extensive interactions within their respective habitats and associated microbial communities. Among the variety of LPXTG proteins, we particularly focused on sortase-dependent pilus gene clusters (PGCs). Common in Gram-positive pathogens, these proteinaceous fibres are also produced by commensal bacterial species such as *L. rhamnosus*^34^ and the SpaCBA pili have been shown to contribute to probiotic properties by mucin binding^35^ and cellular signalling^36^. A total of 67 PGCs were predicted in 51 bacterial strains (Fig. 4), most strains harbouring a single PGC (Supplementary Fig. 10). Only about one-third of the piliated strains possessed PGCs similar to *L. rhamnosus* strain GG pilus clusters in terms of gene order, that is, a cluster of three pilin genes and one pilin-specific sortase gene. The remaining pilus clusters showed the presence of two other major types and numerous other types that are different in organization and sequence from that of *L. rhamnosus* GG (Fig. 4). Five particular clades were associated with the presence of PGCs. The ecologically diverse *L. casei*/*L. rhamnosus* clade (Fig. 4c, Clade ii) harboured the greatest number of piliated species. Some strains, for example, *L. equicursoris*, *W. confusa* and *L. parabuchneri* (DSM 15352) are distinguished by being the only piliated species within their respective clades (Fig. 4c), which we cannot currently explain. The availability from this study of over 50 new PGCs is expected to provide new avenues for addressing their role in probiotic and other functions.

### Differential evolution of cell envelope protease (CEP) genes

CEPs) are multi-subunit, cell-wall-anchored, subtilase-type proteinases produced by many LAB. They are primarily associated with cleaving casein as the first stage in releasing peptides and amino acids during growth in milk, and variations in their sequence and domain structure contribute to determining the flavour of cheese^37^. In particular, the protease-associated (PA) domain and the A domain have been shown to impact on the specificity of the enzyme. The A domain has been subdivided into three fibronectin domains (Fn1, Fn2 and Fn3) and these are implicated in substrate binding^38^. Furthermore, some CEPs of commensal lactobacilli may act upon inflammatory mediators to ameliorate inflammatory bowel disease^39^, so mining the novel *Lactobacillus* genomes for these proteases could identify novel therapeutics for chemokine-mediated inflammatory diseases. We identified genes for 60 CEPs in the 213 genomes, ranging from 1,097 to 2,270 amino acids in length (Supplementary Table 6). Forty-four strains had a single CEP, whereas eight strains encoded two distinct CEPs (Fig. 4). Four disrupted CEP genes were detected, two occurring at contig boundaries. Presence of genes for CEPs exhibited clear clade association, notably with the *L. delbrueckii*, *L. casei* and *L. buchneri* clades, part of the *L. salivarius* clade, and the *Carnobacterium* clade.

The CEPs are defined as cell associated, and different anchoring mechanisms have been identified. In all, 17 of the 60 CEPs incorporated a SLAP domain, putatively responsible for non-covalent interactions with the cell wall, 12 had a canonical LPXTG motif for covalent linkage to peptidoglycan, and a further 18 had a derivative of the LPXTG motif (Fig. 4). Interestingly, 13 of the CEPs had neither an S-layer-type anchoring domain nor an LPXTG-type motif. These proteins all terminated precisely before standard anchoring motifs at a sequence conserved across all of the 60 identified CEPs, suggesting that this was non-random. Of these 13 CEPs, 11 are in the *L. buchneri* clade, suggesting positive selection for release of protease activity into the growth medium in this clade. There may be an advantage to the cell by releasing enzymes away from the cell surface and not saturating or competing for cell wall anchoring. Twelve of these thirteen CEPs cluster in a distinct group in a phylogenetic tree and the multiple alignments indicate the sequences differ from other CEPs along the entire length of the protein (data not shown). Putative anchoring by the SLAP domain is notably associated with the *L. delbrueckii* sub-clade, whereas CEPs containing LPXTG motifs occur in the *L. casei*, *L. salivarius*, *Pediococcus* and *Carnobacterium* groups.

The pair-wise amino-acid identity values between the 60 CEPS ranged from 100% down to just 20%, a level of divergence indicating the likelihood that some of these proteases have novel specificity. Of the 60 CEPs identified, 23 had the PA domain, 57 the Fn1 domain (DUF_1034) and 25 the Fn2 domain (CHU_C). Interestingly, there is some association between anchoring mechanism and domain composition. For the SLAP domain-containing CEPs, 12 out of 17 do not contain the Fn2 domain, and for the CEPs devoid of SLAP or LPXTG sequences, 11 out of 13 do not contain a PA domain. The differential domain composition in the CEPs indicates that a diverse range of substrates and products are likely. These properties may be exploitable for improvement of food flavour or for enhanced probiotic capabilities.

### CRISPR-Cas systems and mobile genetic elements

CRISPR in combination with Cas constitute CRISPR-Cas systems, which provide adaptive immunity against invasive elements in bacteria^40^. Sequences derived from exogenic elements are integrated into CRISPR loci, transcribed and processed into mature small interfering RNAs, and the small CRISPR RNAs (crRNAs) specifically guide Cas effector proteins for sequence-dependent targeting and endonucleolytic cleavage of DNA sequences complementary to the spacer sequence^41^. CRISPR-Cas systems have revolutionized genetic engineering and gene therapy by enabling precise targeted manipulations in prokaryotic^42^ and eukaryotic genomes^43^, and recently in lactobacilli^44^.

A total of 137 CRISPR loci were identified in 62.9% of the genomes analysed, representing all the major phylogenetic groups of lactobacilli evaluated (Fig. 2). This indicates that these systems are evolutionarily widespread throughout this genus, and likely functionally important. This is considerably higher than the ∼46% general occurrence rate in bacterial genomes in CRISPRdb^45^. There was overall congruence between the phylogenomic structure of the lactobacilli (Fig. 2) and CRISPR-Cas system phylogeny (Supplementary Fig. 11) reflecting co-evolutionary patterns. For Type allocation, the signature genes *cas3, cas9* and *cas10* for Types I, II and III, respectively, were used, complemented by comparison of CRISPR-repeat sequences and the universal Cas1 protein^45^. Types I, II and III CRISPR-Cas systems were all detected (66, 68, and 3 systems, respectively; Supplementary Table 7). Comparative analyses of defining CRISPR features revealed a diversity of the universal Cas1 protein and corresponding CRISPR-repeat sequences, with consistent clustering in two main families representing Type I and Type II systems (Supplementary Fig. 11). Strikingly, Type II systems were detected in 36% of the *Lactobacillus* Genus Complex and associated genera, although they occur in only 5% of all bacterial genomes analysed to date^46^, suggesting these LAB are a rich resource for Type II CRISPR systems. Beyond the diversity of CRISPR-Cas systems, we further uncovered dramatic variability in locus size and spacer content, ranging from 2 to 135 CRISPR spacers (Supplementary Table 7).

**Figure 5:**
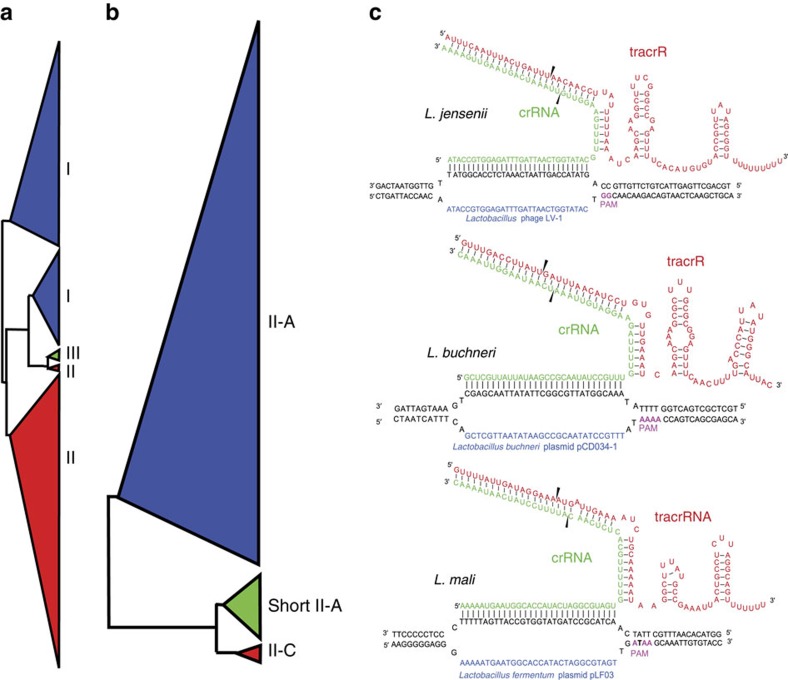
Comparative analysis of CRISPR sequences. The tree in **a** is derived from an alignment of the sequence of the universal Cas protein, Cas1, to create a phylogenetic tree based on the relatedness of all CRISPR-Cas systems in lactobacilli and closely related organisms. Types I, II and III are represented in blue, red and green, respectively. The tree in **b** is derived from an alignment of Cas9, the signature protein for Type II systems, to create a phylogenetic tree showing the relatedness of Cas9 proteins from Type II-A and II-C systems identified in lactobacilli and closely related organisms. A subset of short Type II-A Cas9 proteins is highlighted. In **c**, key guide sequences driving DNA targeting by Cas9 are shown for *L. jensenii*, *L. buchneri* and *L. mali*. Predicted crRNA and tracrRNA sequences are shown at the top (red). Complementarity between CRISPR spacer sequences and target protospacer sequences (blue) in target nucleic acids is shown for phages and plasmids. The predicted protospacer-adjacent motif (PAM) sequences flanking the 3′ end of the protospacer sequence are shown in green.

Type II CRISPR-Cas systems, which comprise the signature Cas9 endonuclease have received tremendous interest given their ability to re-programme Cas9 using customized guide RNAs for sequence-specific genesis of double-stranded breaks and the corresponding ability to edit genomes using DNA repair machinery. Here, we observed a diversity of novel Type II systems with heterogeneous Cas9 sequences (Supplementary Fig. 12A) that expands the Cas9 space considerably, and the corresponding DNA targeting and cleavage features including the proto-spacer adjacent motif and guiding RNAs^47^. Novel Cas9 proteins we discovered include some relatively short Type II-A and Type II-C Cas9 homologues (1,078–1,174 amino acids) that have potential for efficient virus-based packaging and delivery (Fig. 5). Furthermore, we determined corresponding putative *trans*-activating crRNAs (tracrRNAs) for Type II-A systems (Supplementary Fig. 12B), which is instrumental in designing wild-type crRNA:tracrRNA guides and synthetic single-guide RNAs for Cas9 (ref. 47). We further characterized the key elements of Type II systems for *L. jensenii*, *L. buchneri* and *L. mali* (Fig. 5), revealing the sequence diversity and structure conservation for the guide RNAs and their corresponding proto-spacer adjacent motifs.

Phage and plasmid sequences were detected in 92% and 41% of the 213 genomes, respectively (Supplementary Figs 13 and 14). Several synteny-based methods were used for predicting prophages, but the results were inconclusive and subsequent manual analysis did little to improve this. Prediction of phage-specific genes was therefore used as an alternative and synteny-based methods of prophage prediction will be optimized for future studies. There is a trend towards an inverse correlation between abundance of CRISPR sequences and phage sequences that does not reach statistical significance (data now shown). Lactobacilli can have complex genome architecture^48^, and in many genomes multiple plasmids were detected (for example, six plasmids predicted in both *L. parafarraginis* and *P. claussenii*; Supplementary Fig. 14). The phenomenon of very large plasmids exemplified by the sole genome sequence harbouring a megaplasmid in this analysis (the 380 kb megaplasmid of *L. salivarius* DSM20555 (ref. 49)) substantially increases the number of plasmid-borne genes that are assigned to cluster of orthologous groups (COGs) for this genome (Supplementary Fig. 14). However, the influence of the megaplasmid on COG abundance is not evident on a genome-wide scale (Supplementary Fig. 15). These vectors open new avenues for genetic manipulation of model lactobacilli in the laboratory and for food-grade strain development. Furthermore, a diversity of insertion sequence (IS) elements was identified (Supplementary Fig. 16) including widespread IS families (IS3 is nearly universal), as well as sequences that selectively occur in particular niches (for example, IS91 in dairy *L. casei* and *L. paracasei tolerans* and IS481 in brewing *L. paracollinoides*, *L. farraginis* and *P. inopinatus*). Altogether, mobile genetic elements and their occurrence reflect both the open pan-genome of lactobacilli and evolution by gene acquisition, and genome simplification and decay. Functionally, we also show that detected CRISPR spacer sequences can perfectly match target phage and plasmid sequences (Fig. 5), which is consistent with sequence-specific targeting of viruses by CRISPR-Cas adaptive systems. The findings from analysis of these 213 genomes corroborate previous reports implicating CRISPR-Cas systems in adaptive immunity against bacteriophages and plasmids in lactic acid bacteria used as starter cultures in food fermentation.

## Discussion

This *Lactobacillus* genome sequencing initiative provides genomic clarity for a genus bedevilled by phenotypic confusion and inconsistent phylogeny. We generated a resource data set whose analysis explained the phenotypic diversity of lactobacilli and associated genera, and suggested new units for classification. The 200 genomes sequenced were from organisms spanning 9 genera and 174 species; including available *Oenococcus* and *Leuconostoc* genomes brought this to 11 genera and 185 species. We sequenced the genomes of *L. crustorum*, *L. parabrevis*, *L. pobuzihii* and *L. selangorensis* twice, but from different culture collections, and their sequence identity validated the sequencing and analysis pipelines. We elected to produce genomes of high-quality draft standard^50^, which is suitable for mining all relevant phylogenetic and functional information, and allows easy custom finishing as desired for genome regions of interest or whole genomes. Of the 200 type strains sequenced, 179 were previously unavailable on NCBI, which allows an unprecedented degree of integration of *Lactobacillus* genomics into taxonomic discussions and decisions. As we started the sequencing phase, an additional 29 lactobacilli or candidate lactobacilli have been published in the literature; the definition of core genes and robust phylogeny described here will make their addition to the phylogenome easy once their genomes are sequenced.

Uncertainty surrounding species assignment and grouping into larger taxonomical units is undesirable, and it presents a considerable challenge for some bacteria such as those we termed here ‘the *Lactobacillus* Genus Complex'. Formal re-classification is the prerogative of systematic committees, but we examined phylogenomic approaches that might guide such classification. We first examined the most recent phylogeny^1^ containing 16 phylogroups, and determined the frequency distribution of branch distances within phylogroup co-members and non-members (Supplementary Fig. 17A1) based on the core gene tree (Fig. 2). We also calculated the frequency distribution of whole genome-wide genetic distance that is measured by the 1-TNI value (Supplementary Fig. 17B1). The ideal phylogrouping that would yield non-intersecting curves was clearly not achieved through measurement of branch lengths or TNI values. Therefore, we manually edited phylogroup membership primarily to concord with monophyletic clades, as well as to minimize the intersection area between curves (Supplementary Fig. 18). Although the TNI value distribution was still not discriminatory after optimizing the phylogroups (Supplementary Fig. 17B2), we achieved superior separation of branch length distribution (Supplementary Fig. 17A2). However, a stringent cutoff value for judging whether two strains belong to the same phylogroup could not be achieved, which may be due to unequal clock rates or speciation rates throughout the tree (which will be hard to determine based on current strain information). Nevertheless, the revised phylogrouping based on core genome comparison presented here can serve as the basis for discussions of formal re-classification.

Mobile replicons including bacteriophages and plasmids are a prominent feature of this group of bacteria, and have historically attracted attention because of their ability to extend the phenotype of a strain, or in the case of phage, to lyse starter or adjunct cultures. The data in this genome resource extend the knowledge base for exploiting the *Lactobacillus* mobilome. There is also a proportional abundance of systems to modulate the movement of these replicons. Collectively, our data reveal the widespread occurrence of diverse CRISPR-Cas immune systems in the genomes of lactobacilli, including a plethora of novel Type II systems with diverse Cas9 sequences. Of particular interest is the identification of a variety of Cas9 proteins that can be used in combination with novel guide sequences and various associated targeting motifs for flexible DNA targeting and cleavage. We anticipate that these novel systems will open new biotechnological avenues for next-generation Cas9-mediated genome editing in eukaryotes and prokaryotes. The broad occurrence of diverse CRISPR-Cas immune systems in lactobacilli in general also provides enormous potential for strain genotyping and enhancing phage resistance in industrial strains.

The genomic analysis highlights the remarkable diversity of pili in lactic acid bacteria. This also suggests that the pilus biogenesis, assembly and also function may differ quite considerably between strains. To date, there have been only a few reports describing pili in *Lactobacillus* species other than *L. rhamnosus*. The present data offer a useful basis for future functional studies of these potentially piliated species from an environmental and evolutionary perspective.

Our data indicate that the *Lactobacillus* ancestor was facultatively heterofermentative, and that selective gene loss events have fine-tuned glycolysis/hexose/pentose metabolism in clade-specific patterns, against the back-drop of generalized gene loss and genome decay that characterizes the evolution of the Lactobacillales^18^. The selective pressures other than in the dairy environment are not well understood. Further evolutionary analyses are expected to resolve the presence of exceptions we described within major groups (characterized by a different genetic background compared with that of the whole group).

Apart from a pattern driven by genome reduction in animal-associated strains, we did not identify evidence for strong association between the niches of particular species and their genomic content (Supplementary Information), although it must be recognized that the recorded isolation source of any given species may not necessarily be where it evolved. The strongly divergent patterns already illuminated by the current data set for genes involved in carbohydrate management, proteolysis, surface protein production and destruction of foreign DNA provide a rational framework for species selection, trait browsing, replicon design and process optimization in fermentation and bioprocessing applications.

## Methods

### Sequencing and assembly

Whole-genome sequencing was performed using Illumina HiSeq 2000 (Illumina) by generating 100 bp paired-end read libraries following the manufacturer's instructions. An average of 190 Mb of high-quality data were generated for each strain, corresponding to a sequencing depth of 16- to 185-fold (Supplementary Table 1).

The paired-end reads were first *de novo* assembled using SOAPdenovo v1.06, local inner gaps were then filled and single base errors were corrected using the software GapCloser (http://sourceforge.net/projects/soapdenovo2/files/GapCloser/). The individual genome assemblies of 200 strains have been deposited in the NCBI under the project numbers PRJEB3060 and PRJNA222257 with individual accession numbers listed in Supplementary Table 1. Raw reads for 200 strains have been deposited in the sequence read archive under the sample accession IDs listed in Supplementary Table 1.

### Coding sequence (CDS) prediction and annotation

The CDSs of genes were predicted for each sequenced genome by using Glimmer v3.02 (ref. 51). Partial genes were predicted by replacing gaps between contigs by a six-frame start/stop sequence (5′- NNNNNCACACACTTAATTAATTAAGTGTGTGNNNNN -3′). Glimmer3 normally predicts only complete genes, but a partial gene at a contig boundary with the above sequence at one or both ends will be predicted and given artificial end(s) (for example, 5′- NNNNNCACACACTTAA -3′ at the 3′ end). The number of partial genes along with their status (5′ end missing, 3′ end missing, both ends missing) was determined using these artificial ends. To obtain functional annotation, the amino-acid sequences of predicted CDS were blasted (BLASTP) against the nr database with the criterion of e-value<1e-5, identity>40% and length coverage of gene>50%. Additional annotation was obtained from the COG^52^ and Kyoto Encyclopaedia of Genes and Genomes (KEGG)^53^ databases using BLASTP and the same BLAST thresholds.

### Construction of core- and pan-genome families

For identifying the pan-genome, a pair-wise comparison was performed using *L. gasseri* ATCC33323 as the first genome, followed by the random selection of each of the remaining genomes, without replacement, until all 213 genomes were included. Gene families were identified where homologous genes were found with BLASTP above the threshold of 25% identity over 40% of the gene length. Genes that fell below these thresholds formed new families, all of which were summed to give the pan-genome family set. A pan-genome family set was also derived after removing the genomes with greater than 20, 50, 100, 200, 300, 400 and 500 contigs to assess the effect of higher contig number on pan-genome size (Supplementary Fig. 2).

To identify core genes for phylogenetic analysis, gene predictions for the 213 genomes were translated from nucleotide into amino-acid sequences and used as the input for QuartetS^54^. QuartetS first predicted orthologues by reciprocal best BLAST between pairs of genomes using cutoffs of 25% identity and 40% length. The level of identity was kept above 25% given that below this level we cannot assume the shared common ancestry of genes based on sequence data alone^55^. An equation that approximates the construction of a quartet gene tree assigned a confidence value to each reciprocal best blast pair of genes to determine if their relationship was orthologous or paralogous. Two-stage clustering (Markov cluster algorithm (MCL) and single linkage clustering (SLC)) was used to cluster orthologues across all 213 genomes so that a presence and absence distribution could be determined for all gene families. Gene families with a representative sequence in all 213 genomes were selected as core genes for the construction of a phylogenetic tree. This method supported a core of 73 genes (Supplementary Table 2; Supplementary Data, data set 1 for sequences), which was used in all phylogenetic inferences. The effect of fragmented genomes on the core genome was assessed by removing genomes with greater than 20, 50, 100, 200, 300, 400 and 500 contigs and inferring a core genome in each case (Supplementary Fig. 2).

### Assessing the robustness of core gene number and tree topology

We tested for the presence of 114 bacterial core marker genes^56^ in the gene sequences of each of the 213 genomes and found that, whereas no genome had a low number of predicted marker genes (range 96–111), the 4 genomes with fewer than 105 genes all had contig numbers less than 200. Furthermore, when we correlated the number of predicted core genes (out of 114) with contig number, the Spearman correlation value was very low (*ρ* value of 0.078; *P*-value=0.26). This shows that draft genomes with larger contig numbers do not have artificially low core gene numbers.

To investigate the effect of core gene number on robustness of phylogeny, we omitted some of the more peripherally related LAB from the analysis, namely, we omitted the *Atopobium*, *Kandleria*, *Olsenella* and *Lactococcus* species, and this resulted in a core genome of 121 genes. The resulting phylogeny was highly congruent with the 73 core gene phylogeny, and was also supported by equally high bootstrap values. We put back in *Lactococcus* and removed *Carnobacterium*, resulting in a core gene set of 117 genes. Similarly, the resulting phylogeny was highly congruent with the 73 core gene phylogeny, and was also supported by equally high bootstrap values.

### Calculation of ANI and TNI

The pair-wise ANI and TNI values across newly sequenced genomes were calculated according to methods proposed by Goris *et al*.^22^ and Chen *et al*.^23^, respectively. The frequency distributions of the ANI and TNI values of 3,730 published bacterial genomes were acquired from our previous report^23^.

### Phylogenetic analysis

To determine the placement of the *Lactobacillus* Genus complex and associated genera within the Bacterial kingdom, we used AMPHORA2 (ref. 57), a marker gene database used in the phylogenetic inference of prokaryotes, to identity 16 marker genes (Supplementary Table 4; data set S2 for gene sequences), out of a total of 31 possible marker genes, that were shared across 452 representative bacterial species (Supplementary Tables 3 and 4). We aligned the amino-acid sequences for each gene separately using MUSCLE v3.8.31 (ref. 58) and then constructed the maximum likelihood tree based on the concatenated alignment using the software RAxML with the PROTCATWAG model^59^.

A Maximum Likelihood phylogeny concentrating on the *Lactobacillus* Genus complex and associated genera was inferred from 73 core genes present in all 213 strains. Amino-acid sequences were aligned as above and the phylogeny was estimated using the PROTCATWAG model in RAxML v8.0.22 (ref. 59) and rooted using *Atopobium minutum* DSM 20586, *Olsenella uli* DSM 7084 and *Atopobium rimae* DSM 7090. Bootstrapping was carried out using 100 replicates and values are indicated on the nodes of the phylogeny.

### Prediction of glycolysis-related genes

A matrix with the presence/absence of the 10 core glycolytic genes across the 213 genomes was built using a combination of annotation querying and BLAST searching. When a gene was absent in one or more genomes, the result was confirmed with a tblastn^60^ search using *L. salivarius* query genes. In cases where a homologue was found using the blast approach, the sequence was retrieved and aligned with mafft^61^. Alignments were inspected to confirm similarity of the sequences.

We mined the genomes for the presence of phosphoglycerate mutase using the approach published by Foster *et al*.^62^ The query phosphoglycerate mutases from *E. coli* GpmA (dPGM; NCBI GI number 50402115) and *E. coli* GpmM (iPGM,; 586733) were aligned against the six-frame translations of the 213 draft genomes with tblastn. Hits with a bit score larger than 100 were considered as a PGM match.

### Bacteriocin prediction

BAGEL^63^ was utilized to mine genomes for potential bacteriocin operons; results were manually verified within Artemis^64^.

### Amino-acid pathway identification

Amino-acid pathways were investigated through the KEGG suite of tools^65^.

### CRISPR identification

CRISPR-Cas systems were identified using CRISPRFinder^45^ and manual curation of the results.

### Investigation of niche association

The 213 genomes were grouped into six niche categories in order to test for niche-specific associations in functional gene groups and genomic characteristics. The six niche categories are food (*n*=76), animal (*n*=56), plant (*n*=34), wine product (*n*=33), environment (*n*=7) and unknown (*n*=7). The niche category for each genome is shown in Supplementary Table 1. We applied Kruskal–Wallis tests and generated boxplots for visualization in order to determine trends among niches for 104 variables. These variables included all functional groups analysed in this study, MGEs (plasmids, phages and IS elements) and the following genomic parameters: genome size, gene number, contig number, GC content and sequencing depth. Statistics and visualization were carried out in R v3.1.1 (https://www.r-project.org/).

### Profiling of GHs and GTs

The detection and assignment of sequences to families of CAZymes was carried out using a two-step approach. HMMSCAN (from the HMMER package v3.1b1 (http://hmmer.org/)) was used to query hidden Markov models representing the signature domains of each CAZyme family, to predict potential GTs and GHs across the 213 genomes below a threshold cutoff of 1e-05. In a separate approach, genes that have the GH and GT enzyme configuration (EC) designation EC 3.2.1.X and EC 2.4.X.X, respectively, were pooled into a GT and GH database. BLASTp searches were used to predict potential GTs and GHs from the 213 genomes using a cutoff of 40% identity and 50% length with an *e*-value cutoff of 1e-05. Results from the Hidden Markov Model (HMM) and the blast approach were compared to determine if both approaches supported the predicted gene results. Common genes were retained and genes unique to one approach were screened against the Pfam 27.0 database to confirm the presence of GT/GH domains. Copy number of the verified GH/GT family were summarized in a heat map.

### Identifying carbohydrate tranporters

To predict genes involved in carbohydrate transport, we downloaded the protein database (go_20140614-seqdb.fasta.gz) from the Gene Ontology Consortium Database (http://archive.geneontology.org). A subset of this database was created by selecting all sequences that were annotated as carbohydrate transporters. Predicted genes from our study were blasted against this smaller database using BLASTP and genes involved in carbohydrate transport were selected using the thresholds, 40% identity, 50% coverage of query gene aligned and *e*-value <1e-05.

### General metabolism

To generate an overview of metabolism, we blasted all predicted genes against the STRING database v9 (ref. 66). The top hit for each gene (that is, lowest *e*-value) was used to assign a COG category after applying thresholds of 40% identity, 50% of query gene length aligned and *e*-value <1e-05. R v3.1.1 was used for reformatting and for generating the COG heat map.

### Identifying genes involved in stress response

The KEGG database was mined for gene products annotated as playing a part in stress responses. These were categorized into acid stress, oxidative stress, heat/DNA damage, cold stress, osmotic stress and bile tolerance. These genes were compiled into a database of 61,706 proteins. This database served to query (BLASTp) the predicted proteins encoded by the 213 genomes. Hits were considered stress response genes if their gene products displayed greater than 40% identity over 50% of the length of the KEGG stress response protein below an *e*-value of 1e-05. Copy number of the distribution of each of the stress-response proteins was summarized and visualized using a heat map in the R statistical package v3.1.1.

### Identification of ISs

To predict IS elements, Hidden Markov models representing 19 IS transposase families were downloaded from the TnpPred web service (http://www.mobilomics.cl). HMMSCAN (from the HMMER package v3.1b1) was used to query amino-acid sequences of predicted genes against the HMMs.

### Phage identification

Bacteriophage genes were annotated by BLASTP search against the NCBI protein database using cutoffs of 40% identity over 50% of the length with an *e*-value of <1e-05. To predict phage-specific genes, a string search of predefined phage functions was carried out on gene annotations. Phage functions that overlap with non-phage functions such as those involved in transcription and DNA metabolism are annotated as belonging to prophages and these genes were also included in the phage results.

### Plasmid identification

For each genome, contigs were blasted against an NCBI reference database of complete plasmid sequences. A group of contigs was identified as belonging to a plasmid if at least 25% of their combined length aligned to at least 25% of the plasmid at ⩾70% identity. These thresholds were determined empirically by adjusting alignment length and identity cutoffs until the strains in the data set that are known to have plasmids and those that are known to have no plasmids both gave correct predictions. All predicted genes belonging to plasmid-associated contigs were then blasted against the STRING database v9.1 (ref. 66) in order to assign COG categories.

### Analysis of LPXTG proteins, sortases and PGCs

Interproscan v. 5.44.0 with TIGRFAM 13.0 database with default parameters was used to search for conserved domains in the genomes^67^,^68^. Automatic pilus cluster search was performed using LOCP v. 1.0.0 with parameters ‘-P 1' and ‘-P_adj 0.05' (ref. 69). The LOCP output results were then curated. Both programmes were run on the amino-acid CDSs data. R v. 3.0.1 was used for managing and parsing the output data^70^.

### CEP identification and analysis

CEP sequences were identified in the genome sequences using two strategies. The first strategy involved a BLAST search using Subtilisin E as the search model. This returned 1,201 putative homologues. The second strategy used a HMM model for subtilisin as the search model and this returned 151 hits. Both panels of hits were further interrogated using the following strategy. First, the presence of the key catalytic residues was confirmed (Asp, His and Ser, in this order of occurrence) and the proteins binned by number of residues in the sequence. The panels were further rationalized using a HMM search model for domains identified in the only solved structure of an active CEP, the ScpA from *Streptococcus pyogenes*^38^. These searches included the DUF1034, which is equivalent to the Fn1 domain of ScpA; the CHU_C model corresponding to the Fn2 domain; the PA domain; SLAP, which is an S layer-anchoring domain; and a manual inspection for LPXTG derivative sequence. This screening identified 60 CEPs across the genome database. Each of these hits was in turn used as a BLAST search model to confirm no additional CEPs could be identified. These searches proved to be internally consistent with no additional CEPs identified.

## 

## Additional information

**Accession codes:** Whole-genome sequences generated in this study have been deposited in the GeneBank SRA database under accession codes ERX399734, ERX359753, ERX359727, ERX359700, ERX359702, ERX399735, ERX359711, ERX359782, ERX359757, ERX359713, ERX399738, SRX456282, SRX456246, ERX359728, SRX456283, ERX359701, SRX456357, ERX359717, SRX456279, ERX359733, SRX456280, ERX359783, SRX456248, ERX399737, ERX359723, ERX405609, ERX359760, ERX359761, SRX456251, SRX456278, SRX456296, ERX399739, ERX359699, ERX359689, SRX456230, ERX399740, SRX456252, ERX399741, ERX359694, ERX399742, SRX456340, SRX456233, SRX456245, SRX456344, ERX359721, SRX456225, ERX359756, SRX456341, ERX399745, SRX690302, SRX456227, ERX399746, ERX359710, ERX359739, SRX456237, ERX359704, ERX359706, SRX456285, ERX359697, ERX359692, ERX359719, SRX456305, ERX399748, SRX456290, ERX399747, ERX359759, ERX359709, ERX359725, ERX359738, SRX456277, ERX359732, SRX456255, ERX359758, ERX359712, ERX359737, ERX399749, ERX359691, SRX456228, ERX359764, SRX456360, SRX690303, SRX456258, ERX405610, ERX359755, SRX456259, ERX359778, ERX359729, ERX359740, SRX456262, SRX456239, ERX359741, SRX456311, ERX359714, ERX359693, SRX456314, SRX690301, SRX456316, ERX359742, ERX359724, ERX399750, ERX359707, SRX456362, ERX359763, SRX456318, ERX359690, ERX359754, SRX456264, ERX359734, ERX359735, ERX399751, SRX456349, ERX359747, ERX359744, ERX399753, ERX359745, ERX359748, SRX456266, ERX359743, ERX399754, ERX399755, ERX359736, SRX456325, ERX399756, ERX359703, SRX456268, SRX456291, SRX456229, ERX399758, SRX456365, ERX399759, ERX359751, ERX399760, ERX359726, SRX456366, SRX456269, ERX399762, SRX456342, SRX456327, ERX399763, ERX359752, SRX456293, ERX399764, SRX456329, ERX399765, SRX456240, ERX359746, ERX399766, SRX456270, SRX456370, SRX456353, SRX456242, SRX456271, ERX399768, SRX456367, SRX456272, SRX456354, ERX399769, ERX399770, ERX359767, ERX359762, SRX690300, ERX359768, SRX456331, ERX359770, ERX359771, ERX359772, ERX359773, ERX450947, SRX456334, SRX456335, ERX359776, SRX456337, ERX359779, SRX456274, ERX359780, ERX359731, SRX456275, SRX456244, SRX456339, ERX359765, SRX456369, ERX399772, ERX359766, ERX399771, SRX689743, SRX689746, SRX689747, SRX689748, SRX689749, SRX689750, SRX689751, SRX689752, SRX689754, SRX689755, SRX689756, ERX359705, ERX359698, ERX359708, ERX399773, and ERX359781.

**How to cite this article:** Sun, Z. *et al*. Expanding the biotechnology potential of lactobacilli through comparative genomics of 213 strains and associated genera. *Nat. Commun.* 6:8322 doi: 10.1038/ncomms9322 (2015).

## Supplementary Material

Supplementary Information Supplementary Figures 1-25, Supplementary Tables 1-11, Supplementary Notes 1-11 and Supplementary References

Supplementary Data 1 Sequences of core genes and protein sequences across examined strains of lactobacilli

## References

[b1] Salvetti E., Torriani S. & Felis G. E. The genus *Lactobacillus*: a taxonomic update. Probiotics Antimic. Proteins 4, 217–226 (2012). 10.1007/s12602-012-9117-8 26782181

[b2] Bernardeau M., Guguen M. & Vernoux J. P. Beneficial lactobacilli in food and feed: long-term use, biodiversity and proposals for specific and realistic safety assessments. FEMS Microbiol. Rev. 30, 487–513 (2006). 16774584 10.1111/j.1574-6976.2006.00020.x

[b3] Bernardeau M., Vernoux J. P., Henri-Dubernet S. & Gueguen M. Safety assessment of dairy microorganisms: the Lactobacillus genus. Int. J. Food Microbiol. 126, 278–285 (2008). 17889388 10.1016/j.ijfoodmicro.2007.08.015

[b4] Klaenhammer T. R., Kleerebezem M., Kopp M. V. & Rescigno M. The impact of probiotics and prebiotics on the immune system. Nat. Rev. Immunol. 12, 728–734 (2012). 23007572 10.1038/nri3312

[b5] Hill C. . Expert consensus document: The International Scientific Association for Probiotics and Prebiotics consensus statement on the scope and appropriate use of the term probiotic. Nat. Rev. Gastroenterol. Hepatol. 11, 506–514 (2014). 24912386 10.1038/nrgastro.2014.66

[b6] Saxelin M. Probiotic formulations and applications, the current probiotics market, and changes in the marketplace: a European perspective. Clin. Infect. Dis. 46, (Suppl 2): S76–S79 discussion S144-151 (2008). 18181728 10.1086/523337

[b7] Badel S., Bernardi T. & Michaud P. New perspectives for Lactobacilli exopolysaccharides. Biotechnol. Adv. 29, 54–66 (2011). 20807563 10.1016/j.biotechadv.2010.08.011

[b8] Marco M. L. . Convergence in probiotic *Lactobacillus* gut-adaptive responses in humans and mice. ISME J. 4, 1481–1484 (2010). 20505752 10.1038/ismej.2010.61

[b9] Mohamadzadeh M., Duong T., Sandwick S. J., Hoover T. & Klaenhammer T. R. Dendritic cell targeting of *Bacillus anthracis* protective antigen expressed by *Lactobacillus acidophilus* protects mice from lethal challenge. Proc. Natl Acad. Sci. USA 106, 4331–4336 (2009). 19246373 10.1073/pnas.0900029106 PMC2647975

[b10] Bermudez-Humaran L. G. . Engineering lactococci and lactobacilli for human health. Curr. Opin. Microbiol. 16, 278–283 (2013). 23850097 10.1016/j.mib.2013.06.002

[b11] Reddy G., Altaf M., Naveena B. J., Venkateshwar M. & Kumar E. V. Amylolytic bacterial lactic acid fermentation - a review. Biotechnol. Adv. 26, 22–34 (2008). 17884326 10.1016/j.biotechadv.2007.07.004

[b12] Castillo Martinez F. A. . Lactic acid properties, production and applications: a review. Trends Food Sci. Tech. 30, 70–83 (2013).

[b13] Hammes W. P. & Vogel R. F. in The Genera of Lactic Acid Bacteria Vol. 2, eds Wood B. J. B., Holzapfel W. H. 19–54Blackie Academic and Professional (1995).

[b14] Collins M. D. . Phylogenetic analysis of the genus *Lactobacillus* and related lactic-acid bacteria as determined by reverse-transcriptase sequencing of 16s ribosomal-RNA. FEMS Microbiol. Lett. 77, 5–12 (1991).

[b15] Canchaya C., Claesson M. J., Fitzgerald G. F., van Sinderen D. & O'Toole P. W. Diversity of the genus *Lactobacillus* revealed by comparative genomics of five species. Microbiology 152, 3185–3196 (2006). 17074890 10.1099/mic.0.29140-0

[b16] Kant R., Blom J., Palva A., Siezen R. J. & de Vos W. M. Comparative genomics of *Lactobacillus*. Microb. Biotechnol 4, 323–332 (2011). 21375712 10.1111/j.1751-7915.2010.00215.x PMC3818991

[b17] Zhang Z. G., Ye Z. Q., Yu L. & Shi P. Phylogenomic reconstruction of lactic acid bacteria: an update. BMC Evol. Biol. 11, 1 (2011). 21194491 10.1186/1471-2148-11-1 PMC3024227

[b18] Makarova K. . Comparative genomics of the lactic acid bacteria. Proc. Natl Acad. Sci. USA 103, 15611–15616 (2006). 17030793 10.1073/pnas.0607117103 PMC1622870

[b19] Claesson M. J., Van Sinderen D. & O'Toole P. W. *Lactobacillus* phylogenomics - towards a reclassification of the genus. Int. J. Syst. Evol. Microbiol. 58, 2945–2954 (2008). 19060088 10.1099/ijs.0.65848-0

[b20] Felis G. E., Molenaar D., Dellaglio F. & van Hylckama Vlieg J. E. Dichotomy in post-genomic microbiology. Nat. Biotechnol. 25, 848–849 (2007). 17687355 10.1038/nbt0807-848

[b21] Chan J. Z., Halachev M. R., Loman N. J., Constantinidou C. & Pallen M. J. Defining bacterial species in the genomic era: insights from the genus Acinetobacter. BMC. Microbiol. 12, 302 (2012). 23259572 10.1186/1471-2180-12-302 PMC3556118

[b22] Goris J. . DNA-DNA hybridization values and their relationship to whole-genome sequence similarities. Int. J. Syst. Evol. Microbiol. 57, 81–91 (2007). 17220447 10.1099/ijs.0.64483-0

[b23] Chen J. . SISP: a fast species identification system for prokaryotes based on total nucleotide identity of whole genome sequence. Infect. Dis. Transl. Med. 1, 30–55 (2015).

[b24] Kandler O. Carbohydrate metabolism in lactic acid bacteria. Antonie Van Leeuwenhoek 49, 209–224 (1983). 6354079 10.1007/BF00399499

[b25] Leisner J. J. . Alpha-Chitinase activity among lactic acid bacteria. Syst. Appl. Microbiol. 31, 151–156 (2008). 18424038 10.1016/j.syapm.2008.03.003

[b26] Rodríguez Jiménez E. Dextranase in sugar industry: a review. Sugar Tech 11, 124–134 (2009).

[b27] Fujita K. . Identification and molecular cloning of a novel glycoside hydrolase family of core 1 type O-glycan-specific endo-alpha-N-acetylgalactosaminidase from *Bifidobacterium longum*. J. Biol. Chem. 280, 37415–37422 (2005). 16141207 10.1074/jbc.M506874200

[b28] Shallom D., Golan G., Shoham G. & Shoham Y. Effect of dimer dissociation on activity and thermostability of the alpha-glucuronidase from *Geobacillus stearothermophilus*: dissecting the different oligomeric forms of family 67 glycoside hydrolases. J. Bacteriol. 186, 6928–6937 (2004). 15466046 10.1128/JB.186.20.6928-6937.2004 PMC522207

[b29] Beg Q. K., Kapoor M., Mahajan L. & Hoondal G. S. Microbial xylanases and their industrial applications: a review. Appl. Microbiol. Biotechnol. 56, 326–338 (2001). 11548999 10.1007/s002530100704

[b30] Katayama T. . Molecular cloning and characterization of *Bifidobacterium bifidum* 1,2-alpha-L-fucosidase (AfcA), a novel inverting glycosidase (glycoside hydrolase family 95). J. Bacteriol. 186, 4885–4893 (2004). 15262925 10.1128/JB.186.15.4885-4893.2004 PMC451662

[b31] Wang L. & Wise M. J. Glycogen with short average chain length enhances bacterial durability. Naturwissenschaften 98, 719–729 (2011). 21808975 10.1007/s00114-011-0832-x

[b32] Bergman M., Del Prete G., van Kooyk Y. & Appelmelk B. *Helicobacter pylori* phase variation, immune modulation and gastric autoimmunity. Nat. Rev. Microbiol. 4, 151–159 (2006). 16415930 10.1038/nrmicro1344

[b33] Navarre W. W. & Schneewind O. Surface proteins of Gram-positive bacteria and mechanisms of their targeting to the cell wall envelope. Microbiol. Mol. Biol. Rev. 63, 174–229 (1999). 10066836 10.1128/mmbr.63.1.174-229.1999 PMC98962

[b34] Kankainen M. . Comparative genomic analysis of Lactobacillus rhamnosus GG reveals pili containing a human- mucus binding protein. Proc. Natl Acad. Sci. USA 106, 17193–17198 (2009). 19805152 10.1073/pnas.0908876106 PMC2746127

[b35] von Ossowski I. . Mucosal adhesion properties of the probiotic *Lactobacillus rhamnosus* GG SpaCBA and SpaFED pilin subunits. Appl. Environ. Microbiol. 76, 2049–2057 (2010). 20118368 10.1128/AEM.01958-09 PMC2849237

[b36] Ardita C. S. . Epithelial adhesion mediated by pilin SpaC is required for *Lactobacillus rhamnosus* GG-induced cellular responses. Appl. Environ. Microbiol. 80, 5068–5077 (2014). 24928883 10.1128/AEM.01039-14 PMC4135752

[b37] Siezen R. J. Multi-domain cell-envelope proteinases of lactic acid bacteria. Antonie Van Leeuwenhoek 76, 139–155 (1999). 10532376

[b38] Kagawa T. F. . Model for substrate interactions in C5a peptidase from *Streptococcus pyogenes*: a 1.9A crystal structure of the active form of ScpA. J. Mol. Biol. 386, 754–772 (2009). 19152799 10.1016/j.jmb.2008.12.074

[b39] von Schillde M. A. . Lactocepin secreted by Lactobacillus exerts anti-inflammatory effects by selectively degrading proinflammatory chemokines. Cell Host Microbe 11, 387–396 (2012). 22520466 10.1016/j.chom.2012.02.006

[b40] Barrangou R. . CRISPR provides acquired resistance against viruses in prokaryotes. Science 315, 1709–1712 (2007). 17379808 10.1126/science.1138140

[b41] Barrangou R. & Marraffini L. A. CRISPR-Cas systems: prokaryotes upgrade to adaptive immunity. Mol. Cell 54, 234–244 (2014). 24766887 10.1016/j.molcel.2014.03.011 PMC4025954

[b42] Jiang W., Bikard D., Cox D., Zhang F. & Marraffini L. A. RNA-guided editing of bacterial genomes using CRISPR-Cas systems. Nat. Biotechnol. 31, 233–239 (2013). 23360965 10.1038/nbt.2508 PMC3748948

[b43] Sander J. D. & Joung J. K. CRISPR-Cas systems for editing, regulating and targeting genomes. Nat. Biotechnol. 32, 347–355 (2014). 24584096 10.1038/nbt.2842 PMC4022601

[b44] Oh J. H. & van Pijkeren J. P. CRISPR-Cas9-assisted recombineering in Lactobacillus reuteri. Nucleic Acids Res. 42, e131 (2014). 25074379 10.1093/nar/gku623 PMC4176153

[b45] Grissa I., Vergnaud G. & Pourcel C. The CRISPRdb database and tools to display CRISPRs and to generate dictionaries of spacers and repeats. BMC Bioinformatics 8, 172 (2007). 17521438 10.1186/1471-2105-8-172 PMC1892036

[b46] Chylinski K., Makarova K. S., Charpentier E. & Koonin E. V. Classification and evolution of type II CRISPR-Cas systems. Nucleic Acids Res. 42, 6091–6105 (2014). 24728998 10.1093/nar/gku241 PMC4041416

[b47] Jinek M. . A programmable dual-RNA-guided DNA endonuclease in adaptive bacterial immunity. Science 337, 816–821 (2012). 22745249 10.1126/science.1225829 PMC6286148

[b48] Raftis E. J., Forde B. M., Claesson M. J. & O'Toole P. W. Unusual genome complexity in *Lactobacillus salivarius* JCM1046. BMC Genomics 15, 771 (2014). 25201645 10.1186/1471-2164-15-771 PMC4165912

[b49] Li Y. . Distribution of megaplasmids in *Lactobacillus salivarius* and other lactobacilli. J. Bacteriol. 189, 6128–6139 (2007). 17586640 10.1128/JB.00447-07 PMC1951925

[b50] Chain P. S. . Genomics. Genome project standards in a new era of sequencing. Science 326, 236–237 (2009). 19815760 10.1126/science.1180614 PMC3854948

[b51] Delcher A. L., Bratke K. A., Powers E. C. & Salzberg S. L. Identifying bacterial genes and endosymbiont DNA with Glimmer. Bioinformatics 23, 673–679 (2007). 17237039 10.1093/bioinformatics/btm009 PMC2387122

[b52] Tatusov R. L. . The COG database: an updated version includes eukaryotes. BMC Bioinformatics 4, 41 (2003). 12969510 10.1186/1471-2105-4-41 PMC222959

[b53] Kanehisa M. . Data, information, knowledge and principle: back to metabolism in KEGG. Nucleic Acids Res. 42, D199–D205 (2014). 24214961 10.1093/nar/gkt1076 PMC3965122

[b54] Yu C., Zavaljevski N., Desai V. & Reifman J. QuartetS: a fast and accurate algorithm for large-scale orthology detection. Nucleic Acids Res. 39, e88 (2011). 21572104 10.1093/nar/gkr308 PMC3141274

[b55] Chung S. Y. & Subbiah S. A structural explanation for the twilight zone of protein sequence homology. Structure 4, 1123–1127 (1996). 8939745 10.1016/s0969-2126(96)00119-0

[b56] Wu D., Jospin G. & Eisen J. A. Systematic identification of gene families for use as "markers" for phylogenetic and phylogeny-driven ecological studies of bacteria and archaea and their major subgroups. PLoS One 8, e77033 (2013). 24146954 10.1371/journal.pone.0077033 PMC3798382

[b57] Wu M. & Scott A. J. Phylogenomic analysis of bacterial and archaeal sequences with AMPHORA2. Bioinformatics 28, 1033–1034 (2012). 22332237 10.1093/bioinformatics/bts079

[b58] Edgar R. C. MUSCLE: multiple sequence alignment with high accuracy and high throughput. Nucleic Acids Res. 32, 1792–1797 (2004). 15034147 10.1093/nar/gkh340 PMC390337

[b59] Stamatakis A. RAxML version 8: a tool for phylogenetic analysis and post-analysis of large phylogenies. Bioinformatics 30, 1312–1313 (2014). 24451623 10.1093/bioinformatics/btu033 PMC3998144

[b60] Altschul S. F., Gish W., Miller W., Myers E. W. & Lipman D. J. Basic local alignment search tool. J. Mol. Biol. 215, 403–410 (1990). 2231712 10.1016/S0022-2836(05)80360-2

[b61] Katoh K. & Toh H. Recent developments in the MAFFT multiple sequence alignment program. Brief. Bioinform. 9, 286–298 (2008). 18372315 10.1093/bib/bbn013

[b62] Foster J. M. . Evolution of bacterial phosphoglycerate mutases: non-homologous isofunctional enzymes undergoing gene losses, gains and lateral transfers. PLoS One 5, e13576 (2010). 21187861 10.1371/journal.pone.0013576 PMC2964296

[b63] de Jong A., van Heel A. J., Kok J. & Kuipers O. P. BAGEL2: mining for bacteriocins in genomic data. Nucleic Acids Res. 38, W647–W651 (2010). 20462861 10.1093/nar/gkq365 PMC2896169

[b64] Rutherford K. . Artemis: sequence visualization and annotation. Bioinformatics 16, 944–945 (2000). 11120685 10.1093/bioinformatics/16.10.944

[b65] Moriya Y., Itoh M., Okuda S., Yoshizawa A. C. & Kanehisa M. KAAS: an automatic genome annotation and pathway reconstruction server. Nucleic Acids Res. 35, W182–W185 (2007). 17526522 10.1093/nar/gkm321 PMC1933193

[b66] Franceschini A. . STRING v9.1: protein-protein interaction networks, with increased coverage and integration. Nucleic Acids Res. 41, D808–D815 (2013). 23203871 10.1093/nar/gks1094 PMC3531103

[b67] Haft D. H., Selengut J. D. & White O. The TIGRFAMs database of protein families. Nucleic Acids Res. 31, 371–373 (2003). 12520025 10.1093/nar/gkg128 PMC165575

[b68] Quevillon E. . InterProScan: protein domains identifier. Nucleic Acids Res. 33, W116–W120 (2005). 15980438 10.1093/nar/gki442 PMC1160203

[b69] Plyusnin I., Holm L. & Kankainen M. LOCP—locating pilus operons in gram-positive bacteria. Bioinformatics 25, 1187–1188 (2009). 19261721 10.1093/bioinformatics/btp127

[b70] Team R. C. R. A Language and Environment for Statistical Computing R Foundation for Statistical Computing (2013).

