# Supplementary material for: Expanding the biotechnology potential of lactobacilli through comparative genomics of 213 strains and associated genera
Source: Nat Commun. 2015 Sep 29;6:8322. doi: 10.1038/ncomms9322 (PMC4667430; doi:10.1038/ncomms9322)
Supplement: Supplementary Information — Supplementary Figures 1-25, Supplementary Tables 1-11, Supplementary Notes 1-11 and Supplementary References [file ncomms9322-s1.pdf]

Supplementary Figures

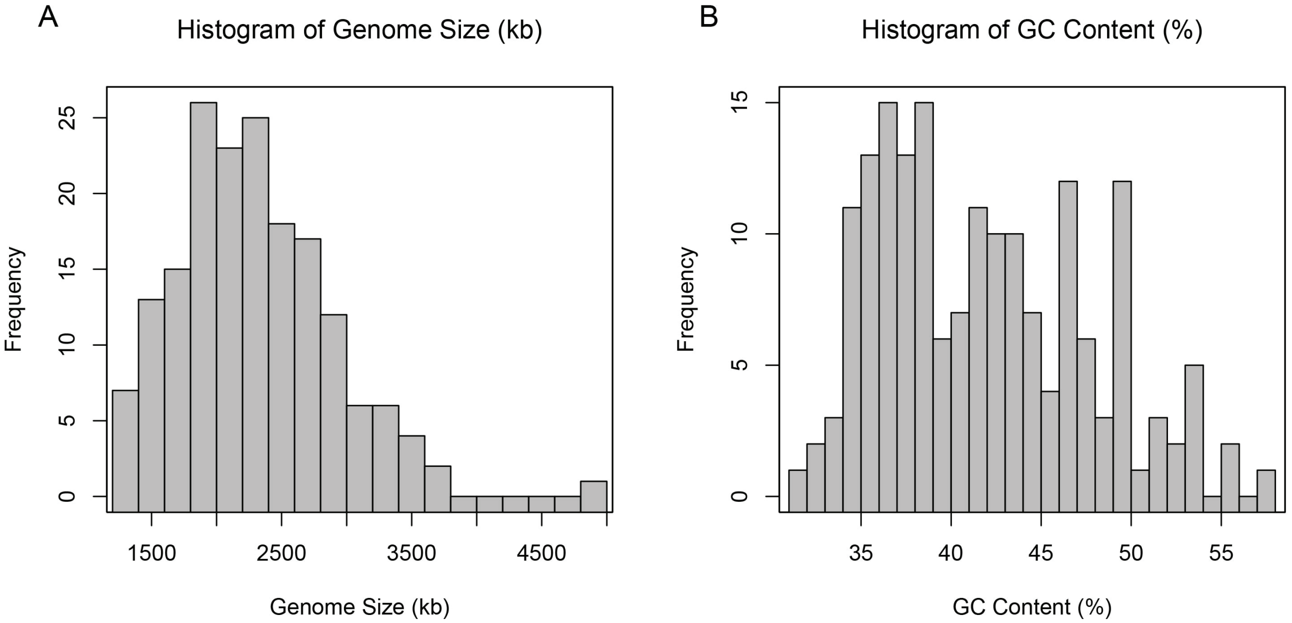

Supp. Figure 1. Histograms of genome size distribution (A) and GC% (B) for 175 *Lactobacillus* genomes.

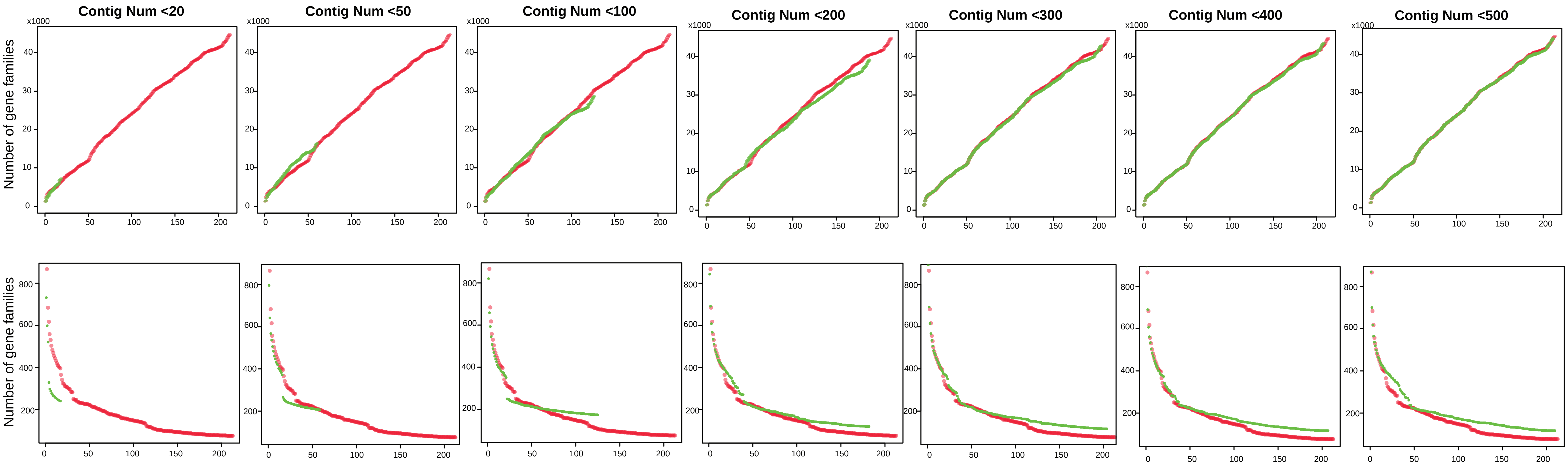

Supp. Figure 2. Sizes of the pan-genome (top) and core-genomes (bottom) in all 213 genomes (red) and in all genomes with less than 20, 50, 100, 200, 300, 400 and 500 contigs (green).

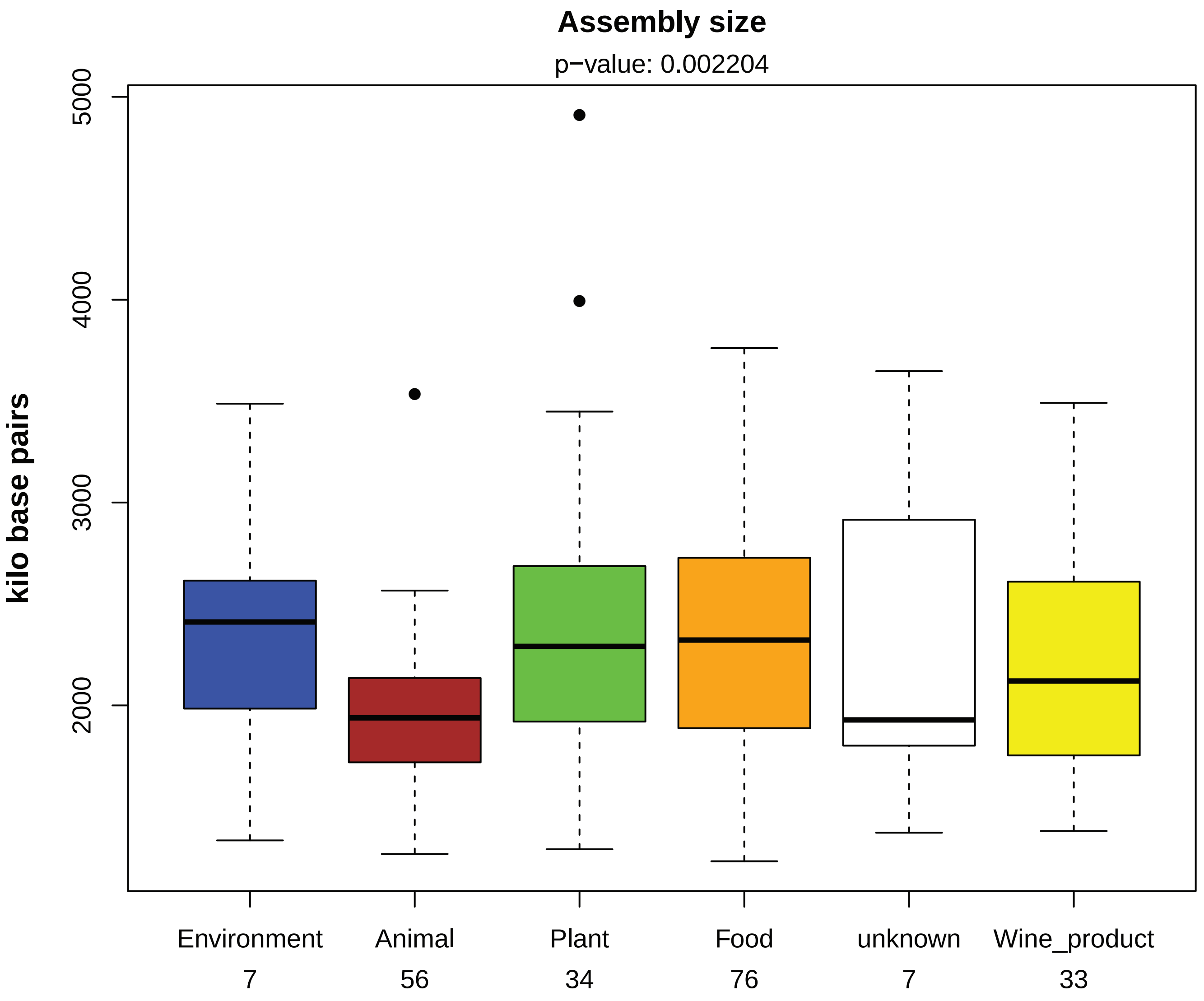

Supp. Figure 3. Analysis of genome assembly size as a function of niche. Niche categories are plotted on the x-axis and genome assembly size in kilobase pairs is plotted on the y-axis. Box-plots represent a five-point summary of the data in the following order (from bottom to top); minimum, first quartile, median, third quartile and maximum. Outliers are represented as individual points above or below the boxplot.

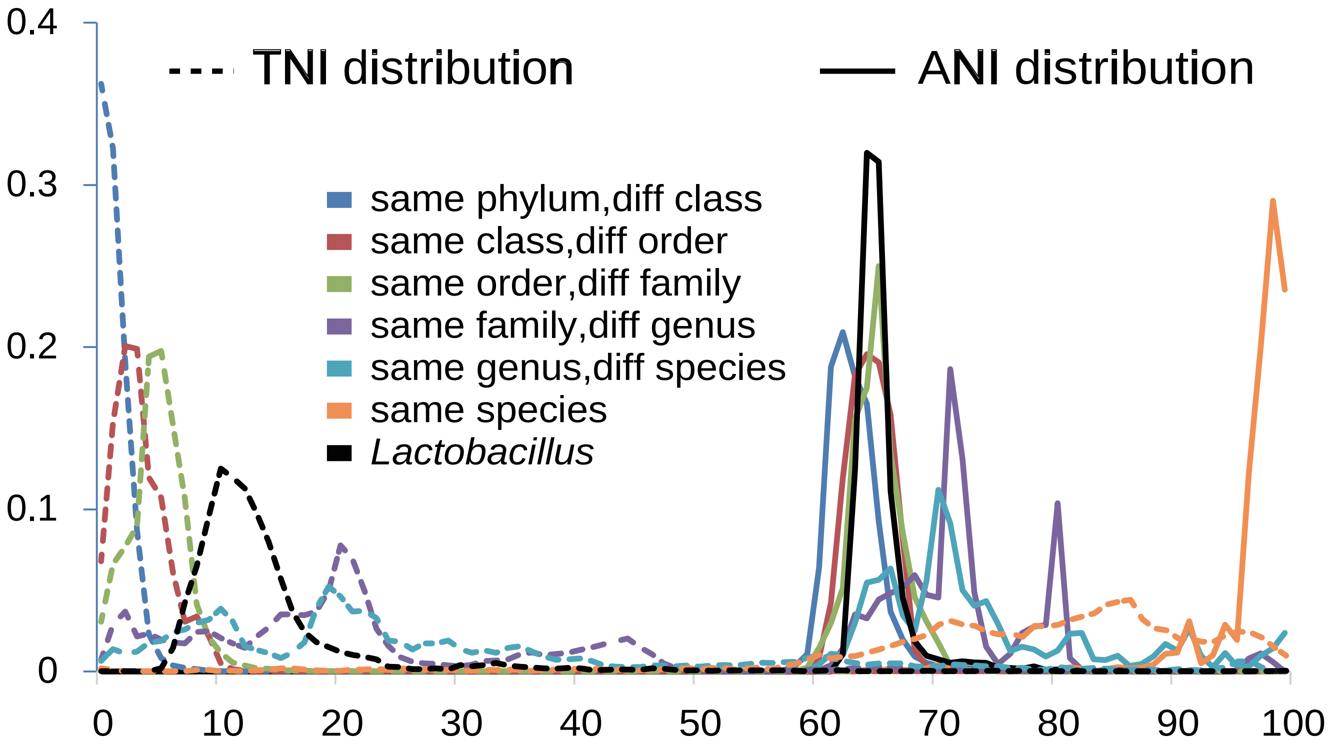

Supp. Figure 4. Frequency distribution of ANI and TNI values for the *Lactobacillus* species compared to those of traditionally defined taxonomic units. The black lines indicate the frequency distribution of values for the lactobacilli, which revealed lower values for both ANI and TNI than the majority of strains within the same family but in different genera.

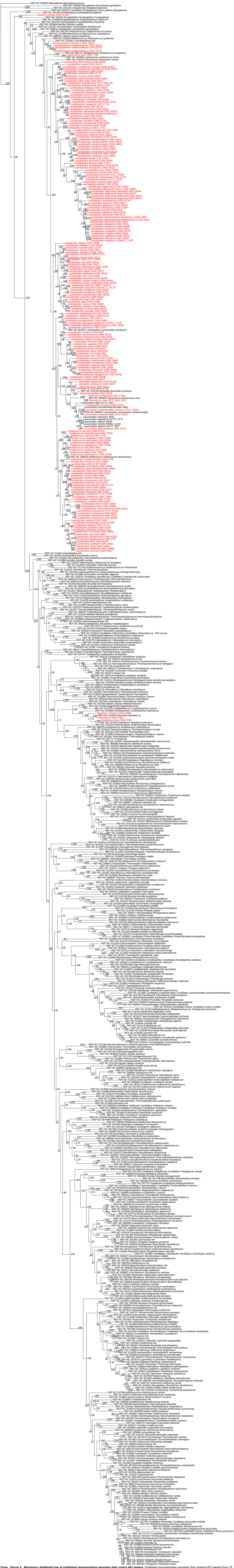

Supp. Figure 5. Maximum Likelihood tree of published representative genomes that covers 452 genera. Published representative genomes that covered 452 genera from 26 phyla, as well as the 213 genomes sequenced in this research. The tree was built based on the concatenated amino acid sequences of 16 marker genes by using RAxML with 100 bootstrap iterations. The numbers at nodes are bootstrap values and the genomes included in this study were indicated by red font.

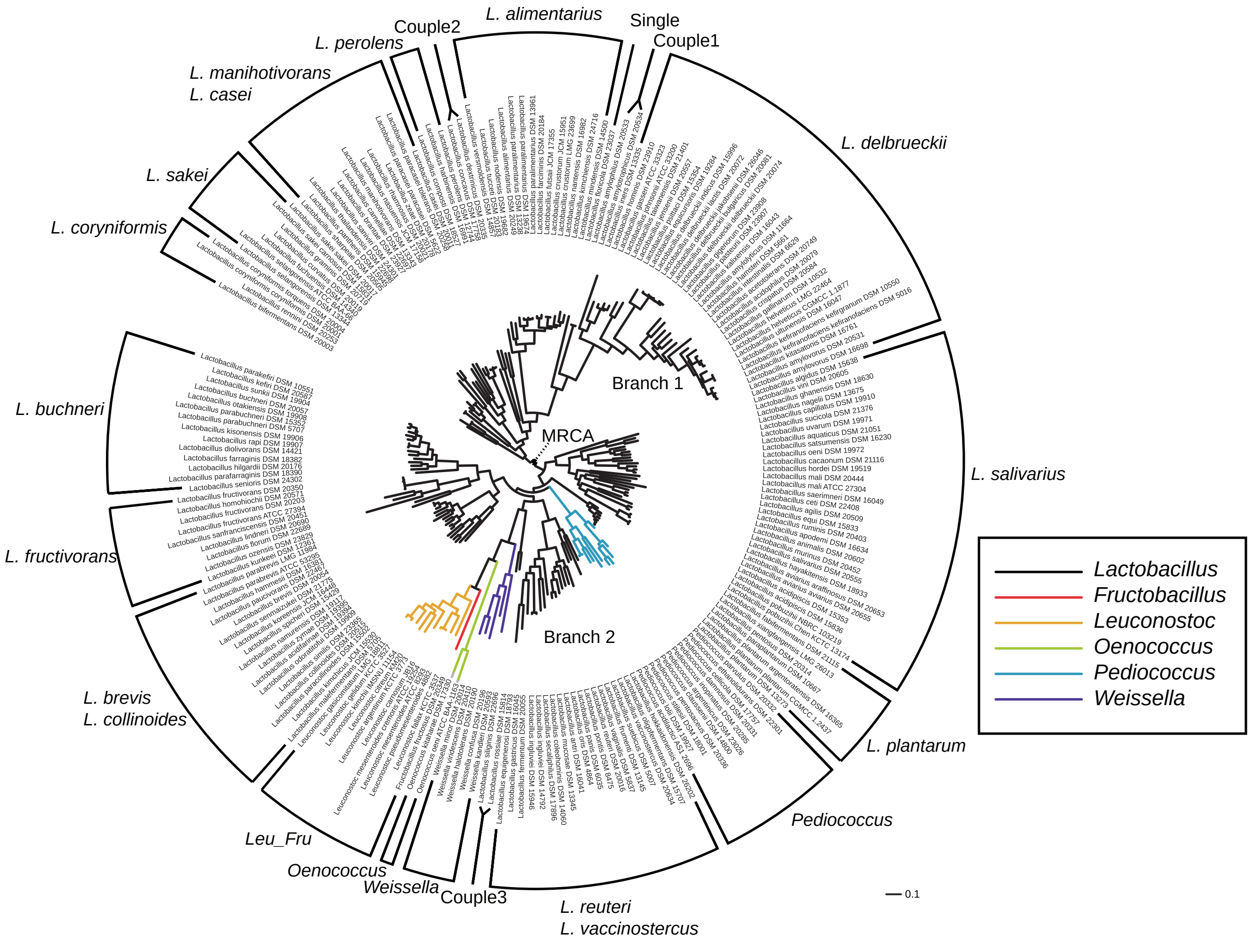

Supp. Figure 6. Maximum likelihood tree of strains of the *Lactobacillus* Genus Complex based on 73 core genes. The branch colors indicate different genera.

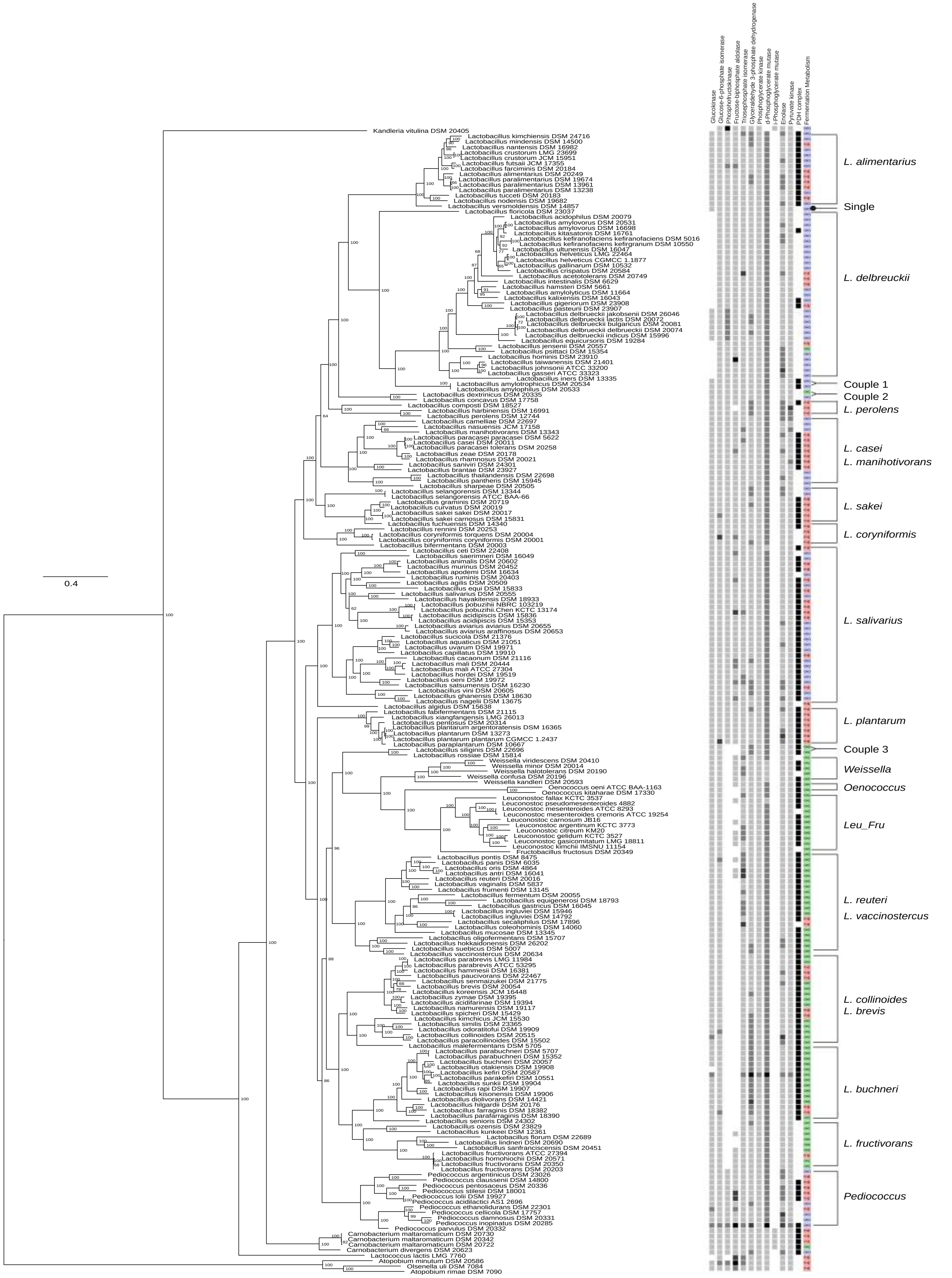

Supp. Figure 7. Distribution of glycolytic and pyruvate dehydrogenase genes across 213 lactobacilli and related species. The distribution of phosphoglycerate mutase is discriminated by the presence of genes encoding the cofactor-dependent (d) or the cofactor-independent (i) isofunctional enzymes. For all 10 core glycolytic enzymes, gene distribution is indicated in grey-scale from absence (white) to presence of 4 gene copies (black). For the pyruvate dehydrogenase operon (4 genes), presence of a functional complex is indicated in black, and absence of a functional complex in white. The fermentation metabolism phenotype is indicated as OHO: obligately homofermentative (purple), FHE: facultatively heterofermentative (pink) or OHE: obligately heterofermentative (green).

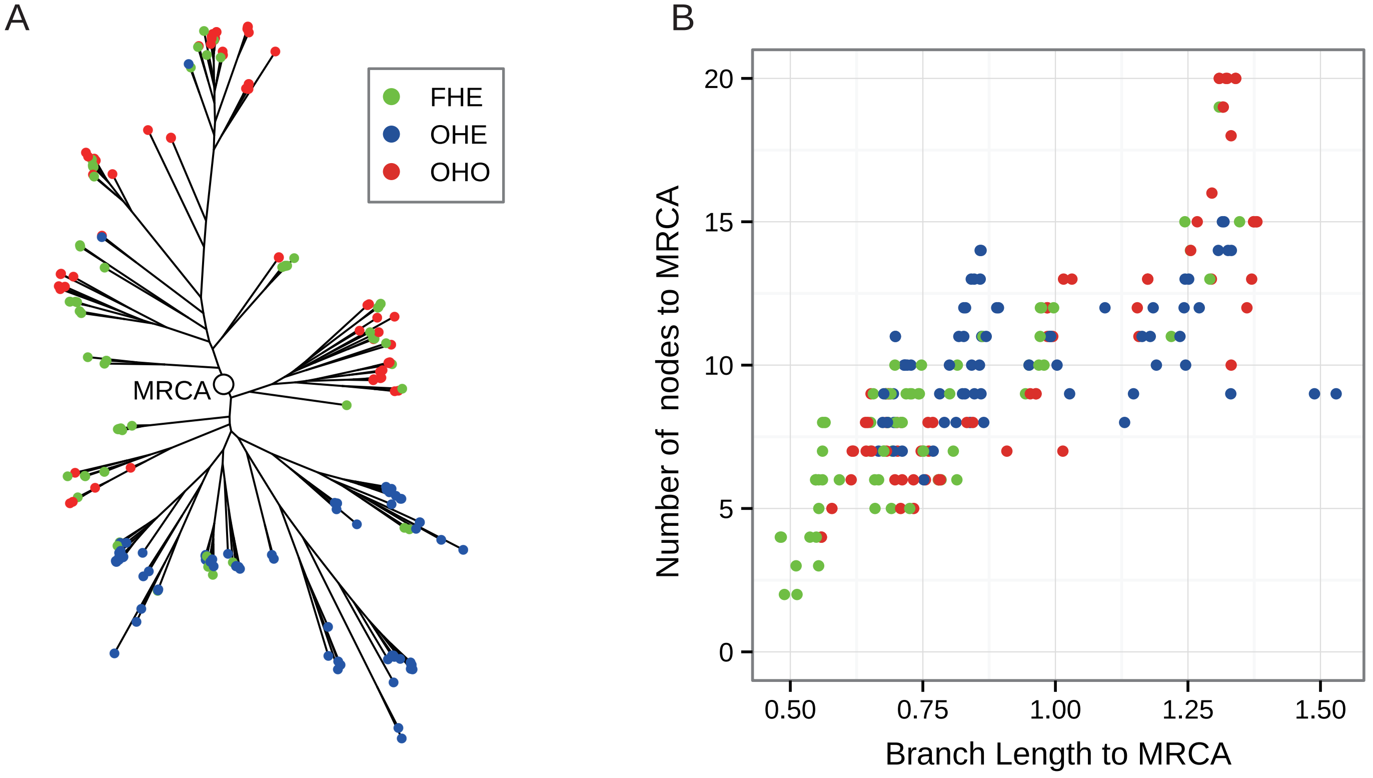

Supp. Figure 8. Evolution of carbohydrate metabolism in the *Lactobacillus* Genus Complex. A) Maximum likelihood tree of 204 strains of the *Lactobacillus* Genus Complex based on concatenated amino acid sequence of 73 core genes. The tree was built using RAxML with 100 bootstrap iterations. B) The number of nodes and the branch lengths to the MRCA for each strain/genome. The color of the branches in panel A and the dots in panel B indicate different fermentation types, with green representing FHE, blue representing OHE and red representing OHO.

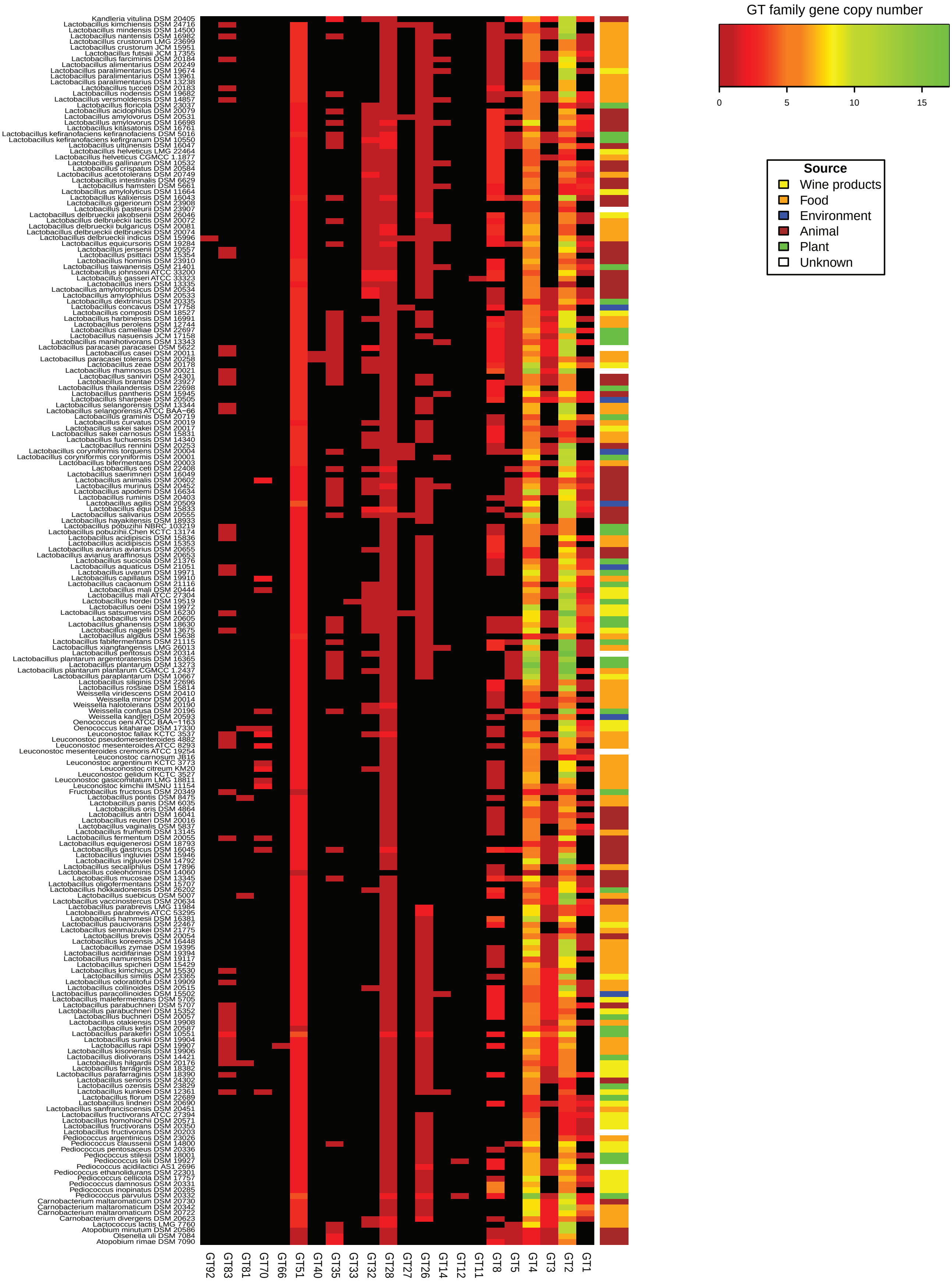

Supp. Figure 9. Heatmap of the distribution and abundance of glycosyl transferase families across the *Lactobacillus* Genus Complex and associated genera. Gene copy number of each of the 22 represented GT family members is indicated by the color key ranging from black (absent) to green. Strains are graphed in the same order from top to bottom as they appear top to bottom in the phylogeny (Fig. 2) with the isolation source of each strain indicated by the color bar at the top of the heat-map.

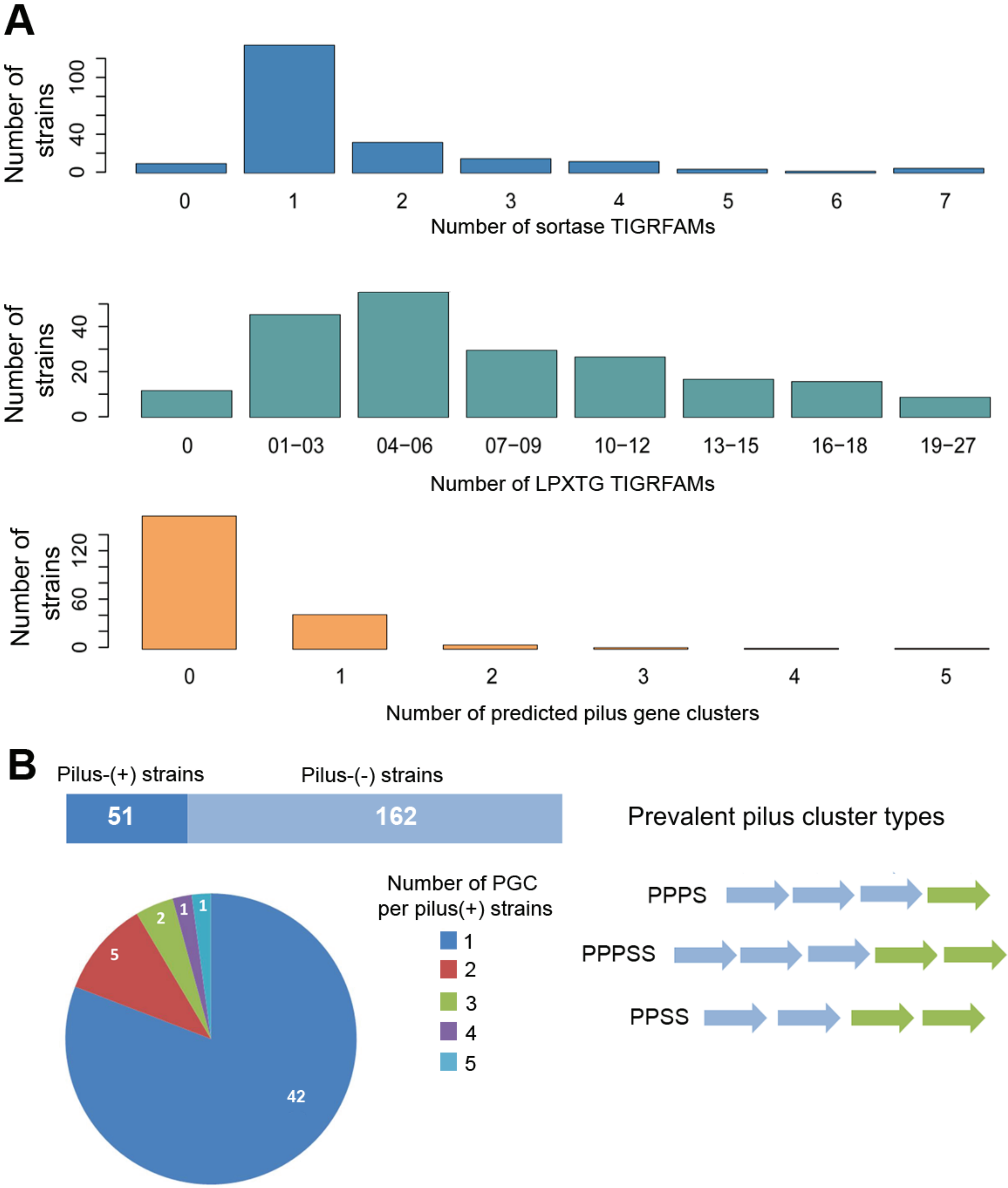

Supp. Figure 10. Distribution of LPXTG proteins, sortases and pilus gene clusters among the 213 genomes analysed. Panel A shows the pilus gene clusters (PGCs) that were found in 24% of all analyzed genomes and had prevalently one of the four types illustrated in Panel B Legend: green arrow = sortase gene; blue arrow = pilin gene.

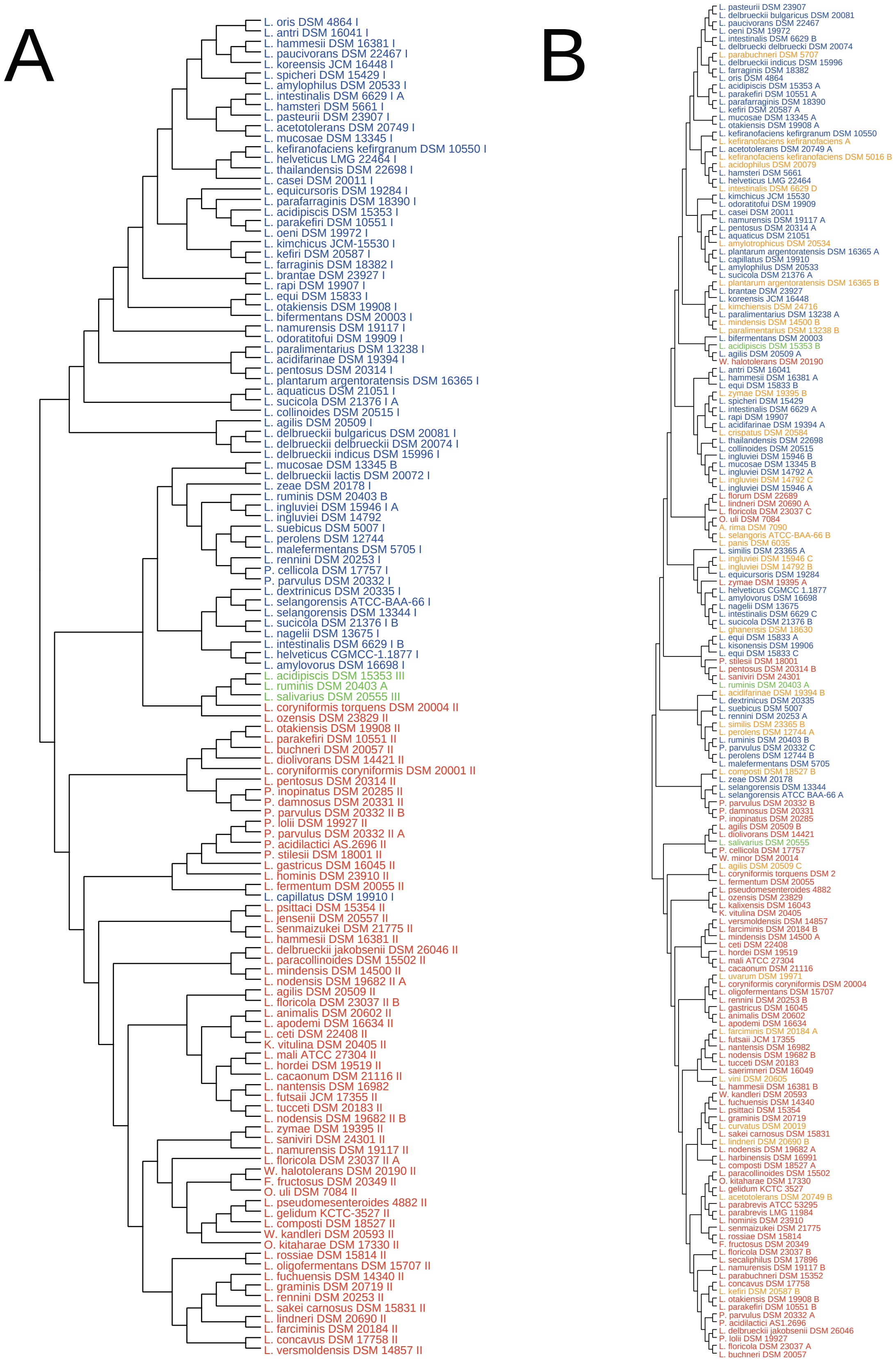

Supp. Figure 11. Comparative analysis of core CRISPR elements. The tree in panel A is derived from an alignment of the sequence of the universal Cas protein, Cas1, to create a phylogenetic tree showing the relatedness of all CRISPR-Cas systems in lactobacilli and closely related organisms (see Fig. 5A). The strain designation is followed by I, II, or III, corresponding to the respective CRISPR-Cas system type, using blue, red and green for Type I, II and III systems, respectively. Undefined systems are represented in orange. When multiple Cas1 proteins were found within a genome, they were differentiated by a letter. The tree in panel B is derived from an alignment of the CRISPR repeat sequences. All strain names correlate with the master CRISPR table (Supplementary Table 7). When a strain had multiple CRISPR repeats, they were given different letters to distinguish the repeats.

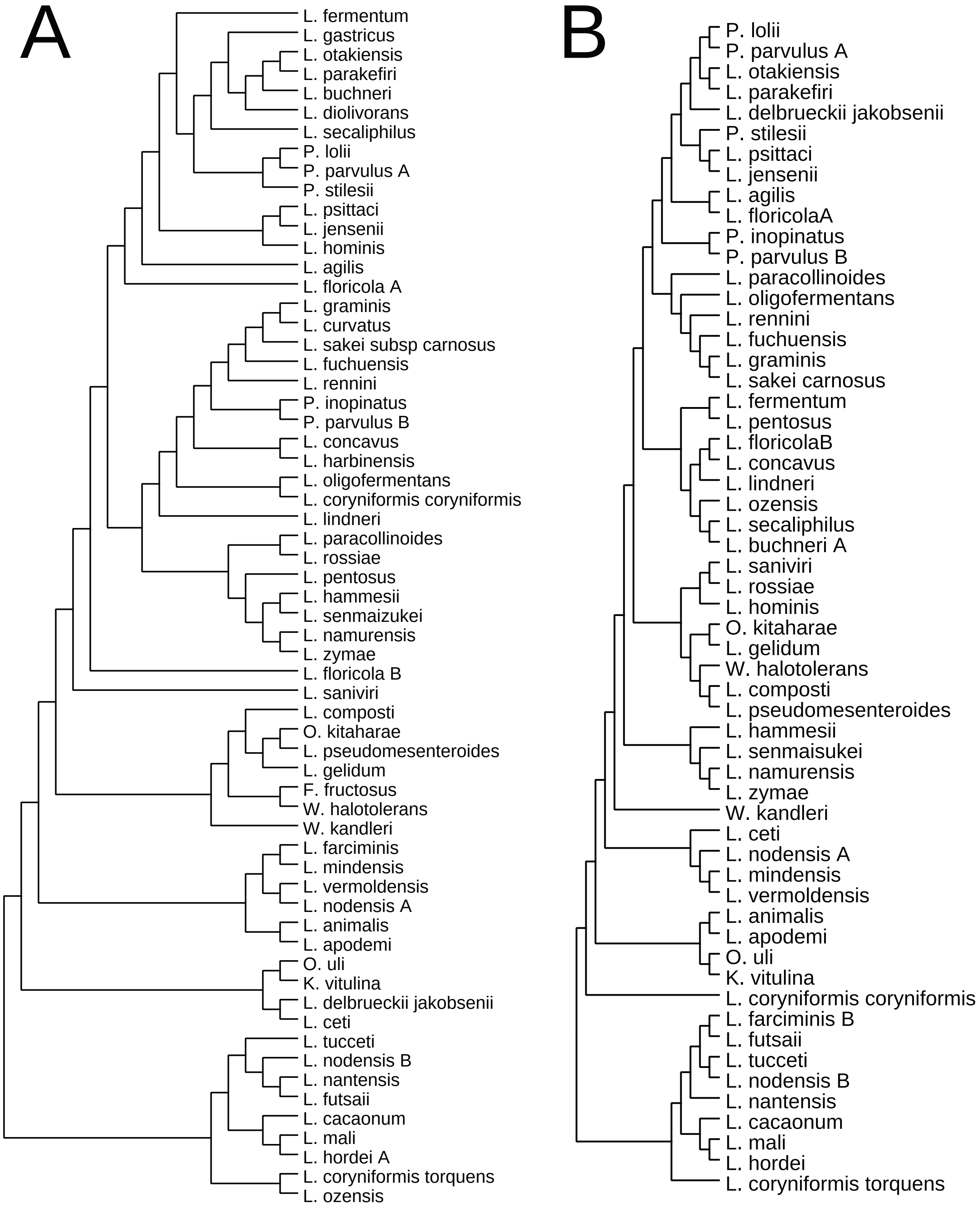

Supp. Figure 12. Comparative analysis of Type II CRISPR-Cas systems. The tree in panel A is derived from an alignment of the sequence of the Type II signature Cas protein, Cas9, to create a phylogenetic tree showing the relatedness of Cas9 proteins from Type II-A and II-C systems (see Fig. 5B). The tree in panel B is derived from an alignment of the predicted tracrRNA sequences for Type II-A systems.

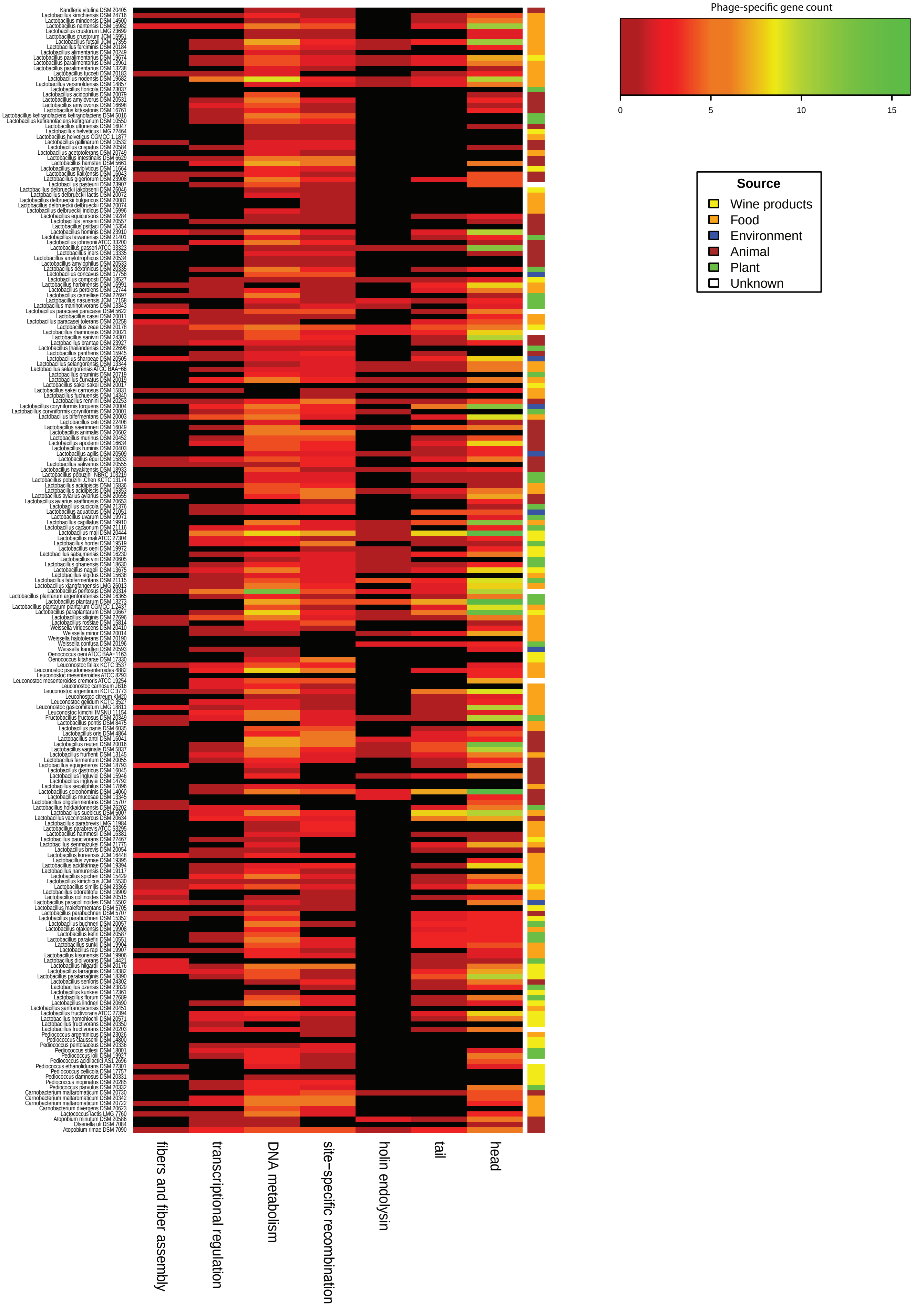

Supp. Figure 13. Heatmap of the distribution of 7 phage functional categories over the 213 genomes. The order of rows, from top to bottom, follows the order of genomes in the phylogenetic tree in Fig. 2 from top to bottom. The color key shows a gradation in color from black to red to yellow to green representing gene counts from 0 to 16.

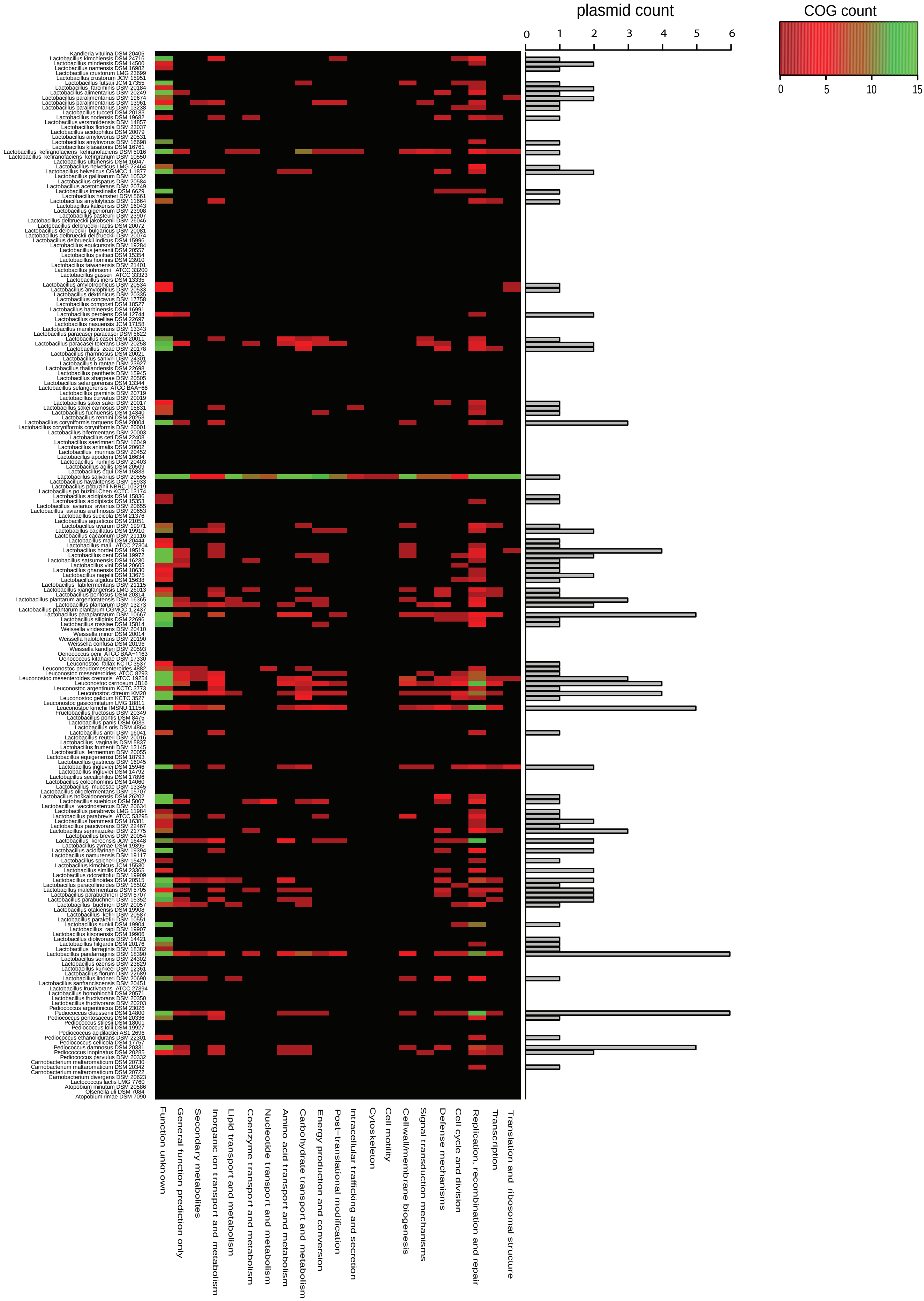

Supp. Figure 14. Heatmap and barplot showing the distribution of plasmid-associated COGs and the number of plasmids in the 213 genomes. The order of rows, from top to bottom, follows the order of genomes in the phylogenetic tree in Fig. 2 from top to bottom. The colour key shows a gradation in colour from black to red to green representing gene counts from 0 to 15.

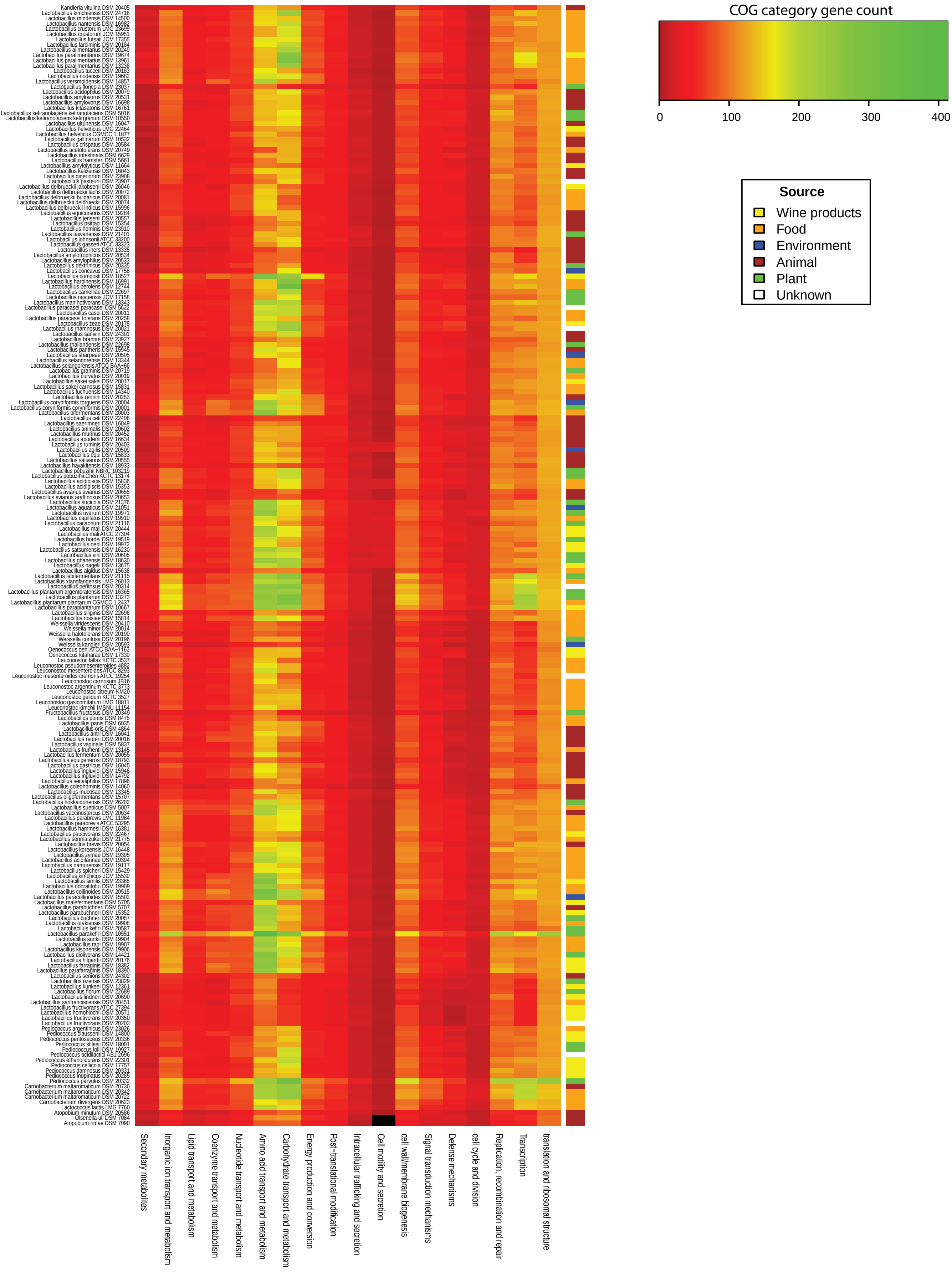

Supp. Figure 15. Heatmap of the distribution and abundance of 18 different COG categories across the 213 genomes. Number of genes assigned to each of the different COG categories is indicated by the color bar from black (absent) to green. Strains are ordered from top to bottom as they appear top-down in the phylogeny (Fig. 2) with source information indicated by the color bar along the top of the heatmap.

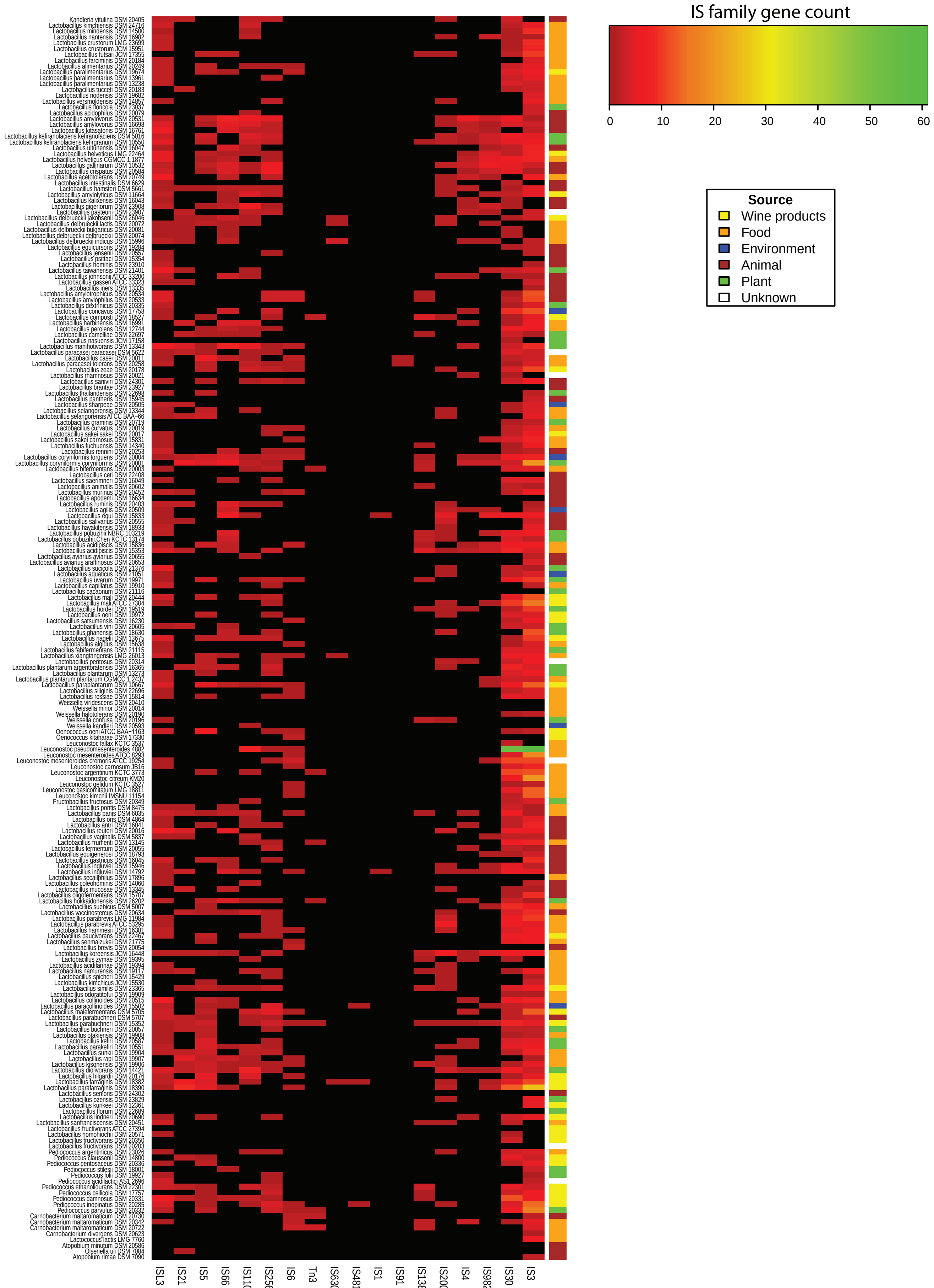

Supp. Figure 16. Heatmap of the distribution and abundance of 18 insertion sequence families across the 213 genomes. The number of genes assigned to each IS family is indicated by the color bar from black (absent) to green. The strains appear from top to bottom as they are featured top-down in the phylogeny (Fig. 2). Source information for each strain is indicated by a color bar along the top of the heatmap.

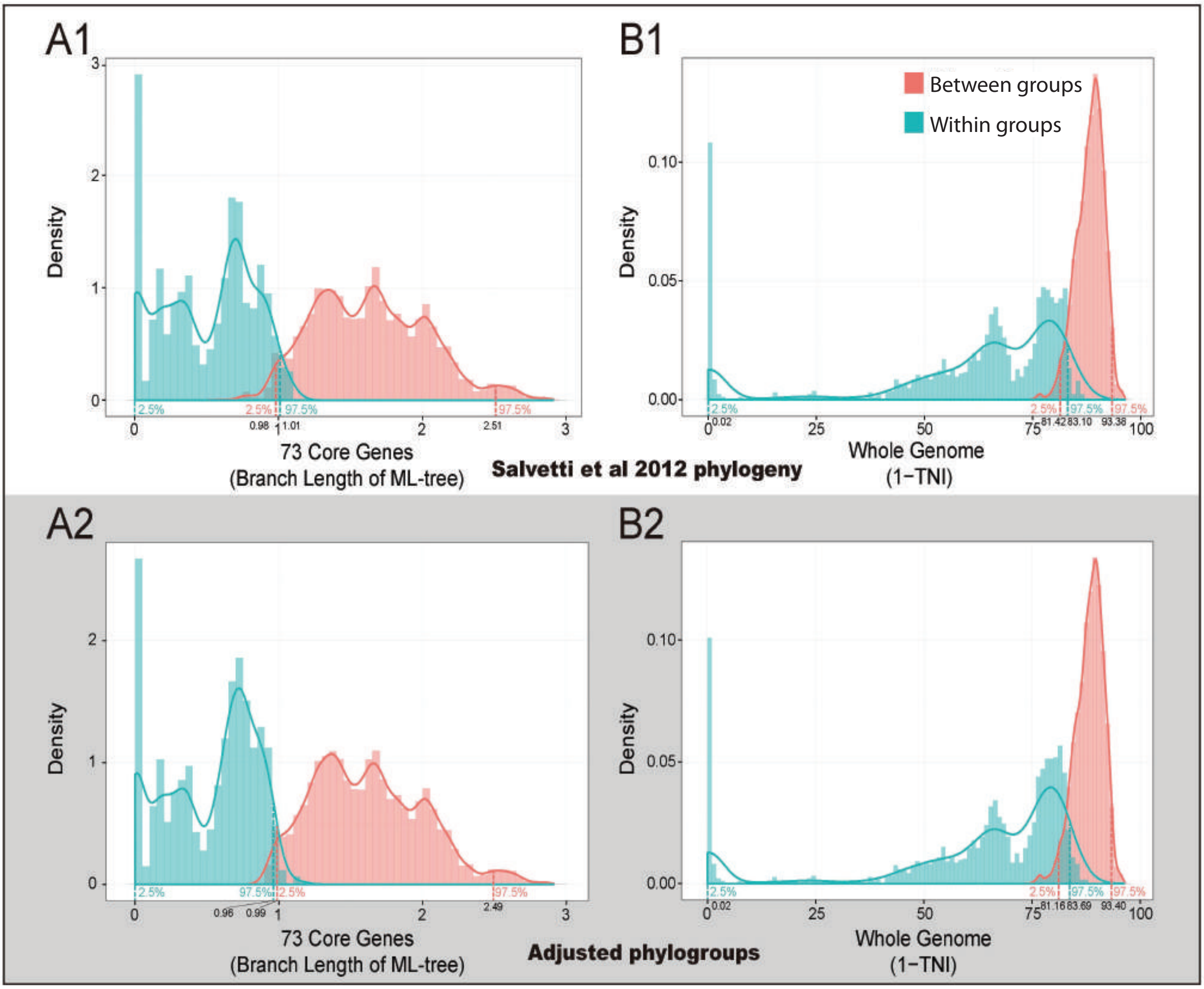

Supp. Figure 17. Branch length distribution and TNI value distribution (1-TNI). A current phylogrouping of the *Lactobacillus* Genus complex and a manually curated phylogrouping based on the maximum likelihood tree of 73 core genes (this study; see Supplementary Figure 18).

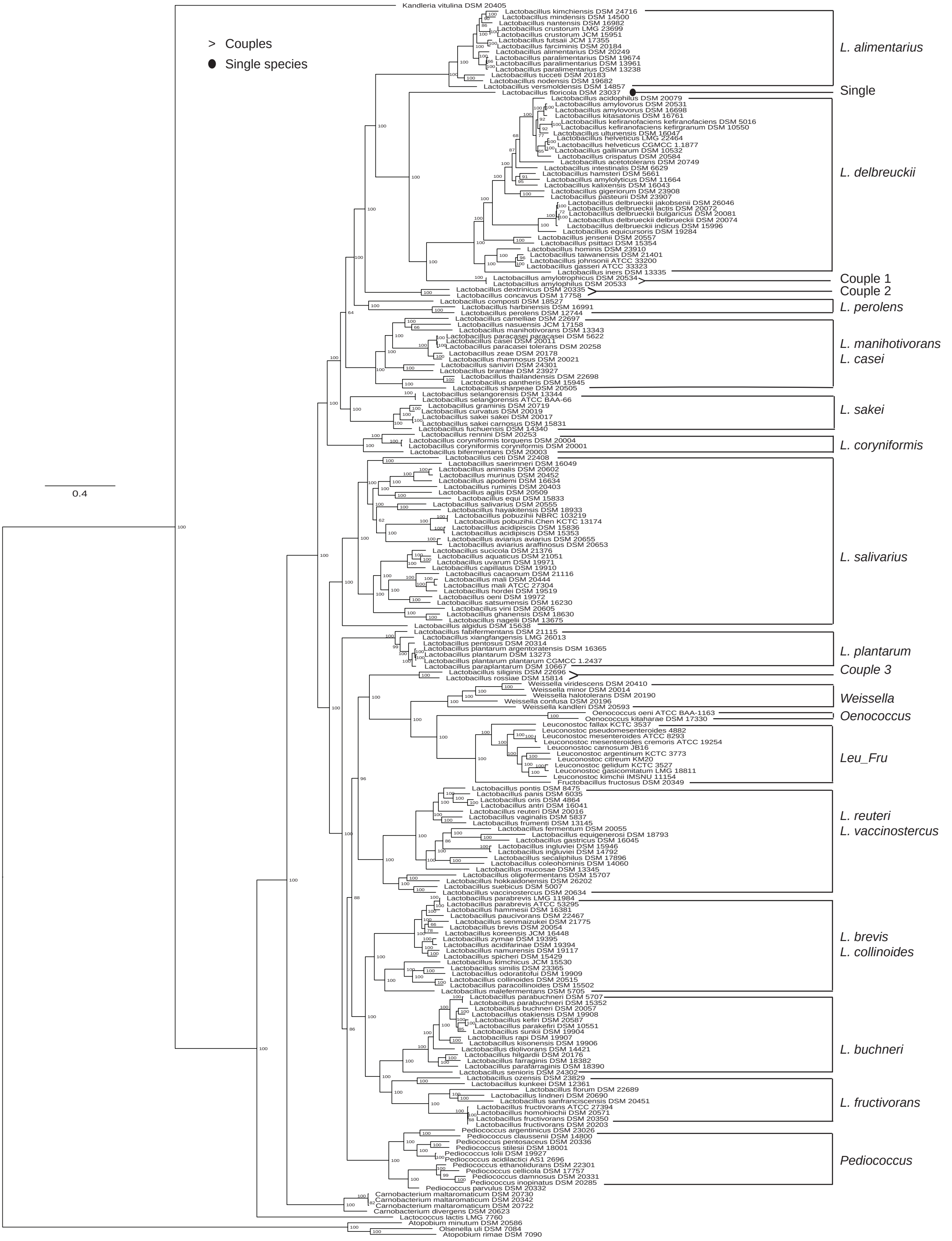

Supp. Figure 18. Manually curated phylogrouping of the *Lactobacillus* complex and associated genera based on 73 core genes maximum likelihood phylogeny. According to this revised phylogrouping, when the branch length between two strains is greater than 0.99, the probability is very high (>97.5%) that they belong to different phylogroups, and when the branch length is less than 0.96 between two strains, the probability that they belong to the same phylogroup is > 97.5%. Compared to the existing phylogrouping in Salvetti et al., 2012, the adjustments made here are:

1. Two species, *L. amylotrophicus* and *L. amylophilus* that originally belonged to the *L. delbrueckii* group were excluded from *L. delbrueckii* and defined as a new Couple.
2. The single species *L. composti* was combined with the phylogroup *L. perolens.*
3. The phylogroup *L. casei* and *L. manihotivorans* were combined together with the previously defined single species, *L. camelliae, L. saniviri, L. brantae, L. sharpeae*, and the Couple that contained *L. thailandensis* and *L. pantheris*, was defined as a single phylogroup.
4. The single species *L.algidus* was combined with the phylogroup *L. salivarius.*
5. *Leuconostoc* and *Fructobacillus* were defined as a single phylogroup.
6. The phylogroups *L. reuteri* and *L. vaccinostercus* were combined together.
7. The phylogroups *L. brevis* and *L. collinoides* and a single species, *L. malefermentans,* were combined as a single phylogroup.
8. *L. senioris* was combined with the phylogroup *L. buchneri.*
9. The couple that contained *L. ozensis* and *L. kunkeei* was combined into the phylogroup *L. fructivorans.*

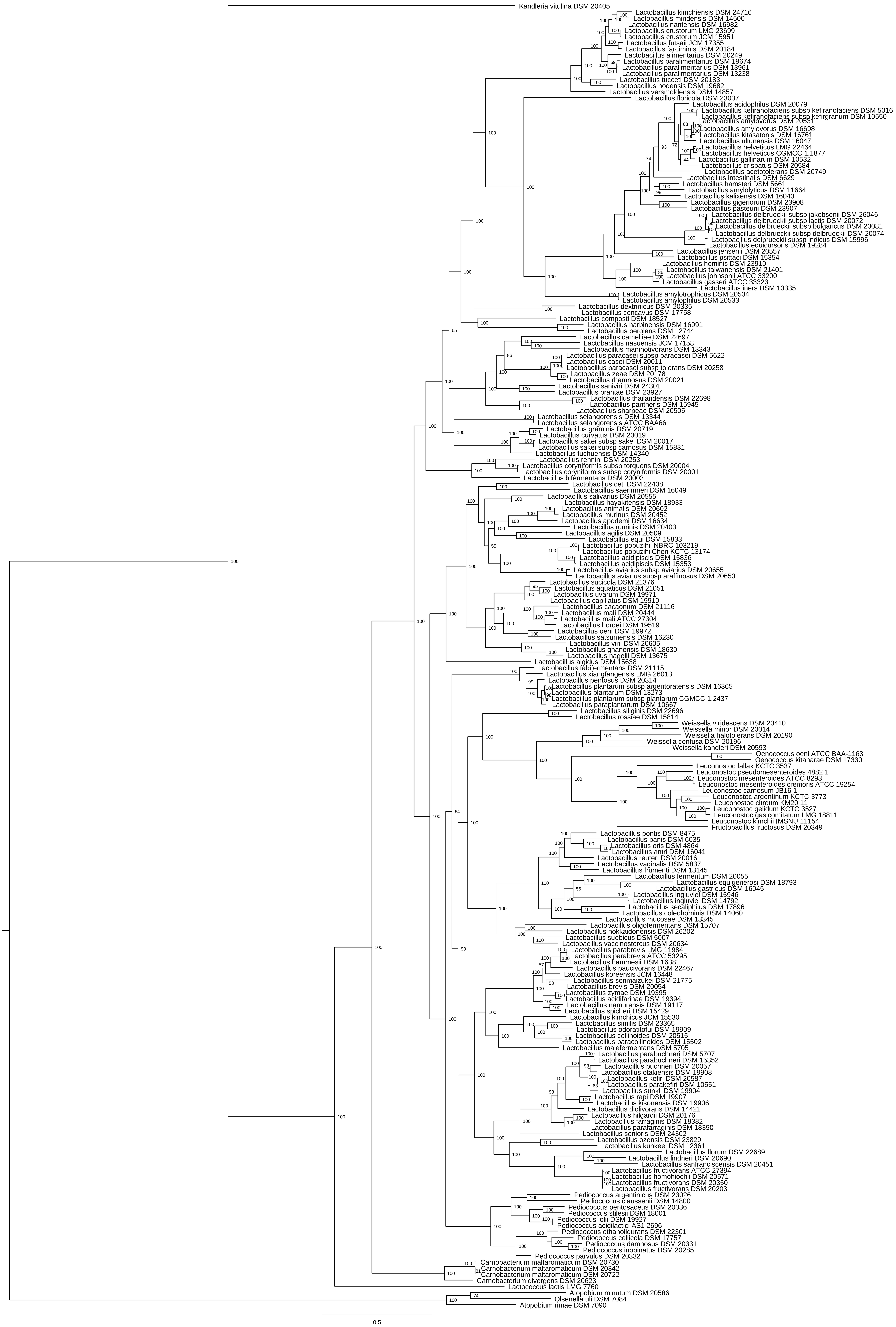

Supp. Figure 19. Phylogeny inferred from a 100 core gene dataset (27 partial + 73 complete core genes).

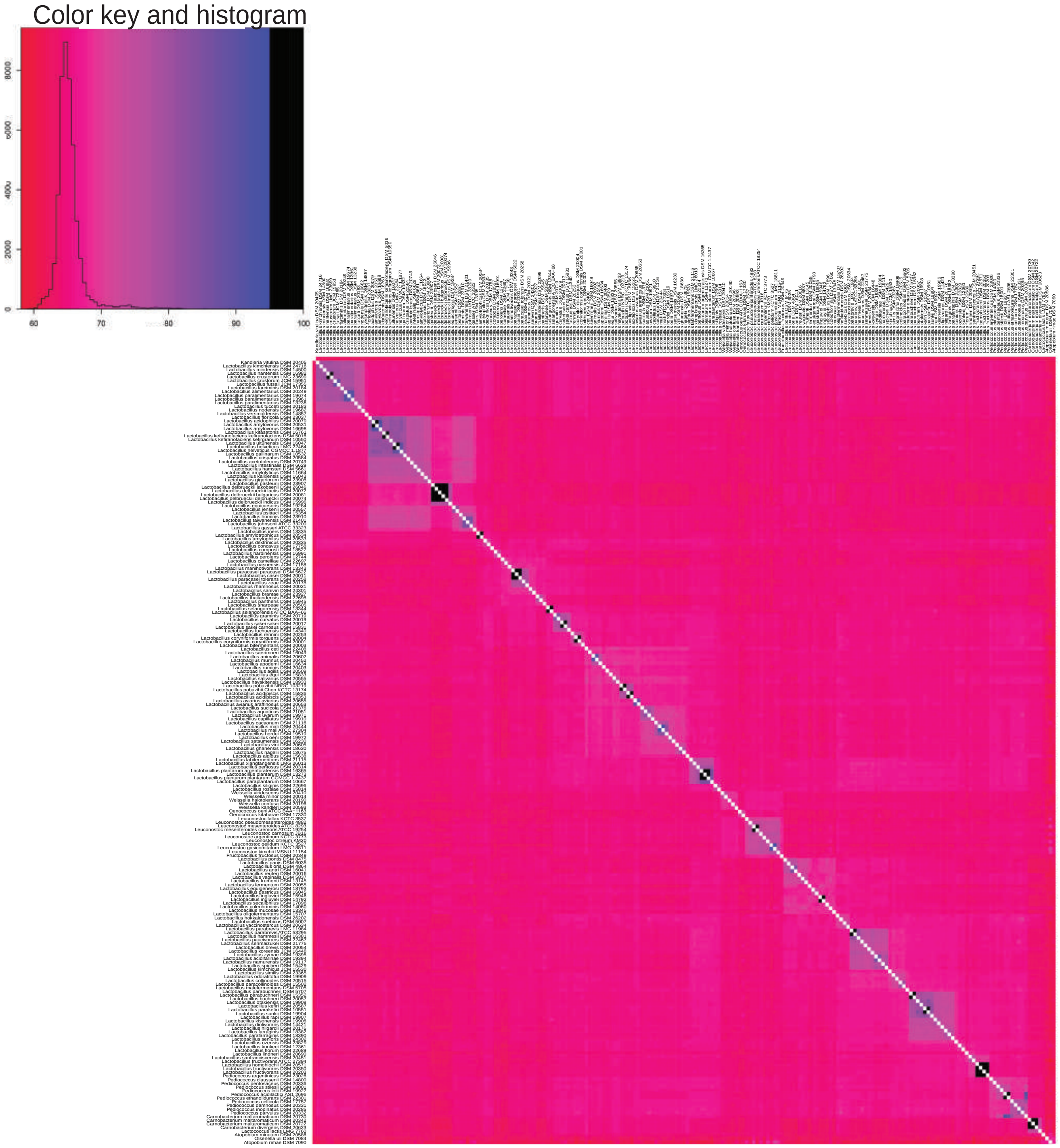

Supp. Figure 20. Heatmap of pairwise ANI values for 213 genomes. The order of the rows (top to bottom) and columns (left to right) is according to their position in the phylogenetic tree based on 73 core proteins (Fig. 2).

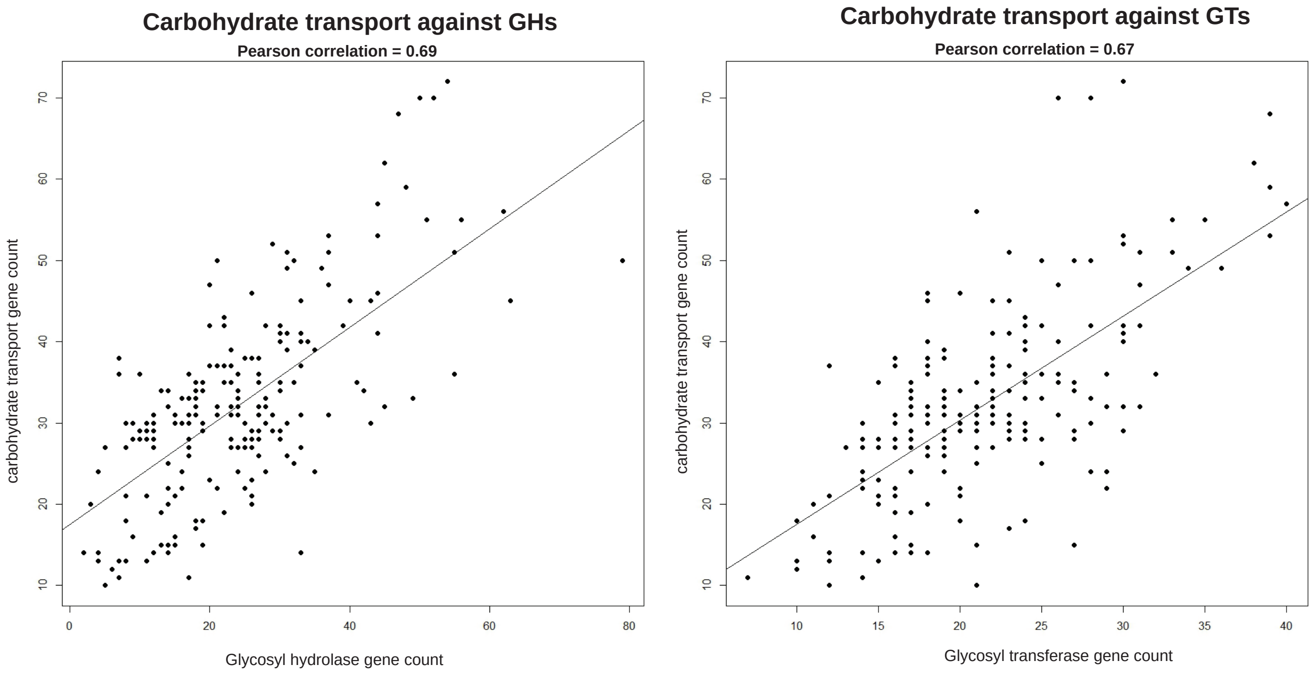

Supp. Figure 21. Scatterplots showing the correlation between the number of carbohydrate transport genes (y-axes) and the number of glycosyl hydrolase genes (x axis; left) and the number of glycosyl transferase genes (x-axis; right). The line of best fit for each plot was estimated using a least squares linear model.

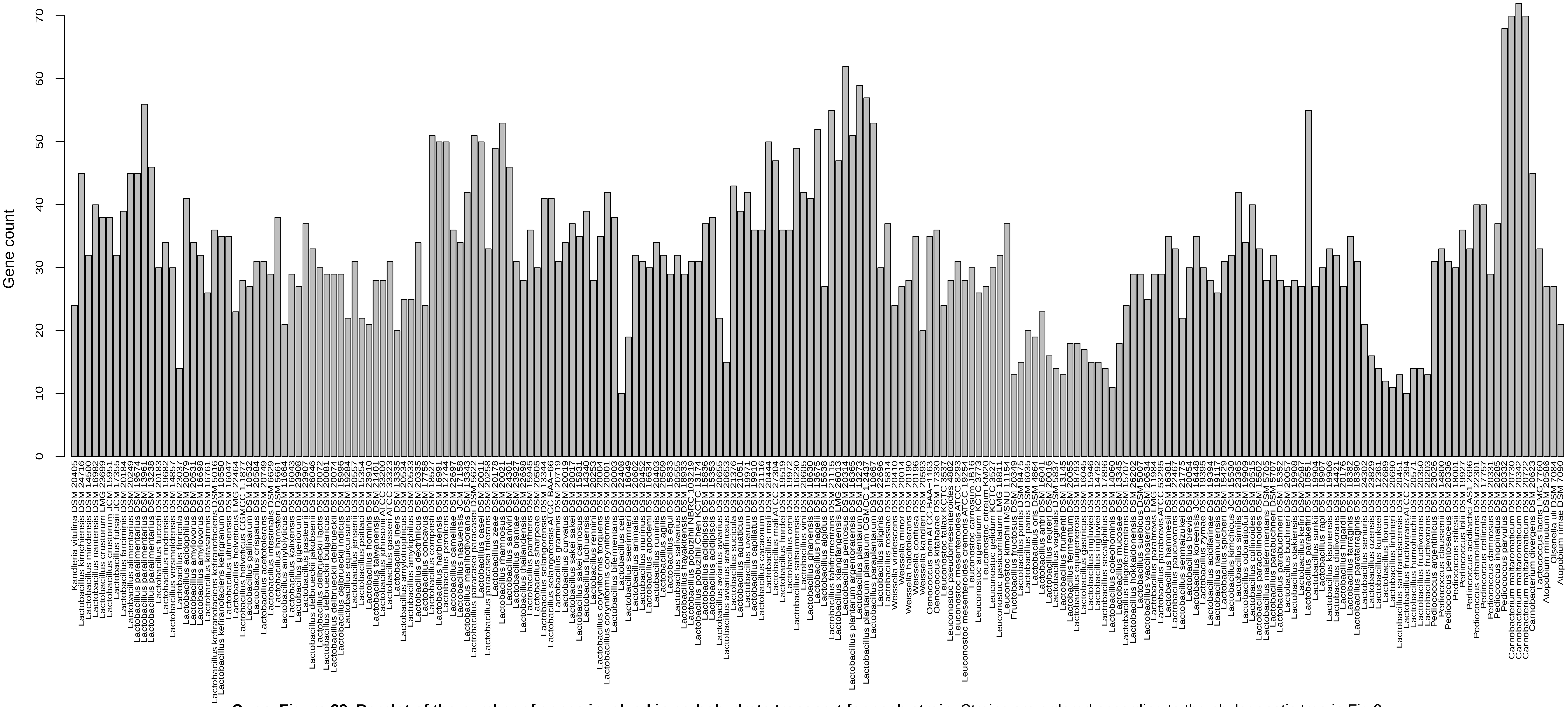

Supp. Figure 22. Barplot of the number of genes involved in carbohydrate transport for each strain. Strains are ordered according to the phylogenetic tree in Fig 2.

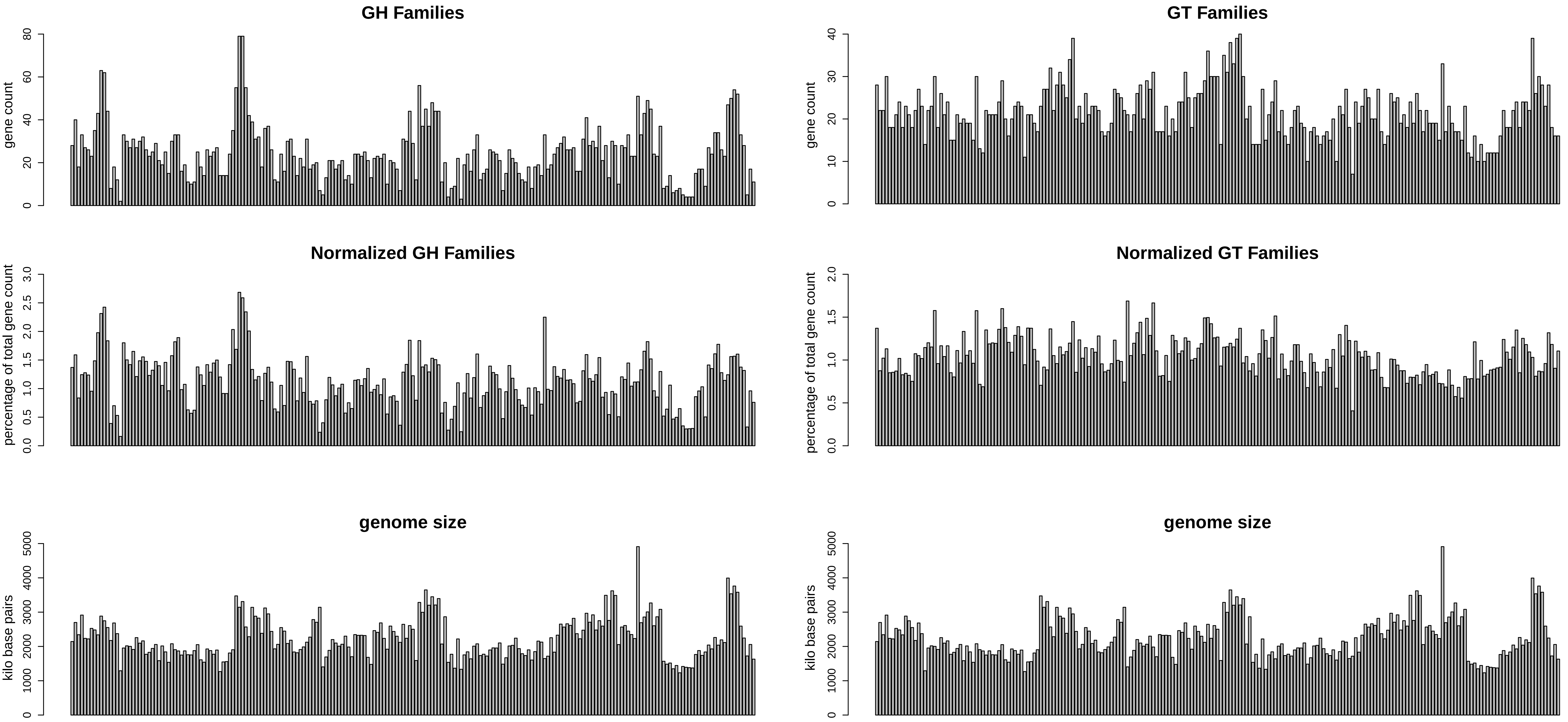

Supp. Figure 23. The effect of normalizing counts of GHs and GTs by genome size. The three bar-graphs on the left show, from top to bottom, GH gene counts, GH gene counts expressed as a percentage of the total gene count and genome size (in kbps). The equivalent for GTs is shown in the three bar-graphs on the right. Genome size is highly correlated with total number of genes per genome (Pearson; 0.99).

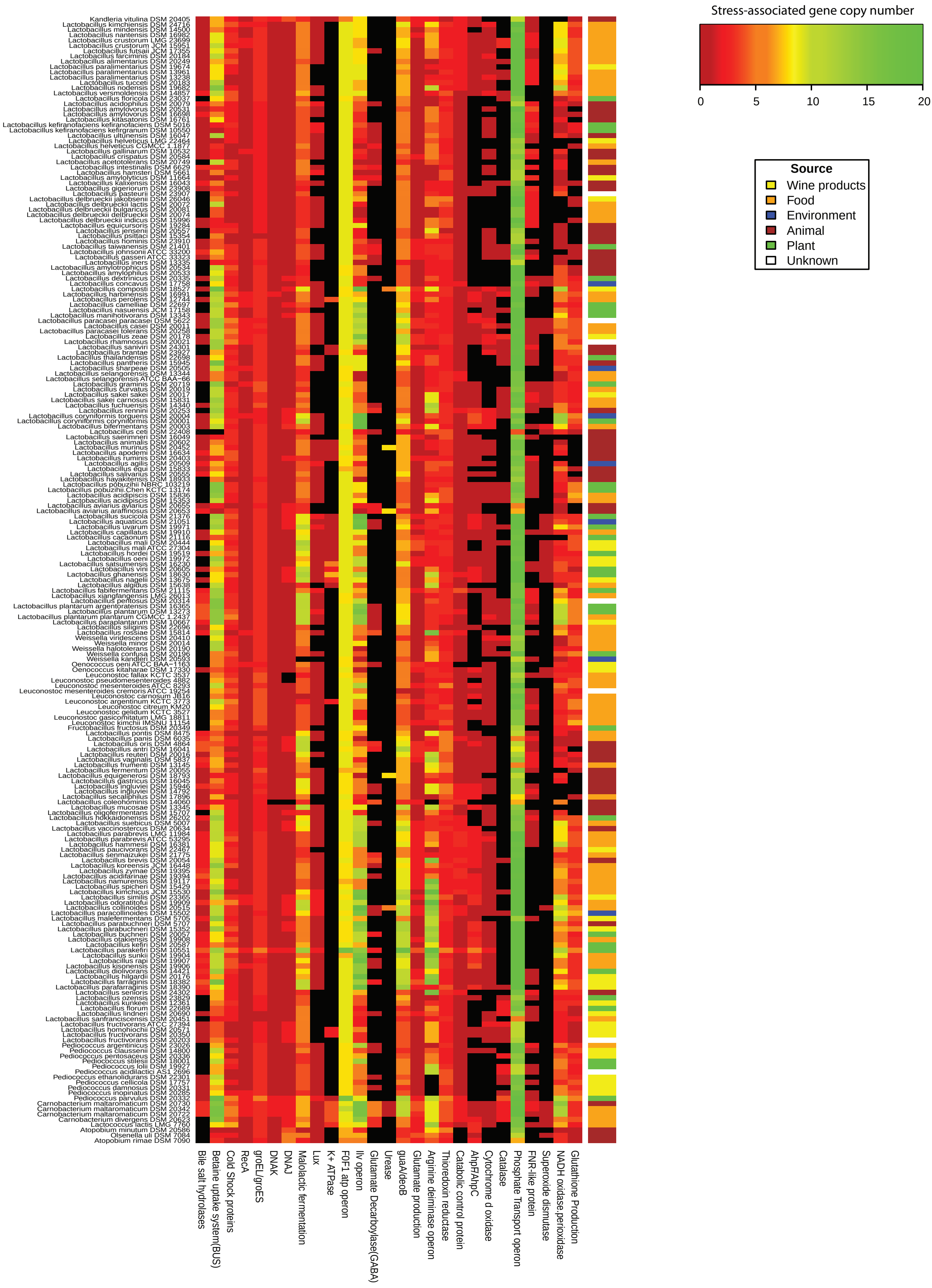

Supp. Figure 24. Heatmap of the distribution and abundance of stress response genes across the *Lactobacillus* Genus Complex and associated genera. Gene copy number for 27 stress-associated genes is indicated by the color key from black (absent) to green. Type of stress resistance is indicated by the names at the bottom of the figure. Strains are ordered from top to bottom as they appear top-down on the phylogeny (Fig. 2) with source information for each strain indicated by a color bar at the side of the heat-map.

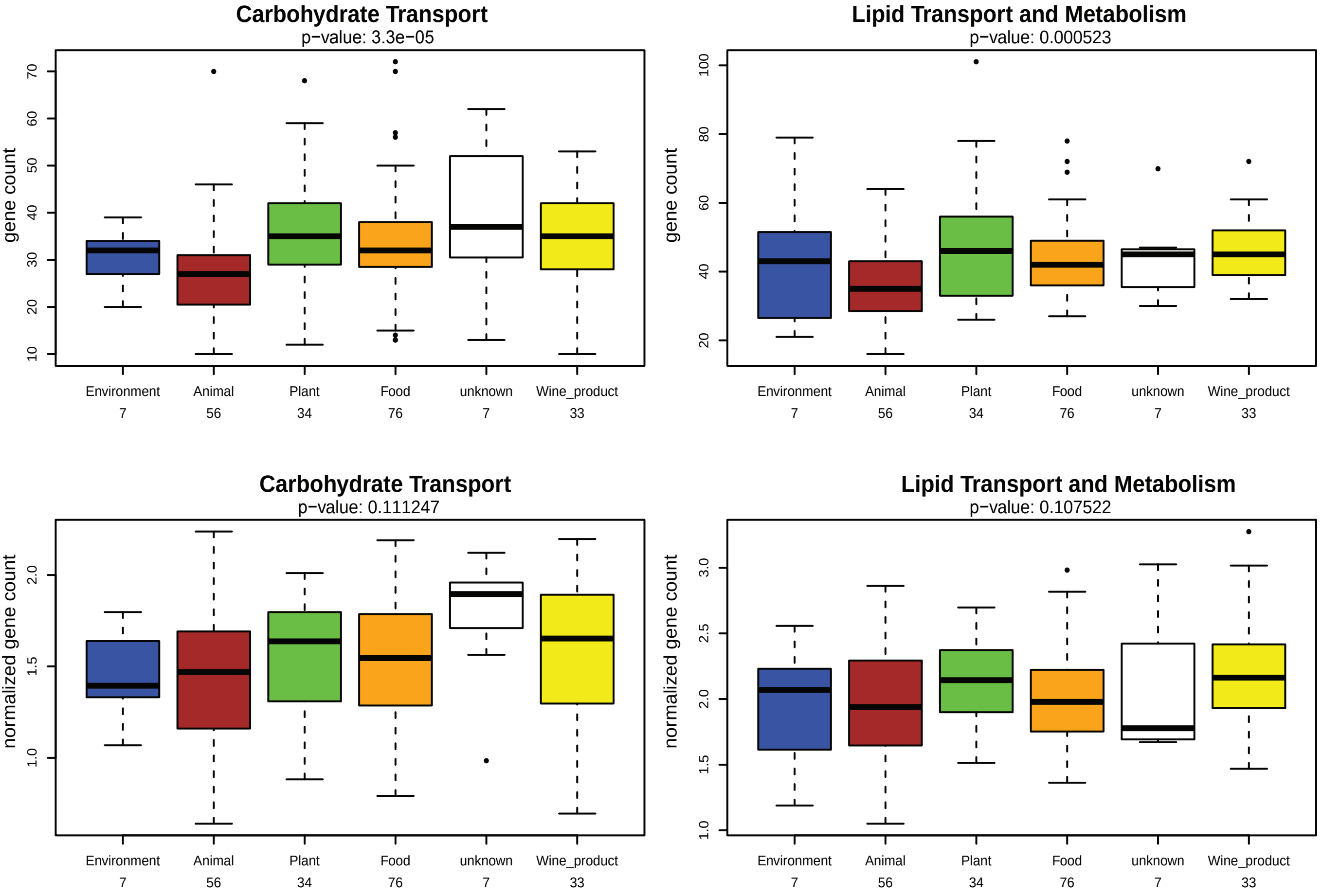

Supp. Figure 25. Association of carbohydrate transport and lipid transport/metabolism with niche. Top panels display raw gene counts and bottom panels display gene counts normalized by total genes. Boxplots represent a five-point summary of the data in the following order (from bottom to top): minimum, first quartile, median, third quartile and maximum. Outliers are represented as individual points above or below the boxplot.

Supplementary Tables

Supp. Table 1. Species analysed and their genomic features.

Supp. Table 2. Sequence information for the 73 core complete genes. The core genome was established based on genomes listed in Table 1. *Lactobacillus salivarius* genes are shown as exemplars for sequence retrieval. Columns are protein ID numbers, gene designation, locus tag, COG, annotation, co-ordinates in the *L. salivarius* UCC118 genome (NC_007929.1), strand and gene length.

Supp. Table 3. Genera used in building the tree of bacteria.

Supp. Table 4. Sixteen marker genes used to build the bacterial tree composed of 452 genera and 213 *Lactobacillus* genomes and *Lactobacillus* associated genera.

Supp. Table 5. Distribution of LPXTG-containing and sortase enzmyes across the 213 genomes.

Supp. Table 6. Distribution and abundance of cell envelope proteases (CEPs) and associated anchoring domains and motifs.

Supp. Table 7. CRISPR occurrence and diversity. CRISPR-Cas system type designation was determined by the presence of CRISPR repeats, spacers, the universal *cas1* gene, and the signature for each type, namely *cas3*, *cas9*, and *cas10*, for Type I, II, and III, respectively. A "Y" in the each of the gene columns designates that the gene was positioned next to the CRISPR locus. In instances where only a partial gene was annotated, the symbol "Y*" is shown. An N indicates that no *cas* gene was found. The type of the CRISPR-Cas system is noted in the "Type" column. When multiple CRISPR loci of the same type were present in the same genome, the type was then designated with a differential letter demarking their order in identification. When *cas* genes could not be annotated, the CRISPR-Cas system type was labeled "undefined." Strains that contained neither CRISPR repeats nor *cas* genes were labeled with the "N/A" designation. "DR length" corresponds to the number of nucleotides in the CRISPR direct repeat. The number of spacers was also determined for each repeat-spacer array.

Supp. Table 8. Sequence information for the 27 core partial genes.

Supp. Table 9. Presence and absence of the complete pathways for production of the 20 standard amino acids. A = auxotrophic; P = prototrophic.

Supp. Table 10. Presence of sirtuin homologs in the 213 genomes analyzed.

Supp. Table 11. Genomic regions related to bacteriocin production identified in the 213 genomes.

Table S1. Species analyzed and their genome features

Species Name	StrainID	Isolation year	Source	Genome Size (Mbp)	GC%	Predicted ORF number	Sequencing depth	Contig number	Short-read Archive Accession	Genome Accession No.	Niche category	Phylogroup*
Atopobium minutum	DSM-20586	1937	Perineal abscess	1.72	48.69	1595	116.11	64	ERX399734	JQBO00000000	Animal	Other
Atopobium rimae	DSM-7090	1991	Human gingival crevice	1.63	49.26	1524	122.70	9	ERX359753	JQCP00000000	Animal	Other
Carnobacterium divergens	DSM-20623	1984	vacuum packaged minced beef	2.59	34.95	2451	77.13	51	ERX359727	JQBS00000000	Food	Carnobacterium
Carnobacterium maltaromaticum	DSM-20342	1974	Milk with malty flavour	3.76	34.31	3569	53.17	155	ERX359700	JQBG00000000	Food	Carnobacterium
Carnobacterium maltaromaticum	DSM-20722	1974	Vacuum-packaged meat	3.58	34.31	3341	55.86	64	ERX359702	JQBU00000000	Food	Carnobacterium
Carnobacterium maltaromaticum	DSM-20730	1974	diseased rainbow trout	3.54	34.38	3369	56.57	56	ERX399735	JQBV00000000	Animal	Carnobacterium
Fructobacillus fructosus	DSM-20349	1956	Flowers	1.48	44.56	1514	134.69	45	ERX359711	JQBH00000000	Plant	Leu_Fru
Kandleria vitulina	DSM-20405	1973	Calf rumen	2.14	35.03	2126	93.31	138	ERX359782	JQBL00000000	Animal	Other
Lactobacillus acetotolerans	DSM-20749	1986	Fermented Vinegar Broth	1.59	36.26	1531	151.41	123	ERX359757	AYZC00000000	Food	L. delbrueckii
Lactobacillus acidifarinae	DSM-19394	2005	Artisanal wheat sourdough	2.92	51.59	2735	82.17	47	ERX359713	AZDV00000000	Food	L. brevis_L. collinoides
Lactobacillus acidipiscis	DSM-15353	2001	Cheese, Halloumi	2.32	39.06	2280	86.27	298	ERX399738	JQBK00000000	Food	L. salivarius
Lactobacillus acidipiscis	DSM-15836	2000	Fermented fish	2.33	39.07	2307	51.59	457	SRX456282	AZFI00000000	Food	L. salivarius
Lactobacillus acidophilus	DSM-20079	1900	Human	1.95	34.59	1886	61.41	30	SRX456246	AZCS00000000	Animal	L. delbrueckii
Lactobacillus agilis	DSM-20509	1982	Municipal sewage	2.06	41.74	2041	116.27	115	ERX359728	AYYP00000000	Environment	L. salivarius
Lactobacillus algidus	DSM-15638	2000	Vacuum-packed beef	1.59	36.03	1535	150.91	28	SRX456283	AZDI00000000	Food	L. salivarius
Lactobacillus alimentarius	DSM-20249	1970	Marinated fish product	2.34	35.4	2223	102.65	58	ERX359701	AZDQ00000000	Food	L. alimentarius
Lactobacillus amylolyticus	DSM-11664	1999	Acidified beer wort	1.54	38.24	1628	78.05	98	SRX456357	AZEP00000000	Wine product	L. delbrueckii
Lactobacillus amylophilus	DSM-20533	1981	Swine waste-corn fermentation	1.56	43.61	1582	153.98	44	ERX359717	AYYS00000000	Animal	Couple1
Lactobacillus amylotrophicus	DSM-20534	2006	Swine waste-corn fermentation	1.55	43.59	1589	154.98	21	SRX456279	AZCV00000000	Animal	Couple1
Lactobacillus amylovorus	DSM-16698	1981	Faeces, piglet intestine	2.00	37.82	2004	99.92	162	ERX359733	JQBQ00000000	Animal	L. delbrueckii
Lactobacillus amylovorus	DSM-20531	1981	Cattle waste-corn fermentation	2.02	37.77	2061	59.48	116	SRX456280	AZCM00000000	Animal	L. delbrueckii
Lactobacillus animalis	DSM-20602	1983	Dental plaque of baboon	1.89	41.06	1813	127.29	93	ERX359783	AYYW00000000	Animal	L. salivarius
Lactobacillus antri	DSM-16041	2005	Gastric biopsies, Human stomach mucosa	2.24	51.11	2124	53.53	92	SRX456248	AZDK00000000	Animal	L. reuteri_L. vaccinostercus
Lactobacillus apodemi	DSM-16634	2006	Faeces of wild Japanese wood mouse	2.10	38.63	2009	114.35	109	ERX399737	AZFT00000000	Animal	L. salivarius
Lactobacillus aquaticus	DSM-21051	2009	Surface of a eutrophic freshwater pond	2.41	37.41	2279	99.53	61	ERX359723	AYZD00000000	Environment	L. salivarius
Lactobacillus aviarius subsp. araffinosus	DSM-20653	1986	Intestine of chicken	1.48	38.13	1429	162.40	49	ERX405609	AYYZ00000000	Animal	L. salivarius
Lactobacillus aviarius subsp. aviarius	DSM-20655	1985	Faeces of chicken	1.68	40.12	1591	142.61	41	ERX359760	AYZA00000000	Animal	L. salivarius
Lactobacillus bifermentans	DSM-20003	1943	Blown cheese	3.14	44.29	3088	76.38	205	ERX359761	AZDA00000000	Food	L. coryniformis
Lactobacillus brantae	DSM-23927	2012	Faeces of Canada goose	1.93	47.48	1910	124.36	12	SRX456251	AYZQ00000000	Animal	L. manihotivorans_L. casei
Lactobacillus brevis	DSM-20054	1919	Faeces	2.47	45.96	2425	48.50	11	SRX456278	AZCP00000000	Animal	L. brevis_L. collinoides
Lactobacillus buchneri	DSM-20057	1903	Tomato pulp	2.45	44.41	2349	49.05	90	SRX456296	AZDM00000000	Plant	L. buchneri
Lactobacillus cacaonum	DSM-21116	2009	Cocoa bean heap fermentation	1.92	33.87	1833	124.98	25	ERX399739	AYZE00000000	Plant	L. salivarius
Lactobacillus camelliae	DSM-22697	2007	Fermented tea leaves (miang)	2.57	55.39	2430	93.46	144	ERX359699	AYZJ00000000	Plant	L. manihotivorans_L. casei
Lactobacillus capillatus	DSM-19910	2008	Fermented brine used for stinky tofu productio	2.24	37.63	2113	107.36	79	ERX359689	AZEF00000000	Food	L. salivarius
Lactobacillus casei	DSM-20011	1916	Cheese	2.83	46.45	2817	42.46	140	SRX456230	AZCO00000000	Food	L. manihotivorans_L. casei
Lactobacillus ceti	DSM-22408	2008	Lungs of a beaked whale	1.40	33.73	1258	142.46	69	ERX399740	JQBZ00000000	Animal	L. salivarius
Lactobacillus coleohominis	DSM-14060	2001	Human vagina	1.72	40.81	1882	15.69	964	SRX456252	AZEW00000000	Animal	L. reuteri_L. vaccinostercus
Lactobacillus collinoides	DSM-20515	1972	Fermenting apple juice	3.62	46.11	3380	66.27	165	ERX399741	AYYR00000000	Food	L. brevis_L. collinoides
Lactobacillus composti	DSM-18527	2007	Composting material of distilled shochu residue	3.47	43.95	3370	48.57	131	ERX359694	AZGA00000000	Wine product	L. perolens
Lactobacillus concavus	DSM-17758	2005	Walls of a distilled spirit fermenting cellar	1.90	43.3	1852	85.63	141	ERX399742	AZFX00000000	Environment	Couple2
Lactobacillus coryniformis subsp. coryniformis	DSM-20001	1965	Silage	2.71	42.86	2625	44.36	198	SRX456340	AZCN00000000	Plant	L. coryniformis
Lactobacillus coryniformis subsp. torquens	DSM-20004	1965	Air of cow shed	2.78	42.94	2699	43.11	410	SRX456233	AZDC00000000	Environment	L. coryniformis
Lactobacillus crispatus	DSM-20584	1953	Eye	2.06	36.59	2038	58.34	150	SRX456245	AZCW00000000	Animal	L. delbrueckii
Lactobacillus crustorum	JCM-15951	2007	Wheat sourdough	2.22	35	2150	54.07	101	SRX456344	AZDB00000000	Food	L. alimentarius
Lactobacillus crustorum	LMG-23699	2007	Wheat sourdough	2.24	34.99	2206	89.46	87	ERX359721	JQCK00000000	Food	L. alimentarius
Lactobacillus curvatus	DSM-20019	1903	Milk	1.82	41.97	1819	66.09	250	SRX456225	AZDL00000000	Food	L. sakei
Lactobacillus delbrueckii subsp. bulgaricus	DSM-20081	1919	Bulgarian yoghourt	1.76	49.91	1907	113.73	80	ERX359756	JQAV00000000	Food	L. delbrueckii
Lactobacillus delbrueckii subsp. delbrueckii	DSM-20074	1896	Sour grain mash	1.75	49.86	1884	136.93	69	SRX456341	AZCR00000000	Food	L. delbrueckii
Lactobacillus delbrueckii subsp. indicus	DSM-15996	2005	Traditional dairy fermented product (Dahi type)	1.88	49.54	1871	127.80	239	ERX399745	AZFL00000000	Food	L. delbrueckii
Lactobacillus delbrueckii subsp. jakobsenii	DSM-26046	2013	Dolo wort (Alcholic fermented beverage)	1.75	50.31	1715	102.89	135	SRX690302	JQCG00000000	Wine product	L. delbrueckii
Lactobacillus delbrueckii subsp. lactis	DSM-20072	1919	Emmental cheese	1.87	49.86	1842	64.16	223	SRX456227	AZDE00000000	Food	L. delbrueckii
Lactobacillus dextrinicus	DSM-20335	1964	Silage	1.81	38.05	1743	93.72	36	ERX399746	AYYK00000000	Plant	Couple2
Lactobacillus diolivorans	DSM-14421	2002	Maize silage	3.27	40.01	3119	73.44	154	ERX359710	AZEY00000000	Plant	L. buchneri
Lactobacillus equi	DSM-15833	2002	Faeces of horses	2.30	39.03	2193	104.34	183	ERX359739	AZFH00000000	Animal	L. salivarius
Lactobacillus equicursoris	DSM-19284	2010	Healthy thoroughbred racehorse	2.05	47.71	1887	58.46	316	SRX456237	AZDU00000000	Animal	L. delbrueckii
Lactobacillus equigenerosi	DSM-18793	2008	Faeces of thoroughbred horse	1.60	42.65	1534	149.66	93	ERX359704	AZGC00000000	Animal	L. reuteri_L. vaccinostercus
Lactobacillus fabifermentans	DSM-21115	2009	Cocoa bean heap fermentation	3.28	45.03	3222	73.09	214	ERX359706	AYGX00000000	Plant	L. plantarum
Lactobacillus farciminis	DSM-20184	1970	Sausage	2.48	36.38	2403	48.37	76	SRX456285	AZDR00000000	Food	L. alimentarius
Lactobacillus farraginis	DSM-18382	2007	Composting material of distilled shochu residue	2.86	42.05	2863	83.78	193	ERX359697	AZFY00000000	Wine product	L. buchneri
Lactobacillus fermentum	DSM-20055	1901	Human saliva	1.90	52.42	1856	105.26	102	ERX359692	JQAU00000000	Animal	L. reuteri_L. vaccinostercus
Lactobacillus floricola	DSM-23037	2011	Flower of Caltha palustris	1.29	34.53	1240	185.93	30	ERX359719	AYZL00000000	Plant	Single
Lactobacillus florum	DSM-22689	2010	Peony (Paeonia suffruticosa)	1.35	41.14	1317	88.98	50	SRX456305	AYZI00000000	Plant	L. fructivorans
Lactobacillus fructivorans	ATCC-27394	1934	Wine	1.42	38.82	1490	141.19	12	ERX399748	JQAS00000000	Wine product	L. fructivorans
Lactobacillus fructivorans	DSM-20203	1934	N/A	1.37	38.88	1348	87.44	16	SRX456290	AZDS00000000	Unknown	L. fructivorans
Lactobacillus fructivorans	DSM-20350	1934	Spoiled sake	1.38	38.91	1380	144.85	47	ERX399747	JQBI00000000	Wine product	L. fructivorans
Lactobacillus frumenti	DSM-13145	2000	Rye-bran sourdough	1.73	42.55	1681	138.35	34	ERX359759	AZER00000000	Food	L. reuteri_L. vaccinostercus
Lactobacillus fuchuensis	DSM-14340	2002	Vacuum-packaged beef	2.12	41.78	2029	113.00	89	ERX359709	AZEX00000000	Food	L. sakei
Lactobacillus futsaii	JCM-17355	2010	Fu-tsai, a traditional fermented mustard product	2.53	35.6	2482	95.00	208	ERX359725	AZDO00000000	Food	L. alimentarius
Lactobacillus gallinarum	DSM-10532	1992	Chicken crop	1.94	36.48	1956	123.63	113	ERX359738	AZEL00000000	Animal	L. delbrueckii
Lactobacillus gasseri	ATCC-33323	1980	Human	1.89	35.26	1863	NA	1	NA	NC_008530	Animal	L. delbrueckii
Lactobacillus gastricus	DSM-16045	2005	Gastric biopsies, Human stomach mucosa	1.85	41.64	1810	64.92	66	SRX456277	AZFN00000000	Animal	L. reuteri_L. vaccinostercus
Lactobacillus ghanensis	DSM-18630	2007	Cocoa fermentation	2.61	37.09	2485	92.03	49	ERX359732	AZGB00000000	Plant	L. salivarius
Lactobacillus gigeriorum	DSM-23908	2012	Chicken crop	1.91	36.9	1867	62.96	116	SRX456255	AYZO00000000	Animal	L. delbrueckii
Lactobacillus graminis	DSM-20719	1989	Grass silage	1.84	40.26	1766	130.53	100	ERX359758	AYZB00000000	Plant	L. sakei
Lactobacillus hammesii	DSM-16381	2005	Wheat sourdough	2.82	49.38	2582	85.11	94	ERX359712	AZFS00000000	Food	L. brevis_L. collinoides
Lactobacillus hamsteri	DSM-5661	1988	Faeces of hamster	1.84	35.08	1744	130.37	112	ERX359737	AZGI00000000	Animal	L. delbrueckii
Lactobacillus harbinensis	DSM-16991	2006	Chinese traditional fermented vegetable Suan ca	3.14	53.08	3067	76.35	210	ERX399749	AZFW00000000	Food	L. perolens
Lactobacillus hayakitensis	DSM-18933	2007	Faeces of thoroughbred horse	1.70	34.08	1597	141.11	216	ERX359691	AZGD00000000	Animal	L. salivarius
Lactobacillus helveticus	CGMCC-1.1877	1919	Emmental cheese	1.83	36.78	1969	65.62	257	SRX456228	AZEK00000000	Food	L. delbrueckii
Lactobacillus helveticus	LMG-22464	2005	Malt whiskey fermentation	1.77	36.52	1869	112.88	267	ERX359764	JQCJ00000000	Wine product	L. delbrueckii
Lactobacillus hilgardii	DSM-20176	1936	Wine	2.60	39.58	2635	46.10	125	SRX456360	AZDF00000000	Wine product	L. buchneri
Lactobacillus hokkaidonensis	DSM-26202	2013	timothy grass (Phleum pratense L.) silage	2.33	38.13	2285	103.03	84	SRX690303	JQCH00000000	Plant	#L. reuteri_L. vaccinostercus
Lactobacillus hominis	DSM-23910	2013	Human intestine	1.93	35.15	1873	62.29	47	SRX456258	AYZP00000000	Animal	L. delbrueckii
Lactobacillus homohiochii	DSM-20571	1957	Spoiled sake	1.39	38.85	1392	172.28	33	ERX405610	JQBN00000000	Wine product	L. fructivorans
Lactobacillus hordei	DSM-19519	2008	Malted barley	2.30	34.77	2243	104.44	137	ERX359755	AZDX00000000	Plant	L. salivarius
Lactobacillus iners	DSM-13335	1999	Human urine	1.27	32.52	1186	94.66	58	SRX456259	AZET00000000	Animal	L. delbrueckii
Lactobacillus ingluviei	DSM-14792	2003	Chicken faeces	2.13	49.99	2068	94.05	111	ERX359778	JQBA00000000	Animal	L. reuteri_L. vaccinostercus
Lactobacillus ingluviei	DSM-15946	2003	Pigeon, crop	2.16	49.88	2101	111.34	113	ERX359729	AZFK00000000	Animal	L. reuteri_L. vaccinostercus
Lactobacillus intestinalis	DSM-6629	1974	Intestine of rat	2.01	35.37	1858	119.13	87	ERX359740	AZGN00000000	Animal	L. delbrueckii
Lactobacillus jensenii	DSM-20557	1970	Human vaginal discharge	1.61	34.33	1473	74.51	99	SRX456262	AYYU00000000	Animal	L. delbrueckii
Lactobacillus johnsonii	ATCC-33200	1992	Human blood	1.77	34.43	1779	67.66	37	SRX456239	AZCY00000000	Animal	L. delbrueckii
Lactobacillus kalixensis	DSM-16043	2005	Gastric biopsies, Human stomach mucosa	2.08	36.1	1948	115.49	108	ERX359741	AZFM00000000	Animal	L. delbrueckii
Lactobacillus kefiranofaciens subsp. kefiranofa	DSM-5016	1988	Kefir grains	2.26	37.22	2320	53.16	133	SRX456311	AZGG00000000	Plant	L. delbrueckii
Lactobacillus kefiranofaciens subsp. kefirgranu	DSM-10550	1994	Kefir grains	2.10	37.48	2111	114.49	213	ERX359714	AZEM00000000	Plant	L. delbrueckii
Lactobacillus kefiri	DSM-20587	1983	Kefir grains	2.23	41.66	2128	107.57	109	ERX359693	AYYV00000000	Plant	L. buchneri
Lactobacillus kimchicus	JCM-15530	2008	Kimchi	2.59	46.59	2568	92.55	45	SRX456314	AZCX00000000	Food	L. brevis_L. collinoides
Lactobacillus kimchiensis	DSM-24716	2013	Kimchi	2.70	35.48	2577	88.93	71	SRX690301	JQCF00000000	Food	L. alimentarius
Lactobacillus kisonensis	DSM-19906	2009	Sunki, a Japanese traditional pickle	3.01	41.74	2825	39.92	122	SRX456316	AZEB00000000	Food	L. buchneri
Lactobacillus kitasatonis	DSM-16761	2003	Chicken intestine	1.91	37.51	1927	125.47	55	ERX359742	AZFU00000000	Animal	L. delbrueckii
Lactobacillus koreensis	JCM-16448	2011	Cabbage Kimchi	2.97	49.15	2687	80.88	195	ERX359724	AZDP00000000	Food	L. brevis_L. collinoides
Lactobacillus kunkeei	DSM-12361	1998	Commerical grape wine	1.52	36.43	1343	158.28	41	ERX399750	AZCK00000000	Wine product	L. fructivorans
Lactobacillus lindneri	DSM-20690	1901	Spoit beer	1.44	34.14	1437	166.15	49	ERX359707	JQBT00000000	Wine product	L. fructivorans
Lactobacillus malefermentans	DSM-5705	1953	Sour beer	2.05	41.03	2037	58.40	152	SRX456362	AZGJ00000000	Wine product	L. brevis_L. collinoides
Lactobacillus mali	ATCC-27304	1970	Wine must	2.44	36.4	2378	82.07	78	ERX359763	JQAR00000000	Wine product	L. salivarius
Lactobacillus mali	DSM-20444	1970	Apple juice from cider press	2.59	36.06	2538	46.26	211	SRX456318	AYYH00000000	Wine product	L. salivarius
Lactobacillus manihotivorans	DSM-13343	1998	Cassava sour starch fermentation	3.14	47.7	3055	76.42	436	ERX359690	AZEU00000000	Plant	L. manihotivorans_L. casei
Lactobacillus mindensis	DSM-14500	2003	Sourdough	2.34	38.21	2222	102.48	143	ERX359754	AZEZ00000000	Food	L. alimentarius
Lactobacillus mucosae	DSM-13345	2000	Pig small intestine	2.25	46.4	2094	53.23	91	SRX456264	AZEQ00000000	Animal	L. reuteri_L. vaccinostercus
Lactobacillus murinus	DSM-20452	1982	Intestine of rat	2.20	40.05	2042	83.42	294	ERX359734	AYYN00000000	Animal	L. salivarius
Lactobacillus nagelii	DSM-13675	2000	Partially fermented wine	2.50	36.69	2449	95.93	66	ERX359735	AZEV00000000	Wine product	L. salivarius
Lactobacillus namurensis	DSM-19117	2007	Sourdough	2.48	51.99	2248	96.80	88	ERX399751	AZDT00000000	Food	L. brevis_L. collinoides
Lactobacillus nantensis	DSM-16982	2006	Wheat sourdough	2.91	36.18	2716	82.35	95	SRX456349	AZFV00000000	Food	L. alimentarius
Lactobacillus nasuensis	JCM-17158	2012	Sudangrass silage sample	2.28	57.02	2151	105.08	60	ERX359747	AZDJ00000000	Plant	L. manihotivorans_L. casei
Lactobacillus nodensis	DSM-19682	2009	Japanese pickles	2.68	37.57	2639	89.42	28	ERX359744	AZDZ00000000	Food	L. alimentarius
Lactobacillus odoratitofui	DSM-19909	2010	Fermented brine used for stinky tofu productior	2.76	44.25	2474	86.99	83	ERX399753	AZEE00000000	Food	L. brevis_L. collinoides
Lactobacillus oeni	DSM-19972	2009	Bobal wine	2.12	37.33	2045	113.19	60	ERX359745	AZEH00000000	Wine product	L. salivarius
Lactobacillus oligofermentans	DSM-15707	2005	Broiler leg	1.83	35.57	1771	130.87	43	ERX359748	AZFE00000000	Animal	L. reuteri_L. vaccinostercus
Lactobacillus oris	DSM-4864	1988	Human saliva	2.03	49.98	1916	59.06	75	SRX456266	AZGE00000000	Animal	L. reuteri_L. vaccinostercus
Lactobacillus otakiensis	DSM-19908	2009	Sunki, a Japanese traditional pickle	2.35	42.39	2280	102.25	28	ERX359743	AZED00000000	Food	L. buchneri
Lactobacillus ozensis	DSM-23829	2011	Chrysanthemum, Oze National Park	1.48	31.93	1426	161.73	64	ERX399754	AYYQ00000000	Plant	L. fructivorans
Lactobacillus panis	DSM-6035	1996	Sourdough	2.01	48.05	1915	119.18	198	ERX399755	AZGM00000000	Food	L. reuteri_L. vaccinostercus
Lactobacillus pantheris	DSM-15945	2002	Jaguar faeces	2.55	52.9	2420	94.10	124	ERX359736	AZFJ00000000	Animal	L. manihotivorans_L. casei
Lactobacillus parabrevis	ATCC-53295	2006	Cheese	2.61	49.05	2332	45.90	133	SRX456325	AZCZ00000000	Food	L. brevis_L. collinoides
Lactobacillus parabrevis	LMG-11984	2006	Wheat	2.66	48.98	2398	75.19	123	ERX399756	JQCI00000000	Food	L. brevis_L. collinoides
Lactobacillus parabuchneri	DSM-15352	1989	Blair athol distillery	2.61	43.47	2423	76.63	274	ERX359703	JQBJ00000000	Wine product	L. buchneri
Lactobacillus parabuchneri	DSM-5707	1989	Human saliva	2.57	43.43	2407	93.53	50	SRX456268	AZGK00000000	Animal	L. buchneri
Lactobacillus paracasei subsp. paracasei	DSM-5622	1989	N/A	2.88	46.5	2879	41.64	170	SRX456291	AZGH00000000	Unknown	L. manihotivorans_L. casei
Lactobacillus paracasei subsp. tolerans	DSM-20258	1965	Pasteurized milk	2.38	46.42	2433	50.35	389	SRX456229	AYYJ00000000	Food	L. manihotivorans_L. casei
Lactobacillus paracollinoides	DSM-15502	2004	Brewery environment	3.49	46.84	3247	68.82	275	ERX399758	AZFD00000000	Environment	L. brevis_L. collinoides
Lactobacillus parafarraginis	DSM-18390	2007	Composting material of distilled shochun residu	3.08	45.23	3007	38.94	254	SRX456365	AZFZ00000000	Wine product	L. buchneri
Lactobacillus parakefiri	DSM-10551	1994	Kefir grain	4.91	42.59	4758	40.73	672	ERX399759	AZEN00000000	Plant	L. buchneri
Lactobacillus paralimentarius	DSM-13238	1999	Sourdough	2.55	35.1	2454	94.07	188	ERX359751	AZES00000000	Food	L. alimentarius
Lactobacillus paralimentarius	DSM-13961	1999	Fermented vegetable (Kimchi)	2.75	35.13	2597	87.32	41	ERX399760	AZDH00000000	Food	L. alimentarius
Lactobacillus paralimentarius	DSM-19674	1999	Spanish Bobal grape must	2.89	35.26	2780	83.19	56	ERX359726	AZDY00000000	Wine product	L. alimentarius
Lactobacillus paraplantarum	DSM-10667	1996	Beer contaminant	3.40	43.69	3281	35.34	252	SRX456366	AZEO00000000	Wine product	L. plantarum
Lactobacillus pasteurii	DSM-23907	2013	N/A	1.87	38.51	1794	64.24	44	SRX456269	AYZN00000000	Unknown	L. delbrueckii
Lactobacillus paucivorans	DSM-22467	2010	Yeast storage tank containing lager beer	2.37	49.06	2180	101.18	231	ERX399762	JQCA00000000	Wine product	L. brevis_L. collinoides
Lactobacillus pentosus	DSM-20314	1921	N/A	3.65	46.31	3460	32.90	60	SRX456342	AZCU00000000	Unknwon	L. plantarum
Lactobacillus perolens	DSM-12744	2000	Orange lemonade	3.31	49.08	3169	72.53	101	SRX456327	AZEC00000000	Food	L. perolens
Lactobacillus plantarum	DSM-13273	1919	Jojoba meal fermentation	3.45	44.24	3314	58.00	319	ERX399763	JQAW00000000	Plant	L. plantarum
Lactobacillus plantarum subsp. argentoratensis	DSM-16365	2005	Fermented cassava roots (fufu)	3.20	45	3024	74.94	150	ERX359752	AZFR00000000	Plant	L. plantarum
Lactobacillus plantarum subsp. plantarum	CGMCC-1.2437	1919	Pickled cabbage	3.21	44.48	3071	37.40	48	SRX456293	AZEJ00000000	Food	L. plantarum
Lactobacillus pobuzihii	NBRC-103219	2010	Pobuzih (fermented cummingcordia), Cordia di	2.35	37.72	2175	85.27	91	ERX399764	JQCN00000000	Plant	L. salivarius
Lactobacillus pobuzihii.Chen	KCTC-13174	2010	Pobuzih (fermented cummingcordia), Cordia di	2.32	37.71	2144	51.64	103	SRX456329	AZCL00000000	Plant	L. salivarius
Lactobacillus pontis	DSM-8475	1994	Rye sourdough	1.67	53.43	1638	143.66	88	ERX399765	AZGO00000000	Food	L. reuteri_L. vaccinostercus
Lactobacillus psittaci	DSM-15354	2001	Lung of parrott	1.54	35.64	1340	155.70	50	SRX456240	AZFB00000000	Animal	L. delbrueckii
Lactobacillus rapi	DSM-19907	2009	Sunki, a Japanese traditional pickle	2.86	42.95	2729	83.98	109	ERX359746	AZEI00000000	Food	L. buchneri
Lactobacillus rennini	DSM-20253	2006	Rennin	2.27	40.67	2284	105.63	125	ERX399766	AYYI00000000	Animal	L. coryniformis
Lactobacillus reuteri	DSM-20016	1982	Intestine of adult	1.94	38.63	1922	61.98	151	SRX456270	AZDD00000000	Animal	L. reuteri_L. vaccinostercus
Lactobacillus rhamnosus	DSM-20021	1968	N/A	2.95	46.68	2921	40.69	38	SRX456370	AZCQ00000000	Unknown	L. manihotivorans_L. casei
Lactobacillus rossiae	DSM-15814	2005	Wheat sourdough	2.87	43.34	2743	83.76	61	SRX456353	AZFF00000000	Food	Couple3
Lactobacillus ruminis	DSM-20403	1973	Bovine rumen	2.01	43.39	2061	59.75	115	SRX456242	AYYL00000000	Animal	L. salivarius
Lactobacillus saerimneri	DSM-16049	2004	Pig faeces	1.69	42.57	1669	70.88	92	SRX456271	AZFP00000000	Animal	L. salivarius
Lactobacillus sakei subsp. carnosus	DSM-15831	1996	Fermented meat product	1.99	41.02	1977	120.85	69	ERX399768	AZFG00000000	Food	L. sakei
Lactobacillus sakei subsp. sakei	DSM-20017	1934	"Moto" starter of sake	1.91	41.07	1900	62.82	42	SRX456367	AZDN00000000	Wine product	L. sakei
Lactobacillus salivarius	DSM-20555	1953	Saliva	1.98	32.5	1907	60.52	63	SRX456272	AYYT00000000	Animal	L. salivarius
Lactobacillus sanfranciscensis	DSM-20451	1971	Sourdough	1.23	34.7	1267	97.42	68	SRX456354	AYYM00000000	Food	L. fructivorans
Lactobacillus saniviri	DSM-24301	2012	Faeces of a Japanese healthy adult male	2.44	47.73	2395	98.43	94	ERX399769	JQCE00000000	Animal	L. manihotivorans_L. casei
Lactobacillus satsumensis	DSM-16230	2005	Shochu mash	2.65	39.94	2539	90.73	93	ERX399770	AZFQ00000000	Wine product	L. salivarius
Lactobacillus secaliphilus	DSM-17896	2007	Sourdough	1.65	47.71	1500	121.41	19	ERX359767	JQBW00000000	Food	L. reuteri_L. vaccinostercus
Lactobacillus selangorensis	ATCC-BAA-66	2000	Chili bo	2.18	46.3	2213	91.66	101	ERX359762	JQAT00000000	Food	L. sakei

Lactobacillus selangorensis	DSM-13344	2000	Chili bo	2.09	46.45	2082	95.83	32	SRX690300	JQAZ00000000	Food	L. sakei
Lactobacillus senioris	DSM-24302	2012	Faeces of a healthy 100-year-old Japanese fema	1.57	39.09	1567	153.08	16	ERX359768	AYZR00000000	Animal	L. buchneri
Lactobacillus senmaizukei	DSM-21775	2008	Senmaizuke, a Japanese pickle	2.22	48.64	2149	107.96	68	SRX456331	AYZH00000000	Food	L. brevis_L. collinoides
Lactobacillus sharpeae	DSM-20505	1982	Municipal sewage	2.45	53.38	2371	98.06	79	ERX359770	AYYO00000000	Environment	L. manihotivorans_L. casei
Lactobacillus siliginis	DSM-22696	2006	Wheat sourdough	2.07	44.08	2059	96.60	52	ERX359771	JQCB00000000	Food	Couple3
Lactobacillus similis	DSM-23365	2010	Fermented cane molasses at alcohol plants	3.49	46.99	3206	68.75	282	ERX359772	AYZM00000000	Wine product	L. brevis_L. collinoides
Lactobacillus spicheri	DSM-15429	2004	Rice sourdough	2.75	55.91	2494	87.23	51	ERX359773	AZFC00000000	Food	L. brevis_L. collinoides
Lactobacillus sucicola	DSM-21376	2009	Sap of an Oak tree	2.46	38.48	2335	97.51	31	ERX450947	AYZF00000000	Plant	L. salivarius
Lactobacillus suebicus	DSM-5007	1989	Apple mash	2.65	38.98	2517	45.26	81	SRX456334	AZGF00000000	Food	L. reuteri_L. vaccinostercus
Lactobacillus sunkii	DSM-19904	2009	Sunki, a Japanese traditional pickle	2.69	42.06	2574	89.12	79	SRX456335	AZEA00000000	Food	L. buchneri
Lactobacillus taiwanensis	DSM-21401	2009	Silage cattle feed	1.88	33.97	1851	127.73	93	ERX359776	AYZG00000000	Plant	L. delbrueckii
Lactobacillus thailandensis	DSM-22698	2007	Fermented tea leaves (miang)	2.06	53.5	1943	116.47	23	SRX456337	AYZK00000000	Plant	L. manihotivorans_L. casei
Lactobacillus tucceti	DSM-20183	2009	Sausage	2.17	34.07	2093	110.40	51	ERX359779	AZDG00000000	Food	L. alimentarius
Lactobacillus ultunensis	DSM-16047	2005	Gastric biopsies, Human stomach mucosa	2.16	35.95	2113	55.51	105	SRX456274	AZFO00000000	Animal	L. delbrueckii
Lactobacillus uvarum	DSM-19971	2009	Must of Bobal grape variety	2.69	36.88	2608	89.34	164	ERX359780	AZEG00000000	Plant	L. salivarius
Lactobacillus vaccinostercus	DSM-20634	1983	Cow dung	2.57	43.48	2485	93.56	112	ERX359731	AYYY00000000	Animal	L. reuteri_L. vaccinostercus
Lactobacillus vaginalis	DSM-5837	1989	Vaginal swab	1.79	40.46	1733	67.13	149	SRX456275	AZGL00000000	Animal	L. reuteri_L. vaccinostercus
Lactobacillus versmoldensis	DSM-14857	2003	Poultry salami	2.37	38.27	2319	50.56	62	SRX456244	AZFA00000000	Food	L. alimentarius
Lactobacillus vini	DSM-20605	2006	Must of grape	2.24	37.54	2191	53.62	269	SRX456339	AYYX00000000	Plant	L. salivarius
Lactobacillus xiangfangensis	LMG-26013	2012	Pickles	3.00	45.1	2806	80.11	145	ERX359765	JQCL00000000	Food	L. plantarum
Lactobacillus zeae	DSM-20178	1959	Corn steep liquor	3.12	47.74	3043	38.45	55	SRX456369	AZCT00000000	Wine product	L. manihotivorans_L. casei
Lactobacillus zymae	DSM-19395	2005	Artisanal wheat sourdough	2.71	53.57	2460	88.66	75	ERX399772	AZDW00000000	Food	#L. brevis_L. collinoides
Lactococcus lactis	LMG-7760	1873	Anchu mash	2.25	35.02	2237	89.07	45	ERX359766	JQCM00000000	Food	Other
Leuconostoc_argentinum	KCTC-3773	1993	Raw milk	1.72	42.89	1821	NA	98	NA	AEGQ00000000	Food	Leu_Fru
Leuconostoc_carnosum	JB16	1989	Kimchi	1.77	37.13	1711	NA	5	NA	ːP003851 - CP00385:	Food	Leu_Fru
Leuconostoc_citreum	KM20	2008	Kimchi	1.90	38.87	1866	NA	5	NA	)Q489736 - DQ48974	Food	Leu_Fru
Leuconostoc_fallax	KCTC-3537	1992	Sauerkraut	1.64	37.53	1916	NA	30	NA	AEIZ00000000	Food	Leu_Fru
Leuconostoc_gasicomitatum	LMG-18811	2001	Tomato-marinated broiler meat strips	1.95	36.66	1929	NA	1	NA	FN822744	Food	Leu_Fru
Leuconostoc_gelidum	KCTC-3527	1989	Vacuum packaged beef	1.96	36.6	1951	NA	43	NA	AEMI00000000	Food	Leu_Fru
Leuconostoc_kimchii	IMSNU-11154	2000	Kimchi	2.10	37.91	2110	NA	6	NA	ːP001753 - CP00175	Food	Leu_Fru
Leuconostoc_mesenteroides	ATCC-8293	1878	Fermenting olives	2.08	37.67	2056	NA	2	NA	C_008496, NC_0085:	Food	Leu_Fru
Leuconostoc_mesenteroides_cremoris	ATCC-19254	1929	Hansen's dried starter powder	1.74	37.9	1791	NA	29	NA	C2KK01	Unknown	Leu_Fru
Leuconostoc_pseudomesenteroides	4882	N/A	N/A	2.01	39.06	2180	NA	106	NA	CAKV00000000	Food	Leu_Fru
Oenococcus_kitaharae	DSM-17330	2006	Distilled residue of shochu mashes	1.84	42.68	1900	NA	2	NA	AFVZ00000000	Wine product	Oenococcus
Oenococcus_oeni	ATCC-BAA-1163	2002	Fermented beverages	1.75	37.94	2055	NA	62	NA	AAUV00000000	Wine product	Oenococcus
Olsenella uli	DSM-7084	1991	Human gingival crevice	2.06	64.69	1848	97.22	13	ERX399771	JQCO00000000	Animal	Other
Pediococcus acidilactici	AS1.2696	N/A	N/A	1.93	42.13	1849	51.85	18	SRX689743	JQAQ00000000	Unknown	Pediococcus
Pediococcus argentinicus	DSM-23026	2008	Fermented wheat flour	1.76	36.67	1772	56.66	93	SRX689746	JQCQ00000000	Food	Pediococcus
Pediococcus cellicola	DSM-17757	2005	Distilled pirit-fermenting cellar	2.04	39.04	1974	49.06	22	SRX689747	JQBR00000000	Wine product	Pediococcus
Pediococcus claussenii	DSM-14800	2002	Spolied beer	1.88	36.74	1807	53.21	44	SRX689748	JQBB00000000	Wine product	Pediococcus
Pediococcus damnosus	DSM-20331	1903	Lager beer yeast	2.19	38.23	2085	45.63	201	SRX689749	JQBD00000000	Wine product	Pediococcus
Pediococcus ethanolidurans	DSM-22301	2006	Walls of a distilled-spirit-fermenting cellar	2.26	37.18	2180	44.23	66	SRX689750	JQBY00000000	Wine product	Pediococcus
Pediococcus inopinatus	DSM-20285	1988	Brewery yeast	2.11	38.61	2081	47.30	157	SRX689751	JQBC00000000	Wine product	Pediococcus
Pediococcus lolii	DSM-19927	1887	Ryegrass silage	2.04	42.13	1971	49.00	31	SRX689752	JQCC00000000	Plant	Pediococcus
Pediococcus parvulus	DSM-20332	1961	Silage	3.99	40.38	3917	25.04	153	SRX689754	JQBE00000000	Plant	Pediococcus
Pediococcus pentosaceus	DSM-20336	1934	Dried American beer yeast	1.74	37.25	1687	57.49	28	SRX689755	JQBF00000000	Wine product	Pediococcus
Pediococcus stilesii	DSM-18001	2006	White maize grains	1.84	38.11	1834	54.41	47	SRX689756	JQBX00000000	Plant	Pediococcus
Weissella confusa	DSM-20196	1969	Sugar cane	2.22	44.73	2075	90.16	40	ERX359705	JQAY00000000	Plant	Weissella
Weissella halotolerans	DSM-20190	1983	Sausage	1.37	43.06	1341	146.41	12	ERX359698	JQAX00000000	Food	Weissella
Weissella kandleri	DSM-20593	1983	Desert spring	1.33	39.67	1281	149.88	21	ERX359708	JQBP00000000	Environment	Weissella
Weissella minor	DSM-20014	1983	Milking machine slime	1.77	39.29	1777	112.85	59	ERX399773	JQCD00000000	Food	Weissella
Weissella viridescens	DSM-20410	1957	Cured meat products	1.54	41.09	1525	130.11	8	ERX359781	JQBM00000000	Food	Weissella

Table S2. Sequence information for the 73 core genes

PID*	Gene	locus tag*	COG	Annotation	Co-ordinates*	Strand*	Length*
90960992	*dnaN*	LSL_0002	COG0592L	DNA polymerase III subunit beta	1532..2671	+	1140
90960995	*gyrB*	LSL_0005	COG0187L	DNA gyrase subunit B	4451..6409	+	1959
90960996	*gyrA*	LSL_0006	COG0188L	DNA gyrase subunit A	6446..8998	+	2553
90960997	*rpsF*	LSL_0007	COG0360J	30S ribosomal protein S6	9217..9507	+	291
90960998	*ssb*	LSL_0008	COG0629L	Single-strand DNA binding protein	9548..10099	+	552
90960999	*rpsR*	LSL_0009	COG0238J	30S ribosomal protein S18	10121..10357	+	237
90961180	*rpoC*	LSL_0198	COG0086K	DNA-directed RNA polymerase subunit beta'	238653..242318	+	3666
90961182	*rpsL*	LSL_0200	COG0048J	30S ribosomal protein S12	243364..243777	+	414
90961184	*efg*	LSL_0202	COG0480J	elongation factor G	244403..246496	+	2094
90961200	*trpS*	LSL_0218	COG0180J	Tryptophanyl-tRNA synthetase II	271973..272992	+	1020
90961335	*murF*	LSL_0355	COG0770M	UDP-N-acetylmuramoyl-tripeptide--D-alanyl-D-alanine ligase	387857..389227	+	1371
90961453	-	LSL_0477	COG0537FGR	bis(5'-nucleosyl)-tetraphosphatase	525874..526302	-	429
90961460	-	LSL_0484	COG0073R	tRNA-binding domain-containing protein	530707..531357	+	651
90961464	*polA*	LSL_0488	COG0749L	DNA polymerase I	536545..539223	+	2679
90961470	*thrS*	LSL_0494	COG0441J	threonyl-tRNA synthetase	543759..545708	+	1950
90961471	*infC*	LSL_0495	COG0290J	translation initiation factor IF-3	545916..546440	+	525
90961480	-	LSL_0504	COG0799S	Iojap-related protein	552036..552389	+	354
90961487	*rpsB*	LSL_0511	COG0052J	30S ribosomal protein S2	557234..558031	+	798
90961488	*tsf*	LSL_0512	COG0264J	elongation factor Ts	558125..559000	+	876
90961519	-	LSL_0543	COG0218R	GTP-binding protein	584473..585063	+	591
90961537	*pyrH*	LSL_0561	COG0528F	uridylate kinase	600230..600952	+	723
90961538	*frr*	LSL_0562	COG0233J	ribosome recycling factor	600955..601518	+	564
90961539	*uppS*	LSL_0563	COG0020I	undecaprenyl pyrophosphate synthetase	601653..602435	+	783
90961540	*cdsA*	LSL_0564	COG0575I	phosphatidate cytidylyltransferase	602438..603226	+	789
90961544	-	LSL_0568	COG0779S	hypothetical protein	610934..611407	+	474
90961545	*nusA*	LSL_0569	COG0195K	transcription elongation factor NusA	611433..612560	+	1128
90961567	-	LSL_0591	COG2890J	peptide release factor-glutamine N5-methyltransferase	634747..635586	+	840
90961569	*upp*	LSL_0593	COG0035F	uracil phosphoribosyltransferase	636708..637337	+	630
90961575	*atpG*	LSL_0599	COG0224C	F0F1 ATP synthase subunit gamma	641185..642114	+	930
90961576	*atpD*	LSL_0600	COG0055C	F0F1 ATP synthase subunit beta	642139..643545	+	1407
90961630	*typA*	LSL_0653	COG1217T	GTP-binding protein	697034..698875	+	1842
90961790	*pheS*	LSL_0813	COG0016J	phenylalanyl-tRNA synthetase subunit alpha	829184..830230	+	1047
90961836	*pfs*	LSL_0859	COG0775F	5'-methylthioadenosine nucleosidase	878731..879417	+	687
90962022	*ftsZ*	LSL_1047	COG0206D	cell division protein FtsZ	1070613..1071866	-	1254
90962026	*murD*	LSL_1051	COG0771M	UDP-N-acetylmuramoyl-L-alanyl-D-glutamate synthetase	1075412..1076788	-	1377
90962027	*mraY*	LSL_1052	COG0472M	phospho-N-acetylmuramoyl-pentapeptide-transferase	1076817..1077785	-	969
90962042	*mreC*	LSL_1067	COG1792M	rod shape-determining protein MreC	1089421..1090275	-	855
90962072	*obgE*	LSL_1097	COG0536R	GTPase ObgE	1125579..1126877	-	1299
90962084	-	LSL_1109	COG0816L	Holliday junction resolvase-like protein	1136102..1136533	-	432
90962100	*ruvB*	LSL_1125	COG2255L	Holliday junction DNA helicase RuvB	1153506..1154516	-	1011
90962101	*ruvA*	LSL_1126	COG0632L	Holliday junction DNA helicase RuvA	1154555..1155160	-	606
90962106	*pgsA*	LSL_1131	COG0558I	CDP-diacylglycerol--glycerol-3-phosphate 3-phosphatidyltransferase	1163042..1163629	-	588
90962110	*ftsK*	LSL_1135	COG1674D	cell division protein	1168139..1170418	-	2280
90962125	-	LSL_1150	COG0802R	ATP/GTP hydrolase	1183489..1183941	-	453
90962130	*smpB*	LSL_1155	COG0691O	SsrA-binding protein	1187273..1187740	-	468
90962160	*prfB*	LSL_1185	COG1186J	peptide chain release factor 2	1222388..1223455	-	1068
90962186	*groEL*	LSL_1211	COG0459O	molecular chaperone GroEL	1246385..1248007	-	1623
90962187	*groS*	LSL_1212	COG0234O	molecular chaperone GroES	1248037..1248321	-	285
90962191	*gcp*	LSL_1216	COG0533O	O-sialoglycoprotein endopeptidase	1252076..1253107	-	1032
90962198	*holB*	LSL_1223	COG2812L	DNA polymerase III subunit delta'	1257561..1258553	-	993
90962199	*tmk*	LSL_1224	COG0125F	thymidylate kinase	1258588..1259214	-	627
90962204	-	LSL_1229	COG0590FJ	cytosine/adenosine deaminase	1262491..1262991	-	501
90962212	*rplJ*	LSL_1238	COG0244J	50S ribosomal protein L10	1271311..1271814	-	504
90962215	*nusG*	LSL_1241	COG0250K	transcription antitermination protein	1273402..1273962	-	561
90962219	*spoU*	LSL_1245	COG0566J	tRNA/rRNA methyltransferase	1275013..1275759	-	747
90962221	*cysS*	LSL_1247	COG0215J	cysteinyl-tRNA synthetase	1276159..1277571	-	1413
90962278	*ppnK*	LSL_1304	COG0061G	inorganic polyphosphate/ATP-NAD kinase	1351254..1352060	-	807
90962327	-	LSL_1355	COG0037D	tRNA(Ile)-lysidine synthase TilS	1418710..1420068	-	1359
90962332	*mfd*	LSL_1360	COG1197LK	transcription-repair coupling factor	1422969..1426493	-	3525
90962333	*pth*	LSL_1361	COG0193J	peptidyl-tRNA hydrolase	1426515..1427072	-	558
90962374	*rplM*	LSL_1403	COG0102J	50S ribosomal protein L13	1478312..1478755	-	444
90962376	*cbiQ*	LSL_1405	COG0619P	cobalt transport permease	1479676..1480470	-	795
90962380	*rpoA*	LSL_1409	COG0202K	DNA-directed RNA polymerase subunit alpha	1482888..1483832	-	945
90962387	*rplO*	LSL_1416	COG0200J	50S ribosomal protein L15	1487364..1487798	-	435
90962389	*rpsE*	LSL_1418	COG0098J	30S ribosomal protein S5	1488030..1488530	-	501
90962391	*rplF*	LSL_1420	COG0097J	50S ribosomal protein L6	1488953..1489489	-	537
90962392	*rpsH*	LSL_1421	COG0096J	30S ribosomal protein S8	1489522..1489920	-	399
90962394	*rplE*	LSL_1423	COG0094J	50S ribosomal protein L5	1490153..1490695	-	543
90962400	*rpsC*	LSL_1429	COG0092J	30S ribosomal protein S3	1492402..1493058	-	657
90962404	*rplW*	LSL_1433	COG0089J	50S ribosomal protein L23	1494625..1494909	-	285
90962405	*rplD*	LSL_1434	COG0088J	50S ribosomal protein L4	1494909..1495532	-	624
90962406	*rplC*	LSL_1435	COG0087J	50S ribosomal protein L3	1495557..1496180	-	624
90962696	*rplI*	LSL_1727	COG0359J	50S ribosomal protein L9	1809124..1809573	-	450

*These columns are provided according to the reference genome*L. salivarius* UCC118

Table S3. Genera used in building the tree of bacteria

Accession No.	Phylum	Class	Order	Family	Genus	Species
NC_012483	Acidobacteria	Acidobacteria	Acidobacteriales	Acidobacteriaceae	*Acidobacterium*	*Acidobacterium capsulatum*
NC_014963	Acidobacteria	Acidobacteria	Acidobacteriales	Acidobacteriaceae	*Terriglobus*	*Terriglobus saanensis*
NC_015064	Acidobacteria	Acidobacteria	Acidobacteriales	Acidobacteriaceae	*Granulicella*	*Granulicella tundricola*
NC_008536	Acidobacteria	Solibacteres	Solibacterales	Solibacteraceae	*Candidatus Solibacter*	*Candidatus Solibacter usitatus*
NC_008009	Acidobacteria	Unclassified	Unclassified	Unclassified	*Candidatus Koribacter*	*Candidatus Koribacter versatilis*
NC_000962	Actinobacteria	Actinobacteria	Actinomycetales	Mycobacteriaceae	*Mycobacterium*	*Mycobacterium tuberculosis*
NC_002935	Actinobacteria	Actinobacteria	Actinomycetales	Corynebacteriaceae	*Corynebacterium*	*Corynebacterium diphtheriae*
NC_003155	Actinobacteria	Actinobacteria	Actinomycetales	Streptomycetaceae	*Streptomyces*	*Streptomyces avermitilis*
NC_004307	Actinobacteria	Actinobacteria	Bifidobacteriales	Bifidobacteriaceae	*Bifidobacterium*	*Bifidobacterium longum*
NC_004551	Actinobacteria	Actinobacteria	Actinomycetales	Unclassified	*Tropheryma*	*Tropheryma whipplei*
NC_006085	Actinobacteria	Actinobacteria	Actinomycetales	Propionibacteriaceae	*Propionibacterium*	*Propionibacterium acnes*
NC_006087	Actinobacteria	Actinobacteria	Actinomycetales	Microbacteriaceae	*Leifsonia*	*Leifsonia xyli*
NC_006361	Actinobacteria	Actinobacteria	Actinomycetales	Nocardiaceae	*Nocardia*	*Nocardia farcinica*
NC_007333	Actinobacteria	Actinobacteria	Actinomycetales	Nocardiopsaceae	*Thermobifida*	*Thermobifida fusca*
NC_007777	Actinobacteria	Actinobacteria	Actinomycetales	Frankiaceae	*Frankia*	*Unclassified*
NC_008148	Actinobacteria	Actinobacteria	Rubrobacterales	Rubrobacteraceae	*Rubrobacter*	*Rubrobacter xylanophilus*
NC_008268	Actinobacteria	Actinobacteria	Actinomycetales	Nocardiaceae	*Rhodococcus*	*Rhodococcus jostii*
NC_008541	Actinobacteria	Actinobacteria	Actinomycetales	Micrococcaceae	*Arthrobacter*	*Unclassified*
NC_008578	Actinobacteria	Actinobacteria	Actinomycetales	Acidothermaceae	*Acidothermus*	*Acidothermus cellulolyticus*
NC_008699	Actinobacteria	Actinobacteria	Actinomycetales	Nocardioidaceae	*Nocardioides*	*Unclassified*
NC_009142	Actinobacteria	Actinobacteria	Actinomycetales	Pseudonocardiaceae	*Saccharopolyspora*	*Saccharopolyspora erythraea*
NC_009380	Actinobacteria	Actinobacteria	Actinomycetales	Micromonosporaceae	*Salinispora*	*Salinispora tropica*
NC_009480	Actinobacteria	Actinobacteria	Actinomycetales	Microbacteriaceae	*Clavibacter*	*Clavibacter michiganensis*
NC_009664	Actinobacteria	Actinobacteria	Actinomycetales	Kineosporiaceae	*Kineococcus*	*Kineococcus radiotolerans*
NC_010168	Actinobacteria	Actinobacteria	Actinomycetales	Micrococcaceae	*Renibacterium*	*Renibacterium salmoninarum*
NC_010617	Actinobacteria	Actinobacteria	Actinomycetales	Micrococcaceae	*Kocuria*	*Kocuria rhizophila*
NC_012669	Actinobacteria	Actinobacteria	Actinomycetales	Beutenbergiaceae	*Beutenbergia*	*Beutenbergia cavernae*
NC_012803	Actinobacteria	Actinobacteria	Actinomycetales	Micrococcaceae	*Micrococcus*	*Micrococcus luteus*
NC_013093	Actinobacteria	Actinobacteria	Actinomycetales	Pseudonocardiaceae	*Actinosynnema*	*Actinosynnema mirum*
NC_013124	Actinobacteria	Actinobacteria	Acidimicrobiales	Acidimicrobiaceae	*Acidimicrobium*	*Acidimicrobium ferrooxidans*
NC_013131	Actinobacteria	Actinobacteria	Actinomycetales	Catenulisporaceae	*Catenulispora*	*Catenulispora acidiphila*
NC_013159	Actinobacteria	Actinobacteria	Actinomycetales	Pseudonocardiaceae	*Saccharomonospora*	*Saccharomonospora viridis*
NC_013165	Actinobacteria	Actinobacteria	Coriobacteriales	Coriobacteriaceae	*Slackia*	*Slackia heliotrinireducens*
NC_013169	Actinobacteria	Actinobacteria	Actinomycetales	Dermacoccaceae	*Kytococcus*	*Kytococcus sedentarius*
NC_013170	Actinobacteria	Actinobacteria	Coriobacteriales	Coriobacteriaceae	*Cryptobacterium*	*Cryptobacterium curtum*
NC_013172	Actinobacteria	Actinobacteria	Actinomycetales	Dermabacteraceae	*Brachybacterium*	*Brachybacterium faecium*
NC_013174	Actinobacteria	Actinobacteria	Actinomycetales	Jonesiaceae	*Jonesia*	*Jonesia denitrificans*
NC_013203	Actinobacteria	Actinobacteria	Coriobacteriales	Coriobacteriaceae	*Atopobium*	*Atopobium parvulum*
NC_013204	Actinobacteria	Actinobacteria	Coriobacteriales	Coriobacteriaceae	*Eggerthella*	*Eggerthella lenta*
NC_013235	Actinobacteria	Actinobacteria	Actinomycetales	Nakamurellaceae	*Nakamurella*	*Nakamurella multipartita*
NC_013510	Actinobacteria	Actinobacteria	Actinomycetales	Thermomonosporaceae	*Thermomonospora*	*Thermomonospora curvata*
NC_013521	Actinobacteria	Actinobacteria	Actinomycetales	Sanguibacteraceae	*Sanguibacter*	*Sanguibacter keddieii*
NC_013530	Actinobacteria	Actinobacteria	Actinomycetales	Promicromonosporaceae	*Xylanimonas*	*Xylanimonas cellulosilytica*
NC_013595	Actinobacteria	Actinobacteria	Actinomycetales	Streptosporangiaceae	*Streptosporangium*	*Streptosporangium roseum*
NC_013715	Actinobacteria	Actinobacteria	Actinomycetales	Micrococcaceae	*Rothia*	*Rothia mucilaginosa*
NC_013721	Actinobacteria	Actinobacteria	Bifidobacteriales	Bifidobacteriaceae	*Gardnerella*	*Gardnerella vaginalis*
NC_013739	Actinobacteria	Actinobacteria	Solirubrobacterales	Conexibacteraceae	*Conexibacter*	*Conexibacter woesei*
NC_013947	Actinobacteria	Actinobacteria	Actinomycetales	Glycomycetaceae	*Stackebrandtia*	*Stackebrandtia nassauensis*
NC_014151	Actinobacteria	Actinobacteria	Actinomycetales	Cellulomonadaceae	*Cellulomonas*	*Cellulomonas flavigena*
NC_014158	Actinobacteria	Actinobacteria	Actinomycetales	Tsukamurellaceae	*Tsukamurella*	*Tsukamurella paurometabola*
NC_014165	Actinobacteria	Actinobacteria	Actinomycetales	Pseudonocardiaceae	*Thermobispora*	*Thermobispora bispora*
NC_014168	Actinobacteria	Actinobacteria	Actinomycetales	Segniliparaceae	*Segniliparus*	*Segniliparus rotundus*
NC_014218	Actinobacteria	Actinobacteria	Actinomycetales	Actinomycetaceae	*Arcanobacterium*	*Arcanobacterium haemolyticum*
NC_014246	Actinobacteria	Actinobacteria	Actinomycetales	Actinomycetaceae	*Mobiluncus*	*Mobiluncus curtisii*
NC_014318	Actinobacteria	Actinobacteria	Actinomycetales	Pseudonocardiaceae	*Amycolatopsis*	*Amycolatopsis mediterranei*
NC_014363	Actinobacteria	Actinobacteria	Coriobacteriales	Coriobacteriaceae	*Olsenella*	*Olsenella uli*
NC_014830	Actinobacteria	Actinobacteria	Actinomycetales	Intrasporangiaceae	*Intrasporangium*	*Intrasporangium calvum*
NC_015125	Actinobacteria	Actinobacteria	Actinomycetales	Microbacteriaceae	*Microbacterium*	*Microbacterium testaceum*
NC_015312	Actinobacteria	Actinobacteria	Actinomycetales	Pseudonocardiaceae	*Pseudonocardia*	*Pseudonocardia dioxanivorans*
NC_015389	Actinobacteria	Actinobacteria	Coriobacteriales	Coriobacteriaceae	*Coriobacterium*	*Coriobacterium glomerans*
NC_015564	Actinobacteria	Actinobacteria	Actinomycetales	Mycobacteriaceae	*Amycolicicoccus*	*Amycolicicoccus subflavus*
NC_015588	Actinobacteria	Actinobacteria	Actinomycetales	Promicromonosporaceae	*Isoptericola*	*Isoptericola variabilis*
NC_015635	Actinobacteria	Actinobacteria	Actinomycetales	Propionibacteriaceae	*Microlunatus*	*Microlunatus phosphovorus*
NC_000918	Aquificae	Aquificae	Aquificales	Aquificaceae	*Aquifex*	*Aquifex aeolicus*
NC_010730	Aquificae	Aquificae	Aquificales	Hydrogenothermaceae	*Sulfurihydrogenibium*	*Unclassified*
NC_011126	Aquificae	Aquificae	Aquificales	Aquificaceae	*Hydrogenobaculum*	*Unclassified*
NC_012440	Aquificae	Aquificae	Aquificales	Hydrogenothermaceae	*Persephonella*	*Persephonella marina*
NC_013799	Aquificae	Aquificae	Aquificales	Aquificaceae	*Hydrogenobacter*	*Hydrogenobacter thermophilus*
NC_013894	Aquificae	Aquificae	Aquificales	Aquificaceae	*Thermocrinis*	*Thermocrinis albus*
NC_014926	Aquificae	Aquificae	Aquificales	Desulfurobacteriaceae	*Thermovibrio*	*Thermovibrio ammonificans*
NC_015185	Aquificae	Aquificae	Aquificales	Desulfurobacteriaceae	*Desulfurobacterium*	*Desulfurobacterium thermolithotrophum*
NC_002950	Bacteroidetes	Bacteroidia	Bacteroidales	Porphyromonadaceae	*Porphyromonas*	*Porphyromonas gingivalis*
NC_003228	Bacteroidetes	Bacteroidia	Bacteroidales	Bacteroidaceae	*Bacteroides*	*Bacteroides fragilis*
NC_009615	Bacteroidetes	Bacteroidia	Bacteroidales	Porphyromonadaceae	*Parabacteroides*	*Parabacteroides distasonis*
NC_011565	Bacteroidetes	Bacteroidia	Bacteroidales	Unclassified	*Candidatus Azobacteroides*	*Candidatus Azobacteroides pseudotrichonymphae*
NC_014033	Bacteroidetes	Bacteroidia	Bacteroidales	Prevotellaceae	*Prevotella*	*Prevotella ruminicola*
NC_014734	Bacteroidetes	Bacteroidia	Bacteroidales	Porphyromonadaceae	*Paludibacter*	*Paludibacter propionicigenes*
NC_015160	Bacteroidetes	Bacteroidia	Bacteroidales	Porphyromonadaceae	*Odoribacter*	*Odoribacter splanchnicus*
NC_008255	Bacteroidetes	Cytophagia	Cytophagales	Cytophagaceae	*Cytophaga*	*Cytophaga hutchinsonii*
NC_013037	Bacteroidetes	Cytophagia	Cytophagales	Cytophagaceae	*Dyadobacter*	*Dyadobacter fermentans*
NC_013730	Bacteroidetes	Cytophagia	Cytophagales	Cytophagaceae	*Spirosoma*	*Spirosoma linguale*
NC_014655	Bacteroidetes	Cytophagia	Cytophagales	Cytophagaceae	*Leadbetterella*	*Leadbetterella byssophila*
NC_014759	Bacteroidetes	Cytophagia	Cytophagales	Flammeovirgaceae	*Marivirga*	*Marivirga tractuosa*
NC_015703	Bacteroidetes	Cytophagia	Cytophagales	Cytophagaceae	*Runella*	*Runella slithyformis*
NC_015914	Bacteroidetes	Cytophagia	Cytophagales	Cyclobacteriaceae	*Cyclobacterium*	*Cyclobacterium marinum*
NC_008571	Bacteroidetes	Flavobacteria	Flavobacteriales	Flavobacteriaceae	*Gramella*	*Gramella forsetii*
NC_009441	Bacteroidetes	Flavobacteria	Flavobacteriales	Flavobacteriaceae	*Flavobacterium*	*Flavobacterium johnsoniae*

NC_013123	Bacteroidetes	Flavobacteria	Flavobacteriales	Unclassified	*Candidatus Sulcia*	*Candidatus Sulcia muelleri*
NC_013162	Bacteroidetes	Flavobacteria	Flavobacteriales	Flavobacteriaceae	*Capnocytophaga*	*Capnocytophaga ochracea*
NC_013222	Bacteroidetes	Flavobacteria	Flavobacteriales	Flavobacteriaceae	*Robiginitalea*	*Robiginitalea biformata*
NC_013418	Bacteroidetes	Flavobacteria	Flavobacteriales	Blattabacteriaceae	*Blattabacterium*	*Blattabacterium sp. (Periplaneta americana)*
NC_014041	Bacteroidetes	Flavobacteria	Flavobacteriales	Flavobacteriaceae	*Zunongwangia*	*Zunongwangia profunda*
NC_014230	Bacteroidetes	Flavobacteria	Flavobacteriales	Flavobacteriaceae	*Croceibacter*	*Croceibacter atlanticus*
NC_014472	Bacteroidetes	Flavobacteria	Flavobacteriales	Flavobacteriaceae	*Maribacter*	*Unclassified*
NC_014738	Bacteroidetes	Flavobacteria	Flavobacteriales	Flavobacteriaceae	*Riemerella*	*Riemerella anatipestifer*
NC_014934	Bacteroidetes	Flavobacteria	Flavobacteriales	Flavobacteriaceae	*Cellulophaga*	*Cellulophaga algicola*
NC_015144	Bacteroidetes	Flavobacteria	Flavobacteriales	Flavobacteriaceae	*Weeksella*	*Weeksella virosa*
NC_015321	Bacteroidetes	Flavobacteria	Flavobacteriales	Cryomorphaceae	*Fluviicola*	*Fluviicola taffensis*
NC_015496	Bacteroidetes	Flavobacteria	Flavobacteriales	Flavobacteriaceae	*Krokinobacter*	*Unclassified*
NC_015638	Bacteroidetes	Flavobacteria	Flavobacteriales	Flavobacteriaceae	*Lacinutrix*	*Unclassified*
NC_015844	Bacteroidetes	Flavobacteria	Flavobacteriales	Flavobacteriaceae	*Zobellia*	*Unclassified*
NC_015945	Bacteroidetes	Flavobacteria	Flavobacteriales	Flavobacteriaceae	*Muricauda*	*Muricauda ruestringensis*
NC_013061	Bacteroidetes	Sphingobacteria	Sphingobacteriales	Sphingobacteriaceae	*Pedobacter*	*Pedobacter heparinus*
NC_013132	Bacteroidetes	Sphingobacteria	Sphingobacteriales	Unclassified	*Chitinophaga*	*Chitinophaga pinensis*
NC_015277	Bacteroidetes	Sphingobacteria	Sphingobacteriales	Sphingobacteriaceae	*Sphingobacterium*	*Unclassified*
NC_015510	Bacteroidetes	Sphingobacteria	Sphingobacteriales	Saprospiraceae	*Haliscomenobacter*	*Haliscomenobacter hydrossis*
NC_007677	Bacteroidetes	Unclassified	Bacteroidetes Order II.	Rhodothermaceae	*Salinibacter*	*Salinibacter ruber*
NC_010830	Bacteroidetes	Unclassified	Unclassified	Unclassified	*Candidatus Amoebophilus*	*Candidatus Amoebophilus asiaticus*
NC_015966	Bacteroidetes	Unclassified	Bacteroidetes Order II.	Rhodothermaceae	*Rhodothermus*	*Rhodothermus marinus*
NC_000117	Chlamydiae	Chlamydiia	Chlamydiales	Chlamydiaceae	*Chlamydia*	*Chlamydia trachomatis*
NC_003361	Chlamydiae	Chlamydiia	Chlamydiales	Chlamydiaceae	*Chlamydophila*	*Chlamydophila caviae*
NC_005861	Chlamydiae	Chlamydiia	Chlamydiales	Parachlamydiaceae	*Candidatus Protochlamydia*	*Candidatus Protochlamydia amoebophila*
NC_014225	Chlamydiae	Chlamydiia	Chlamydiales	Waddliaceae	*Waddlia*	*Waddlia chondrophila*
NC_015702	Chlamydiae	Chlamydiia	Chlamydiales	Parachlamydiaceae	*Parachlamydia*	*Parachlamydia acanthamoebae*
NC_015713	Chlamydiae	Chlamydiia	Chlamydiales	Simkaniaceae	*Simkania*	*Simkania negevensis*
NC_002932	Chlorobi	Chlorobia	Chlorobiales	Chlorobiaceae	*Chlorobaculum*	*Chlorobaculum tepidum*
NC_007514	Chlorobi	Chlorobia	Chlorobiales	Chlorobiaceae	*Chlorobium*	*Chlorobium chlorochromatii*
NC_011026	Chlorobi	Chlorobia	Chlorobiales	Chlorobiaceae	*Chloroherpeton*	*Chloroherpeton thalassium*
NC_011059	Chlorobi	Chlorobia	Chlorobiales	Chlorobiaceae	*Prosthecochloris*	*Prosthecochloris aestuarii*
NC_011060	Chlorobi	Chlorobia	Chlorobiales	Chlorobiaceae	*Pelodictyon*	*Pelodictyon phaeoclathratiforme*
NC_014960	Chloroflexi	Anaerolineae	Anaerolineales	Anaerolineaceae	*Anaerolinea*	*Anaerolinea thermophila*
NC_009523	Chloroflexi	Chloroflexi	Chloroflexales	Chloroflexaceae	*Roseiflexus*	*Unclassified*
NC_009972	Chloroflexi	Chloroflexi	Herpetosiphonales	Herpetosiphonaceae	*Herpetosiphon*	*Herpetosiphon aurantiacus*
NC_010175	Chloroflexi	Chloroflexi	Chloroflexales	Chloroflexaceae	*Chloroflexus*	*Chloroflexus aurantiacus*
NC_002936	Chloroflexi	Dehalococcoidia	Dehalococcoidales	Dehalococcoidaceae	*Dehalococcoides*	*Dehalococcoides mccartyi*
NC_014314	Chloroflexi	Dehalococcoidia	Unclassified	Unclassified	*Dehalogenimonas*	*Dehalogenimonas lykanthroporepellens*
NC_011959	Chloroflexi	Thermomicrobia	Thermomicrobiales	Thermomicrobiaceae	*Thermomicrobium*	*Thermomicrobium roseum*
NC_013523	Chloroflexi	Thermomicrobia	Sphaerobacterales	Sphaerobacteraceae	*Sphaerobacter*	*Sphaerobacter thermophilus*
NC_014836	Chrysiogenetes	Chrysiogenetes	Chrysiogenales	Chrysiogenaceae	*Desulfurispirillum*	*Desulfurispirillum indicum*
NC_005125	Cyanobacteria	Gloeobacteria	Gloeobacterales	Unclassified	*Gloeobacter*	*Gloeobacter violaceus*
NC_003272	Cyanobacteria	Unclassified	Nostocales	Nostocaceae	*Nostoc*	*Unclassified*
NC_004113	Cyanobacteria	Unclassified	Chroococcales	Unclassified	*Thermosynechococcus*	*Thermosynechococcus elongatus*
NC_005042	Cyanobacteria	Unclassified	Prochlorales	Prochlorococcaceae	*Prochlorococcus*	*Prochlorococcus marinus*
NC_005070	Cyanobacteria	Unclassified	Chroococcales	Unclassified	*Synechococcus*	*Unclassified*
NC_007413	Cyanobacteria	Unclassified	Nostocales	Nostocaceae	*Anabaena*	*Anabaena variabilis*
NC_008312	Cyanobacteria	Unclassified	Oscillatoriales	Unclassified	*Trichodesmium*	*Trichodesmium erythraeum*
NC_011726	Cyanobacteria	Unclassified	Chroococcales	Unclassified	*Cyanothece*	*Unclassified*
NC_014248	Cyanobacteria	Unclassified	Nostocales	Nostocaceae	*Trichormus*	*Trichormus azollae*
NC_013939	Deferribacteres	Deferribacteres	Deferribacterales	Deferribacteraceae	*Deferribacter*	*Deferribacter desulfuricans*
NC_013943	Deferribacteres	Deferribacteres	Deferribacterales	Deferribacteraceae	*Denitrovibrio*	*Denitrovibrio acetiphilus*
NC_014758	Deferribacteres	Deferribacteres	Deferribacterales	Deferribacteraceae	*Calditerrivibrio*	*Calditerrivibrio nitroreducens*
NC_015672	Deferribacteres	Deferribacteres	Deferribacterales	Deferribacteraceae	*Flexistipes*	*Flexistipes sinusarabici*
NC_001263	Deinococcus-Thermu	Deinococci	Deinococcales	Deinococcaceae	*Deinococcus*	*Deinococcus radiodurans*
NC_005835	Deinococcus-Thermu	Deinococci	Thermales	Thermaceae	*Thermus*	*Thermus thermophilus*
NC_013946	Deinococcus-Thermu	Deinococci	Thermales	Thermaceae	*Meiothermus*	*Meiothermus ruber*
NC_014221	Deinococcus-Thermu	Deinococci	Deinococcales	Trueperaceae	*Truepera*	*Truepera radiovictrix*
NC_014761	Deinococcus-Thermu	Deinococci	Thermales	Thermaceae	*Oceanithermus*	*Oceanithermus profundus*
NC_015387	Deinococcus-Thermu	Deinococci	Thermales	Thermaceae	*Marinithermus*	*Marinithermus hydrothermalis*
NC_011297	Dictyoglomi	Dictyoglomia	Dictyoglomales	Dictyoglomaceae	*Dictyoglomus*	*Dictyoglomus thermophilum*
NC_010644	Elusimicrobia	Elusimicrobia	Elusimicrobiales	Elusimicrobiaceae	*Elusimicrobium*	*Elusimicrobium minutum*
NC_013410	Fibrobacteres	Fibrobacteria	Fibrobacterales	Fibrobacteraceae	*Fibrobacter*	*Fibrobacter succinogenes*
NC_000964	Firmicutes	Bacilli	Bacillales	Bacillaceae	*Bacillus*	*Bacillus subtilis*
NC_002662	Firmicutes	Bacilli	Lactobacillales	Streptococcaceae	*Lactococcus*	*Lactococcus lactis*
NC_002737	Firmicutes	Bacilli	Lactobacillales	Streptococcaceae	*Streptococcus*	*Streptococcus pyogenes*
NC_002745	Firmicutes	Bacilli	Bacillales	Staphylococcaceae	*Staphylococcus*	*Staphylococcus aureus*
NC_004193	Firmicutes	Bacilli	Bacillales	Bacillaceae	*Oceanobacillus*	*Oceanobacillus iheyensis*
NC_004567	Firmicutes	Bacilli	Lactobacillales	Lactobacillaceae	*Lactobacillus*	*Lactobacillus plantarum*
NC_006510	Firmicutes	Bacilli	Bacillales	Bacillaceae	*Geobacillus*	*Geobacillus kaustophilus*
NC_008525	Firmicutes	Bacilli	Lactobacillales	Lactobacillaceae	*Pediococcus*	*Pediococcus pentosaceus*
NC_008528	Firmicutes	Bacilli	Lactobacillales	Leuconostocaceae	*Oenococcus*	*Oenococcus oeni*
NC_008531	Firmicutes	Bacilli	Lactobacillales	Leuconostocaceae	*Leuconostoc*	*Leuconostoc mesenteroides*
NC_008555	Firmicutes	Bacilli	Bacillales	Listeriaceae	*Listeria*	*Listeria welshimeri*
NC_010556	Firmicutes	Bacilli	Bacillales	Unclassified	*Exiguobacterium*	*Exiguobacterium sibiricum*
NC_011567	Firmicutes	Bacilli	Bacillales	Bacillaceae	*Anoxybacillus*	*Anoxybacillus flavithermus*
NC_011999	Firmicutes	Bacilli	Bacillales	Staphylococcaceae	*Macrococcus*	*Macrococcus caseolyticus*
NC_012491	Firmicutes	Bacilli	Bacillales	Paenibacillaceae	*Brevibacillus*	*Brevibacillus brevis*
NC_012914	Firmicutes	Bacilli	Bacillales	Paenibacillaceae	*Paenibacillus*	*Unclassified*
NC_013205	Firmicutes	Bacilli	Bacillales	Alicyclobacillaceae	*Alicyclobacillus*	*Alicyclobacillus acidocaldarius*
NC_014098	Firmicutes	Bacilli	Bacillales	Alicyclobacillaceae	*Kyrpidia*	*Kyrpidia tusciae*
NC_015278	Firmicutes	Bacilli	Lactobacillales	Aerococcaceae	*Aerococcus*	*Aerococcus urinae*
NC_015391	Firmicutes	Bacilli	Lactobacillales	Carnobacteriaceae	*Carnobacterium*	*Unclassified*
NC_015516	Firmicutes	Bacilli	Lactobacillales	Enterococcaceae	*Melissococcus*	*Melissococcus plutonius*
NC_015759	Firmicutes	Bacilli	Lactobacillales	Leuconostocaceae	*Weissella*	*Weissella koreensis*
NC_003030	Firmicutes	Clostridia	Clostridiales	Clostridiaceae	*Clostridium*	*Clostridium acetobutylicum*
NC_003869	Firmicutes	Clostridia	Thermoanaerobacterales	Thermoanaerobacteraceae	*Caldanaerobacter*	*Caldanaerobacter subterraneus*
NC_006177	Firmicutes	Clostridia	Clostridiales	Clostridiales Family XVIII. In	*Symbiobacterium*	*Symbiobacterium thermophilum*
NC_007503	Firmicutes	Clostridia	Thermoanaerobacterales	Thermoanaerobacteraceae	*Carboxydothermus*	*Carboxydothermus hydrogenoformans*

NC_007644	Firmicutes	Clostridia	Thermoanaerobacterale	Thermoanaerobacteraceae	Moorella	Moorella thermoacetica
NC_007907	Firmicutes	Clostridia	Clostridiales	Peptococcaceae	Desulfitobacterium	Desulfitobacterium hafniense
NC_008346	Firmicutes	Clostridia	Clostridiales	Syntrophomonadaceae	Syntrophomonas	Syntrophomonas wolfei
NC_009253	Firmicutes	Clostridia	Clostridiales	Peptococcaceae	Desulfotomaculum	Desulfotomaculum reducens
NC_009437	Firmicutes	Clostridia	Thermoanaerobacterale	Thermoanaerobacterales Fami	Caldicellulosiruptor	Caldicellulosiruptor saccharolyticus
NC_009454	Firmicutes	Clostridia	Clostridiales	Peptococcaceae	Pelotomaculum	Pelotomaculum thermopropionicum
NC_009633	Firmicutes	Clostridia	Clostridiales	Clostridiaceae	Alkaliphilus	Alkaliphilus metalliredigens
NC_010320	Firmicutes	Clostridia	Thermoanaerobacterale	Thermoanaerobacteraceae	Thermoanaerobacter	Unclassified
NC_010337	Firmicutes	Clostridia	Clostridiales	Heliobacteriaceae	Heliobacterium	Heliobacterium modesticaldum
NC_010376	Firmicutes	Clostridia	Clostridiales	Clostridiales Family XI. Incer	Finegoldia	Finegoldia magna
NC_010424	Firmicutes	Clostridia	Clostridiales	Peptococcaceae	Candidatus Desulforudis	Candidatus Desulforudis audaxviator
NC_010718	Firmicutes	Clostridia	Natranaerobiales	Natranaerobiaceae	Natranaerobius	Natranaerobius thermophilus
NC_011295	Firmicutes	Clostridia	Thermoanaerobacterale	Thermodesulfobiaceae	Coprothermobacter	Coprothermobacter proteolyticus
NC_011899	Firmicutes	Clostridia	Halanaerobiales	Halanaerobiaceae	Halothermothrix	Halothermothrix orenii
NC_012781	Firmicutes	Clostridia	Clostridiales	Eubacteriaceae	Eubacterium	Eubacterium rectale
NC_013171	Firmicutes	Clostridia	Clostridiales	Clostridiales Family XI. Incer	Anaerococcus	Anaerococcus prevotii
NC_013385	Firmicutes	Clostridia	Thermoanaerobacterale	Thermoanaerobacteraceae	Ammonifex	Ammonifex degensii
NC_014152	Firmicutes	Clostridia	Clostridiales	Peptococcaceae	Thermincola	Thermincola potens
NC_014220	Firmicutes	Clostridia	Clostridiales	Syntrophomonadaceae	Syntrophothermus	Syntrophothermus lipocalidus
NC_014377	Firmicutes	Clostridia	Thermoanaerobacterale	Thermoanaerobacterales Fami	Thermosediminibacter	Thermosediminibacter oceani
NC_014378	Firmicutes	Clostridia	Halanaerobiales	Halobacteroidaceae	Acetohalobium	Acetohalobium arabaticum
NC_014387	Firmicutes	Clostridia	Clostridiales	Lachnospiraceae	Butyrivibrio	Butyrivibrio proteoclasticus
NC_014410	Firmicutes	Clostridia	Thermoanaerobacterale	Thermoanaerobacterales Fami	Thermoanaerobacterium	Thermoanaerobacterium thermosaccharolyticum
NC_014654	Firmicutes	Clostridia	Halanaerobiales	Halanaerobiaceae	Halanaerobium	Unclassified
NC_014828	Firmicutes	Clostridia	Clostridiales	Ruminococcaceae	Ethanoligenens	Ethanoligenens harbinense
NC_014831	Firmicutes	Clostridia	Clostridiales	Clostridiales Family XVII. Inc	Thermaerobacter	Thermaerobacter marianensis
NC_014833	Firmicutes	Clostridia	Clostridiales	Ruminococcaceae	Ruminococcus	Ruminococcus albus
NC_015172	Firmicutes	Clostridia	Clostridiales	Peptococcaceae	Syntrophobotulus	Syntrophobotulus glycolicus
NC_015275	Firmicutes	Clostridia	Clostridiales	Lachnospiraceae	Cellulosilyticum	Cellulosilyticum lentocellum
NC_015499	Firmicutes	Clostridia	Thermoanaerobacterale	Thermodesulfobiaceae	Thermodesulfobium	Thermodesulfobium narugense
NC_015519	Firmicutes	Clostridia	Thermoanaerobacterale	Thermoanaerobacteraceae	Tepidanaerobacter	Tepidanaerobacter acetatoxydans
NC_015520	Firmicutes	Clostridia	Thermoanaerobacterale	Thermoanaerobacterales Fami	Mahella	Mahella australiensis
NC_015757	Firmicutes	Clostridia	Clostridiales	Clostridiales Family XVII. Inc	Sulfobacillus	Sulfobacillus acidophilus
NC_015913	Firmicutes	Clostridia	Clostridiales	Clostridiaceae	Candidatus Arthromitus	Candidatus Arthromitus sp. SFB-mouse
NC_015977	Firmicutes	Clostridia	Clostridiales	Lachnospiraceae	Roseburia	Roseburia hominis
NC_015601	Firmicutes	Erysipelotrichia	Erysipelotrichales	Erysipelotrichaceae	Erysipelothrix	Erysipelothrix rhusiopathiae
NC_013520	Firmicutes	Negativicutes	Selenomonadales	Veillonellaceae	Veillonella	Veillonella parvula
NC_013740	Firmicutes	Negativicutes	Selenomonadales	Acidaminococcaceae	Acidaminococcus	Acidaminococcus fermentans
NC_015437	Firmicutes	Negativicutes	Selenomonadales	Veillonellaceae	Selenomonas	Selenomonas sputigena
NC_003454	Fusobacteria	Fusobacteria	Fusobacteriales	Fusobacteriaceae	Fusobacterium	Fusobacterium nucleatum
NC_013192	Fusobacteria	Fusobacteria	Fusobacteriales	Leptotrichiaceae	Leptotrichia	Leptotrichia buccalis
NC_013515	Fusobacteria	Fusobacteria	Fusobacteriales	Leptotrichiaceae	Streptobacillus	Streptobacillus moniliformis
NC_014632	Fusobacteria	Fusobacteria	Fusobacteriales	Fusobacteriaceae	Ilyobacter	Ilyobacter polytropus
NC_012489	Gemmatimonadetes	Gemmatimonadetes	Gemmatimonadales	Gemmatimonadaceae	Gemmatimonas	Gemmatimonas aurantiaca
NC_011296	Nitrospirae	Nitrospira	Nitrospirales	Nitrospiraceae	Thermodesulfovibrio	Thermodesulfovibrio yellowstonii
NC_014355	Nitrospirae	Nitrospira	Nitrospirales	Nitrospiraceae	Nitrospira	Unclassified
NC_005027	Planctomycetes	Planctomycetia	Planctomycetales	Planctomycetaceae	Rhodopirellula	Rhodopirellula baltica
NC_013720	Planctomycetes	Planctomycetia	Planctomycetales	Planctomycetaceae	Pirellula	Pirellula staleyi
NC_014148	Planctomycetes	Planctomycetia	Planctomycetales	Planctomycetaceae	Planctomyces	Planctomyces limnophilus
NC_014962	Planctomycetes	Planctomycetia	Planctomycetales	Planctomycetaceae	Isosphaera	Isosphaera pallida
NC_000963	Proteobacteria	Alphaproteobacteria	Rickettsiales	Rickettsiaceae	Rickettsia	Rickettsia prowazekii
NC_002678	Proteobacteria	Alphaproteobacteria	Rhizobiales	Phyllobacteriaceae	Mesorhizobium	Mesorhizobium loti
NC_002696	Proteobacteria	Alphaproteobacteria	Caulobacterales	Caulobacteraceae	Caulobacter	Caulobacter vibrioides
NC_003047	Proteobacteria	Alphaproteobacteria	Rhizobiales	Rhizobiaceae	Sinorhizobium	Sinorhizobium meliloti
NC_003317	Proteobacteria	Alphaproteobacteria	Rhizobiales	Brucellaceae	Brucella	Brucella melitensis
NC_003911	Proteobacteria	Alphaproteobacteria	Rhodobacterales	Rhodobacteraceae	Ruegeria	Ruegeria pomeroyi
NC_004463	Proteobacteria	Alphaproteobacteria	Rhizobiales	Bradyrhizobiaceae	Bradyrhizobium	Bradyrhizobium diazoefficiens
NC_005295	Proteobacteria	Alphaproteobacteria	Rickettsiales	Anaplasmataceae	Ehrlichia	Ehrlichia ruminantium
NC_005296	Proteobacteria	Alphaproteobacteria	Rhizobiales	Bradyrhizobiaceae	Rhodopseudomonas	Rhodopseudomonas palustris
NC_005955	Proteobacteria	Alphaproteobacteria	Rhizobiales	Bartonellaceae	Bartonella	Bartonella quintana
NC_006526	Proteobacteria	Alphaproteobacteria	Sphingomonadales	Sphingomonadaceae	Zymomonas	Zymomonas mobilis
NC_006677	Proteobacteria	Alphaproteobacteria	Rhodospirillales	Acetobacteraceae	Gluconobacter	Gluconobacter oxydans
NC_007205	Proteobacteria	Alphaproteobacteria	Unclassified	Unclassified	Candidatus Pelagibacter	Candidatus Pelagibacter ubique
NC_007406	Proteobacteria	Alphaproteobacteria	Rhizobiales	Bradyrhizobiaceae	Nitrobacter	Nitrobacter winogradskyi
NC_007493	Proteobacteria	Alphaproteobacteria	Rhodobacterales	Rhodobacteraceae	Rhodobacter	Rhodobacter sphaeroides
NC_007626	Proteobacteria	Alphaproteobacteria	Rhodospirillales	Rhodospirillaceae	Magnetospirillum	Magnetospirillum magneticum
NC_007643	Proteobacteria	Alphaproteobacteria	Rhodospirillales	Rhodospirillaceae	Rhodospirillum	Rhodospirillum rubrum
NC_007722	Proteobacteria	Alphaproteobacteria	Sphingomonadales	Erythrobacteraceae	Erythrobacter	Erythrobacter litoralis
NC_007761	Proteobacteria	Alphaproteobacteria	Rhizobiales	Rhizobiaceae	Rhizobium	Rhizobium etli
NC_007794	Proteobacteria	Alphaproteobacteria	Sphingomonadales	Sphingomonadaceae	Novosphingobium	Novosphingobium aromaticivorans
NC_007797	Proteobacteria	Alphaproteobacteria	Rickettsiales	Anaplasmataceae	Anaplasma	Anaplasma phagocytophilum
NC_007798	Proteobacteria	Alphaproteobacteria	Rickettsiales	Anaplasmataceae	Neorickettsia	Neorickettsia sennetsu
NC_007802	Proteobacteria	Alphaproteobacteria	Rhodobacterales	Rhodobacteraceae	Jannaschia	Unclassified
NC_008048	Proteobacteria	Alphaproteobacteria	Sphingomonadales	Sphingomonadaceae	Sphingopyxis	Sphingopyxis alaskensis
NC_008209	Proteobacteria	Alphaproteobacteria	Rhodobacterales	Rhodobacteraceae	Roseobacter	Roseobacter denitrificans
NC_008254	Proteobacteria	Alphaproteobacteria	Rhizobiales	Phyllobacteriaceae	Chelativorans	Unclassified
NC_008343	Proteobacteria	Alphaproteobacteria	Rhodospirillales	Acetobacteraceae	Granulibacter	Granulibacter bethesdensis
NC_008347	Proteobacteria	Alphaproteobacteria	Rhodobacterales	Hyphomonadaceae	Maricaulis	Maricaulis maris
NC_008358	Proteobacteria	Alphaproteobacteria	Rhodobacterales	Hyphomonadaceae	Hyphomonas	Hyphomonas neptunium
NC_008576	Proteobacteria	Alphaproteobacteria	Magnetococcales	Magnetococcaceae	Magnetococcus	Magnetococcus marinus
NC_009484	Proteobacteria	Alphaproteobacteria	Rhodospirillales	Acetobacteraceae	Acidiphilium	Acidiphilium cryptum
NC_009488	Proteobacteria	Alphaproteobacteria	Rickettsiales	Rickettsiaceae	Orientia	Orientia tsutsugamushi
NC_009511	Proteobacteria	Alphaproteobacteria	Sphingomonadales	Sphingomonadaceae	Sphingomonas	Sphingomonas wittichii
NC_009667	Proteobacteria	Alphaproteobacteria	Rhizobiales	Brucellaceae	Ochrobactrum	Ochrobactrum anthropi
NC_009719	Proteobacteria	Alphaproteobacteria	Rhizobiales	Rhodobiaceae	Parvibaculum	Parvibaculum lavamentivorans
NC_009720	Proteobacteria	Alphaproteobacteria	Rhizobiales	Xanthobacteraceae	Xanthobacter	Xanthobacter autotrophicus
NC_009937	Proteobacteria	Alphaproteobacteria	Rhizobiales	Xanthobacteraceae	Azorhizobium	Azorhizobium caulinodans
NC_009952	Proteobacteria	Alphaproteobacteria	Rhodobacterales	Rhodobacteraceae	Dinoroseobacter	Dinoroseobacter shibae
NC_010125	Proteobacteria	Alphaproteobacteria	Rhodospirillales	Acetobacteraceae	Gluconacetobacter	Gluconacetobacter diazotrophicus
NC_010172	Proteobacteria	Alphaproteobacteria	Rhizobiales	Methylobacteriaceae	Methylobacterium	Methylobacterium extorquens

NC_010581	Proteobacteria	Alphaproteobacteria	Rhizobiales	Beijerinckiaceae	*Beijerinckia*	*Beijerinckia indica*
NC_011144	Proteobacteria	Alphaproteobacteria	Caulobacterales	Caulobacteraceae	*Phenylobacterium*	*Phenylobacterium zucineum*
NC_011386	Proteobacteria	Alphaproteobacteria	Rhizobiales	Bradyrhizobiaceae	*Oligotropha*	*Oligotropha carboxidovorans*
NC_011666	Proteobacteria	Alphaproteobacteria	Rhizobiales	Beijerinckiaceae	*Methylocella*	*Methylocella silvestris*
NC_011985	Proteobacteria	Alphaproteobacteria	Rhizobiales	Rhizobiaceae	*Agrobacterium*	*Agrobacterium tumefaciens*
NC_012982	Proteobacteria	Alphaproteobacteria	Rhodobacterales	Hyphomonadaceae	*Hirschia*	*Hirschia baltica*
NC_012985	Proteobacteria	Alphaproteobacteria	Rhizobiales	Rhizobiaceae	*Candidatus Liberibacter*	*Candidatus Liberibacter asiaticus*
NC_013209	Proteobacteria	Alphaproteobacteria	Rhodospirillales	Acetobacteraceae	*Acetobacter*	*Acetobacter pasteurianus*
NC_014006	Proteobacteria	Alphaproteobacteria	Sphingomonadales	Sphingomonadaceae	*Sphingobium*	*Sphingobium japonicum*
NC_014010	Proteobacteria	Alphaproteobacteria	Unclassified	Unclassified	*Candidatus Puniceispirillum*	*Candidatus Puniceispirillum marinum*
NC_014217	Proteobacteria	Alphaproteobacteria	Rhizobiales	Xanthobacteraceae	*Starkeya*	*Starkeya novella*
NC_014313	Proteobacteria	Alphaproteobacteria	Rhizobiales	Hyphomicrobiaceae	*Hyphomicrobium*	*Hyphomicrobium denitrificans*
NC_014375	Proteobacteria	Alphaproteobacteria	Caulobacterales	Caulobacteraceae	*Brevundimonas*	*Brevundimonas subvibrioides*
NC_014414	Proteobacteria	Alphaproteobacteria	Parvularculales	Parvularculaceae	*Parvularcula*	*Parvularcula bermudensis*
NC_014664	Proteobacteria	Alphaproteobacteria	Rhizobiales	Hyphomicrobiaceae	*Rhodomicrobium*	*Rhodomicrobium vannielii*
NC_002927	Proteobacteria	Betaproteobacteria	Burkholderiales	Alcaligenaceae	*Bordetella*	*Bordetella bronchiseptica*
NC_002946	Proteobacteria	Betaproteobacteria	Neisseriales	Neisseriaceae	*Neisseria*	*Neisseria gonorrhoeae*
NC_003295	Proteobacteria	Betaproteobacteria	Burkholderiales	Burkholderiaceae	*Ralstonia*	*Ralstonia solanacearum*
NC_004757	Proteobacteria	Betaproteobacteria	Nitrosomonadales	Nitrosomonadaceae	*Nitrosomonas*	*Nitrosomonas europaea*
NC_005085	Proteobacteria	Betaproteobacteria	Neisseriales	Neisseriaceae	*Chromobacterium*	*Chromobacterium violaceum*
NC_006350	Proteobacteria	Betaproteobacteria	Burkholderiales	Burkholderiaceae	*Burkholderia*	*Burkholderia pseudomallei*
NC_006513	Proteobacteria	Betaproteobacteria	Rhodocyclales	Rhodocyclaceae	*Aromatoleum*	*Aromatoleum aromaticum*
NC_007298	Proteobacteria	Betaproteobacteria	Rhodocyclales	Rhodocyclaceae	*Dechloromonas*	*Dechloromonas aromatica*
NC_007404	Proteobacteria	Betaproteobacteria	Hydrogenophilales	Hydrogenophilaceae	*Thiobacillus*	*Thiobacillus denitrificans*
NC_007614	Proteobacteria	Betaproteobacteria	Nitrosomonadales	Nitrosomonadaceae	*Nitrosospira*	*Nitrosospira multiformis*
NC_007908	Proteobacteria	Betaproteobacteria	Burkholderiales	Comamonadaceae	*Albidiferax*	*Albidiferax ferrireducens*
NC_007947	Proteobacteria	Betaproteobacteria	Methylophilales	Methylophilaceae	*Methylobacillus*	*Methylobacillus flagellatus*
NC_007948	Proteobacteria	Betaproteobacteria	Burkholderiales	Comamonadaceae	*Polaromonas*	*Unclassified*
NC_007973	Proteobacteria	Betaproteobacteria	Burkholderiales	Burkholderiaceae	*Cupriavidus*	*Cupriavidus metallidurans*
NC_008702	Proteobacteria	Betaproteobacteria	Rhodocyclales	Rhodocyclaceae	*Azoarcus*	*Unclassified*
NC_008752	Proteobacteria	Betaproteobacteria	Burkholderiales	Comamonadaceae	*Acidovorax*	*Acidovorax citrulli*
NC_008786	Proteobacteria	Betaproteobacteria	Burkholderiales	Comamonadaceae	*Verminephrobacter*	*Verminephrobacter eiseniae*
NC_008825	Proteobacteria	Betaproteobacteria	Burkholderiales	Unclassified	*Methylibium*	*Methylibium petroleiphilum*
NC_009379	Proteobacteria	Betaproteobacteria	Burkholderiales	Burkholderiaceae	*Polynucleobacter*	*Polynucleobacter necessarius*
NC_010002	Proteobacteria	Betaproteobacteria	Burkholderiales	Comamonadaceae	*Delftia*	*Delftia acidovorans*
NC_010524	Proteobacteria	Betaproteobacteria	Burkholderiales	Unclassified	*Leptothrix*	*Leptothrix cholodnii*
NC_011662	Proteobacteria	Betaproteobacteria	Rhodocyclales	Rhodocyclaceae	*Thauera*	*Unclassified*
NC_012559	Proteobacteria	Betaproteobacteria	Neisseriales	Neisseriaceae	*Laribacter*	*Laribacter hongkongensis*
NC_012791	Proteobacteria	Betaproteobacteria	Burkholderiales	Comamonadaceae	*Variovorax*	*Variovorax paradoxus*
NC_012968	Proteobacteria	Betaproteobacteria	Methylophilales	Methylophilaceae	*Methylotenera*	*Methylotenera mobilis*
NC_012969	Proteobacteria	Betaproteobacteria	Methylophilales	Methylophilaceae	*Methylovorus*	*Methylovorus glucosotrophus*
NC_013194	Proteobacteria	Betaproteobacteria	Unclassified	Unclassified	*Candidatus Accumulibacter*	*Candidatus Accumulibacter phosphatis*
NC_013446	Proteobacteria	Betaproteobacteria	Burkholderiales	Comamonadaceae	*Comamonas*	*Comamonas testosteroni*
NC_013959	Proteobacteria	Betaproteobacteria	Gallionellales	Gallionellaceae	*Sideroxydans*	*Sideroxydans lithotrophicus*
NC_014153	Proteobacteria	Betaproteobacteria	Burkholderiales	Unclassified	*Thiomonas*	*Thiomonas intermedia*
NC_014323	Proteobacteria	Betaproteobacteria	Burkholderiales	Oxalobacteraceae	*Herbaspirillum*	*Herbaspirillum seropedicae*
NC_014394	Proteobacteria	Betaproteobacteria	Gallionellales	Gallionellaceae	*Gallionella*	*Gallionella capsiferriformans*
NC_014640	Proteobacteria	Betaproteobacteria	Burkholderiales	Alcaligenaceae	*Achromobacter*	*Achromobacter xylosoxidans*
NC_014910	Proteobacteria	Betaproteobacteria	Burkholderiales	Comamonadaceae	*Alicycliphilus*	*Alicycliphilus denitrificans*
NC_014914	Proteobacteria	Betaproteobacteria	Burkholderiales	Alcaligenaceae	*Taylorella*	*Taylorella equigenitalis*
NC_015458	Proteobacteria	Betaproteobacteria	Burkholderiales	Alcaligenaceae	*Pusillimonas*	*Unclassified*
NC_015677	Proteobacteria	Betaproteobacteria	Burkholderiales	Comamonadaceae	*Ramlibacter*	*Ramlibacter tataouinensis*
NC_015856	Proteobacteria	Betaproteobacteria	Burkholderiales	Oxalobacteraceae	*Collimonas*	*Collimonas fungivorans*
NC_016002	Proteobacteria	Betaproteobacteria	Neisseriales	Neisseriaceae	*Pseudogulbenkiania*	*Unclassified*
NC_002937	Proteobacteria	Deltaproteobacteria	Desulfovibrionales	Desulfovibrionaceae	*Desulfovibrio*	*Desulfovibrio vulgaris*
NC_002939	Proteobacteria	Deltaproteobacteria	Desulfuromonadales	Geobacteraceae	*Geobacter*	*Geobacter sulfurreducens*
NC_005363	Proteobacteria	Deltaproteobacteria	Bdellovibrionales	Bdellovibrionaceae	*Bdellovibrio*	*Bdellovibrio bacteriovorus*
NC_007498	Proteobacteria	Deltaproteobacteria	Desulfuromonadales	Pelobacteraceae	*Pelobacter*	*Pelobacter carbinolicus*
NC_007759	Proteobacteria	Deltaproteobacteria	Syntrophobacterales	Syntrophaceae	*Syntrophus*	*Syntrophus aciditrophicus*
NC_007760	Proteobacteria	Deltaproteobacteria	Myxococcales	Myxococcaceae	*Anaeromyxobacter*	*Anaeromyxobacter dehalogenans*
NC_008011	Proteobacteria	Deltaproteobacteria	Desulfovibrionales	Desulfovibrionaceae	*Lawsonia*	*Lawsonia intracellularis*
NC_008095	Proteobacteria	Deltaproteobacteria	Myxococcales	Myxococcaceae	*Myxococcus*	*Myxococcus xanthus*
NC_008554	Proteobacteria	Deltaproteobacteria	Syntrophobacterales	Syntrophobacteraceae	*Syntrophobacter*	*Syntrophobacter fumaroxidans*
NC_009943	Proteobacteria	Deltaproteobacteria	Desulfobacterales	Desulfobacteraceae	*Desulfococcus*	*Desulfococcus oleovorans*
NC_010162	Proteobacteria	Deltaproteobacteria	Myxococcales	Polyangiaceae	*Sorangium*	*Sorangium cellulosum*
NC_011768	Proteobacteria	Deltaproteobacteria	Desulfobacterales	Desulfobacteraceae	*Desulfatibacillum*	*Desulfatibacillum alkenivorans*
NC_012108	Proteobacteria	Deltaproteobacteria	Desulfobacterales	Desulfobacteraceae	*Desulfobacterium*	*Desulfobacterium autotrophicum*
NC_013173	Proteobacteria	Deltaproteobacteria	Desulfovibrionales	Desulfomicrobiaceae	*Desulfomicrobium*	*Desulfomicrobium baculatum*
NC_013223	Proteobacteria	Deltaproteobacteria	Desulfovibrionales	Desulfohalobiaceae	*Desulfohalobium*	*Desulfohalobium retbaense*
NC_013440	Proteobacteria	Deltaproteobacteria	Myxococcales	Kofleriaceae	*Haliangium*	*Haliangium ochraceum*
NC_014216	Proteobacteria	Deltaproteobacteria	Desulfobacterales	Desulfobulbaceae	*Desulfurivibrio*	*Desulfurivibrio alkaliphilus*
NC_014365	Proteobacteria	Deltaproteobacteria	Desulfarculales	Desulfarculaceae	*Desulfarculus*	*Desulfarculus baarsii*
NC_014972	Proteobacteria	Deltaproteobacteria	Desulfobacterales	Desulfobulbaceae	*Desulfobulbus*	*Desulfobulbus propionicus*
NC_015318	Proteobacteria	Deltaproteobacteria	Desulfurellales	Desulfurellaceae	*Hippea*	*Hippea maritima*
NC_015388	Proteobacteria	Deltaproteobacteria	Syntrophobacterales	Syntrophaceae	*Desulfobacca*	*Desulfobacca acetoxidans*
NC_002163	Proteobacteria	Epsilonproteobacteria	Campylobacterales	Campylobacteraceae	*Campylobacter*	*Campylobacter jejuni*
NC_007575	Proteobacteria	Epsilonproteobacteria	Campylobacterales	Helicobacteraceae	*Sulfurimonas*	*Sulfurimonas denitrificans*
NC_009662	Proteobacteria	Epsilonproteobacteria	Unclassified	Unclassified	*Nitratiruptor*	*Unclassified*
NC_009663	Proteobacteria	Epsilonproteobacteria	Unclassified	Unclassified	*Sulfurovum*	*Unclassified*
NC_009850	Proteobacteria	Epsilonproteobacteria	Campylobacterales	Campylobacteraceae	*Arcobacter*	*Arcobacter butzleri*
NC_011333	Proteobacteria	Epsilonproteobacteria	Campylobacterales	Helicobacteraceae	*Helicobacter*	*Helicobacter pylori*
NC_012115	Proteobacteria	Epsilonproteobacteria	Nautiliales	Nautiliaceae	*Nautilia*	*Nautilia profundicola*
NC_013512	Proteobacteria	Epsilonproteobacteria	Campylobacterales	Campylobacteraceae	*Sulfurospirillum*	*Sulfurospirillum deleyianum*
NC_014762	Proteobacteria	Epsilonproteobacteria	Campylobacterales	Helicobacteraceae	*Sulfuricurvum*	*Sulfuricurvum kujiense*
NC_014935	Proteobacteria	Epsilonproteobacteria	Campylobacterales	Unclassified	*Nitratifractor*	*Nitratifractor salsuginis*
NC_000907	Proteobacteria	Gammaproteobacteria	Pasteurellales	Pasteurellaceae	*Haemophilus*	*Haemophilus influenzae*
NC_000913	Proteobacteria	Gammaproteobacteria	Enterobacteriales	Enterobacteriaceae	*Escherichia*	*Escherichia coli*
NC_002488	Proteobacteria	Gammaproteobacteria	Xanthomonadales	Xanthomonadaceae	*Xylella*	*Xylella fastidiosa*
NC_002516	Proteobacteria	Gammaproteobacteria	Pseudomonadales	Pseudomonadaceae	*Pseudomonas*	*Pseudomonas aeruginosa*
NC_002528	Proteobacteria	Gammaproteobacteria	Enterobacteriales	Enterobacteriaceae	*Buchnera*	*Buchnera aphidicola*

NC_002663	Proteobacteria	Gammaproteobacteria	Pasteurellales	Pasteurellaceae	*Pasteurella*	*Pasteurella multocida*
NC_002971	Proteobacteria	Gammaproteobacteria	Legionellales	Coxiellaceae	*Coxiella*	*Coxiella burnetii*
NC_003143	Proteobacteria	Gammaproteobacteria	Enterobacteriales	Enterobacteriaceae	*Yersinia*	*Yersinia pestis*
NC_003197	Proteobacteria	Gammaproteobacteria	Enterobacteriales	Enterobacteriaceae	*Salmonella*	*Salmonella enterica*
NC_003902	Proteobacteria	Gammaproteobacteria	Xanthomonadales	Xanthomonadaceae	*Xanthomonas*	*Xanthomonas campestris*
NC_003910	Proteobacteria	Gammaproteobacteria	Alteromonadales	Colwelliaceae	*Colwellia*	*Colwellia psychrerythraea*
NC_004337	Proteobacteria	Gammaproteobacteria	Enterobacteriales	Enterobacteriaceae	*Shigella*	*Shigella flexneri*
NC_004344	Proteobacteria	Gammaproteobacteria	Enterobacteriales	Enterobacteriaceae	*Wigglesworthia*	*Wigglesworthia glossinidia*
NC_004347	Proteobacteria	Gammaproteobacteria	Alteromonadales	Shewanellaceae	*Shewanella*	*Shewanella oneidensis*
NC_004459	Proteobacteria	Gammaproteobacteria	Vibrionales	Vibrionaceae	*Vibrio*	*Vibrio vulnificus*
NC_004547	Proteobacteria	Gammaproteobacteria	Enterobacteriales	Enterobacteriaceae	*Pectobacterium*	*Pectobacterium atrosepticum*
NC_005061	Proteobacteria	Gammaproteobacteria	Enterobacteriales	Enterobacteriaceae	*Candidatus Blochmannia*	*Unclassified*
NC_005126	Proteobacteria	Gammaproteobacteria	Enterobacteriales	Enterobacteriaceae	*Photorhabdus*	*Photorhabdus luminescens*
NC_005966	Proteobacteria	Gammaproteobacteria	Pseudomonadales	Moraxellaceae	*Acinetobacter*	*Unclassified*
NC_006300	Proteobacteria	Gammaproteobacteria	Pasteurellales	Pasteurellaceae	*Basfia*	*Mannheimia succiniciproducens*
NC_006368	Proteobacteria	Gammaproteobacteria	Legionellales	Legionellaceae	*Legionella*	*Legionella pneumophila*
NC_006370	Proteobacteria	Gammaproteobacteria	Vibrionales	Vibrionaceae	*Photobacterium*	*Photobacterium profundum*
NC_006512	Proteobacteria	Gammaproteobacteria	Alteromonadales	Idiomarinaceae	*Idiomarina*	*Idiomarina loihiensis*
NC_006570	Proteobacteria	Gammaproteobacteria	Thiotrichales	Francisellaceae	*Francisella*	*Francisella tularensis*
NC_006840	Proteobacteria	Gammaproteobacteria	Vibrionales	Vibrionaceae	*Aliivibrio*	*Aliivibrio fischeri*
NC_007204	Proteobacteria	Gammaproteobacteria	Pseudomonadales	Moraxellaceae	*Psychrobacter*	*Psychrobacter arcticus*
NC_007481	Proteobacteria	Gammaproteobacteria	Alteromonadales	Pseudoalteromonadaceae	*Pseudoalteromonas*	*Pseudoalteromonas haloplanktis*
NC_007484	Proteobacteria	Gammaproteobacteria	Chromatiales	Chromatiaceae	*Nitrosococcus*	*Nitrosococcus oceani*
NC_007520	Proteobacteria	Gammaproteobacteria	Thiotrichales	Piscirickettsiaceae	*Thiomicrospira*	*Thiomicrospira crunogena*
NC_007645	Proteobacteria	Gammaproteobacteria	Oceanospirillales	Hahellaceae	*Hahella*	*Hahella chejuensis*
NC_007912	Proteobacteria	Gammaproteobacteria	Alteromonadales	Alteromonadaceae	*Saccharophagus*	*Saccharophagus degradans*
NC_007963	Proteobacteria	Gammaproteobacteria	Oceanospirillales	Halomonadaceae	*Chromohalobacter*	*Chromohalobacter salexigens*
NC_008260	Proteobacteria	Gammaproteobacteria	Oceanospirillales	Alcanivoracaceae	*Alcanivorax*	*Alcanivorax borkumensis*
NC_008309	Proteobacteria	Gammaproteobacteria	Pasteurellales	Pasteurellaceae	*Histophilus*	*Histophilus somni*
NC_008340	Proteobacteria	Gammaproteobacteria	Chromatiales	Ectothiorhodospiraceae	*Alkalilimnicola*	*Alkalilimnicola ehrlichii*
NC_008570	Proteobacteria	Gammaproteobacteria	Aeromonadales	Aeromonadaceae	*Aeromonas*	*Aeromonas hydrophila*
NC_008709	Proteobacteria	Gammaproteobacteria	Alteromonadales	Psychromonadaceae	*Psychromonas*	*Psychromonas ingrahamii*
NC_008740	Proteobacteria	Gammaproteobacteria	Alteromonadales	Alteromonadaceae	*Marinobacter*	*Marinobacter hydrocarbonoclasticus*
NC_008789	Proteobacteria	Gammaproteobacteria	Chromatiales	Ectothiorhodospiraceae	*Halorhodospira*	*Halorhodospira halophila*
NC_009053	Proteobacteria	Gammaproteobacteria	Pasteurellales	Pasteurellaceae	*Actinobacillus*	*Actinobacillus pleuropneumoniae*
NC_009436	Proteobacteria	Gammaproteobacteria	Enterobacteriales	Enterobacteriaceae	*Enterobacter*	*Unclassified*
NC_009648	Proteobacteria	Gammaproteobacteria	Enterobacteriales	Enterobacteriaceae	*Klebsiella*	*Klebsiella pneumoniae*
NC_009654	Proteobacteria	Gammaproteobacteria	Oceanospirillales	Oceanospirillaceae	*Marinomonas*	*Unclassified*
NC_009778	Proteobacteria	Gammaproteobacteria	Enterobacteriales	Enterobacteriaceae	*Cronobacter*	*Cronobacter sakazakii*
NC_009792	Proteobacteria	Gammaproteobacteria	Enterobacteriales	Enterobacteriaceae	*Citrobacter*	*Citrobacter koseri*
NC_009832	Proteobacteria	Gammaproteobacteria	Enterobacteriales	Enterobacteriaceae	*Serratia*	*Serratia proteamaculans*
NC_010554	Proteobacteria	Gammaproteobacteria	Enterobacteriales	Enterobacteriaceae	*Proteus*	*Proteus mirabilis*
NC_010694	Proteobacteria	Gammaproteobacteria	Enterobacteriales	Enterobacteriaceae	*Erwinia*	*Erwinia tasmaniensis*
NC_010943	Proteobacteria	Gammaproteobacteria	Xanthomonadales	Xanthomonadaceae	*Stenotrophomonas*	*Stenotrophomonas maltophilia*
NC_010995	Proteobacteria	Gammaproteobacteria	Pseudomonadales	Pseudomonadaceae	*Cellvibrio*	*Cellvibrio japonicus*
NC_011206	Proteobacteria	Gammaproteobacteria	Acidithiobacillales	Acidithiobacillaceae	*Acidithiobacillus*	*Acidithiobacillus ferrooxidans*
NC_012560	Proteobacteria	Gammaproteobacteria	Pseudomonadales	Pseudomonadaceae	*Azotobacter*	*Azotobacter vinelandii*
NC_012691	Proteobacteria	Gammaproteobacteria	Aeromonadales	Aeromonadaceae	*Tolumonas*	*Tolumonas auensis*
NC_012751	Proteobacteria	Gammaproteobacteria	Enterobacteriales	Enterobacteriaceae	*Candidatus Hamiltonella*	*Candidatus Hamiltonella defensa*
NC_012880	Proteobacteria	Gammaproteobacteria	Enterobacteriales	Enterobacteriaceae	*Dickeya*	*Dickeya dadantii*
NC_012913	Proteobacteria	Gammaproteobacteria	Pasteurellales	Pasteurellaceae	*Aggregatibacter*	*Aggregatibacter aphrophilus*
NC_012997	Proteobacteria	Gammaproteobacteria	Alteromonadales	Unclassified	*Teredinibacter*	*Teredinibacter turnerae*
NC_013166	Proteobacteria	Gammaproteobacteria	Oceanospirillales	Alcanivoracaceae	*Kangiella*	*Kangiella koreensis*
NC_013422	Proteobacteria	Gammaproteobacteria	Chromatiales	Halothiobacillaceae	*Halothiobacillus*	*Halothiobacillus neapolitanus*
NC_013851	Proteobacteria	Gammaproteobacteria	Chromatiales	Chromatiaceae	*Allochromatium*	*Allochromatium vinosum*
NC_013889	Proteobacteria	Gammaproteobacteria	Chromatiales	Ectothiorhodospiraceae	*Thioalkalivibrio*	*Unclassified*
NC_013892	Proteobacteria	Gammaproteobacteria	Enterobacteriales	Enterobacteriaceae	*Xenorhabdus*	*Xenorhabdus bovienii*
NC_013956	Proteobacteria	Gammaproteobacteria	Enterobacteriales	Enterobacteriaceae	*Pantoea*	*Pantoea ananatis*
NC_014109	Proteobacteria	Gammaproteobacteria	Enterobacteriales	Enterobacteriaceae	*Candidatus Riesia*	*Candidatus Riesia pediculicola*
NC_014147	Proteobacteria	Gammaproteobacteria	Pseudomonadales	Moraxellaceae	*Moraxella*	*Moraxella catarrhalis*
NC_014532	Proteobacteria	Gammaproteobacteria	Oceanospirillales	Halomonadaceae	*Halomonas*	*Halomonas elongata*
NC_014541	Proteobacteria	Gammaproteobacteria	Alteromonadales	Ferrimonadaceae	*Ferrimonas*	*Ferrimonas balearica*
NC_014924	Proteobacteria	Gammaproteobacteria	Xanthomonadales	Xanthomonadaceae	*Pseudoxanthomonas*	*Pseudoxanthomonas suwonensis*
NC_015061	Proteobacteria	Gammaproteobacteria	Enterobacteriales	Enterobacteriaceae	*Rahnella*	*Unclassified*
NC_015460	Proteobacteria	Gammaproteobacteria	Pasteurellales	Pasteurellaceae	*Gallibacterium*	*Gallibacterium anatis*
NC_015497	Proteobacteria	Gammaproteobacteria	Alteromonadales	Alteromonadaceae	*Glaciecola*	*Unclassified*
NC_015554	Proteobacteria	Gammaproteobacteria	Alteromonadales	Alteromonadaceae	*Alteromonas*	*Unclassified*
NC_015572	Proteobacteria	Gammaproteobacteria	Methylococcales	Methylococcaceae	*Methylomonas*	*Methylomonas methanica*
NC_015581	Proteobacteria	Gammaproteobacteria	Thiotrichales	Piscirickettsiaceae	*Thioalkalimicrobium*	*Thioalkalimicrobium cyclicum*
NC_015735	Proteobacteria	Gammaproteobacteria	Enterobacteriales	Enterobacteriaceae	*Candidatus Moranella*	*Candidatus Moranella endobia*
NC_000919	Spirochaetes	Spirochaetia	Spirochaetales	Spirochaetaceae	*Treponema*	*Treponema pallidum*
NC_001318	Spirochaetes	Spirochaetia	Spirochaetales	Spirochaetaceae	*Borrelia*	*Borrelia burgdorferi*
NC_004342	Spirochaetes	Spirochaetia	Spirochaetales	Leptospiraceae	*Leptospira*	*Leptospira interrogans*
NC_012225	Spirochaetes	Spirochaetia	Spirochaetales	Brachyspiraceae	*Brachyspira*	*Brachyspira hyodysenteriae*
NC_014364	Spirochaetes	Spirochaetia	Spirochaetales	Spirochaetaceae	*Spirochaeta*	*Spirochaeta smaragdinae*
NC_015152	Spirochaetes	Spirochaetia	Spirochaetales	Spirochaetaceae	*Sphaerochaeta*	*Sphaerochaeta globosa*
NC_013522	Synergistetes	Synergistia	Synergistales	Synergistaceae	*Thermanaerovibrio*	*Thermanaerovibrio acidaminovorans*
NC_014011	Synergistetes	Synergistia	Synergistales	Synergistaceae	*Aminobacterium*	*Aminobacterium colombiense*
NC_000908	Tenericutes	Mollicutes	Mycoplasmatales	Mycoplasmataceae	*Mycoplasma*	*Mycoplasma genitalium*
NC_002162	Tenericutes	Mollicutes	Mycoplasmatales	Mycoplasmataceae	*Ureaplasma*	*Ureaplasma parvum*
NC_005303	Tenericutes	Mollicutes	Acholeplasmatales	Acholeplasmataceae	*Candidatus Phytoplasma*	*Onion yellows phytoplasma*
NC_006055	Tenericutes	Mollicutes	Entomoplasmatales	Entomoplasmataceae	*Mesoplasma*	*Mesoplasma florum*
NC_010163	Tenericutes	Mollicutes	Acholeplasmatales	Acholeplasmataceae	*Acholeplasma*	*Acholeplasma laidlawii*
NC_015681	Thermodesulfobacte	Thermodesulfobacteria	Thermodesulfobacterial	Thermodesulfobacteriaceae	*Thermodesulfatator*	*Thermodesulfatator indicus*
NC_015682	Thermodesulfobacte	Thermodesulfobacteria	Thermodesulfobacterial	Thermodesulfobacteriaceae	*Thermodesulfobacterium*	*Thermodesulfobacterium geofontis*
NC_000853	Thermotogae	Thermotogae	Thermotogales	Thermotogaceae	*Thermotoga*	*Thermotoga maritima*
NC_009616	Thermotogae	Thermotogae	Thermotogales	Thermotogaceae	*Thermosipho*	*Thermosipho melanesiensis*
NC_009718	Thermotogae	Thermotogae	Thermotogales	Thermotogaceae	*Fervidobacterium*	*Fervidobacterium nodosum*
NC_010003	Thermotogae	Thermotogae	Thermotogales	Thermotogaceae	*Petrotoga*	*Petrotoga mobilis*
NC_012785	Thermotogae	Thermotogae	Thermotogales	Thermotogaceae	*Kosmotoga*	*Kosmotoga olearia*

NC_013525	Unclassified	Unclassified	Unclassified	Unclassified	*Thermobaculum*	*Thermobaculum terrenum*
NC_010571	Verrucomicrobia	Opitutae	Unclassified	Opitutaceae	*Opitutus*	*Opitutus terrae*
NC_014008	Verrucomicrobia	Opitutae	Puniceicoccales	Puniceicoccaceae	*Coraliomargarita*	*Coraliomargarita akajimensis*
NC_010794	Verrucomicrobia	Unclassified	Methylacidiphilales	Methylacidiphilaceae	*Methylacidiphilum*	*Methylacidiphilum infernorum*
NC_010655	Verrucomicrobia	Verrucomicrobiae	Verrucomicrobiales	Verrucomicrobiaceae	*Akkermansia*	*Akkermansia muciniphila*

Table S4. List of genes used in building the tree of bacteria

PID	Gene	Locus tag*	COG	Predicted product	Position*	Length (bp)*
90961179	*rpoB*	LSL_0197	COG0085K	DNA-directed RNA polymerase subunit beta	235025..238624	3600
90961471	*infC*	LSL_0495	COG0290J	translation initiation factor IF-3	545916..546440	525
90961487	*rpsB*	LSL_0511	COG0052J	30S ribosomal protein S2	557234..558031	798
90961488	*tsf*	LSL_0512	COG0264J	elongation factor Ts	558125..559000	876
90961538	*frr*	LSL_0562	COG0233J	ribosome recycling factor	600955..601518	564
90961545	*nusA*	LSL_0569	COG0195K	transcription elongation factor NusA	611433..612560	1128
90962130	*smpB*	LSL_1155	COG0691O	SsrA-binding protein	1187273..1187740	468
90962374	*rplM*	LSL_1403	COG0102J	50S ribosomal protein L13	1478312..1478755	444
90962389	*rpsE*	LSL_1418	COG0098J	30S ribosomal protein S5	1488030..1488530	501
90962391	*rplF*	LSL_1420	COG0097J	50S ribosomal protein L6	1488953..1489489	537
90962394	*rplE*	LSL_1423	COG0094J	50S ribosomal protein L5	1490153..1490695	543
90962399	*rplP*	LSL_1428	COG0197J	50S ribosomal protein L16	1491964..1492398	435
90962400	*rpsC*	LSL_1429	COG0092J	30S ribosomal protein S3	1492402..1493058	657
90962403	*rplB*	LSL_1432	COG0090J	50S ribosomal protein L2	1493766..1494599	834
90962405	*rplD*	LSL_1434	COG0088J	50S ribosomal protein L4	1494909..1495532	624
90962406	*rplC*	LSL_1435	COG0087J	50S ribosomal protein L3	1495557..1496180	624

*These columns are provided according to the reference genome *L. salivarius* UCC118

Table S5. Distribution of LPXTG-containing and sortase enzmyes across the 213 genomes

Species Name	StrainID	Proteins with LPXTG motif	LPXTG gene IDs	Sortase enzyme count	Sortase gene IDs	Initial LOCP output	Pilus-positive strain*	Number of clusters	Locus tags
Kandleria vitulina	DSM-20405	4	DSM_20405GL000656, DSM_20405GL000651, DSM_20405GL	7	DSM_20405GL000648, DSM_20405GL000647,	Yes	1	1	DSM_20405GL000645-DSM_20405GL000657
Lactobacillus kimchiensis	DSM-24716	6	DSM_24716GL002188, DSM_24716GL000302, DSM_24716GL	1	DSM_24716GL000255	No	0	0	
Lactobacillus mindensis	DSM-14500	9	DSM_14500GL000481, DSM_14500GL001981, DSM_14500GL	1	DSM_14500GL001396	No	0	0	
Lactobacillus nantensis	DSM-16982	8	DSM_16982GL002497, DSM_16982GL001450, DSM_16982GL	1	DSM_16982GL002198	No	0	0	
Lactobacillus crustorum	LMG-23699	6	LMG_23699GL001827, LMG_23699GL000313, LMG_23699GL	1	LMG_23699GL001530	No	0	0	
Lactobacillus crustorum	JCM-15951	6	JCM_15951GL000895, JCM_15951GL000541, JCM_15951GL0	1	JCM_15951GL001441	No	0	0	
Lactobacillus futsaii	JCM-17355	5	JCM_17355GL001149, JCM_17355GL000512, JCM_17355GL0	1	JCM_17355GL001208	No	0	0	
Lactobacillus farciminis	DSM-20184	5	DSM_20184GL000284, DSM_20184GL001314, DSM_20184GL	1	DSM_20184GL001465	No	0	0	
Lactobacillus alimentarius	DSM-20249	5	DSM_20249GL001951, DSM_20249GL000453, DSM_20249GL	1	DSM_20249GL002165	No	0	0	
Lactobacillus paralimentarius	DSM-19674	6	DSM_19674GL002499, DSM_19674GL002023, DSM_19674GL	1	DSM_19674GL001187	No	0	0	
Lactobacillus paralimentarius	DSM-13961	6	DSM_13961GL000684, DSM_13961GL002440, DSM_13961GL	1	DSM_13961GL002182	No	0	0	
Lactobacillus paralimentarius	DSM-13238	5	DSM_13238GL001227, DSM_13238GL000164, DSM_13238GL	1	DSM_13238GL000661	No	0	0	
Lactobacillus tucceti	DSM-20183	6	DSM_20183GL001473, DSM_20183GL000618, DSM_20183GL	1	DSM_20183GL000199	No	0	0	
Lactobacillus nodensis	DSM-19682	7	DSM_19682GL001072, DSM_19682GL000295, DSM_19682GL	1	DSM_19682GL000261	No	0	0	
Lactobacillus versmoldensis	DSM-14857	4	DSM_14857GL001286, DSM_14857GL002013, DSM_14857GL	1	DSM_14857GL000145	No	0	0	
Lactobacillus floricola	DSM-23037	0	0	1	DSM_23037GL000896	No	0	0	
Lactobacillus acidophilus	DSM-20079	11	DSM_20079GL000490, DSM_20079GL001512, DSM_20079GL	1	DSM_20079GL000934	No	0	0	
Lactobacillus amylovorus	DSM-20531	10	DSM_20531GL000807, DSM_20531GL001919, DSM_20531GL	1	DSM_20531GL000577	No	0	0	
Lactobacillus amylovorus	DSM-16698	6	DSM_16698GL001813, DSM_16698GL000171, DSM_16698GL	1	DSM_16698GL001620	No	0	0	
Lactobacillus kitasatonis	DSM-16761	11	DSM_16761GL001138, DSM_16761GL001485, DSM_16761GL	1	DSM_16761GL001604	No	0	0	
Lactobacillus kefiranofaciens kefirano	DSM-5016	8	DSM_5016GL001593, DSM_5016GL001467, DSM_5016GL00	2	DSM_5016GL000350, DSM_5016GL000099	No	0	0	
Lactobacillus kefiranofaciens kefirgra	DSM-10550	4	DSM_10550GL001210, DSM_10550GL002011, DSM_10550GL	1	DSM_10550GL000062	No	0	0	
Lactobacillus ultunensis	DSM-16047	3	DSM_16047GL001570, DSM_16047GL001218, DSM_16047GL	1	DSM_16047GL000409	No	0	0	
Lactobacillus helveticus	LMG-22464	9	LMG_22464GL001772, LMG_22464GL001551, LMG_22464GL	1	LMG_22464GL000006	No	0	0	
Lactobacillus helveticus	CGMCC-1.1877	5	CGMCC_1.1877GL000568, CGMCC_1.1877GL000883, CGMC	1	CGMCC_1.1877GL000871	No	0	0	
Lactobacillus gallinarum	DSM-10532	11	DSM_10532GL001210, DSM_10532GL000798, DSM_10532GL	1	DSM_10532GL001659	No	0	0	
Lactobacillus crispatus	DSM-20584	11	DSM_20584GL000238, DSM_20584GL000824, DSM_20584GL	1	DSM_20584GL001854	No	0	0	
Lactobacillus acetotolerans	DSM-20749	0	0	1	DSM_20749GL001333	Yes	0	0	
Lactobacillus intestinalis	DSM-6629	13	DSM_6629GL000820, DSM_6629GL001159, DSM_6629GL001	1	DSM_6629GL000007	Yes	0	0	
Lactobacillus hamsteri	DSM-5661	10	DSM_5661GL000059, DSM_5661GL001728, DSM_5661GL00	1	DSM_5661GL000439	No	0	0	
Lactobacillus amylolyticus	DSM-11664	2	DSM_11664GL001247, DSM_11664GL000217	1	DSM_11664GL000186	No	0	0	
Lactobacillus kalixensis	DSM-16043	10	DSM_16043GL001402, DSM_16043GL000893, DSM_16043GL	1	DSM_16043GL001759	No	0	0	
Lactobacillus gigeriorum	DSM-23908	9	DSM_23908GL001420, DSM_23908GL000570, DSM_23908GL	1	DSM_23908GL000896	No	0	0	
Lactobacillus pasteurii	DSM-23907	11	DSM_23907GL001563, DSM_23907GL001030, DSM_23907GL	1	DSM_23907GL000159	No	0	0	
Lactobacillus delbrueckii jakobsenii	DSM-26046	1	DSM_26046GL001229	2	DSM_26046GL000643, DSM_26046GL000289	No	0	0	
Lactobacillus delbrueckii lactis	DSM-20072	2	DSM_20072GL001164, DSM_20072GL000182	2	DSM_20072GL000714, DSM_20072GL001764	No	0	0	
Lactobacillus delbrueckii bulgaricus	DSM-20081	2	DSM_20081GL001481, DSM_20081GL001900	1	DSM_20081GL001567	No	0	0	
Lactobacillus delbrueckii delbrueckii	DSM-20074	2	DSM_20074GL000853, _DSM_20074GL001664	1	DSM_20074GL001418	No	0	0	
Lactobacillus delbrueckii indicus	DSM-15996	1	DSM_15996GL000211	2	DSM_15996GL000941, DSM_15996GL001548	No	0	0	
Lactobacillus equicursoris	DSM-19284	0	0	3	DSM_19284GL000558, DSM_19284GL000191,	Yes	1	1	DSM_19284GL000189-DSM_19284GL000193
Lactobacillus jensenii	DSM-20557	10	DSM_20557GL000947, DSM_20557GL000697, DSM_20557GL	1	DSM_20557GL000120	No	0	0	
Lactobacillus psittaci	DSM-15354	14	DSM_15354GL001320, DSM_15354GL001152, DSM_15354GL	1	DSM_15354GL000286	Yes	0	0	
Lactobacillus hominis	DSM-23910	16	DSM_23910GL001758, DSM_23910GL000242, DSM_23910GL	1	DSM_23910GL000185	Yes	0	0	
Lactobacillus taiwanensis	DSM-21401	8	DSM_21401GL000754, DSM_21401GL001759, DSM_21401GL	1	DSM_21401GL000422	No	0	0	
Lactobacillus johnsonii	ATCC-33200	8	ATCC_33200GL000689, ATCC_33200GL001379, ATCC_3320	1	ATCC_33200GL000369	No	0	0	
Lactobacillus gasseri	ATCC-33323	9	ATCC_3332300450, ATCC_3332300166, ATCC_3332301827,	1	ATCC_3332300912	No	0	0	
Lactobacillus iners	DSM-13335	6	DSM_13335GL000092, DSM_13335GL000001, DSM_13335GL	1	DSM_13335GL000307	No	0	0	
Lactobacillus amylotrophicus	DSM-20534	6	DSM_20534GL001366, DSM_20534GL000953, DSM_20534GL	4	DSM_20534GL001368, DSM_20534GL001384,	Yes	1	1	DSM_20534GL000078-DSM_20534GL000080
Lactobacillus amylophilus	DSM-20533	6	DSM_20533GL000200, DSM_20533GL000760, DSM_20533GL	4	DSM_20533GL000713, DSM_20533GL001214,	Yes	1	1	DSM_20533GL000759-DSM_20533GL000761
Lactobacillus dextrinicus	DSM-20335	4	DSM_20335GL000025, DSM_20335GL000538, DSM_20335GL	1	DSM_20335GL001519	No	0	0	
Lactobacillus concavus	DSM-17758	4	DSM_17758GL000815, DSM_17758GL000814, DSM_17758GL	2	DSM_17758GL000818, DSM_17758GL000939	Yes	1	1	DSM_17758GL000814-DSM_17758GL000818
Lactobacillus composti	DSM-18527	6	DSM_18527GL002080, DSM_18527GL002347, DSM_18527GL	2	DSM_18527GL000267, DSM_18527GL002269	Yes	1	1	DSM_18527GL000264-DSM_18527GL000267
Lactobacillus harbinensis	DSM-16991	5	DSM_16991GL002809, DSM_16991GL000067, DSM_16991GL	7	DSM_16991GL000066, DSM_16991GL000067,	Yes	1	1	DSM_16991GL002440-DSM_16991GL002444
Lactobacillus perolens	DSM-12744	11	DSM_12744GL002691, DSM_12744GL000201, DSM_12744GL	3	DSM_12744GL003091, DSM_12744GL003090,	Yes	1	1	DSM_12744GL003087-DSM_12744GL003091
Lactobacillus camelliae	DSM-22697	16	DSM_22697GL001249, DSM_22697GL001930, DSM_22697GL	2	DSM_22697GL001227, DSM_22697GL001529	Yes	1	1	DSM_22697GL001224-DSM_22697GL001227
Lactobacillus nasuensis	JCM-17158	20	JCM_17158GL002113, JCM_17158GL001381, JCM_17158GL0	5	JCM_17158GL000214, JCM_17158GL001503, JC	Yes	1	3	JCM_17158GL001379-JCM_17158GL001384,JCM_17158GL001503-JC
Lactobacillus manihotivorans	DSM-13343	9	DSM_13343GL001150, DSM_13343GL002810, DSM_13343GL	7	DSM_13343GL001490, DSM_13343GL001151,	Yes	1	4	DSM_13343GL000818-DSM_13343GL000822, DSM_13343GL001392-
Lactobacillus paracasei paracasei	DSM-5622	9	DSM_5622GL002008, DSM_5622GL002113, DSM_5622GL001	4	DSM_5622GL000483, DSM_5622GL001345, DS	Yes	1	3	DSM_5622GL002077-DSM_5622GL002080, DSM_5622GL002703-DS
Lactobacillus casei	DSM-20011	11	DSM_20011GL001051, DSM_20011GL000676, DSM_20011GL	4	DSM_20011GL000675, DSM_20011GL001014,	Yes	1	2	DSM_20011GL000675-DSM_20011GL000679 & DSM_20011GL001884
Lactobacillus paracasei tolerans	DSM-20258	6	DSM_20258GL001272, DSM_20258GL000196, DSM_20258GL	3	DSM_20258GL000472, DSM_20258GL000385,	Yes	1	1	DSM_20258GL001269-DSM_20258GL001273
Lactobacillus zeae	DSM-20178	15	DSM_20178GL001213, DSM_20178GL002422, DSM_20178GL	1	DSM_20178GL000621	Yes	0	0	
Lactobacillus rhamnosus	DSM-20021	11	DSM_20021GL001756, DSM_20021GL000043, DSM_20021GL	2	DSM_20021GL002528, DSM_20021GL000983	Yes	1	1	DSM_20021GL000983-DSM_20021GL000986
Lactobacillus saniviri	DSM-24301	4	DSM_24301GL001627, DSM_24301GL002208, DSM_24301GL	2	DSM_24301GL000206, DSM_24301GL000570	No	0	0	
Lactobacillus brantae	DSM-23927	13	DSM_23927GL001495, DSM_23927GL001391, DSM_23927GL	5	DSM_23927GL001737, DSM_23927GL001396,	Yes	1	1	DSM_23927GL001391-DSM_23927GL001396
Lactobacillus thailandensis	DSM-22698	11	DSM_22698GL000980, DSM_22698GL001891, DSM_22698GL	6	DSM_22698GL000953, DSM_22698GL000950,	Yes	1	2	DSM_22698GL000947-DSM_22698GL000951, DSM_22698GL001891-
Lactobacillus pantheris	DSM-15945	13	DSM_15945GL002217, DSM_15945GL001936, DSM_15945GL	5	DSM_15945GL001147, DSM_15945GL001146,	Yes	1	2	DSM_15945GL000317-DSM_15945GL000320, DSM_15945GL001143-
Lactobacillus sharpeae	DSM-20505	17	DSM_20505GL000259, DSM_20505GL001026, DSM_20505GL	5	DSM_20505GL001290, DSM_20505GL000365,	Yes	1	5	DSM_20505GL001771-DSM_20505GL001774, DSM_20505GL001023-
Lactobacillus selangorensis	DSM-13344	6	DSM_13344GL000084, DSM_13344GL001050, DSM_13344GL	4	DSM_13344GL001052, DSM_13344GL000915,	Yes	1	1	DSM_13344GL001049-DSM_13344GL001052
Lactobacillus selangorensis	ATCC-BAA-66	6	ATCC_BAA-66GL001894, ATCC_BAA-66GL000803, ATCC_	4	ATCC_BAA-66GL001277, ATCC_BAA-66GL00	Yes	1	1	ATCC_BAA-66GL000801-ATCC_BAA-66GL000804
Lactobacillus graminis	DSM-20719	4	DSM_20719GL001158, DSM_20719GL000066, DSM_20719GL	2	DSM_20719GL000709, DSM_20719GL000591	No	0	0	
Lactobacillus curvatus	DSM-20019	2	DSM_20019GL001659, DSM_20019GL000479	1	DSM_20019GL000931	No	0	0	
Lactobacillus sakei sakei	DSM-20017	3	DSM_20017GL000885, DSM_20017GL001848, DSM_20017GL	2	DSM_20017GL001603, DSM_20017GL001759	No	0	0	
Lactobacillus sakei carnosus	DSM-15831	2	DSM_15831GL001730, DSM_15831GL001597	2	DSM_15831GL001007, DSM_15831GL000783	No	0	0	
Lactobacillus fuchuensis	DSM-14340	11	DSM_14340GL001061, DSM_14340GL000533, DSM_14340GL	2	DSM_14340GL001064, DSM_14340GL001839	No	0	0	
Lactobacillus rennini	DSM-20253	1	DSM_20253GL001773	1	DSM_20253GL001147	No	0	0	
Lactobacillus coryniformis torquens	DSM-20004	6	DSM_20004GL001254, DSM_20004GL001644, DSM_20004GL	1	DSM_20004GL001323	No	0	0	
Lactobacillus coryniformis coryniform	DSM-20001	3	DSM_20001GL001561, DSM_20001GL001914, DSM_20001GL	1	DSM_20001GL002353	No	0	0	
Lactobacillus bifermentans	DSM-20003	19	DSM_20003GL003041, DSM_20003GL001394, DSM_20003GL	1	DSM_20003GL000268	No	0	0	
Lactobacillus ceti	DSM-22408	12	DSM_22408GL001179, DSM_22408GL000237, DSM_22408GL	1	DSM_22408GL000079	Yes	0	0	
Lactobacillus saerimneri	DSM-16049	11	DSM_16049GL000506, DSM_16049GL000767, DSM_16049GL	1	DSM_16049GL000237	No	0	0	
Lactobacillus animalis	DSM-20602	7	DSM_20602GL001492, DSM_20602GL000660, DSM_20602GL	2	DSM_20602GL000309, DSM_20602GL000659	Yes	1	1	DSM_20602GL000659-DSM_20602GL000662
Lactobacillus murinus	DSM-20452	14	DSM_20452GL001615, DSM_20452GL001141, DSM_20452GL	4	DSM_20452GL000752, DSM_20452GL001251,	Yes	1	2	DSM_20452GL001251-DSM_20452GL001254, DSM_20452GL001778-
Lactobacillus apodemi	DSM-16634	17	DSM_16634GL001785, DSM_16634GL000537, DSM_16634GL	2	DSM_16634GL000825, DSM_16634GL000209	Yes	1	1	DSM_16634GL000822-DSM_16634GL000825
Lactobacillus ruminis	DSM-20403	2	DSM_20403GL001548, DSM_20403GL001549	2	DSM_20403GL001547, DSM_20403GL001202	Yes	1	1	DSM_20403GL001547-DSM_20403GL001550
Lactobacillus agilis	DSM-20509	1	DSM_20509GL000672	1	DSM_20509GL000133	No	0	0	
Lactobacillus equi	DSM-15833	10	DSM_15833GL000904, DSM_15833GL001458, DSM_15833GL	1	DSM_15833GL000512	No	0	0	
Lactobacillus salivarius	DSM-20555	10	DSM_20555GL001355, DSM_20555GL001345, DSM_20555GL	3	DSM_20555GL000340, DSM_20555GL001342,	Yes	1	1	DSM_20555GL001342-DSM_20555GL001346
Lactobacillus hayakitensis	DSM-18933	12	DSM_18933GL001209, DSM_18933GL001131, DSM_18933GL	1	DSM_18933GL001461	No	0	0	
Lactobacillus pobuzihii	NBRC-103219	1	NBRC_103219GL001356	1	NBRC_103219GL001355	Yes	1	1	NBRC_103219GL001355-NBRC_103219GL001360
Lactobacillus pobuzihii.Chen	KCTC-13174	1	KCTC_13174GL000405	1	KCTC_13174GL000406	Yes	1	1	KCTC_13174GL000401-KCTC_13174GL000406
Lactobacillus acidipiscis	DSM-15836	0	0	0	0	Yes	0	0	
Lactobacillus acidipiscis	DSM-15353	0	0	0	0	No	0	0	
Lactobacillus aviarius aviarius	DSM-20655	15	DSM_20655GL000800, DSM_20655GL000912, DSM_20655GL	2	DSM_20655GL001022, DSM_20655GL000842	Yes	0	0	
Lactobacillus aviarius araffinosus	DSM-20653	4	DSM_20653GL000101, DSM_20653GL000406, DSM_20653GL	3	DSM_20653GL000808, DSM_20653GL001336,	No	0	0	
Lactobacillus sucicola	DSM-21376	4	DSM_21376GL002225, DSM_21376GL000824, DSM_21376GL	1	DSM_21376GL000924	No	0	0	
Lactobacillus aquaticus	DSM-21051	5	DSM_21051GL001805, DSM_21051GL002236, DSM_21051GL	1	DSM_21051GL001874	No	0	0	
Lactobacillus uvarum	DSM-19971	4	DSM_19971GL001019, DSM_19971GL002388, DSM_19971GL	1	DSM_19971GL002229	No	0	0	
Lactobacillus capillatus	DSM-19910	2	DSM_19910GL002045, DSM_19910GL001092	1	DSM_19910GL001950	No	0	0	
Lactobacillus cacaonum	DSM-21116	3	DSM_21116GL000555, DSM_21116GL001136, DSM_21116GL	1	DSM_21116GL001133	Yes	0	0	
Lactobacillus mali	DSM-20444	4	DSM_20444GL002356, DSM_20444GL001903, DSM_20444GL	1	DSM_20444GL002197	No	0	0	
Lactobacillus mali	ATCC-27304	2	ATCC_27304GL000830, ATCC_27304GL000316	1	ATCC_27304GL000170	No	0	0	
Lactobacillus hordei	DSM-19519	2	DSM_19519GL000851, DSM_19519GL000949	1	DSM_19519GL001591	No	0	0	
Lactobacillus oeni	DSM-19972	1	DSM_19972GL000478	1	DSM_19972GL000450	No	0	0	
Lactobacillus satsumensis	DSM-16230	3	DSM_16230GL001686, DSM_16230GL001897, DSM_16230GL	1	DSM_16230GL001163	No	0	0	
Lactobacillus vini	DSM-20605	1	DSM_20605GL001645	1	DSM_20605GL001364	No	0	0	
Lactobacillus ghanensis	DSM-18630	5	DSM_18630GL000918, DSM_18630GL000355, DSM_18630GL	1	DSM_18630GL001725	No	0	0	
Lactobacillus nagelii	DSM-13675	3	DSM_13675GL000470, DSM_13675GL001778, DSM_13675GL	1	DSM_13675GL001651	No	0	0	
Lactobacillus algidus	DSM-15638	7	DSM_15638GL001423, DSM_15638GL000902, DSM_15638GL	1	DSM_15638GL001287	No	0	0	
Lactobacillus fabifermentans	DSM-21115	14	DSM_21115GL001291, DSM_21115GL000394, DSM_21115GL	1	DSM_21115GL000343	No	0	0	
Lactobacillus xiangfangensis	LMG-26013	17	LMG_26013GL002218, LMG_26013GL001093, LMG_26013GL	1	LMG_26013GL002749	No	0	0	
Lactobacillus pentosus	DSM-20314	17	DSM_20314GL000260, DSM_20314GL002586, DSM_20314GL	1	DSM_20314GL002810	No	0	0	
Lactobacillus plantarum argentoratens	DSM-16365	16	DSM_16365GL001172, DSM_16365GL000907, DSM_16365GL	1	DSM_16365GL000737	No	0	0	
Lactobacillus plantarum	DSM-13273	17	DSM_13273GL003164, DSM_13273GL003307, DSM_13273GL	1	DSM_13273GL002714	No	0	0	
Lactobacillus plantarum plantarum	CGMCC-1.2437	17	CGMCC_1.2437GL001848, CGMCC_1.2437GL002767, CGMC	1	CGMCC_1.2437GL000951	No	0	0	
Lactobacillus paraplantarum	DSM-10667	12	DSM_10667GL000604, DSM_10667GL003005, DSM_10667GL	2	DSM_10667GL002231, DSM_10667GL001666	No	0	0	
Lactobacillus siliginis	DSM-22696	4	DSM_22696GL000946, DSM_22696GL000633, DSM_22696GL	1	DSM_22696GL001637	Yes	0	0	
Lactobacillus rossiae	DSM-15814	12	DSM_15814GL002690, DSM_15814GL000659, DSM_15814GL	1	DSM_15814GL002440	No	0	0	
Weissella viridescens	DSM-20410	7	DSM_20410GL000919, DSM_20410GL001336, DSM_20410GL	1	DSM_20410GL000457	No	0	0	
Weissella minor	DSM-20014	6	DSM_20014GL001262, DSM_20014GL001263, DSM_20014GL	1	DSM_20014GL000152	No	0	0	
Weissella halotolerans	DSM-20190	1	DSM_20190GL000797	1	DSM_20190GL000080	No	0	0	
Weissella confusa	DSM-20196	2	DSM_20196GL002068, DSM_20196GL000011	3	DSM_20196GL000684, DSM_20196GL001573,	Yes	1	1	DSM_20196GL000684-DSM_20196GL000687
Weissella kandleri	DSM-20593	1	DSM_20593GL000407	0	0	No	0	0	
Oenococcus oeni	ATCC-BAA-116	0	0	0	0	No	0	0	
Oenococcus kitaharae	DSM-17330	4	DSM_17330GL00021, DSM_17330GL02268, DSM_17330GL0	2	DSM_17330GL00019, DSM_17330GL00020	Yes	1	1	DSM_17330GL000019-DSM_17330GL000025
Leuconostoc fallax	KCTC-3537	1	KCTC_3537GL01532	0	0	No	0	0	
Leuconostoc pseudomesenteroides	4882	2	4882GL00444, 4882GL01575	0	0	No	0	0	
Leuconostoc mesenteroides	ATCC-8293	1	ATCC_8293GL01689	2	ATCC_8293GL01687, ATCC_8293GL01686	Yes	1	1	ATCC_8293GL001686-ATCC_8293GL001691
Leuconostoc mesenteroides cremoris	ATCC-19254	0	0	1	ATCC_19254GL00587	No	0	0	
Leuconostoc carnosum	JB16	3	JB16GL01760, JB16GL00061, JB16GL01763	2	JB16GL01758, JB16GL01759	Yes	1	1	JB16GL001758-JB16GL001763
Leuconostoc argentinum	KCTC-3773	2	KCTC_3773GL00587, KCTC_3773GL01754	1	KCTC_3773GL01755	No	0	0	
Leuconostoc citreum	KM20	1	KM20GL00082	0	0	No	0	0	
Leuconostoc gelidum	KCTC-3527	1	KCTC_3527GL00516	0	0	No	0	0	
Leuconostoc gasicomitatum	LMG-18811	0	0	0	0	No	0	0	
Leuconostoc kimchii	IMSNU-11154	3	IMSNU_11154GL01197, IMSNU_11154GL02005, IMSNU_111	2	IMSNU_11154GL01825, IMSNU_11154GL01824	Yes	1	1	IMSNU_11154GL001821-IMSNU_11154GL001825
Fructobacillus fructosus	DSM-20349	0	0	0	0	No	0	0	
Lactobacillus pontis	DSM-8475	7	DSM_8475GL000768, DSM_8475GL000331, DSM_8475GL00	1	DSM_8475GL001343	No	0	0	
Lactobacillus panis	DSM-6035	3	DSM_6035GL001091, DSM_6035GL001738, DSM_6035GL001	1	DSM_6035GL000046	No	0	0	
Lactobacillus oris	DSM-4864	17	DSM_4864GL001046, DSM_4864GL001819, DSM_4864GL001	1	DSM_4864GL000568	No	0	0	
Lactobacillus antri	DSM-16041	26	DSM_16041GL001256, DSM_16041GL000013, DSM_16041GL	2	DSM_16041GL001094, DSM_16041GL000799	Yes	0	0	
Lactobacillus reuteri	DSM-20016	5	DSM_20016GL000247, DSM_20016GL001914, DSM_20016GL	1	DSM_20016GL001231	No	0	0	
Lactobacillus vaginalis	DSM-5837	5	DSM_5837GL000330, DSM_5837GL000015, DSM_5837GL00	1	DSM_5837GL001172	No	0	0	
Lactobacillus frumenti	DSM-13145	3	DSM_13145GL000149, DSM_13145GL001023, DSM_13145GL	1	DSM_13145GL001525	No	0	0	
Lactobacillus fermentum	DSM-20055	5	DSM_20055GL000692, DSM_20055GL001065, DSM_20055GL	1	DSM_20055GL001680	No	0	0	
Lactobacillus equigenerosi	DSM-18793	5	DSM_18793GL001526, DSM_18793GL000962, DSM_18793GL	1	DSM_18793GL001354	No	0	0	
Lactobacillus gastricus	DSM-16045	3	DSM_16045GL000205, DSM_16045GL000773, DSM_16045GL	1	DSM_16045GL000048	No	0	0	
Lactobacillus ingluviei	DSM-15946	8	DSM_15946GL000381, DSM_15946GL000686, DSM_15946GL	1	DSM_15946GL000241	No	0	0	
Lactobacillus ingluviei	DSM-14792	14	DSM_14792GL001808, DSM_14792GL000615, DSM_14792GL	1	DSM_14792GL000402	No	0	0	
Lactobacillus secaliphilus	DSM-17896	6	DSM_17896GL000271, DSM_17896GL000056, DSM_17896GL	1	DSM_17896GL001397	No	0	0	
Lactobacillus coleohominis	DSM-14060	15	DSM_14060GL000186, DSM_14060GL001520, DSM_14060GL	1	DSM_14060GL001320	No	0	0	
Lactobacillus mucosae	DSM-13345	15	DSM_13345GL001360, DSM_13345GL002094, DSM_13345GL	1	DSM_13345GL000548	No	0	0	
Lactobacillus oligofermentans	DSM-15707	5	DSM_15707GL000001, DSM_15707GL001343, DSM_15707GL	2	DSM_15707GL000087, DSM_15707GL000044	No	0	0	
Lactobacillus hokkaidonensis	DSM-26202	13	DSM_26202GL001556, DSM_26202GL002130, DSM_26202GL	4	DSM_26202GL001535, DSM_26202GL001536,	Yes	1	1	DSM_26202GL001535-DSM_26202GL001538
Lactobacillus suebicus	DSM-5007	15	DSM_5007GL000525, DSM_5007GL001404, DSM_5007GL00	1	DSM_5007GL002085	No	0	0	
Lactobacillus vaccinostercus	DSM-20634	7	DSM_20634GL001369, DSM_20634GL001300, DSM_20634GL	1	DSM_20634GL001201	No	0	0	
Lactobacillus parabrevis	LMG-11984	17	LMG_11984GL001839, LMG_11984GL000308, LMG_11984GL	3	LMG_11984GL002001, LMG_11984GL001996,	Yes	1	1	LMG_11984GL002001-LMG_11984GL002004
Lactobacillus parabrevis	ATCC-53295	16	ATCC_53295GL000129, ATCC_53295GL000027, ATCC_5329	3	ATCC_53295GL001936, ATCC_53295GL002031	Yes	1	1	ATCC_53295GL002030-ATCC_53295GL002033
Lactobacillus hammesii	DSM-16381	18	DSM_16381GL002462, DSM_16381GL000826, DSM_16381GL	3	DSM_16381GL000002, DSM_16381GL000001,	Yes	1	1	DSM_16381GL000001-DSM_16381GL000004
Lactobacillus paucivorans	DSM-22467	23	DSM_22467GL001771, DSM_22467GL000286, DSM_22467GL	3	DSM_22467GL000284, DSM_22467GL001653,	Yes	1	1	DSM_22467GL001653-DSM_22467GL001656
Lactobacillus senmaizukei	DSM-21775	17	DSM_21775GL001681, DSM_21775GL001003, DSM_21775GL	3	DSM_21775GL001641, DSM_21775GL001140,	Yes	1	1	DSM_21775GL001640-DSM_21775GL001643
Lactobacillus brevis	DSM-20054	7	DSM_20054GL001191, DSM_20054GL001910, DSM_20054GL	1	DSM_20054GL002361	Yes	0	0	

Lactobacillus koreensis	JCM-16448	9	JCM_16448GL000085, JCM_16448GL001438, JCM_16448GL0	3	JCM_16448GL001675, JCM_16448GL000224, JC	Yes	1	1	JCM_16448GL0000222-JCM_16448GL000225
Lactobacillus zymae	DSM-19395	16	DSM_19395GL002319, DSM_19395GL000381, DSM_19395GL	1	DSM_19395GL000934	No	0	0	
Lactobacillus acidifarinae	DSM-19394	14	DSM_19394GL001854, DSM_19394GL002248, DSM_19394GL	2	DSM_19394GL001800, DSM_19394GL000905	Yes	0	0	
Lactobacillus namurensis	DSM-19117	15	DSM_19117GL000223, DSM_19117GL002162, DSM_19117GL	1	DSM_19117GL001722	No	0	0	
Lactobacillus spicheri	DSM-15429	15	DSM_15429GL000237, DSM_15429GL001364, DSM_15429GL	1	DSM_15429GL000943	No	0	0	
Lactobacillus kimchicus	JCM-15530	7	JCM_15530GL002468, JCM_15530GL000731, JCM_15530GL0	1	JCM_15530GL000783	No	0	0	
Lactobacillus similis	DSM-23365	21	DSM_23365GL001270, DSM_23365GL000121, DSM_23365GL	1	DSM_23365GL001158	Yes	0	0	
Lactobacillus odoratitofui	DSM-19909	23	DSM_19909GL000375, DSM_19909GL000334, DSM_19909GL	1	DSM_19909GL002358	Yes	0	0	
Lactobacillus collinoides	DSM-20515	10	DSM_20515GL000349, DSM_20515GL002667, DSM_20515GL	1	DSM_20515GL000897	Yes	0	0	
Lactobacillus paracollinoides	DSM-15502	15	DSM_15502GL003136, DSM_15502GL000710, DSM_15502GL	1	DSM_15502GL001533	Yes	0	0	
Lactobacillus malefermentans	DSM-5705	12	DSM_5705GL001772, DSM_5705GL000489, DSM_5705GL000	1	DSM_5705GL000241	No	0	0	
Lactobacillus parabuchneri	DSM-5707	9	DSM_5707GL002343, DSM_5707GL002311, DSM_5707GL001	1	DSM_5707GL002384	No	0	0	
Lactobacillus parabuchneri	DSM-15352	11	DSM_15352GL001529, DSM_15352GL000299, DSM_15352GL	3	DSM_15352GL002182, DSM_15352GL001528, D	Yes	1	1	DSM_15352GL001527-DSM_15352GL001531
Lactobacillus buchneri	DSM-20057	5	DSM_20057GL002290, DSM_20057GL002208, DSM_20057GL	1	DSM_20057GL002253	No	0	0	
Lactobacillus otakiensis	DSM-19908	6	DSM_19908GL000970, DSM_19908GL000708, DSM_19908GL	1	DSM_19908GL002000	Yes	0	0	
Lactobacillus kefiri	DSM-20587	3	DSM_20587GL001017, DSM_20587GL001780, DSM_20587GL	1	DSM_20587GL001982	No	0	0	
Lactobacillus parakefiri	DSM-10551	6	DSM_10551GL001097, DSM_10551GL001348, DSM_10551GL	2	DSM_10551GL000796, DSM_10551GL004542	No	0	0	
Lactobacillus sunkii	DSM-19904	4	DSM_19904GL000561, DSM_19904GL002117, DSM_19904GL	1	DSM_19904GL000603	No	0	0	
Lactobacillus rapi	DSM-19907	6	DSM_19907GL001146, DSM_19907GL002649, DSM_19907GL	1	DSM_19907GL001524	No	0	0	
Lactobacillus kisonensis	DSM-19906	6	DSM_19906GL002469, DSM_19906GL002006, DSM_19906GL	1	DSM_19906GL001744	No	0	0	
Lactobacillus diolivorans	DSM-14421	4	DSM_14421GL000349, DSM_14421GL002640, DSM_14421GL	2	DSM_14421GL002029, DSM_14421GL002101	Yes	0	0	
Lactobacillus hilgardii	DSM-20176	2	DSM_20176GL001813, DSM_20176GL000973	1	DSM_20176GL000386	No	0	0	
Lactobacillus farraginis	DSM-18382	1	DSM_18382GL000336	1	DSM_18382GL002163	Yes	0	0	
Lactobacillus parafarraginis	DSM-18390	7	DSM_18390GL001600, DSM_18390GL000527, DSM_18390GL	2	DSM_18390GL002766, DSM_18390GL001314	No	0	0	
Lactobacillus senioris	DSM-24302	3	DSM_24302GL001476, DSM_24302GL001193, DSM_24302GL	3	DSM_24302GL001196, DSM_24302GL001052, D	Yes	1	1	DSM_24302GL001193-DSM_24302GL001196
Lactobacillus ozensis	DSM-23829	0	0	1	DSM_23829GL001214	No	0	0	
Lactobacillus kunkeei	DSM-12361	6	DSM_12361GL001115, DSM_12361GL001310, DSM_12361GL	1	DSM_12361GL001048	No	0	0	
Lactobacillus florum	DSM-22689	6	DSM_22689GL000154, DSM_22689GL000095, DSM_22689GL	1	DSM_22689GL000108	No	0	0	
Lactobacillus lindneri	DSM-20690	6	DSM_20690GL001120, DSM_20690GL001342, DSM_20690GL	1	DSM_20690GL000658	No	0	0	
Lactobacillus sanfranciscensis	DSM-20451	4	DSM_20451GL000977, DSM_20451GL000262, DSM_20451GL	1	DSM_20451GL000270	No	0	0	
Lactobacillus fructivorans	ATCC-27394	9	ATCC_27394GL000693, ATCC_27394GL000680, ATCC_2739	1	ATCC_27394GL000683	Yes	0	0	
Lactobacillus homohiochii	DSM-20571	9	DSM_20571GL000572, DSM_20571GL000664, DSM_20571GL	1	DSM_20571GL000680	Yes	0	0	
Lactobacillus fructivorans	DSM-20350	8	DSM_20350GL000774, DSM_20350GL000329, DSM_20350GL	1	DSM_20350GL001071	Yes	0	0	
Lactobacillus fructivorans	DSM-20203	8	DSM_20203GL001276, DSM_20203GL000494, DSM_20203GL	1	DSM_20203GL000504	Yes	0	0	
Pediococcus argentinicus	DSM-23026	4	DSM_23026GL001175, DSM_23026GL000894, DSM_23026GL	1	DSM_23026GL000528	No	0	0	
Pediococcus claussenii	DSM-14800	3	DSM_14800GL000637, DSM_14800GL001642, DSM_14800GL	2	DSM_14800GL001036, DSM_14800GL001113	Yes	1	1	DSM_14800GL001113-DSM_14800GL001115
Pediococcus pentosaceus	DSM-20336	3	DSM_20336GL000909, DSM_20336GL000913, DSM_20336GL	1	DSM_20336GL000314	No	0	0	
Pediococcus stilesii	DSM-18001	0	0	1	DSM_18001GL000326	No	0	0	
Pediococcus lolii	DSM-19927	3	DSM_19927GL001047, DSM_19927GL001900, DSM_19927GL	1	DSM_19927GL001675	Yes	0	0	
Pediococcus acidilactici	AS1-2696	3	AS1.2696GL001049, AS1.2696GL001783, AS1.2696GL000415	1	AS1.2696GL000951	No	0	0	
Pediococcus ethanolidurans	DSM-22301	9	DSM_22301GL000128, DSM_22301GL000049, DSM_22301GL	4	DSM_22301GL001131, DSM_22301GL000126, D	Yes	1	1	DSM_22301GL000125-DSM_22301GL000128
Pediococcus cellicola	DSM-17757	7	DSM_17757GL000421, DSM_17757GL001525, DSM_17757GL	2	DSM_17757GL001526, DSM_17757GL001909	No	0	0	
Pediococcus damnosus	DSM-20331	10	DSM_20331GL001090, DSM_20331GL001685, DSM_20331GL	3	DSM_20331GL001092, DSM_20331GL001093, D	Yes	1	1	DSM_20331GL001090-DSM_20331GL001093
Pediococcus inopinatus	DSM-20285	10	DSM_20285GL000864, DSM_20285GL000210, DSM_20285GL	4	DSM_20285GL001776, DSM_20285GL000520, D	Yes	1	1	DSM_20285GL000546-DSM_20285GL000549
Pediococcus parvulus	DSM-20332	10	DSM_20332GL002774, DSM_20332GL003420, DSM_20332GL	6	DSM_20332GL003316, DSM_20332GL001496, D	Yes	1	2	DSM_20332GL003313-DSM_20332GL003316, DSM_20332GL003417-D
Carnobacterium maltaromaticum	DSM-20730	22	DSM_20730GL003263, DSM_20730GL000714, DSM_20730GL	4	DSM_20730GL003099, DSM_20730GL003366, D	Yes	0	0	
Carnobacterium maltaromaticum	DSM-20342	27	DSM_20342GL002886, DSM_20342GL001571, DSM_20342GL	7	DSM_20342GL001625, DSM_20342GL002378, D	Yes	1	1	DSM_20342GL002941-DSM_20342GL002944
Carnobacterium maltaromaticum	DSM-20722	22	DSM_20722GL002004, DSM_20722GL001429, DSM_20722GL	7	DSM_20722GL000754, DSM_20722GL001730, D	Yes	1	1	DSM_20722GL000754-DSM_20722GL000757
Carnobacterium divergens	DSM-20623	18	DSM_20623GL001163, DSM_20623GL000792, DSM_20623GL	4	DSM_20623GL001820, DSM_20623GL001147, D	No	0	0	
Lactococcus lactis	LMG-7760	7	LMG_7760GL001946, LMG_7760GL001608, LMG_7760GL00	2	LMG_7760GL001539, LMG_7760GL001947	Yes	1	1	LMG_7760GL0001946-LMG_7760GL001949
Atopobium minutum	DSM-20586	9	DSM_20586GL001354, DSM_20586GL000632, DSM_20586GL	1	DSM_20586GL001167	No	0	0	
Olsenella uli	DSM-7084	5	DSM_7084GL001434, DSM_7084GL000839, DSM_7084GL001	2	DSM_7084GL001746, DSM_7084GL001525	Yes	1	1	DSM_7084GL001746-DSM_7084GL001749
Atopobium rimae	DSM-7090	0	0	1	DSM_7090GL000687	No	0	0	
**Total**		**1628**		**357**			**51**	**67**	

Footnote: The gene sequences for all locus tags listed above are provided as supplementary datasets 3,4 and

***** Cells colored in light gray indicate strain harboring at least 1 pilus gene cluster with a similar gene order a *L. rhamnosus* strain GG.

Table S6. Distribution and abundance of cell envelope proteins and associated anchoring domains and motifs

			Cell anchor type		
**Species name**	**StrainID**	**CEPs**	**LPxTG**	**SLAP**	**T**
Kandleria vitulina	DSM-20405	0	0	0	0
Lactobacillus kimchiensis	DSM-24716	0	0	0	0
Lactobacillus mindensis	DSM-14500	0	0	0	0
Lactobacillus nantensis	DSM-16982	0	0	0	0
Lactobacillus crustorum	LMG-23699	0	0	0	0
Lactobacillus crustorum	JCM-15951	0	0	0	0
Lactobacillus futsaii	JCM-17355	0	0	0	0
Lactobacillus farciminis	DSM-20184	0	0	0	0
Lactobacillus alimentarius	DSM-20249	0	0	0	0
Lactobacillus paralimentarius	DSM-19674	0	0	0	0
Lactobacillus paralimentarius	DSM-13961	0	0	0	0
Lactobacillus paralimentarius	DSM-13238	0	0	0	0
Lactobacillus tucceti	DSM-20183	0	0	0	0
Lactobacillus nodensis	DSM-19682	0	0	0	0
Lactobacillus versmoldensis	DSM-14857	0	0	0	0
Lactobacillus floricola	DSM-23037	0	0	0	0
Lactobacillus acidophilus	DSM-20079	1	0	1	0
Lactobacillus amylovorus	DSM-20531	0	0	0	0
Lactobacillus amylovorus	DSM-16698	0	0	0	0
Lactobacillus kitasatonis	DSM-16761	0	0	0	0
Lactobacillus kefiranofaciens kefiranofaciens	DSM-5016	2	0	2	0
Lactobacillus kefiranofaciens kefirgranum	DSM-10550	0	0	0	0
Lactobacillus ultunensis	DSM-16047	1	0	1	0
Lactobacillus helveticus	LMG-22464	1	0	1	0
Lactobacillus helveticus	CGMCC-1.1877	1	0	1	0
Lactobacillus gallinarum	DSM-10532	1	0	1	0
Lactobacillus crispatus	DSM-20584	0	0	0	0
Lactobacillus acetotolerans	DSM-20749	0	0	0	0
Lactobacillus intestinalis	DSM-6629	1	0	1	0
Lactobacillus hamsteri	DSM-5661	0	0	0	0
Lactobacillus amylolyticus	DSM-11664	0	0	0	0
Lactobacillus kalixensis	DSM-16043	1	0	1	0
Lactobacillus gigeriorum	DSM-23908	1	0	1	0
Lactobacillus pasteurii	DSM-23907	0	0	0	0
Lactobacillus delbrueckii jakobsenii	DSM-26046	1	0	1	0
Lactobacillus delbrueckii lactis	DSM-20072	1	0	1	0
Lactobacillus delbrueckii bulgaricus	DSM-20081	1	0	1	0
Lactobacillus delbrueckii delbrueckii	DSM-20074	1	0	1	0
Lactobacillus delbrueckii indicus	DSM-15996	1	0	1	0
Lactobacillus equicursoris	DSM-19284	1	0	1	0
Lactobacillus jensenii	DSM-20557	0	0	0	0
Lactobacillus psittaci	DSM-15354	0	0	0	0
Lactobacillus hominis	DSM-23910	1	0	1	0
Lactobacillus taiwanensis	DSM-21401	1	1	0	0
Lactobacillus johnsonii	ATCC-33200	0	0	0	0
Lactobacillus gasseri	ATCC-33323	0	0	0	0
Lactobacillus iners	DSM-13335	0	0	0	0
Lactobacillus amylotrophicus	DSM-20534	0	0	0	0
Lactobacillus amylophilus	DSM-20533	0	0	0	0
Lactobacillus dextrinicus	DSM-20335	0	0	0	0
Lactobacillus concavus	DSM-17758	1	1	0	0
Lactobacillus composti	DSM-18527	0	0	0	0
Lactobacillus harbinensis	DSM-16991	0	0	0	0
Lactobacillus perolens	DSM-12744	1	0	0	1
Lactobacillus camelliae	DSM-22697	2	**2**	0	0
Lactobacillus nasuensis	JCM-17158	1	1	0	0
Lactobacillus manihotivorans	DSM-13343	1	1	0	0
Lactobacillus paracasei paracasei	DSM-5622	2	**2**	0	0
Lactobacillus casei	DSM-20011	1	1	0	0
Lactobacillus paracasei tolerans	DSM-20258	0	0	0	0
Lactobacillus zeae	DSM-20178	2	2	0	0
Lactobacillus rhamnosus	DSM-20021	2	**2**	0	0
Lactobacillus saniviri	DSM-24301	0	0	0	0
Lactobacillus brantae	DSM-23927	0	0	0	0
Lactobacillus thailandensis	DSM-22698	0	0	0	0
Lactobacillus pantheris	DSM-15945	1	1	0	0
Lactobacillus sharpeae	DSM-20505	0	0	0	0
Lactobacillus selangorensis	DSM-13344	0	0	0	0
Lactobacillus selangorensis	ATCC-BAA-66	0	0	0	0
Lactobacillus graminis	DSM-20719	0	0	0	0
Lactobacillus curvatus	DSM-20019	0	0	0	0
Lactobacillus sakei sakei	DSM-20017	0	0	0	0
Lactobacillus sakei carnosus	DSM-15831	0	0	0	0
Lactobacillus fuchuensis	DSM-14340	0	0	0	0
Lactobacillus rennini	DSM-20253	0	0	0	0
Lactobacillus coryniformis torquens	DSM-20004	0	0	0	0
Lactobacillus coryniformis coryniformis	DSM-20001	0	0	0	0
Lactobacillus bifermentans	DSM-20003	0	0	0	0
Lactobacillus ceti	DSM-22408	0	0	0	0
Lactobacillus saerimneri	DSM-16049	0	0	0	0
Lactobacillus animalis	DSM-20602	1	1	0	0
Lactobacillus murinus	DSM-20452	1	1	0	0
Lactobacillus apodemi	DSM-16634	1	1	0	0
Lactobacillus ruminis	DSM-20403	1	1	0	0
Lactobacillus agilis	DSM-20509	0	0	0	0
Lactobacillus equi	DSM-15833	1	1	0	0
Lactobacillus salivarius	DSM-20555	1	1	0	0
Lactobacillus hayakitensis	DSM-18933	0	0	0	0
Lactobacillus pobuzihii	NBRC-103219	0	0	0	0

Lactobacillus pobuzihii.Chen	KCTC-13174	0	0	0	0
Lactobacillus acidipiscis	DSM-15836	0	0	0	0
Lactobacillus acidipiscis	DSM-15353	0	0	0	0
Lactobacillus aviarius aviarius	DSM-20655	0	0	0	0
Lactobacillus aviarius araffinosus	DSM-20653	0	0	0	0
Lactobacillus sucicola	DSM-21376	0	0	0	0
Lactobacillus aquaticus	DSM-21051	0	0	0	0
Lactobacillus uvarum	DSM-19971	0	0	0	0
Lactobacillus capillatus	DSM-19910	0	0	0	0
Lactobacillus cacaonum	DSM-21116	0	0	0	0
Lactobacillus mali	DSM-20444	0	0	0	0
Lactobacillus mali	ATCC-27304	0	0	0	0
Lactobacillus hordei	DSM-19519	0	0	0	0
Lactobacillus oeni	DSM-19972	0	0	0	0
Lactobacillus satsumensis	DSM-16230	0	0	0	0
Lactobacillus vini	DSM-20605	0	0	0	0
Lactobacillus ghanensis	DSM-18630	0	0	0	0
Lactobacillus nagelii	DSM-13675	0	0	0	0
Lactobacillus algidus	DSM-15638	0	0	0	0
Lactobacillus fabifermentans	DSM-21115	0	0	0	0
Lactobacillus xiangfangensis	LMG-26013	0	0	0	0
Lactobacillus pentosus	DSM-20314	0	0	0	0
Lactobacillus plantarum argentoratensis	DSM-16365	0	0	0	0
Lactobacillus plantarum	DSM-13273	0	0	0	0
Lactobacillus plantarum plantarum	CGMCC-1.2437	0	0	0	0
Lactobacillus paraplantarum	DSM-10667	0	0	0	0
Lactobacillus siliginis	DSM-22696	0	0	0	0
Lactobacillus rossiae	DSM-15814	0	0	0	0
Weissella viridescens	DSM-20410	0	0	0	0
Weissella minor	DSM-20014	0	0	0	0
Weissella halotolerans	DSM-20190	0	0	0	0
Weissella confusa	DSM-20196	0	0	0	0
Weissella kandleri	DSM-20593	0	0	0	0
Oenococcus oeni	ATCC-BAA-1163	0	0	0	0
Oenococcus kitaharae	DSM-17330	0	0	0	0
Leuconostoc fallax	KCTC-3537	0	0	0	0
Leuconostoc pseudomesenteroides	4882-	1	0	0	1
Leuconostoc mesenteroides	ATCC-8293	0	0	0	0
Leuconostoc mesenteroides cremoris	ATCC-19254	0	0	0	0
Leuconostoc carnosum	JB16-	0	0	0	0
Leuconostoc argentinum	KCTC-3773	0	0	0	0
Leuconostoc citreum	KM20-	0	0	0	0
Leuconostoc gelidum	KCTC-3527	0	0	0	0
Leuconostoc gasicomitatum	LMG-18811	0	0	0	0
Leuconostoc kimchii	IMSNU-11154	0	0	0	0
Fructobacillus fructosus	DSM-20349	0	0	0	0
Lactobacillus pontis	DSM-8475	0	0	0	0
Lactobacillus panis	DSM-6035	0	0	0	0
Lactobacillus oris	DSM-4864	0	0	0	0
Lactobacillus antri	DSM-16041	0	0	0	0
Lactobacillus reuteri	DSM-20016	0	0	0	0
Lactobacillus vaginalis	DSM-5837	0	0	0	0
Lactobacillus frumenti	DSM-13145	0	0	0	0
Lactobacillus fermentum	DSM-20055	0	0	0	0
Lactobacillus equigenerosi	DSM-18793	0	0	0	0
Lactobacillus gastricus	DSM-16045	0	0	0	0
Lactobacillus ingluviei	DSM-15946	0	0	0	0
Lactobacillus ingluviei	DSM-14792	0	0	0	0
Lactobacillus secaliphilus	DSM-17896	0	0	0	0
Lactobacillus coleohominis	DSM-14060	0	0	0	0
Lactobacillus mucosae	DSM-13345	0	0	0	0
Lactobacillus oligofermentans	DSM-15707	0	0	0	0
Lactobacillus hokkaidonensis	DSM-26202	0	0	0	0
Lactobacillus suebicus	DSM-5007	0	0	0	0
Lactobacillus vaccinostercus	DSM-20634	0	0	0	0
Lactobacillus parabrevis	LMG-11984	0	0	0	0
Lactobacillus parabrevis	ATCC-53295	0	0	0	0
Lactobacillus hammesii	DSM-16381	0	0	0	0
Lactobacillus paucivorans	DSM-22467	0	0	0	0
Lactobacillus senmaizukei	DSM-21775	0	0	0	0
Lactobacillus brevis	DSM-20054	0	0	0	0
Lactobacillus koreensis	JCM-16448	0	0	0	0
Lactobacillus zymae	DSM-19395	0	0	0	0
Lactobacillus acidifarinae	DSM-19394	0	0	0	0
Lactobacillus namurensis	DSM-19117	0	0	0	0
Lactobacillus spicheri	DSM-15429	0	0	0	0
Lactobacillus kimchicus	JCM-15530	0	0	0	0
Lactobacillus similis	DSM-23365	0	0	0	0
Lactobacillus odoratitofui	DSM-19909	0	0	0	0
Lactobacillus collinoides	DSM-20515	1	1	0	0
Lactobacillus paracollinoides	DSM-15502	0	0	0	0
Lactobacillus malefermentans	DSM-5705	0	0	0	0
Lactobacillus parabuchneri	DSM-5707	1	0	0	1
Lactobacillus parabuchneri	DSM-15352	1	0	0	1
Lactobacillus buchneri	DSM-20057	1	0	0	1
Lactobacillus otakiensis	DSM-19908	1	0	0	1
Lactobacillus kefiri	DSM-20587	1	0	0	1
Lactobacillus parakefiri	DSM-10551	2	0	0	2
Lactobacillus sunkii	DSM-19904	1	0	0	1
Lactobacillus rapi	DSM-19907	0	0	0	0
Lactobacillus kisonensis	DSM-19906	0	0	0	0
Lactobacillus diolivorans	DSM-14421	2	1	0	1
Lactobacillus hilgardii	DSM-20176	0	0	0	0
Lactobacillus farraginis	DSM-18382	1	0	0	1

Lactobacillus parafarraginis	DSM-18390	1	0	0	1
Lactobacillus senioris	DSM-24302	0	0	0	0
Lactobacillus ozensis	DSM-23829	0	0	0	0
Lactobacillus kunkeei	DSM-12361	0	0	0	0
Lactobacillus florum	DSM-22689	0	0	0	0
Lactobacillus lindneri	DSM-20690	0	0	0	0
Lactobacillus sanfranciscensis	DSM-20451	0	0	0	0
Lactobacillus fructivorans	ATCC-27394	0	0	0	0
Lactobacillus homohiochii	DSM-20571	0	0	0	0
Lactobacillus fructivorans	DSM-20350	0	0	0	0
Lactobacillus fructivorans	DSM-20203	0	0	0	0
Pediococcus argentinicus	DSM-23026	0	0	0	0
Pediococcus claussenii	DSM-14800	0	0	0	0
Pediococcus pentosaceus	DSM-20336	0	0	0	0
Pediococcus stilesii	DSM-18001	0	0	0	0
Pediococcus lolii	DSM-19927	0	0	0	0
Pediococcus acidilactici	AS1-2696	0	0	0	0
Pediococcus ethanolidurans	DSM-22301	1	1	0	0
Pediococcus cellicola	DSM-17757	1	1	0	0
Pediococcus damnosus	DSM-20331	0	0	0	0
Pediococcus inopinatus	DSM-20285	0	0	0	0
Pediococcus parvulus	DSM-20332	0	0	0	0
Carnobacterium maltaromaticum	DSM-20730	1	1	0	0
Carnobacterium maltaromaticum	DSM-20342	1	1	0	0
Carnobacterium maltaromaticum	DSM-20722	1	1	0	0
Carnobacterium divergens	DSM-20623	1	1	0	0
Lactococcus lactis	LMG-7760	0	0	0	0
Atopobium minutum	DSM-20586	2	**2**	0	0
Olsenella uli	DSM-7084	0	0	0	0
Atopobium rimae	DSM-7090	0	0	0	0
**Total**		**60**	**30**	**17**	**13**

Table S7. CRISPR occurrence and diversity.

Species Name	StrainID	CRISPR Type	CRISPR Repeat Sequence	DR Length	No. of Spacers	Cas1	Cas3	Cas9	Cas10
Kandleria vitulina	DSM-20405	II	GTTTTAGAGTTGTGTTATTTTGAACAGATACAAAAC	36	43	Y		Y	
Lactobacillus kimchiensis	DSM-24716	Undefined	GTGTTCCCCATATACATGGGGATGATTCT	29	3				
Lactobacillus mindensis A	DSM-14500	II	GTTTTAGAAGTAAGTCATCTCAATTACTAAGAACC	35	25	Y		Y	
Lactobacillus mindensis B	DSM-14500	Undefined	GTGCTCCCCATAAACATGGGGATGATTCT	29	3				
Lactobacillus nantensis	DSM-16982	II	GTTTTTGTACCTTCAAAGATTTAGAAACAGTAAAAC	36	22	Y		Y	
Lactobacillus crustorum	LMG-23699	N/A	None found						
Lactobacillus crustorum	JCM-15951	N/A	None found						
Lactobacillus futsaii	JCM-17355	II	GTTTTTGTACCTTAAAGAATCTAGAAATAGTAAAAC	36	19	Y		Y	
Lactobacillus farciminis A	DSM-20184	Undefined	GTTTTTGTACCTTAAAGAATCTAGAAATAGTAAAAC	36	4				
Lactobacillus farciminis B	DSM-20184	II	GTTTTAGAAGTATGTCTTTCTATTTACTTAAGAACC	36	11	Y		Y	
Lactobacillus alimentarius	DSM-20249	N/A	None found						
Lactobacillus paralimentarius	DSM-19674	N/A	None found						
Lactobacillus paralimentarius	DSM-13961	N/A	None found						
Lactobacillus paralimentarius A	DSM-13238	I	GTACTCCCCATGTATATGGGGATGATTCC	29	11	Y	Y		
Lactobacillus paralimentarius B	DSM-13238	Undefined	GTGCTCCCCATATACATGGGGATGATTCT	29	15				
Lactobacillus tucceti	DSM-20183	II	GTTTTTGTACTCTTAAGGATTTAGTAATAGTAAAAC	36	15	Y		Y	
Lactobacillus nodensis A	DSM-19682	II	GTTTTAGAAGTACGTCATTTCATGTAGTTAAGAACC	36	9	Y		Y	
Lactobacillus nodensis B	DSM-19682	II	GTTTTAGTACTCTCAAGAATTTAGTAACAGTAAAAC	36	9	Y		Y	
Lactobacillus versmoldensis	DSM-14857	II	GTTTTAGATCTAAGTCATCTCAATTACTTAAGAACC	36	2	Y		Y	
Lactobacillus floricola A	DSM-23037	II	GTTTTAGAAGTATGTCAAATCAATAAGGTTAATACC	36	5	Y		Y	
Lactobacillus floricola B	DSM-23037	II	GTTTCAGAAGTATGTCATTTGAATAATGTTAGGACT	36	5	Y		Y	
Lactobacillus floricola C	DSM-23037	Undefined	CTTTTTCTCGCATACGCGAGAGTGATCC	28	46				
Lactobacillus acidophilus	DSM-20079	Undefined	ATTTTCTCCACGTATGTGGAGGTGATCC	28	31				
Lactobacillus amylovorus	DSM-20531	N/A	None found						
Lactobacillus amylovorus	DSM-16698	I	GTTTTTATTTAACTTAAGAGAAATGTAAAT	30	53	Y	Y		
Lactobacillus kitasatonis	DSM-16761	N/A	None found						
Lactobacillus kefiranofaciens kefiranofaciens A	DSM-5016	Undefined	GTGTTCTCCACGTATGTGGAGGT	23	5				
Lactobacillus kefiranofaciens kefiranofaciens B	DSM-5016	Undefined	GTGTTCTCCACGTATGTGGAGGTGATCCT	29	4				
Lactobacillus kefiranofaciens kefirgranum	DSM-10550	I	GTGTTCTCCACGTATGTGGAGGTGATCC	28	61	Y	Y		
Lactobacillus ultunensis	DSM-16047	N/A	None found						
Lactobacillus helveticus	LMG-22464	I	GTATTCTCCACGTATGTGGAGGTGATCC	28	26	Y	Y		
Lactobacillus helveticus	CGMCC-1.1877	I	GTTTTTATTTAACTTAAGAGAAATGTAAAG	30	41	Y	Y		
Lactobacillus gallinarum	DSM-10532	N/A	None found						
Lactobacillus crispatus	DSM-20584	Undefined	GTATTCTCCACGTGTGTGGAGGTGATCC	28	3				
Lactobacillus acetotolerans A	DSM-20749	I	GTATTCTCCACGTATGTGGAGGTGATCCT	29	45	Y	Y		
Lactobacillus acetotolerans B	DSM-20749	Undefined	GTTTTAGATGATTGTTAGATCAATGAGGTTTAGACC	36	9				
Lactobacillus intestinalis A	DSM-6629	I	GTATTCCCCACATGGGTGGGGGTGATCC	28	7	Y	Y		
Lactobacillus intestinalis B	DSM-6629	I	GTATTCCCCACACACGTGGGGGTGATCC	28	13	Y	Y		
Lactobacillus intestinalis C	DSM-6629	I	GTTTTTATTTAACTTAAGAGGAATGTAAAT	30	9	Y*	Y*		
Lactobacillus intestinalis D	DSM-6629	Undefined	GGATCACCTCCACATACGTGGAGAACAC	28	11				
Lactobacillus hamsteri	DSM-5661	I	GTATTCTCCACGCATGTGGAGGTGATCC	28	43	Y	Y		
Lactobacillus amylolyticus	DSM-11664	N/A	None found						
Lactobacillus kalixensis	DSM-16043	II	GTTTTAGATCTGATGTACCTAGTTAAGATGTAAAAC	36	4	Y*			
Lactobacillus gigeriorum	DSM-23908	N/A	None found						
Lactobacillus pasteurii	DSM-23907	I	GTGGTCCCCACGTAAGTGGGGGTGATCC	28	9	Y	Y		
Lactobacillus delbrueckii jakobsenii	DSM-26046	II	GTTTTAGAAGGTTGTCTATTCAATAAGGTTTAACCC	36	11	Y		Y	
Lactobacillus delbrueckii lactis	DSM-20072	Undefined	None found			Y	Y		
Lactobacillus delbrueckii bulgaricus	DSM-20081	I	GTATTCCCCACGCAAGTGGGGGTGATCC	28	40	Y	Y		
Lactobacillus delbrueckii delbrueckii	DSM-20074	I	GTATTCCCCACGCAAGTGGGGGTGATCC	28	40	Y	Y		
Lactobacillus delbrueckii indicus	DSM-15996	I	GTATTCCCCACGCAAGTGGGGGTGATCC	28	39	Y	Y		
Lactobacillus equicursoris	DSM-19284	I	GTATTCCCCTCGTATGAGGGGGTGATCC	28	21	Y	Y		
Lactobacillus jensenii	DSM-20557	Undefined	None found			Y		Y	
Lactobacillus psittaci	DSM-15354	II	GTTTTAGAAGGTTGTTAAATCAGTAAGTTGAAAAAC	36	42	Y		Y	
Lactobacillus hominis	DSM-23910	II	GTTTTAGTTAGTTGTTAGATCAATAAGGTTTAGATC	36	9	Y		Y	
Lactobacillus taiwanensis	DSM-21401	N/A	None found						
Lactobacillus johnsonii	ATCC-33200	N/A	None found						
Lactobacillus gasseri	ATCC-33323	N/A	None found						
Lactobacillus iners	DSM-13335	N/A	None found						
Lactobacillus amylotrophicus	DSM-20534	Undefined	GTTTTCCCCGCACAGGCGGGGGTGATCC	28	52				
Lactobacillus amylophilus	DSM-20533	I	GTTTTCCCCGCACAGGCGGGGGTGATCC	28	52	Y	Y		
Lactobacillus dextrinicus	DSM-20335	I	GTTTTTATCTAAAGAGTATTGAATGTAAAT	30	100	Y	Y		
Lactobacillus concavus	DSM-17758	II	GTTTTAGAAGAGTGTCATTTCAATAAGGTTAAGATC	36	68	Y		Y*	
Lactobacillus composti A	DSM-18527	II	GTTCTGGAAGTATGTCAGATCAATGGATTCAAGAGC	36	10	Y		Y	
Lactobacillus composti B	DSM-18527	Undefined	CGCAACTCCTGTAGTTGCGTGAATTGAAAT	30	5				
Lactobacillus harbinensis	DSM-16991	II	GTCCCATTTTAGCCGATTCTGGAAGGATCCAATAGC	36	60	Y*		Y	
Lactobacillus perolens A	DSM-12744	Undefined	TTAGGGGTGCGTGAATTGAAAG	22	9				
Lactobacillus perolens B	DSM-12744	I	GTCGCATCCCTCGGGGTGCGTGAATTGAAAG	31	9	Y	Y		
Lactobacillus camelliae	DSM-22697	N/A	None found						
Lactobacillus nasuensis	JCM-17158	N/A	None found						
Lactobacillus manihotivorans	DSM-13343	N/A	None found						
Lactobacillus paracasei paracasei	DSM-5622	N/A	None found						
Lactobacillus casei	DSM-20011	I	GTTTTCCCCGCACATGCGGGGGTGATCC	28	20	Y	Y*		
Lactobacillus paracasei tolerans	DSM-20258	N/A	None found						
Lactobacillus zeae	DSM-20178	I	GTCGCAGTCTACGTGACTGCGTGAATTGAAAT	32	86	Y	Y		
Lactobacillus rhamnosus	DSM-20021	N/A	None found						
Lactobacillus saniviri	DSM-24301	II	GTTTTAGTTGGATGTCAGATCAAATAGGTTAAGCAC	36	58	Y		Y	
Lactobacillus brantae	DSM-23927	I	GTATTCCCCGTGCATACGGGGGTGATCC	28	45	Y	Y		
Lactobacillus thailandensis	DSM-22698	I	GTGTTCCCCGCAGGTGCGGGGGTAATCC	28	73	Y	Y		
Lactobacillus pantheris	DSM-15945	N/A	None found						
Lactobacillus sharpeae	DSM-20505	N/A	None found						
Lactobacillus selangorensis	DSM-13344	I	GTTTTTATTTAAACAATATGGAATGTAAAT	30	33	Y	Y		
Lactobacillus selangorensis A	ATCC-BAA-66	I	GTTTTTATTTAAACAATATGGAATGTAAAT	30	37	Y	Y		
Lactobacillus selangorensis B	ATCC-BAA-66	Undefined	CTTTTTATTTAAACAATGTGGAATGTAAAT	30	8				
Lactobacillus graminis	DSM-20719	II	GTTTTAGAAGAGTATCAAATCAATGAGTAGTTCAAC	36	35	Y		Y	
Lactobacillus curvatus	DSM-20019	Undefined	GTTTTAGAAGAGTATCAAATCAATGAGTAGTTCAAC	36	6				
Lactobacillus sakei sakei	DSM-20017	N/A	None found						
Lactobacillus sakei carnosus	DSM-15831	II	GTTTTAGAAGAGTATCAAATCAATGAGTAGTTCAAC	36	21	Y		Y	
Lactobacillus fuchuensis	DSM-14340	II	GTTTTAGAAGAGTATCAAATCAATGAGTTCATCAAC	36	27	Y		Y	
Lactobacillus rennini A	DSM-20253	I	GTCGCACTCTATATGGGTGCGTGAATTGAAAT	32	24	Y	Y		
Lactobacillus rennini B	DSM-20253	II	GTTTTAGAAGAGTATCAAATCAATGAGTTTATCAAC	36	12	Y		Y	
Lactobacillus coryniformis torquens	DSM-20004	II	GCTATTGATTCCTTCAGTTTTCAGCTAAAATAGATC	36	5	Y		Y	
Lactobacillus coryniformis coryniformis	DSM-20001	II	GTTTTAGAAGAGTGTTAAATCAATGAGTTTAGAACC	36	30	Y		Y	
Lactobacillus bifermentans	DSM-20003	I	GTATTCCCCGCACCAGCGGGGGTGATCC	28	118	Y	Y		
Lactobacillus ceti	DSM-22408	II	GTTTAAGACCTATCGAGAACAACACACTTCTCAAAC	36	16	Y		Y	
Lactobacillus saerimneri	DSM-16049	II	GTTTTTGTACTCTGAAGAACTTAGTAATGGAATAAC	36	8	Y*		Y*	
Lactobacillus animalis	DSM-20602	II	GTTTTAGAGCTATGTTGTTTTGTATGACTCCAAAAC	36	51	Y		Y	
Lactobacillus murinus	DSM-20452	N/A	None found						
Lactobacillus apodemi	DSM-16634	II	GTTTTAGAGCTATGTAGTTTTGTATGACTCCAAAAC	36	13	Y		Y	
Lactobacillus ruminis A	DSM-20403	III	GTTTTCGTCTCCTTCACTCGGAGATAGGTAATTATC	36	13	Y			Y
Lactobacillus ruminis B	DSM-20403	I	ATTTCAACTCACGCCCCCTATACAGAGGGCGAC	33	29	Y	Y		
Lactobacillus agilis A	DSM-20509	I	CTTCTCCCCACACTAGTGGGGGTAATCC	28	35	Y	Y		
Lactobacillus agilis B	DSM-20509	II	GTCTCAGAAGTATGTTAAATCAATGATGTTTAGTAC	36	32	Y		Y	
Lactobacillus agilis C	DSM-20509	Undefined	GTTTACATTCCTCTTAAGTTAAATAGATTC	30	4				
Lactobacillus equi A	DSM-15833	I	GTGTTCCCTACGTATGTAGGGGTAATCC	28	13	Y*			
Lactobacillus equi B	DSM-15833	I	GTGTTCCCCGTGTGTACGGGGGTGATCC	28	34	Y	Y		
Lactobacillus equi C	DSM-15833	Undefined	GTATTCCCTACGTATGTAGGGGTG	24	14				
Lactobacillus salivarius	DSM-20555	III	GTTTTCGTCTCCTTCATTCGGAGATATGTTCTTATT	36	11	Y			Y
Lactobacillus hayakitensis	DSM-18933	N/A	None found						
Lactobacillus pobuzihii	NBRC-103219	N/A	None found						
Lactobacillus pobuzihii Chen	KCTC-13174	N/A	None found						
Lactobacillus acidipiscis	DSM-15836	N/A	None found						
Lactobacillus acidipiscis A	DSM-15353	I	GTATTCCCCACGCATGTGGGGGTGATCC	28	22	Y	Y		
Lactobacillus acidipiscis B	DSM-15353	III	GTTCTCGTCCCCTATTACCGGGGATCATCTAATACC	36	39	Y			Y
Lactobacillus aviarius aviarius	DSM-20655	N/A	None found						
Lactobacillus aviarius araffinosus	DSM-20653	N/A	None found						
Lactobacillus sucicola A	DSM-21376	I	GTATTCCCCGCGTATGCGGGGGTGATCC	28	16	Y	Y		
Lactobacillus sucicola B	DSM-21376	I	GTATTTATTTAACTTAAGAGGAATGTAAAT	30	41	Y	Y		
Lactobacillus aquaticus	DSM-21051	I	GTATTCCCCGCGCATGCGGGGGTGATCC	28	26	Y	Y		
Lactobacillus uvarum	DSM-19971	Undefined	GTTTTAGAAGAGTGTTAAATCAATGAGTTTAGAACC	36	6				
Lactobacillus capillatus	DSM-19910	I	GTATTCCCCGCGCATGCGGGGGTGATCC	28	44	Y	Y		
Lactobacillus cacaonum	DSM-21116	II	GTTTTTGTACTCTCAACATTTTCCTATCAGTAAAAC	36	36	Y		Y	
Lactobacillus mali	DSM-20444	N/A	None found						
Lactobacillus mali	ATCC-27304	II	GTTTTTGCACTCTCAACATTTTCCTATCAATAAAAC	36	21	Y		Y	
Lactobacillus hordei	DSM-19519	II	GTTTTTGTACTCTCAACATTTTCCTATCAGTAAAAC	36	26	Y		Y	
Lactobacillus oeni	DSM-19972	I	GTATTCCCCACGTATGTGGGGGTGATCC	28	26	Y	Y		
Lactobacillus satsumensis	DSM-16230	N/A	None found						
Lactobacillus vini	DSM-20605	Undefined	GTTTTTGTACTCTTAAGGATTCAGTAACGGTAAAAC	36	23	Y*			
Lactobacillus ghanensis	DSM-18630	Undefined	GATTTTATTTAACTTAAGAGGAATGTAAAT	30	3				
Lactobacillus nagelii	DSM-13675	I	GATTCTATTTAACTTAAGAGGAATGTAAAG	30	19	Y	Y		

Lactobacillus algidus	DSM-15638	N/A	None found					
Lactobacillus fabifermentans	DSM-21115	N/A	None found					
Lactobacillus xiangfangensis	LMG-26013	N/A	None found					
Lactobacillus pentosus A	DSM-20314	I	CTGTTCCCCGYGTATGCGGGGGTGATCC	28	23	Y	Y	
Lactobacillus pentosus B	DSM-20314	II	GTCTTGAATAGTAGTCATATCAAACAGGTTTAGAAC	36	10	Y		Y
Lactobacillus plantarum argentoratensis A	DSM-16365	I	CTGTTCCCCGTGTATGCGGGGGTGATCC	28	12	Y	Y	
Lactobacillus plantarum argentoratensis B	DSM-16365	Undefined	CTATTCCCCGTACATACGGGGGTGATCC	28	10			
Lactobacillus plantarum	DSM-13273	N/A	None found					
Lactobacillus plantarum plantarum	CGMCC-1.2437	N/A	None found					
Lactobacillus paraplantarum	DSM-10667	N/A	None found					
Lactobacillus siliginis	DSM-22696	N/A	None found					
Lactobacillus rossiae	DSM-15814	II	GTTTTAGATGTATGTCAGATCAATAGGGTTAAGAAC	36	14	Y		Y
Weissella viridescens	DSM-20410	N/A	None found					
Weissella minor	DSM-20014	Undefined	GCATCAGCAAGTGTGTCTGAATC	23	4		Y	
Weissella halotolerans	DSM-20190	II	GCTTTAGATGTATGTCAGATTAATGGGGTTTATTCC	36	8	Y		Y
Weissella confusa	DSM-20196	N/A	None found					
Weissella kandleri	DSM-20593	II	GCTTCATATCTCTGTCAAATTAATGAGTTGTTTAGC	36	15	Y		Y
Oenococcus oeni	ATCC-BAA-1163	N/A	None found					
Oenococcus kitaharae	DSM-17330	II	GCTTCAGATGTGTGTCAGATCAATGAGGTAGAACCC	36	57	Y		Y
Leuconostoc fallax	KCTC-3537	N/A	None found					
Leuconostoc pseudomesenteroides	4882	II	GTATAAAGCCCCATTGATCTGACATACATCTGAAGC	36	6	Y		Y
Leuconostoc mesenteroides	ATCC-8293	N/A	None found					
Leuconostoc mesenteroides cremoris	ATCC-19254	N/A	None found					
Leuconostoc carnosum	JB16	N/A	None found					
Leuconostoc argentinum	KCTC-3773	N/A	None found					
Leuconostoc citreum	KM20	N/A	None found					
Leuconostoc gelidum	KCTC-3527	II	GCTTCAGATGTGTGTCAGATCAATGAGGTTTAAACC	36	29	Y		Y
Leuconostoc gasicomitatum	LMG-18811	N/A	None found					
Leuconostoc kimchii	IMSNU-11154	N/A	None found					
Fructobacillus fructosus	DSM-20349	II	GCTTTAGATGTATGTCGGATTAATGGGGTTTCTTCC	36	8	Y		Y
Lactobacillus pontis	DSM-8475	N/A	None found					
Lactobacillus panis	DSM-6035	Undefined	GTATTTATCTAATAGAAGTGGAATGTAAAT	30	30			
Lactobacillus oris	DSM-4864	I	GTATTCCCCATGTACGTGGGGGTGATCC	28	18	Y	Y	
Lactobacillus antri	DSM-16041	I	GTATTCCCCATGTGTATGGGGGTGATCC	28	10	Y	Y	
Lactobacillus reuteri	DSM-20016	N/A	None found					
Lactobacillus vaginalis	DSM-5837	N/A	None found					
Lactobacillus frumenti	DSM-13145	N/A	None found					
Lactobacillus fermentum	DSM-20055	II	GTACTCGGAACTACTGATCTGACACTCATCCAAGAC	36	6	Y		Y
Lactobacillus equigenerosi	DSM-18793	N/A	None found					
Lactobacillus gastricus	DSM-16045	II	GTTTTAGAAGGGCTTCAACTCAATGACCTCAAGACC	36	17	Y		Y
Lactobacillus ingluviei A	DSM-15946	I	TCGCACTCTGTAATGGAGTGCGTGGATTGAAAT	33	43	Y	Y	
Lactobacillus ingluviei B	DSM-15946	I	GTCGCATCCGGATCTTCGGGTGCGCGGATTGAAAT	35	6	Y*	Y*	
Lactobacillus ingluviei C	DSM-15946	Undefined	CTCTTTCCCCCGTATAGGGGGCTGAACCT	29	5			
Lactobacillus ingluviei A	DSM-14792	I	ATCGCACTCTGTAATGGAGTGCGTGGATTGAAAT	34	9	Y	Y	
Lactobacillus ingluviei B	DSM-14792	Undefined	CTCTTTCCCCCGCATAGGGGGCTGAACC	28	11			
Lactobacillus ingluviei C	DSM-14792	Undefined	GTCGCACTCTGTAATGGAGTGCGTGGATTGAAAT	34	16			
Lactobacillus secaliphilus	DSM-17896	II	GTTTTAGATGTACTTCAGATCAATGATGTTTAAAT	35	3	N		Y
Lactobacillus coleohominis	DSM-14060	N/A	None found					
Lactobacillus mucosae A	DSM-13345	I	GTATTCCCCATGTATGTGGGGGTGATCCT	29	25	Y	Y	
Lactobacillus mucosae B	DSM-13345	I	GTCGCACTCTCTTGTAGGGTGCGTGGATTGAAAT	34	20	Y	N	
Lactobacillus oligofermentans	DSM-15707	II	GTTTTAGAAGAGTATCAAATCAATGAGTTCGAGACC	36	30	Y		Y
Lactobacillus hokkaidonensis	DSM-26202	N/A	None found					
Lactobacillus suebicus	DSM-5007	I	GTCGCACTCTTCGTGAGTGCGTGAATTGAAAT	32	12	Y	Y	
Lactobacillus vaccinostercus	DSM-20634	N/A	None found					
Lactobacillus parabrevis	LMG-11984	II	GTTTTAAGTAGATGGTAAATCAATAAGGTCAAGAGC	36	30	N		
Lactobacillus parabrevis	ATCC-53295	II	GTTTTAAGTAGATGGTAAATCAATAAGGTCAAGAGC	36	30	N		
Lactobacillus hammesii A	DSM-16381	I	GTATTCCCCATGTGTATGGGGATGATCC	28	24	Y	Y	
Lactobacillus hammesii B	DSM-16381	II	GCTTTAGATTAGTGACAATTCAGTAAGGTCAAGAGC	36	47	Y		Y
Lactobacillus paucivorans	DSM-22467	I	GTACTCCCCACGTATGTGGGGATGATCC	28	16	Y	Y	
Lactobacillus senmaizukei	DSM-21775	II	GTTTTAGTTCAGTGACAAATCAATAGAGTTAAGAAC	36	39	Y		Y
Lactobacillus brevis	DSM-20054	N/A	None found					
Lactobacillus koreensis	JCM-16448	I	GTATTCCCCGTGTATACGGGGGTGATCC	28	62	Y	Y	
Lactobacillus zymae A	DSM-19395	II	GTTTTAGATGAGTGGTAAATCTAGAAGGTCAAAAGC	36	24	Y		Y
Lactobacillus zymae B	DSM-19395	Undefined	GTATTCCCCACATGTGTGGGGGTGATT	28	45			
Lactobacillus acidifarinae A	DSM-19394	I	GTATTCCCCACACGTGTGGGGGTGATTC	28	121	Y	Y	
Lactobacillus acidifarinae B	DSM-19394	Undefined	GCTTTAAGTTCCTTTCAAATTAGGGTATAAATTAAG	36	16			
Lactobacillus namurensis A	DSM-19117	I	GTATTCCCCGCGTATGCGGGGGTGATTC	28	28	Y	Y	
Lactobacillus namurensis B	DSM-19117	II	GTTTCAAATGAGTGGTAAATCTATAAGGTCAATACC	36	16	Y		Y
Lactobacillus spicheri	DSM-15429	I	GTATTCCCCACACGTGTGGGGGTGATCC	28	40	Y	Y	
Lactobacillus kimchicus	JCM-15530	I	GTACTCTCCGCACAYGCGGAGGTGATCC	28	54	Y	Y	
Lactobacillus similis A	DSM-23365	I	GTCTCGCTCTTTCGTGGAGCGAGTGAATTGAAAT	34	22	N	Y	
Lactobacillus similis B	DSM-23365	Undefined	ATCGCACTCTCTAAAGGAGTGCGTGAATTGAAAT	34	18			
Lactobacillus odoratitofui	DSM-19909	I	GTATTCTCCGCGTATGCGGAGGTGATCC	28	135	Y	Y	
Lactobacillus collinoides	DSM-20515	I	GTATTCCCCGCATGTGCGGGGGTGATCC	28	42	Y	Y	
Lactobacillus paracollinoides	DSM-15502	II	GTTTTAGATGAATATCAAATCAATGAGGTTCAAAGC	36	11	Y		Y
Lactobacillus malefermentans	DSM-5705	I	GTCGCACCTTGTATGAGGTGCGTGAATTGAAAT	33	26	Y	Y	
Lactobacillus parabuchneri	DSM-5707	Undefined	GTATTCCCCACGCATGTGGGGGTAATCC	28	10			
Lactobacillus parabuchneri	DSM-15352	II	GTTTTAGAAGGATGTTAAATCAATAAGGTTAAACCC	36	15	Y*		
Lactobacillus buchneri	DSM-20057	II	GTTTTAGAAGGATGTTAAATCAATAAGGTTAAACCC	36	9	Y		Y
Lactobacillus otakiensis A	DSM-19908	I	GTATTCCCCACGTATGTGGGGGTGATCC	28	11	Y	Y	
Lactobacillus otakiensis B	DSM-19908	II	GTTTTAGAAGGATGTTAAATCAATAAGGTTAAACCC	36	44	Y		Y
Lactobacillus kefiri A	DSM-20587	I	GTATTCCCCACGCATGTGGGGGTGATCC	28	23		Y	
Lactobacillus kefiri B	DSM-20587	Undefined	GTTTTAGAAGGATGTTAAATCAATAAGGTTAAACCC	36	10			
Lactobacillus parakefiri A	DSM-10551	I	GTATTCCCCACGCATGTGGGGGTGATCC	28	13	Y	Y	
Lactobacillus parakefiri B	DSM-10551	II	GTTTTAGAAGGATGTTAAATCAATAAGGTTAAACCC	36	34	Y		Y
Lactobacillus sunkii	DSM-19904	N/A	None found					
Lactobacillus rapi	DSM-19907	I	GTATTCCCCACGTGTGTGGGGGTGATCC	28	37	Y	Y	
Lactobacillus kisonensis	DSM-19906	Undefined	GTATTCCCCACGTATGTAGGGGTGATCC	34	19			
Lactobacillus diolivorans	DSM-14421	II	GTTTTAGAAGACTGTTGAATCAATGATGTTTAGTCC	36	74	Y		Y
Lactobacillus hilgardii	DSM-20176	N/A	None found					
Lactobacillus farraginis	DSM-18382	I	GTATTCCCCACGCATGTGGGGGTGATCC	28	9	Y	Y	
Lactobacillus parafarraginis	DSM-18390	I	GTATTCCCCACGCATGTGGGGGTGATCC	28	126	Y	Y	
Lactobacillus senioris	DSM-24302	N/A	None found					
Lactobacillus ozensis	DSM-23829	II	GTTATTAATACATTACAAATACATAGATGTTATAAT	36	37	Y		Y
Lactobacillus kunkeei	DSM-12361	N/A	None found					
Lactobacillus florum	DSM-22689	II	GTTTTAGAAGTACGTCATTCTAATGAGATTAAGAAC	36	10			Y*
Lactobacillus lindneri A	DSM-20690	II	GTTCTTAATCTCATTAGAATGACGTACTTCTAAAAC	36	23	Y		Y
Lactobacillus lindneri B	DSM-20690	Undefined	GTTTTGTAAGTACGTCATTTCAAGTAGTATTAAACC	36	2			
Lactobacillus sanfranciscensis	DSM-20451	N/A	None found					
Lactobacillus fructivorans	ATCC-27394	N/A	None found					
Lactobacillus homohiochii	DSM-20571	N/A	None found					
Lactobacillus fructivorans	DSM-20350	N/A	None found					
Lactobacillus fructivorans	DSM-20203	N/A	None found					
Pediococcus argentinicus	DSM-23026	N/A	None found					
Pediococcus claussenii	DSM-14800	N/A	None found					
Pediococcus pentosaceus	DSM-20336	N/A	None found					
Pediococcus stilesii	DSM-18001	II	GTTTCAGAGGGATGTTAAAGAAGTAGGTTCAATATC	36	28	Y		Y
Pediococcus lolii	DSM-19927	II	GTTTCAGAAGGATGTTAAATCAATAAGGTTAAGATC	36	19	Y		Y
Pediococcus acidilactici	AS1.2696	II	GTTTCAGAAGGATGTTAAATCAATAAGGTTAAGATC	36	11	Y		Y*
Pediococcus ethanolidurans	DSM-22301	N/A	None found					
Pediococcus cellicola	DSM-17757	I	GTCACATCCTTTATGGATGTGTGAATTGAAAT	32	70	Y	Y	
Pediococcus damnosus	DSM-20331	II	GTTTTAGAAGAGTGTCGAATCAATATAGTTAAGAGC	36	20	Y		Y*
Pediococcus inopinatus	DSM-20285	II	GTTTTAGAAGAGTGTCGAATCAATATAGTTAAGATC	36	80	Y		Y
Pediococcus parvulus A	DSM-20332	II	GTTTCAGAAGGATGTTAAATCAATAAGGTTAAGATC	36	15	Y		Y
Pediococcus parvulus B	DSM-20332	II	GTTTTAGAAGAGTGTCGAATCAATATAGTTAAGAGC	36	90	Y		Y
Pediococcus parvulus C	DSM-20332	I	GTCGCATCCTTTATGGGTGCGTGAATTGAAAT	32	80	Y	Y	
Carnobacterium maltaromaticum	DSM-20730	N/A	None found					
Carnobacterium maltaromaticum	DSM-20342	N/A	None found					
Carnobacterium maltaromaticum	DSM-20722	N/A	None found					
Carnobacterium divergens	DSM-20623	N/A	None found					
Lactococcus lactis	LMG-7760	N/A	None found					
Atopobium minutum	DSM-20586	N/A	None found					
Olsenella uli	DSM-7084	II	GTTTTGGGGCAGTGTCGTTTTGACTGGTAATCAAAC	36	30	Y		Y
Atopobium rimae	DSM-7090	Undefined	GTTTTGGAGCAGTGTCATTCTGACTGGTAATCAAAC	36	5	Y*		

Table S8. Sequence information for the 27 core partial genes

PID*	Gene	*L. salivarius* locus tag*	COG	Annotation	Co-ordinates*	Strand*	Length*
90960991	*dnaA*	LSL_0001	COG0593L	chromosomal replication initiation protein	1..1365	+	454
90960994	*recF*	LSL_0004	COG1195L	recombination protein F	3309..4448	+	379
90961461	*murC*	LSL_0485	COG0773M	UDP-N-acetylmuramate--L-alanine ligase	533809..535140	+	443
90961551	*ribF*	LSL_0575	COG0196H	riboflavin kinase/FMN adenylyltransferase	616816..617772	+	318
90961553	*grpE*	LSL_0577	COG0576O	GrpE protein HSP-70 cofactor	618942..619538	+	198
90961554	*dnaK*	LSL_0578	COG0443O	molecular chaperone DnaK	619577..621424	+	615
90961556	*lepA*	LSL_0580	COG0481M	GTP-binding protein LepA	622843..624672	+	609
90961566	*prfA*	LSL_0590	COG0216J	peptide chain release factor 1	633672..634754	+	360
90961572	*atpF*	LSL_0596	COG0711C	ATP synthase subunit B	638577..639104	+	175
90961615	*rpsO*	LSL_0638	COG0184J	30S ribosomal protein S15	681489..681758	+	89
90962017	*ileS*	LSL_1042	COG0060J	isoleucyl-tRNA synthetase	1065203..1067998	-	931
90962030	*mraW*	LSL_1055	COG0275M	S-adenosyl-methyltransferase MraW	1080326..1081270	-	314
90962140	*pgk*	LSL_1165	COG0126G	phosphoglycerate kinase	1199234..1200436	-	400
90962149	*uvrA*	LSL_1174	COG0178L	excinuclease ABC subunit A	1208147..1210981	-	944
90962150	*uvrB*	LSL_1175	COG0556L	excinuclease ABC subunit B	1211000..1213000	-	666
90962161	*secA*	LSL_1186	COG0653U	preprotein translocase subunit SecA	1223594..1225957	-	787
90962203	*dnaX*	LSL_1228	COG2812L	DNA polymerase III subunit gamma/tau	1260545..1262284	-	579
90962211	*rplL*	LSL_1237	COG0222J	50S ribosomal protein L7/L12	1270871..1271239	-	122
90962242	*galU*	LSL_1268	COG1210M	UTP--glucose-1-phosphate uridylyltransferase	1304213..1305085	-	290
90962313	*trmA*	LSL_1341	COG2265J	tRNA (Uracil-5-) -methyltransferase	1392529..1393908	-	459
90962321	*pcrA*	LSL_1349	COG0210L	ATP-dependent DNA helicase	1403083..1405317	-	744
90962375	*truA*	LSL_1404	COG0101J	tRNA pseudouridine synthase A	1478890..1479660	-	256
90962386	*secY*	LSL_1415	COG0201U	preprotein translocase subunit SecY	1486063..1487361	-	432
90962403	*rplB*	LSL_1432	COG0090J	50S ribosomal protein L2	1493766..1494599	-	277
90962565	*parB*	LSL_1596	COG1475K	chromosome partitioning protein, DNA-binding protein	1676145..1677020	-	291
90962568	*gidB*	LSL_1599	COG0357M	16S rRNA methyltransferase GidB	1678668..1679393	-	241
90962695	*dnaB*	LSL_1726	COG0305L	replicative DNA helicase	1807560..1808951	-	463

*These columns are provided according to the reference genome *L. salivarius* UCC118

Table S9. Presence and absence of the complete pathways for production of the 20 standard amino acids.

Species Name	StrainID	Histidine	Valine	Leucine	Isoleucine	Threonine	Lysine	Asparagine	Aspartate	Alanine	Serine	Glycine	Methionine	Cysteine	Tryptophan	Phenlyalanine	Tyrosine	Arginine	Proline	Glutamate	Glutamine
Kandleria vitulina	DSM-20405	P	P	P	P	P	A	P	A	A	P	P	A	P	P	A	A	P	A	P	P
Lactobacillus kimchiensis	DSM-24716	A	A	A	A	P	P	P	A	A	A	A	A	A	A	A	A	A	A	A	P
Lactobacillus mindensis	DSM-14500	A	A	A	A	A	P	A	A	A	P	A	A	A	P	A	A	P	A	P	P
Lactobacillus nantensis	DSM-16982	A	A	A	A	P	P	P	A	A	A	A	A	A	A	A	A	P	A	A	P
Lactobacillus crustorum	LMG-23699	A	A	A	A	A	A	A	A	A	A	A	A	A	A	A	A	A	A	A	A
Lactobacillus crustorum	JCM-15951	A	A	A	A	A	A	P	A	A	A	A	A	A	A	A	A	A	A	A	A
Lactobacillus futsaii	JCM-17355	P	A	A	A	P	P	A	A	A	A	A	A	A	A	A	A	P	A	P	P
Lactobacillus farciminis	DSM-20184	P	A	A	A	P	P	P	A	A	A	A	A	A	A	A	A	P	A	A	P
Lactobacillus alimentarius	DSM-20249	A	A	A	A	P	A	P	A	A	A	A	A	A	A	A	A	A	A	A	P
Lactobacillus paralimentarius	DSM-19674	A	A	A	A	A	P	A	A	A	A	P	A	A	A	A	A	A	A	A	P
Lactobacillus paralimentarius	DSM-13961	A	A	A	A	A	P	P	A	A	P	P	A	A	A	A	A	A	A	A	A
Lactobacillus paralimentarius	DSM-13238	A	A	A	A	A	P	P	A	A	A	A	A	A	A	A	A	A	A	A	P
Lactobacillus tucceti	DSM-20183	P	A	A	A	A	P	P	A	P	A	A	A	A	A	A	A	A	A	A	A
Lactobacillus nodensis	DSM-19682	P	A	A	A	P	P	P	A	A	A	A	A	A	P	A	A	A	A	A	P
Lactobacillus versmoldensis	DSM-14857	P	P	P	P	P	P	P	P	A	A	A	A	A	P	A	A	P	A	P	P
Lactobacillus floricola	DSM-23037	A	A	A	A	P	A	A	A	A	A	A	P	A	A	A	A	A	A	A	A
Lactobacillus acidophilus	DSM-20079	A	A	A	A	A	A	A	A	A	A	A	A	A	A	A	A	A	A	A	A
Lactobacillus amylovorus	DSM-20531	A	A	A	A	P	A	P	A	A	A	A	A	A	A	A	A	A	A	A	A
Lactobacillus amylovorus	DSM-16698	A	A	A	A	P	P	P	A	A	P	A	A	A	A	A	A	A	A	A	P
Lactobacillus kitasatonis	DSM-16761	A	A	A	A	P	P	P	A	A	A	A	A	A	A	A	A	A	A	A	A
Lactobacillus kefiranofaciens kefiranofaciens	DSM-5016	A	A	A	A	A	P	P	P	A	A	A	A	A	A	A	A	A	A	A	A
Lactobacillus kefiranofaciens kefirgranum	DSM-10550	A	A	A	A	A	P	A	A	A	P	A	P	A	A	A	A	A	A	A	P
Lactobacillus ultunensis	DSM-16047	A	A	A	A	P	P	P	A	A	A	A	A	A	A	A	A	A	A	A	A
Lactobacillus helveticus	LMG-22464	A	A	A	A	P	P	P	A	A	A	A	A	A	A	A	A	A	A	A	A
Lactobacillus helveticus	CGMCC-1.1877	A	A	A	A	A	P	P	P	A	A	A	P	A	A	A	A	A	A	A	A
Lactobacillus gallinarum	DSM-10532	A	A	A	A	A	P	P	A	A	A	A	A	A	A	A	A	A	A	A	P
Lactobacillus crispatus	DSM-20584	A	A	A	A	P	P	P	A	A	A	A	A	A	A	A	A	A	A	A	A
Lactobacillus acetotolerans	DSM-20749	P	P	P	P	A	P	A	A	A	A	A	P	A	A	A	A	P	A	P	P
Lactobacillus intestinalis	DSM-6629	A	A	A	A	A	P	P	P	P	A	A	A	A	A	A	A	A	A	A	A
Lactobacillus hamsteri	DSM-5661	A	A	A	A	A	P	P	P	A	A	A	A	A	A	A	A	A	A	A	A
Lactobacillus amylolyticus	DSM-11664	A	A	A	A	A	P	P	P	A	A	A	A	A	A	A	A	A	A	A	A
Lactobacillus kalixensis	DSM-16043	A	A	A	A	P	P	P	A	P	A	A	A	A	A	A	A	A	A	A	P
Lactobacillus gigeriorum	DSM-23908	A	A	A	A	A	A	A	A	A	A	A	A	A	A	A	A	A	A	A	A
Lactobacillus pasteurii	DSM-23907	A	A	A	A	A	A	A	A	A	A	A	A	A	A	A	A	A	A	A	A
Lactobacillus delbrueckii jakobsenii	DSM-26046	A	A	A	A	P	P	P	A	A	A	A	P	A	A	A	A	A	A	A	P
Lactobacillus delbrueckii lactis	DSM-20072	A	A	A	A	P	P	P	A	A	A	A	P	A	A	A	A	A	A	A	P
Lactobacillus delbrueckii bulgaricus	DSM-20081	A	A	A	A	P	A	A	A	A	A	A	A	A	A	A	A	A	A	A	A
Lactobacillus delbrueckii delbrueckii	DSM-20074	A	A	A	A	P	A	A	A	A	A	A	A	A	A	A	A	A	A	A	P
Lactobacillus delbrueckii indicus	DSM-15996	A	A	A	A	P	P	P	A	A	A	A	A	A	A	A	A	A	A	A	P
Lactobacillus equicursoris	DSM-19284	A	A	A	A	P	P	P	A	A	A	A	P	A	A	A	A	A	A	A	P
Lactobacillus jensenii	DSM-20557	P	A	A	A	P	P	A	A	A	A	A	P	A	P	A	A	P	A	A	P
Lactobacillus psittaci	DSM-15354	A	A	A	A	A	A	A	A	A	A	A	A	A	A	A	A	A	A	A	A
Lactobacillus hominis	DSM-23910	A	A	A	A	A	A	A	A	A	A	A	A	A	A	A	A	A	A	A	A
Lactobacillus taiwanensis	DSM-21401	A	A	A	A	A	A	A	A	A	A	A	A	A	A	A	A	A	A	A	A
Lactobacillus johnsonii	ATCC-33200	A	A	A	A	A	P	A	A	A	A	A	A	A	A	A	A	A	A	A	A
Lactobacillus gasseri	ATCC-33323	A	A	A	A	A	A	A	A	A	A	A	A	A	A	A	A	A	A	A	A
Lactobacillus iners	DSM-13335	A	A	A	A	A	A	P	A	A	A	A	A	A	A	A	A	A	A	A	P
Lactobacillus amylotrophicus	DSM-20534	A	A	A	A	A	P	P	A	A	A	A	A	A	A	A	A	A	A	A	A
Lactobacillus amylophilus	DSM-20533	A	A	A	A	A	P	P	A	A	A	A	A	A	A	A	A	A	A	A	A
Lactobacillus dextrinicus	DSM-20335	A	A	A	A	A	A	A	A	A	A	A	A	A	A	A	A	A	A	A	A
Lactobacillus concavus	DSM-17758	A	A	A	A	A	A	A	A	A	A	A	A	A	A	A	A	A	A	A	P
Lactobacillus composti	DSM-18527	P	P	P	P	P	A	A	A	A	P	P	A	A	P	A	A	P	A	P	P
Lactobacillus harbinensis	DSM-16991	A	A	A	A	A	A	P	A	A	A	A	A	A	A	A	A	A	A	A	P
Lactobacillus perolens	DSM-12744	A	A	A	A	A	A	P	A	A	A	A	A	A	A	A	A	A	A	A	P
Lactobacillus camelliae	DSM-22697	A	A	A	A	A	P	P	A	P	A	A	A	A	A	A	A	A	A	A	P
Lactobacillus nasuensis	JCM-17158	A	A	A	A	A	A	A	A	A	A	A	A	A	A	A	A	A	A	A	A
Lactobacillus manihotivorans	DSM-13343	P	A	P	A	P	P	P	P	P	A	P	P	A	P	A	A	P	A	P	P
Lactobacillus paracasei paracasei	DSM-5622	P	A	A	A	A	P	A	A	A	A	A	A	A	A	A	A	A	P	P	P
Lactobacillus casei	DSM-20011	A	A	A	A	P	P	A	A	P	A	A	A	A	A	A	A	A	A	P	P
Lactobacillus paracasei tolerans	DSM-20258	P	A	A	A	P	P	A	A	A	A	A	A	A	A	A	A	A	A	P	P
Lactobacillus zeae	DSM-20178	P	A	A	A	P	P	A	A	A	A	A	A	A	A	A	A	A	A	A	P
Lactobacillus rhamnosus	DSM-20021	P	A	A	A	P	P	A	A	A	A	A	A	A	A	A	A	A	A	P	P
Lactobacillus saniviri	DSM-24301	A	A	A	A	A	P	A	A	A	A	A	A	A	A	A	A	A	A	A	A
Lactobacillus brantae	DSM-23927	A	A	A	A	A	A	A	A	A	A	A	A	A	A	A	A	A	A	A	A
Lactobacillus thailandensis	DSM-22698	A	A	A	A	A	A	A	A	A	A	A	A	A	A	A	A	A	A	A	A
Lactobacillus pantheris	DSM-15945	A	A	A	A	P	A	P	A	A	P	P	P	A	A	A	A	A	A	A	P
Lactobacillus sharpeae	DSM-20505	A	A	A	A	P	P	A	A	A	A	A	A	A	A	A	A	P	A	A	P
Lactobacillus selangorensis	DSM-13344	A	A	A	A	A	A	A	A	P	A	A	A	A	A	A	A	A	A	A	P
Lactobacillus selangorensis	ATCC-BAA-66	A	A	A	A	A	A	A	A	A	A	A	A	A	A	A	A	A	A	A	A
Lactobacillus graminis	DSM-20719	A	A	A	A	A	A	A	A	A	A	A	A	A	A	A	A	A	A	A	A
Lactobacillus curvatus	DSM-20019	A	A	A	A	A	A	A	A	A	A	A	A	A	A	A	A	A	A	A	A
Lactobacillus sakei sakei	DSM-20017	A	A	A	A	A	A	A	A	A	A	A	A	A	A	A	A	A	A	A	A
Lactobacillus sakei carnosus	DSM-15831	A	A	A	A	A	A	A	A	A	A	A	A	A	A	A	A	A	A	A	A
Lactobacillus fuchuensis	DSM-14340	A	A	A	A	A	A	P	A	P	A	A	A	A	A	A	A	A	A	A	P
Lactobacillus rennini	DSM-20253	A	A	A	A	A	A	A	A	A	A	A	A	A	A	A	A	A	A	A	A
Lactobacillus coryniformis torquens	DSM-20004	P	P	P	P	P	P	P	A	A	P	P	P	P	A	A	A	P	A	P	P
Lactobacillus coryniformis coryniformis	DSM-20001	P	P	P	P	P	P	P	A	A	P	P	P	P	A	A	A	P	A	P	P
Lactobacillus bifermentans	DSM-20003	P	P	P	P	P	P	A	A	A	P	P	P	P	P	A	A	P	A	P	P
Lactobacillus ceti	DSM-22408	A	A	A	A	A	A	A	A	A	A	A	A	P	A	A	A	A	A	A	A
Lactobacillus saerimneri	DSM-16049	A	A	A	A	P	P	P	A	A	A	A	A	A	A	A	A	A	A	A	A
Lactobacillus animalis	DSM-20602	A	A	A	A	P	P	P	A	A	A	A	A	A	A	A	A	A	A	A	P
Lactobacillus murinus	DSM-20452	A	A	A	A	P	P	P	A	A	A	A	A	A	A	A	A	A	A	A	P
Lactobacillus apodemi	DSM-16634	A	A	A	A	A	P	P	A	A	A	A	A	A	A	A	A	A	A	A	P
Lactobacillus ruminis	DSM-20403	P	A	A	A	P	P	A	A	A	A	A	A	A	A	A	A	P	A	P	P
Lactobacillus agilis	DSM-20509	A	A	A	A	A	A	P	A	A	A	A	P	A	A	A	A	A	A	A	P
Lactobacillus equi	DSM-15833	A	A	A	A	P	P	A	A	A	A	A	A	A	A	A	A	P	A	A	P
Lactobacillus salivarius	DSM-20555	A	A	A	A	P	P	P	A	A	A	A	A	A	A	A	A	A	A	A	P
Lactobacillus hayakitensis	DSM-18933	A	A	A	A	A	P	P	A	A	A	A	A	A	A	A	A	A	A	A	A
Lactobacillus pobuzihii	NBRC-103219	A	A	A	A	A	P	P	A	A	A	A	A	A	A	A	A	A	A	A	A
Lactobacillus pobuzihii.Chen	KCTC-13174	A	A	A	A	A	P	A	A	A	A	A	A	A	A	A	A	A	A	A	A
Lactobacillus acidipiscis	DSM-15836	A	A	A	A	A	P	P	A	A	A	A	A	P	A	A	A	A	A	A	P
Lactobacillus acidipiscis	DSM-15353	A	A	A	A	A	P	P	A	A	A	P	P	P	A	A	A	A	A	A	A
Lactobacillus aviarius aviarius	DSM-20655	A	A	A	A	A	A	A	A	A	A	A	A	A	A	A	A	A	A	A	A
Lactobacillus aviarius araffinosus	DSM-20653	A	A	A	A	A	A	A	A	A	A	A	A	A	A	A	A	A	A	A	A
Lactobacillus sucicola	DSM-21376	P	P	P	P	P	P	A	A	A	A	A	P	A	P	A	A	P	A	P	P
Lactobacillus aquaticus	DSM-21051	P	P	P	P	P	P	A	A	A	A	A	P	A	P	A	A	P	A	P	P
Lactobacillus uvarum	DSM-19971	P	P	P	P	P	P	A	A	A	P	P	P	P	P	A	A	P	A	P	P
Lactobacillus capillatus	DSM-19910	P	P	P	P	P	P	A	A	A	A	A	A	A	P	A	A	P	A	P	P
Lactobacillus cacaonum	DSM-21116	P	A	A	A	P	P	A	A	A	A	A	A	A	A	A	A	P	A	P	P
Lactobacillus mali	DSM-20444	P	P	P	P	A	P	A	A	A	A	A	P	A	P	A	A	P	P	P	P
Lactobacillus mali	ATCC-27304	P	P	P	P	P	P	A	A	A	P	P	A	P	P	A	A	P	P	P	P
Lactobacillus hordei	DSM-19519	P	A	P	A	P	P	A	A	A	A	A	P	A	A	A	A	P	A	P	P
Lactobacillus oeni	DSM-19972	P	A	A	A	P	P	A	A	A	A	A	A	A	A	A	A	P	A	P	P
Lactobacillus satsumensis	DSM-16230	P	P	P	P	P	P	A	A	A	P	P	A	P	P	A	A	P	A	P	P
Lactobacillus vini	DSM-20605	P	A	A	A	P	P	A	A	A	A	A	A	A	A	A	A	P	A	P	A
Lactobacillus ghanensis	DSM-18630	P	P	P	P	P	P	A	A	A	P	A	A	A	P	A	A	P	A	P	P
Lactobacillus nagelii	DSM-13675	P	A	P	A	P	P	A	A	A	A	A	A	A	A	A	A	P	A	P	P
Lactobacillus algidus	DSM-15638	A	A	A	A	A	A	A	A	A	A	A	A	A	A	A	A	A	A	A	A
Lactobacillus fabifermentans	DSM-21115	P	A	A	A	P	A	A	A	A	A	A	P	P	A	A	A	P	A	A	P
Lactobacillus xiangfangensis	LMG-26013	P	A	A	A	P	A	P	A	A	A	A	P	A	A	A	A	A	A	A	P
Lactobacillus pentosus	DSM-20314	P	A	P	A	P	A	P	A	A	P	P	P	P	P	A	A	A	A	P	P
Lactobacillus plantarum argentoratensis	DSM-16365	P	A	A	A	P	P	P	A	A	P	P	P	P	P	A	A	P	A	A	P
Lactobacillus plantarum	DSM-13273	P	A	A	A	A	P	P	A	A	P	A	P	P	P	A	P	P	A	A	A
Lactobacillus plantarum plantarum	CGMCC-1.2437	A	A	A	A	A	P	P	P	A	P	A	P	A	P	A	A	A	P	A	P
Lactobacillus paraplantarum	DSM-10667	P	A	A	A	A	A	P	P	A	P	A	P	P	P	A	A	P	P	P	P
Lactobacillus siliginis	DSM-22696	A	A	A	A	A	A	A	A	A	A	A	A	A	A	A	A	A	A	A	A
Lactobacillus rossiae	DSM-15814	A	A	A	A	A	P	A	A	A	A	A	A	P	A	A	A	A	A	A	A
Weissella viridescens	DSM-20410	A	A	A	A	A	A	A	A	A	A	A	A	A	A	A	A	A	A	A	A
Weissella minor	DSM-20014	A	A	A	A	A	A	A	A	A	A	A	A	A	A	A	A	A	A	A	A
Weissella halotolerans	DSM-20190	A	A	A	A	A	A	A	A	A	A	A	A	A	A	A	A	A	A	A	A
Weissella confusa	DSM-20196	A	A	A	A	A	A	A	A	A	A	A	A	A	A	A	A	A	A	A	A
Weissella kandleri	DSM-20593	A	A	A	A	A	A	A	A	A	A	A	A	A	A	A	A	A	A	A	A
Oenococcus oeni	ATCC-BAA-1163	A	A	A	A	P	A	A	A	A	A	A	A	A	A	A	A	A	A	A	A
Oenococcus kitaharae	DSM-17330	P	A	A	A	P	A	A	A	A	A	A	A	A	A	A	A	P	A	A	P
Leuconostoc fallax	KCTC-3537	P	A	A	A	P	A	A	A	A	A	A	A	A	A	A	A	P	A	A	P
Leuconostoc pseudomesenteroides	4882	P	A	A	A	P	A	A	A	A	A	A	P	A	A	A	A	P	A	A	P
Leuconostoc mesenteroides	ATCC-8293	P	P	P	P	P	A	A	A	A	P	P	P	P	P	A	A	P	A	P	P
Leuconostoc mesenteroides cremoris	ATCC-19254	P	P	P	P	P	A	A	A	A	P	P	P	P	P	A	A	P	A	A	P
Leuconostoc carnosum	JB16	P	P	P	P	P	A	A	A	A	P	P	P	P	P	A	A	P	A	A	P
Leuconostoc argentinum	KCTC-3773	A	A	A	A	P	A	A	A	A	A	A	A	A	A	A	A	A	A	A	P
Leuconostoc citreum	KM20	A	A	A	A	P	A	A	A	A	A	A	A	A	A	A	A	P	A	A	P
Leuconostoc gelidum	KCTC-3527	A	A	A	A	P	A	A	A	A	A	A	A	A	P	A	A	P	A	A	P
Leuconostoc gasicomitatum	LMG-18811	P	A	A	A	P	A	A	A	A	A	A	A	A	A	A	A	P	A	A	P
Leuconostoc kimchii	IMSNU-11154	P	A	A	A	P	A	A	A	A	A	A	P	A	P	A	A	P	A	A	P
Fructobacillus fructosus	DSM-20349	A	A	A	A	P	A	A	A	A	A	A	A	A	P	A	A	A	A	A	P
Lactobacillus pontis	DSM-8475	A	A	A	A	A	P	P	A	A	A	A	A	A	A	A	A	A	P	A	P
Lactobacillus panis	DSM-6035	A	A	A	A	A	P	P	P	A	A	A	P	A	A	A	A	A	A	P	P
Lactobacillus oris	DSM-4864	A	A	A	A	P	P	A	P	P	A	P	P	A	A	A	A	A	A	A	A
Lactobacillus antri	DSM-16041	A	A	A	A	A	P	P	A	P	A	A	A	A	A	A	A	A	A	A	P
Lactobacillus reuteri	DSM-20016	A	A	A	A	A	P	P	A	A	A	A	A	A	A	A	A	A	A	A	P
Lactobacillus vaginalis	DSM-5837	A	A	A	A	P	P	P	P	A	A	A	P	A	A	A	A	A	A	P	P
Lactobacillus frumenti	DSM-13145	A	A	A	A	A	P	P	P	A	A	A	A	A	A	A	A	A	A	A	P
Lactobacillus fermentum	DSM-20055	A	A	A	A	P	P	P	A	A	A	A	A	A	A	A	A	P	A	A	P
Lactobacillus equigenerosi	DSM-18793	A	A	A	A	P	P	A	A	A	A	A	A	A	A	A	A	A	A	A	A
Lactobacillus gastricus	DSM-16045	P	A	A	A	P	P	A	A	A	A	A	P	A	A	A	A	P	A	A	P
Lactobacillus ingluviei	DSM-15946	A	A	P	A	P	P	P	A	A	P	A	A	P	A	A	A	A	A	A	P
Lactobacillus ingluviei	DSM-14792	A	A	A	A	P	P	P	A	A	A	A	A	A	A	A	A	A	A	A	P
Lactobacillus secaliphilus	DSM-17896	A	A	A	A	A	P	A	A	P	A	A	A	A	A	A	A	A	A	A	P
Lactobacillus coleohominis	DSM-14060	A	A	A	A	A	A	A	A	A	A	A	A	A	A	A	A	A	A	A	A
Lactobacillus mucosae	DSM-13345	A	A	A	A	P	P	P	A	A	A	P	A	A	A	A	A	P	A	A	P
Lactobacillus oligofermentans	DSM-15707	A	A	A	A	P	P	A	A	A	A	A	A	P	A	A	A	A	A	A	P
Lactobacillus hokkaidonensis	DSM-26202	P	A	A	A	P	A	A	A	P	A	A	A	P	P	A	A	P	A	A	P
Lactobacillus suebicus	DSM-5007	P	P	P	P	P	P	A	A	A	P	P	P	P	P	A	A	P	A	A	P
Lactobacillus vaccinostercus	DSM-20634	A	P	P	P	P	A	A	A	A	P	P	A	P	P	A	A	P	A	P	P
Lactobacillus parabrevis	LMG-11984	A	A	A	A	A	A	A	A	A	A	A	A	A	A	A	A	A	A	A	A
Lactobacillus parabrevis	ATCC-53295	A	A	A	A	A	A	A	A	A	A	A	A	A	A	A	A	A	A	A	A
Lactobacillus hammesii	DSM-16381	A	A	A	A	A	A	A	A	A	A	A	A	A	A	A	A	A	A	A	A
Lactobacillus paucivorans	DSM-22467	A	A	A	A	A	A	A	A	A	A	A	A	A	A	A	A	A	A	A	A
Lactobacillus senmaizukei	DSM-21775	A	A	A	A	A	A	A	A	A	A	A	A	A	A	A	A	A	A	A	A
Lactobacillus brevis	DSM-20054	A	A	A	A	A	A	A	A	A	A	A	A	A	A	A	A	A	A	A	A

Lactobacillus koreensis	JCM-16448	A	A	A	A	A	P	P	A	A	A	A	A	A	A	A	A	A	A	A	A
Lactobacillus zymae	DSM-19395	A	A	A	A	A	P	P	A	A	A	A	A	A	A	A	A	A	A	A	A
Lactobacillus acidifarinae	DSM-19394	A	A	A	A	A	P	P	A	A	A	A	A	A	A	A	A	A	A	A	P
Lactobacillus namurensis	DSM-19117	A	A	A	A	A	A	A	A	A	A	A	A	A	A	A	A	A	A	A	A
Lactobacillus spicheri	DSM-15429	A	A	A	A	A	A	A	A	A	A	P	A	A	A	A	A	A	A	A	P
Lactobacillus kimchicus	JCM-15530	P	P	P	P	P	A	P	P	A	P	P	P	P	P	A	A	P	A	P	P
Lactobacillus similis	DSM-23365	P	P	P	P	P	A	P	A	P	P	P	P	P	P	A	A	P	P	P	P
Lactobacillus odoratitofui	DSM-19909	P	A	A	A	P	P	P	A	A	P	P	P	P	P	A	A	P	A	P	P
Lactobacillus collinoides	DSM-20515	P	P	P	P	P	P	P	A	A	P	P	P	P	P	A	A	P	A	P	P
Lactobacillus paracollinoides	DSM-15502	P	P	P	P	P	P	P	A	A	P	P	P	A	P	A	A	P	A	P	P
Lactobacillus malefermentans	DSM-5705	A	A	A	A	A	P	A	A	A	A	A	A	A	P	A	A	A	A	A	P
Lactobacillus parabuchneri	DSM-5707	P	P	P	A	P	P	A	A	A	A	A	P	A	P	A	A	P	P	P	P
Lactobacillus parabuchneri	DSM-15352	P	A	P	A	P	P	A	A	A	A	A	P	A	P	A	A	P	A	P	P
Lactobacillus buchneri	DSM-20057	A	A	A	A	A	A	A	A	A	A	A	A	A	A	A	A	A	A	A	A
Lactobacillus otakiensis	DSM-19908	P	A	A	A	P	P	A	A	P	A	A	P	A	P	A	A	P	A	A	P
Lactobacillus kefiri	DSM-20587	A	A	A	A	P	A	A	A	A	A	A	A	A	P	A	A	P	A	P	P
Lactobacillus parakefiri	DSM-10551	P	A	A	A	P	P	A	A	A	A	A	P	A	P	A	A	P	P	P	P
Lactobacillus sunkii	DSM-19904	P	A	A	A	P	P	A	A	A	A	A	P	A	P	A	A	P	A	A	P
Lactobacillus rapi	DSM-19907	P	P	P	P	P	P	A	A	A	P	A	A	P	P	A	A	P	A	P	P
Lactobacillus kisonensis	DSM-19906	A	A	A	A	P	P	A	A	A	A	A	A	P	P	A	A	A	A	A	P
Lactobacillus diolivorans	DSM-14421	P	A	A	A	P	P	A	A	A	P	P	P	P	P	A	A	P	A	P	P
Lactobacillus hilgardii	DSM-20176	P	P	P	P	P	P	A	A	A	P	P	P	P	P	A	A	P	A	P	P
Lactobacillus farraginis	DSM-18382	A	A	A	A	A	P	P	A	A	A	A	A	A	A	A	A	A	A	A	A
Lactobacillus parafarraginis	DSM-18390	P	P	P	P	P	P	A	A	A	P	P	P	P	P	A	A	P	A	P	P
Lactobacillus senioris	DSM-24302	A	A	A	A	A	A	A	A	A	A	A	A	A	A	A	A	A	A	A	A
Lactobacillus ozensis	DSM-23829	A	A	A	A	P	A	A	A	A	A	A	A	A	A	A	A	A	A	A	A
Lactobacillus kunkeei	DSM-12361	A	A	A	A	P	P	A	A	A	A	A	A	A	A	A	A	P	A	P	P
Lactobacillus florum	DSM-22689	A	A	A	A	A	A	A	A	A	A	A	A	A	A	A	A	A	A	A	A
Lactobacillus lindneri	DSM-20690	A	A	A	A	A	A	A	A	A	A	A	A	A	A	A	A	A	A	A	A
Lactobacillus sanfranciscensis	DSM-20451	A	A	A	A	A	A	A	A	A	A	A	A	A	A	A	A	A	A	A	A
Lactobacillus fructivorans	ATCC-27394	A	A	A	A	A	P	A	A	A	A	A	A	A	A	A	A	A	A	A	A
Lactobacillus homohiochii	DSM-20571	A	A	A	A	A	A	A	A	A	A	A	A	A	A	A	A	A	A	A	A
Lactobacillus fructivorans	DSM-20350	A	A	A	A	A	A	A	A	A	A	A	A	A	A	A	A	A	A	A	A
Lactobacillus fructivorans	DSM-20203	A	A	A	A	A	A	A	A	A	A	A	A	A	A	A	A	A	A	A	A
Pediococcus argentinicus	DSM-23026	A	A	A	A	A	P	A	A	A	A	A	A	P	A	A	A	A	A	A	A
Pediococcus claussenii	DSM-14800	A	A	A	A	A	P	A	A	A	A	A	A	A	A	A	A	A	A	A	P
Pediococcus pentosaceus	DSM-20336	A	A	A	A	A	A	P	A	A	A	A	A	A	A	A	A	A	A	A	A
Pediococcus stilesii	DSM-18001	A	A	A	A	A	P	P	A	A	A	A	A	A	A	A	A	P	A	A	P
Pediococcus lolii	DSM-19927	A	A	A	A	A	A	A	A	A	A	A	A	A	A	A	A	A	A	A	A
Pediococcus acidilactici	AS1-2696	A	A	A	A	A	A	A	A	A	A	A	A	A	A	A	A	A	A	A	A
Pediococcus ethanolidurans	DSM-22301	A	A	A	A	A	A	A	A	A	A	A	A	A	A	A	A	A	A	A	A
Pediococcus cellicola	DSM-17757	A	A	A	A	A	P	A	A	A	A	A	A	A	A	A	A	A	A	A	P
Pediococcus damnosus	DSM-20331	A	A	A	A	A	A	A	A	A	A	A	A	A	A	A	A	A	A	A	A
Pediococcus inopinatus	DSM-20285	A	A	A	A	A	A	A	A	A	A	A	A	A	A	A	A	A	A	A	A
Pediococcus parvulus	DSM-20332	A	A	A	A	A	P	P	A	A	A	A	A	A	A	A	A	A	A	A	P
Carnobacterium maltaromaticum	DSM-20730	A	P	P	P	P	A	P	A	A	A	A	A	P	P	A	A	P	A	A	P
Carnobacterium maltaromaticum	DSM-20342	A	P	P	P	A	A	P	A	A	A	A	P	P	P	A	A	P	A	A	P
Carnobacterium maltaromaticum	DSM-20722	A	P	P	P	P	A	A	A	A	A	A	A	A	P	A	A	P	A	A	P
Carnobacterium divergens	DSM-20623	A	P	P	P	P	P	P	A	A	P	P	A	P	A	A	A	P	A	A	A
Lactococcus lactis	LMG-7760	P	P	P	P	P	A	A	A	P	P	P	P	P	P	A	A	P	A	P	P
Atopobium minutum	DSM-20586	A	A	A	A	A	A	A	A	A	A	A	A	A	A	A	A	A	A	A	A
Olsenella uli	DSM-7084	P	A	A	A	P	P	A	P	P	A	A	A	A	A	A	A	A	A	P	P
Atopobium rimae	DSM-7090	A	A	A	A	P	A	P	A	A	A	A	P	A	A	A	A	A	A	A	P

Table S10. Presence of sirtuin homologs in the 213 genomes analyzed

Species Name	StrainID	SIR2 family proteins (homolog of mammalian SIRT1, SIR2L1, Sir2α)
Kandleria vitulina	DSM-20405	2
Lactobacillus kimchiensis	DSM-24716	1
Lactobacillus mindensis	DSM-14500	1
Lactobacillus nantensis	DSM-16982	1
Lactobacillus crustorum	LMG-23699	1
Lactobacillus crustorum	JCM-15951	1
Lactobacillus futsaii	JCM-17355	3
Lactobacillus farciminis	DSM-20184	2
Lactobacillus alimentarius	DSM-20249	2
Lactobacillus paralimentarius	DSM-19674	2
Lactobacillus paralimentarius	DSM-13961	2
Lactobacillus paralimentarius	DSM-13238	1
Lactobacillus tucceti	DSM-20183	2
Lactobacillus nodensis	DSM-19682	1
Lactobacillus versmoldensis	DSM-14857	3
Lactobacillus floricola	DSM-23037	0
Lactobacillus acidophilus	DSM-20079	1
Lactobacillus amylovorus	DSM-20531	1
Lactobacillus amylovorus	DSM-16698	1
Lactobacillus kitasatonis	DSM-16761	1
Lactobacillus kefiranofaciens kefiranofaciens	DSM-5016	3
Lactobacillus kefiranofaciens kefirgranum	DSM-10550	3
Lactobacillus ultunensis	DSM-16047	4
Lactobacillus helveticus	LMG-22464	3
Lactobacillus helveticus	CGMCC-1.1877	3
Lactobacillus gallinarum	DSM-10532	1
Lactobacillus crispatus	DSM-20584	1
Lactobacillus acetotolerans	DSM-20749	1
Lactobacillus intestinalis	DSM-6629	1
Lactobacillus hamsteri	DSM-5661	3
Lactobacillus amylolyticus	DSM-11664	1
Lactobacillus kalixensis	DSM-16043	1
Lactobacillus gigeriorum	DSM-23908	1
Lactobacillus pasteurii	DSM-23907	1
Lactobacillus delbrueckii jakobsenii	DSM-26046	1
Lactobacillus delbrueckii lactis	DSM-20072	1
Lactobacillus delbrueckii bulgaricus	DSM-20081	1
Lactobacillus delbrueckii delbrueckii	DSM-20074	1
Lactobacillus delbrueckii indicus	DSM-15996	1
Lactobacillus equicursoris	DSM-19284	1
Lactobacillus jensenii	DSM-20557	1
Lactobacillus psittaci	DSM-15354	1
Lactobacillus hominis	DSM-23910	1
Lactobacillus taiwanensis	DSM-21401	1

Lactobacillus johnsonii	ATCC-33200	2
Lactobacillus gasseri	ATCC-33323	1
Lactobacillus iners	DSM-13335	0
Lactobacillus amylotrophicus	DSM-20534	1
Lactobacillus amylophilus	DSM-20533	1
Lactobacillus dextrinicus	DSM-20335	1
Lactobacillus concavus	DSM-17758	1
Lactobacillus composti	DSM-18527	1
Lactobacillus harbinensis	DSM-16991	1
Lactobacillus perolens	DSM-12744	1
Lactobacillus camelliae	DSM-22697	1
Lactobacillus nasuensis	JCM-17158	1
Lactobacillus manihotivorans	DSM-13343	1
Lactobacillus paracasei paracasei	DSM-5622	1
Lactobacillus casei	DSM-20011	1
Lactobacillus paracasei tolerans	DSM-20258	1
Lactobacillus zeae	DSM-20178	2
Lactobacillus rhamnosus	DSM-20021	2
Lactobacillus saniviri	DSM-24301	1
Lactobacillus brantae	DSM-23927	1
Lactobacillus thailandensis	DSM-22698	1
Lactobacillus pantheris	DSM-15945	1
Lactobacillus sharpeae	DSM-20505	0
Lactobacillus selangorensis	DSM-13344	1
Lactobacillus selangorensis	ATCC-BAA-66	1
Lactobacillus graminis	DSM-20719	3
Lactobacillus curvatus	DSM-20019	3
Lactobacillus sakei sakei	DSM-20017	0
Lactobacillus sakei carnosus	DSM-15831	0
Lactobacillus fuchuensis	DSM-14340	0
Lactobacillus rennini	DSM-20253	1
Lactobacillus coryniformis torquens	DSM-20004	2
Lactobacillus coryniformis coryniformis	DSM-20001	1
Lactobacillus bifermentans	DSM-20003	1
Lactobacillus ceti	DSM-22408	1
Lactobacillus saerimneri	DSM-16049	0
Lactobacillus animalis	DSM-20602	0
Lactobacillus murinus	DSM-20452	0
Lactobacillus apodemi	DSM-16634	1
Lactobacillus ruminis	DSM-20403	1
Lactobacillus agilis	DSM-20509	1
Lactobacillus equi	DSM-15833	2
Lactobacillus salivarius	DSM-20555	1
Lactobacillus hayakitensis	DSM-18933	0
Lactobacillus pobuzihii	NBRC-103219	1
Lactobacillus pobuzihii.Chen	KCTC-13174	1
Lactobacillus acidipiscis	DSM-15836	1

Lactobacillus acidipiscis	DSM-15353	1
Lactobacillus aviarius aviarius	DSM-20655	1
Lactobacillus aviarius araffinosus	DSM-20653	1
Lactobacillus sucicola	DSM-21376	1
Lactobacillus aquaticus	DSM-21051	1
Lactobacillus uvarum	DSM-19971	1
Lactobacillus capillatus	DSM-19910	1
Lactobacillus cacaonum	DSM-21116	0
Lactobacillus mali	DSM-20444	0
Lactobacillus mali	ATCC-27304	0
Lactobacillus hordei	DSM-19519	0
Lactobacillus oeni	DSM-19972	1
Lactobacillus satsumensis	DSM-16230	1
Lactobacillus vini	DSM-20605	1
Lactobacillus ghanensis	DSM-18630	1
Lactobacillus nagelii	DSM-13675	1
Lactobacillus algidus	DSM-15638	0
Lactobacillus fabifermentans	DSM-21115	1
Lactobacillus xiangfangensis	LMG-26013	1
Lactobacillus pentosus	DSM-20314	1
Lactobacillus plantarum argentoratensis	DSM-16365	1
Lactobacillus plantarum	DSM-13273	1
Lactobacillus plantarum plantarum	CGMCC-1.2437	1
Lactobacillus paraplantarum	DSM-10667	1
Lactobacillus siliginis	DSM-22696	1
Lactobacillus rossiae	DSM-15814	1
Weissella viridescens	DSM-20410	0
Weissella minor	DSM-20014	0
Weissella halotolerans	DSM-20190	1
Weissella confusa	DSM-20196	1
Weissella kandleri	DSM-20593	0
Oenococcus oeni	ATCC-BAA-11[illegible]	1
Oenococcus kitaharae	DSM-17330	1
Leuconostoc fallax	KCTC-3537	0
Leuconostoc pseudomesenteroides	4882	1
Leuconostoc mesenteroides	ATCC-8293	1
Leuconostoc mesenteroides cremoris	ATCC-19254	1
Leuconostoc carnosum	JB16	0
Leuconostoc argentinum	KCTC-3773	1
Leuconostoc citreum	KM20	1
Leuconostoc gelidum	KCTC-3527	0
Leuconostoc gasicomitatum	LMG-18811	0
Leuconostoc kimchii	IMSNU-11154	0
Fructobacillus fructosus	DSM-20349	1
Lactobacillus pontis	DSM-8475	1
Lactobacillus panis	DSM-6035	2
Lactobacillus oris	DSM-4864	1

Lactobacillus antri	DSM-16041	1
Lactobacillus reuteri	DSM-20016	1
Lactobacillus vaginalis	DSM-5837	1
Lactobacillus frumenti	DSM-13145	2
Lactobacillus fermentum	DSM-20055	1
Lactobacillus equigenerosi	DSM-18793	1
Lactobacillus gastricus	DSM-16045	1
Lactobacillus ingluviei	DSM-15946	1
Lactobacillus ingluviei	DSM-14792	1
Lactobacillus secaliphilus	DSM-17896	1
Lactobacillus coleohominis	DSM-14060	0
Lactobacillus mucosae	DSM-13345	1
Lactobacillus oligofermentans	DSM-15707	1
Lactobacillus hokkaidonensis	DSM-26202	1
Lactobacillus suebicus	DSM-5007	1
Lactobacillus vaccinostercus	DSM-20634	1
Lactobacillus parabrevis	LMG-11984	1
Lactobacillus parabrevis	ATCC-53295	1
Lactobacillus hammesii	DSM-16381	1
Lactobacillus paucivorans	DSM-22467	1
Lactobacillus senmaizukei	DSM-21775	1
Lactobacillus brevis	DSM-20054	1
Lactobacillus koreensis	JCM-16448	1
Lactobacillus zymae	DSM-19395	1
Lactobacillus acidifarinae	DSM-19394	1
Lactobacillus namurensis	DSM-19117	1
Lactobacillus spicheri	DSM-15429	1
Lactobacillus kimchicus	JCM-15530	1
Lactobacillus similis	DSM-23365	1
Lactobacillus odoratitofui	DSM-19909	1
Lactobacillus collinoides	DSM-20515	1
Lactobacillus paracollinoides	DSM-15502	1
Lactobacillus malefermentans	DSM-5705	1
Lactobacillus parabuchneri	DSM-5707	1
Lactobacillus parabuchneri	DSM-15352	1
Lactobacillus buchneri	DSM-20057	1
Lactobacillus otakiensis	DSM-19908	1
Lactobacillus kefiri	DSM-20587	1
Lactobacillus parakefiri	DSM-10551	2
Lactobacillus sunkii	DSM-19904	2
Lactobacillus rapi	DSM-19907	1
Lactobacillus kisonensis	DSM-19906	1
Lactobacillus diolivorans	DSM-14421	1
Lactobacillus hilgardii	DSM-20176	1
Lactobacillus farraginis	DSM-18382	1
Lactobacillus parafarraginis	DSM-18390	2
Lactobacillus senioris	DSM-24302	1

Lactobacillus ozensis	DSM-23829	1
Lactobacillus kunkeei	DSM-12361	1
Lactobacillus florum	DSM-22689	1
Lactobacillus lindneri	DSM-20690	1
Lactobacillus sanfranciscensis	DSM-20451	1
Lactobacillus fructivorans	ATCC-27394	1
Lactobacillus homohiochii	DSM-20571	1
Lactobacillus fructivorans	DSM-20350	1
Lactobacillus fructivorans	DSM-20203	1
Pediococcus argentinicus	DSM-23026	0
Pediococcus claussenii	DSM-14800	0
Pediococcus pentosaceus	DSM-20336	0
Pediococcus stilesii	DSM-18001	0
Pediococcus lolii	DSM-19927	0
Pediococcus acidilactici	AS1-2696	0
Pediococcus ethanolidurans	DSM-22301	0
Pediococcus cellicola	DSM-17757	0
Pediococcus damnosus	DSM-20331	0
Pediococcus inopinatus	DSM-20285	0
Pediococcus parvulus	DSM-20332	0
Carnobacterium maltaromaticum	DSM-20730	0
Carnobacterium maltaromaticum	DSM-20342	0
Carnobacterium maltaromaticum	DSM-20722	0
Carnobacterium divergens	DSM-20623	1
Lactococcus lactis	LMG-7760	0
Atopobium minutum	DSM-20586	0
Olsenella uli	DSM-7084	2
Atopobium rimae	DSM-7090	1

Table S11. Genomic regions related to bacteriocin production identified in the 213 genomes.

Species Name	StrainID	Number of AOI identified in BAGEL	Bacteriocin homolog 1	CLASS	Confirmed in Artemis	Bacteriocin homolog 2	CLASS	Confirmed in Artemis	Bacteriocin homolog 3	CLASS	Confirmed in Artemis	Bacteriocin homolog 4	CLASS	Confirmed in Artemis	Bacteriocin homolog 5	CLASS	Confirmed in Artemis	Bacteriocin homolog 6	CLASS	Confirmed in Artemis	Bacteriocin homolog 7	CLASS	Confirmed in Artemis
Kandleria vitulina	DSM-20405	0																					
Lactobacillus kimchiensis	DSM-24716	1	Carnocin_CP52	Unmodified	Potential																		
Lactobacillus mindensis	DSM-14500	1	Carnocin_CP52	Unmodified	Potential																		
Lactobacillus nantensis	DSM-16982	2	Lactocin	Unmodified	Yes	Carnocin_CP52	Unmodified	Not a bacteriocin operon															
Lactobacillus crustorum	LMG-23699	0																					
Lactobacillus crustorum	JCM-15951	0																					
Lactobacillus futsaii	JCM-17355	1	Plantaricin like	Unmodified	Potential but across multiple contigs																		
Lactobacillus farciminis	DSM-20184	0																					
Lactobacillus alimentarius	DSM-20249	0																					
Lactobacillus paralimentarius	DSM-19674	0																					
Lactobacillus paralimentarius	DSM-13961	2	Sackacin /Enterocin	Unmodified	Yes	Carnocin	Unmodified	Not a bacteriocin operon															
Lactobacillus paralimentarius	DSM-13238	1	Carnocin_CP52	Unmodified	Potential																		
Lactobacillus tucceti	DSM-20183	0																					
Lactobacillus nodensis	DSM-19682	2	Carnocin_CP52	Unmodified	Not a bacteriocin operon	Gassericin A	Unmodified	Potential															
Lactobacillus versmoldensis	DSM-14857	0																					
Lactobacillus floricola	DSM-23037	0																					
Lactobacillus acidophilus	DSM-20079	3	Enterolysin_A	Bacteriocin >10kd	Not a bacteriocin operon	Lactacin f		Yes	Helveticin J	Bacteriocin >10kd	Not a bacteriocin operon												
Lactobacillus amylovorus	DSM-20531	7	Helveticin J	Bacteriocin >10kd	Not a bacteriocin operon	Enterolysin	Bacteriocin >10kd	Potential	Helveticin J	Bacteriocin >10kd	Potential	Enterolysin A	Unmodified	Not a bacter	Unknown	Lanthipeptic	Potential	Gassericin K7	Unmodified	Not a bacter	Helveticin	Bacteriocin	Tranposase i
Lactobacillus amylovorus	DSM-16698	5	Enterolysin_A	Bacteriocin >10kd	Not a bacteriocin operon	Helveticin_J	Bacteriocin >10kd	Potential	Helveticin_J	Bacteriocin >10kd	Not a bacteriocin operc	Helveticin_J	Bacteriocin	Not a bacter	Helveticin_J	Bacteriocin	Not a bacteriocin operon						
Lactobacillus kitasatonis	DSM-16761	3	Helveticin J	Bacteriocin >10kd	Not a bacteriocin operon	Helveticin_J	Bacteriocin >10kd	Not a bacteriocin operon	Enterolysin A	Bacteriocin >10kd	Not a bacteriocin operon												
Lactobacillus kefiranofaciens kefiranofaciens	DSM-5016	0																					
Lactobacillus kefiranofaciens kefirgranum	DSM-10550	3	Helveticin	Bacteriocin >10kd	Potential	Enterolysin	Bacteriocin >10kd	Potential but across multiple co	Helveticin_J	Bacteriocin >10kd	Not a bacteriocin operon												
Lactobacillus ultunensis	DSM-16047	3	Helveticin J	Bacteriocin >10kd	Potential	Enterolysin	Bacteriocin >10kd	potential	Helveticin_J	Bacteriocin >10kd	Not a bacteriocin operon												
Lactobacillus helveticus	LMG-22464	4	Enterolysin_A	Bacteriocin >10kd	Potential but across multiple contigs	Enterolysin_A	Bacteriocin >10kd	Potential but across multiple co	Helveticin J	Bacteriocin >10kd	Potential but across mu	Helveticin J	Bacteriocin	Not a bacteriocin operon									
Lactobacillus helveticus	CGMCC-1.1877	3	Enterolysin_A	Glyocin	Not a bacteriocin operon	Helveticin_J	Bacteriocin >10kd	Potential	Helveticin_J	Bacteriocin >10kd	Potential												
Lactobacillus gallinarum	DSM-10532	4	Enterolysin_A	Bacteriocin >10kd	Not a bacteriocin operon	Helveticin_J	Bacteriocin >10kd	Potential but across multiple co	Helveticin_J	Bacteriocin >10kd	Not a bacteriocin operc	Helveticin_J	Bacteriocin	Not a bacteriocin operon									
Lactobacillus crispatus	DSM-20584	6	Helveticin	Bacteriocin >10kd	Not a bacteriocin operon	Thermophilin_A	Unmodified	Potential	Lactacin F	Unmodified	Yes	Enterolysin A	Bacteriocin	Not a bacter	Streptolysin	Bacteriocin	Potential but across multiple contigs						
Lactobacillus acetotolerans	DSM-20749	1	Enterolysin_A	Unmodified	Potential but across multiple contigs																		
Lactobacillus intestinalis	DSM-6629	6	Streptolysin	LAPs	Yes	Helveticin_J	Bacteriocin >10kd	Not a bacteriocin operon	Planataracin	Unmodified	Potential	Helveticin_J	Bacteriocin	Not a bacter	Helveticin_J	Bacteriocin	Not a bacter	Enterolysin	Bacteriocin	Not a bacteriocin operon			
Lactobacillus hamsteri	DSM-5661	3	Helveticin J	Bacteriocin >10kd	Not a bacteriocin operon	Enterolysin_A	Bacteriocin >10kd	Not a bacteriocin operon	Enterolysin A/Lacto	Unmodified	Potential												
Lactobacillus amylolyticus	DSM-11664	3	Helveticin	Bacteriocin >10kd	Not a bacteriocin operon	Enterolysin	Bacteriocin >10kd	Not a bacteriocin operon	Helveticin_J	Bacteriocin >10kd	Potential but across multiple contigs												
Lactobacillus kalixensis	DSM-16043	6	Helveticin J	Bacteriocin >10kd	Potential	Enterolysin	Bacteriocin >10kd	Yes	Helveticin_J	Bacteriocin >10kd	Potential but across mu	Helveticin_J	Bacteriocin	Not a bacter	Lactococcin A	Unmodified	Not a bacter	Enterolysin	Bacteriocin	Not a bacteriocin operon			
Lactobacillus gigeriorum	DSM-23908	2	entetolysin_A	Bacteriocin >10kd	Potential but across multiple contigs	Helveticin_J	Bacteriocin >10kd	Not a bacteriocin operon															
Lactobacillus pasteurii	DSM-23907	4	Enterolysin_A	Bacteriocin >10kd	Yes	Bovicin		Potenital	Helveticin J	Bacteriocin >10kd	Not a bacteriocin operc	Helveticin J	Bacteriocin	Not a bacteriocin operon									
Lactobacillus delbrueckii jakobsenii	DSM-26046	1	Enterolysin_A	Bacteriocin >10kd	Not a bacteriocin operon																		
Lactobacillus delbrueckii lactis	DSM-20072	1	Enterolysin_A	Bacteriocin >10kd	Not a bacteriocin operon																		
Lactobacillus delbrueckii bulgaricus	DSM-20081	1	Enterolysin_A	Bacteriocin >10kd	Potential																		
Lactobacillus delbrueckii delbrueckii	DSM-20074	1	Enterolysin_A	Bacteriocin >10kd	Not a bacteriocin operon																		
Lactobacillus delbrueckii indicus	DSM-15996	1	Enterolysin_A	Bacteriocin >10kd	Not a bacteriocin operon																		
Lactobacillus equicursoris	DSM-19284	3	Enterolysin_A	Bacteriocin >10kd	Not a bacteriocin operon	Lactococcin	Unmodified	Potential	Helveticin_J	Bacteriocin >10kd	Potential												
Lactobacillus jensenii	DSM-20557	0																					
Lactobacillus psittaci	DSM-15354	0																					
Lactobacillus hominis	DSM-23910	1	Helveticin J	Bacteriocin >10kd	Yes																		
Lactobacillus taiwanensis	DSM-21401	3	Subtilin	Lanthipeptide_class_	Yes	Helveticin_J	Bacteriocin >10kd	Potenital	Lactacin F /Pediocir	Unmodified	Potential												
Lactobacillus johnsonii	ATCC-33200	3	Enterolysin_A	Bacteriocin >10kd	Not a bacteriocin operon	Helveticin_J	Bacteriocin >10kd	Not a bacteriocin operon	Pediocin	Unmodified	Yes												
Lactobacillus gasseri	ATCC-33323	0																					
Lactobacillus iners	DSM-13335	0																					
Lactobacillus amylotrophicus	DSM-20534	1	Enterolysin_A	Unmodified	Not a bacteriocin operon																		
Lactobacillus amylophilus	DSM-20533	1	Lactococcin_972	Unmodified	Yes																		
Lactobacillus dextrinicus	DSM-20335	0																					
Lactobacillus concavus	DSM-17758	0																					
Lactobacillus composti	DSM-18527	2	Enterolysin_A	Bacteriocin >10kd	Prophage	Pediocin	Bacteriocin >10kd	yes															
Lactobacillus harbinensis	DSM-16991	1	Plantaricin	Unmodified	Potential but across multiple contigs																		
Lactobacillus perolens	DSM-12744	0																					
Lactobacillus camelliae	DSM-22697	0																					
Lactobacillus nasuensis	JCM-17158	0																					
Lactobacillus manihotivorans	DSM-13343	0																					
Lactobacillus paracasei paracasei	DSM-5622	4	Unknown	Head_to_tail_cyclize	Not a bacteriocin operon	Gassericin A	Unmodified	Not a bacteriocin operon	Lactococcin A	Unmodified	Yes	Carnobacteric	Unmodified	Not a bacteriocin operon									
Lactobacillus casei	DSM-20011	3	Unknown	Head_to_tail_cyclize	Potential	Enterocin_X_chain_bet	Unmodified	Potential	Unknown	Unmodified	Not a bacteriocin operon												
Lactobacillus paracasei tolerans	DSM-20258	1	Unknown	Head_to_tail_cyclize	Potential																		
Lactobacillus zeae	DSM-20178	3																					
Lactobacillus rhamnosus	DSM-20021	2	Unknown	Head_to_tail_cyclize	Potential	Lactobin A/Cerein 7B	Unmodified	Yes															
Lactobacillus saniviri	DSM-24301	1	Enterolysin_A	Bacteriocin >10kd	Yes																		
Lactobacillus brantae	DSM-23927	0																					
Lactobacillus thailandensis	DSM-22698	0																					
Lactobacillus pantheris	DSM-15945	0																					
Lactobacillus sharpeae	DSM-20505	0																					
Lactobacillus selangorensis	DSM-13344	1	Enterolysin_A	Bacteriocin >10kd	Not a bacteriocin operon																		
Lactobacillus selangorensis	ATCC-BAA-66	1	Enterolysin_A	Bacteriocin >10kd	Not a bacteriocin operon																		
Lactobacillus graminis	DSM-20719	1	Lactococcin_972	Unmodified	Yes																		
Lactobacillus curvatus	DSM-20019	0																					
Lactobacillus sakei sakei	DSM-20017	0																					
Lactobacillus sakei carnosus	DSM-15831	0																					
Lactobacillus fuchuensis	DSM-14340	1	Sackacin	Unmodified	Not a bacteriocin operon																		
Lactobacillus rennini	DSM-20253	1	Pediocin Ach	Bacteriocin >10kd	Yes																		
Lactobacillus coryniformis torquens	DSM-20004	2	Enterolysin_A	Bacteriocin >10kd	Not a bacteriocin operon	Unknown	Head_to_tail_cycliz	Potenital															
Lactobacillus coryniformis coryniformis	DSM-20001	1	Unknown	Head_to_tail_cyclize	Potential																		
Lactobacillus bifermentans	DSM-20003	0																					
Lactobacillus ceti	DSM-22408	0																					
Lactobacillus saerimneri	DSM-16049	0																					
Lactobacillus animalis	DSM-20602	0																					
Lactobacillus murinus	DSM-20452	4	Hiracin_JM79	Unmodified	Not a bacteriocin operon	Plantaricin_S	Unmodified	Potential but across multiple co	Plantaricin_NC8	Unmodified	Potential but across mu	Enterolysin A	Unmodified	Not a bacteriocin operon									
Lactobacillus apodemi	DSM-16634	2	Enterolysin_A	Bacteriocin >10kd	Not a bacteriocin operon	Plantacarin	Unmodified	Yes															
Lactobacillus ruminis	DSM-20403	1	Pediocin-like	Unmodified	Potential but across multiple contigs																		

Lactobacillus agilis	DSM-20509	1	Plantacarin A	Unmodified	Yes						
Lactobacillus equi	DSM-15833	0									
Lactobacillus salivarius	DSM-20555	0									
Lactobacillus hayakitensis	DSM-18933	2	Enterolysin_A	Bacteriocin >10kd	Not a bacteriocin operon	Enterolysin	Bacteriocin >10kd	Not a bacteriocin operon			
Lactobacillus pobuzihii	NBRC-103219	0									
Lactobacillus pobuzihii.Chen	KCTC-13174	0									
Lactobacillus acidipiscis	DSM-15836	3	Plantaricin_K /Carn	Unmodified	Potential but across multiple contigs	Pediocin/Colicin V	Unmodified	Potential but across multiple co	Pediocin/Colicin V	Unmodified	Potential but across multiple contigs
Lactobacillus acidipiscis	DSM-15353	0									
Lactobacillus aviarius aviarius	DSM-20655	0									
Lactobacillus aviarius araffinosus	DSM-20653	0									
Lactobacillus sucicola	DSM-21376	0									
Lactobacillus aquaticus	DSM-21051	2	Mundticin_ATO6	Unmodified	Yes	Carnocin_CP52	Unmodified	Not a bacteriocin operon			
Lactobacillus uvarum	DSM-19971	0									
Lactobacillus capillatus	DSM-19910	0									
Lactobacillus cacaonum	DSM-21116	0									
Lactobacillus mali	DSM-20444	1	Carnocin_CP52	Unmodified	Yes						
Lactobacillus mali	ATCC-27304	0									
Lactobacillus hordei	DSM-19519	3	Carnocin_CP52	Unmodified	Potential	Plantacarin	Unmodified	yes	Coagulin	Bacteriocin >10kd	Not a bacteriocin operon
Lactobacillus oeni	DSM-19972	0									
Lactobacillus satsumensis	DSM-16230	0									
Lactobacillus vini	DSM-20605	0									
Lactobacillus ghanensis	DSM-18630	0									
Lactobacillus nagelii	DSM-13675	0									
Lactobacillus algidus	DSM-15638	0									
Lactobacillus fabifermentans	DSM-21115	1	Carnocin_CP52	Unmodified	Yes						
Lactobacillus xiangfangensis	LMG-26013	2	Carnocin_CP52	Unmodified	Not a bacteriocin operon	Lactococcin_B	Unmodified	Potential but across multiple contigs			
Lactobacillus pentosus	DSM-20314	2	Plantacarin A	Unmodified	Yes	Pediocin	Unmodified	Not a bacteriocin operon			
Lactobacillus plantarum argentoratensis	DSM-16365	1	Enterocin		Potential but across multiple contigs						
Lactobacillus plantarum	DSM-13273	1	Plantaricin_K	Glyocin	Yes						
Lactobacillus plantarum plantarum	CGMCC-1.2437	1	Plantaricin_K	Glyocin	Yes						
Lactobacillus paraplantarum	DSM-10667	1	Plantaricin_K	Glyocin	Yes						
Lactobacillus siliginis	DSM-22696	0									
Lactobacillus rossiae	DSM-15814	2	Pediocin		Potential	Lactococcin	Unmodified	Potential but across multiple contigs			
Weissella viridescens	DSM-20410	0									
Weissella minor	DSM-20014	0									
Weissella halotolerans	DSM-20190	0									
Weissella confusa	DSM-20196	0									
Weissella kandleri	DSM-20593	0									
Oenococcus oeni	ATCC-BAA-1163	1	Unknown	Head_to_tail_cyclize	Potential						
Oenococcus kitaharae	DSM-17330	1	Streptolysin	LAPs	Yes						
Leuconostoc fallax	KCTC-3537	1	Lactobin A	Unmodified	Yes						
Leuconostoc pseudomesenteroides	4882	0									
Leuconostoc mesenteroides	ATCC-8293	0									
Leuconostoc mesenteroides cremoris	ATCC-19254	0									
Leuconostoc carnosum	JB16	0									
Leuconostoc argentinum	KCTC-3773	1	Lactococcin 972	Unmodified	Potential but across multiple contigs						
Leuconostoc citreum	KM20	1	Unknown	Head_to_tail_cyclize	Yes						
Leuconostoc gelidum	KCTC-3527	2	Enterocin	Unmodified	Potenital	Penocin_A	Unmodified	Yes			
Leuconostoc gasicomitatum	LMG-18811	1	Plantaricin NC8	Unmodified	Potential						
Leuconostoc kimchii	IMSNU-11154	1	Mesentericin B105	Unmodified	Yes						
Fructobacillus fructosus	DSM-20349	0									
Lactobacillus pontis	DSM-8475	0									
Lactobacillus panis	DSM-6035	1	Enterolysin_A	Bacteriocin >10kd	Potential						
Lactobacillus oris	DSM-4864	0									
Lactobacillus antri	DSM-16041	2	Enterolysin_A	Bacteriocin >10kd	Not a bacteriocin operon	Enterolysin	Bacteriocin >10kd	Potential			
Lactobacillus reuteri	DSM-20016	2	Enterolysin_A	Bacteriocin >10kd	Not a bacteriocin operon	Bovicin		Not a bacteriocin operon			
Lactobacillus vaginalis	DSM-5837	2	Enterolysin_A	Bacteriocin >10kd	Not a bacteriocin operon	Enterolysin_A	Bacteriocin >10kd	Not a bacteriocin operon			
Lactobacillus frumenti	DSM-13145	1	Enterolysin_A	Bacteriocin >10kd	Not a bacteriocin operon						
Lactobacillus fermentum	DSM-20055	1	Enterolysin_A	Bacteriocin >10kd	Not a bacteriocin operon						
Lactobacillus equigenerosi	DSM-18793	2	Enterolysin_A	Bacteriocin >10kd	Not a bacteriocin operon	Enterolysin	Bacteriocin >10kd	Potential			
Lactobacillus gastricus	DSM-16045	1	Cytolysin A	Lanthipeptide_class_	Yes						
Lactobacillus ingluviei	DSM-15946	2	Enterolysin_A	Bacteriocin >10kd	Not a bacteriocin operon	Enterolysin	Bacteriocin >10kd	Potential			
Lactobacillus ingluviei	DSM-14792	1	Enterolysin_A	Bacteriocin >10kd	Potential						
Lactobacillus secaliphilus	DSM-17896	0									
Lactobacillus coleohominis	DSM-14060	0									
Lactobacillus mucosae	DSM-13345	2	Enterolysin_A	Bacteriocin >10kd	Not a bacteriocin operon	Enterolysin	Bacteriocin >10kd	Not a bacteriocin operon			
Lactobacillus oligofermentans	DSM-15707	0									
Lactobacillus hokkaidonensis	DSM-26202	1	Leucocin A like	Unmodified	Yes						
Lactobacillus suebicus	DSM-5007	0									
Lactobacillus vaccinostercus	DSM-20634	0									
Lactobacillus parabrevis	LMG-11984	0									
Lactobacillus parabrevis	ATCC-53295	0									
Lactobacillus hammesii	DSM-16381	0									
Lactobacillus paucivorans	DSM-22467	0									
Lactobacillus senmaizukei	DSM-21775	0									
Lactobacillus brevis	DSM-20054	0									
Lactobacillus koreensis	JCM-16448	0									
Lactobacillus zymae	DSM-19395	0									
Lactobacillus acidifarinae	DSM-19394	0									
Lactobacillus namurensis	DSM-19117	0									
Lactobacillus spicheri	DSM-15429	0									
Lactobacillus kimchicus	JCM-15530	1	Pediocin	Unmodified	Yes						
Lactobacillus similis	DSM-23365	1	Unknown	Unknown	Potential but across multiple contigs						
Lactobacillus odoratitofui	DSM-19909	0									
Lactobacillus collinoides	DSM-20515	0									
Lactobacillus paracollinoides	DSM-15502	0									
Lactobacillus malefermentans	DSM-5705	0									
Lactobacillus parabuchneri	DSM-5707	1	Helveticin J	Bacteriocin >10kd	Not a bacteriocin operon						

Lactobacillus parabuchneri	DSM-15352	0												
Lactobacillus buchneri	DSM-20057	1	Unknown	Head_to_tail_cyclize	Potential									
Lactobacillus otakiensis	DSM-19908	1	Unknown	Head_to_tail_cyclize	Not a bacteriocin operon									
Lactobacillus kefiri	DSM-20587	0												
Lactobacillus parakefiri	DSM-10551	0	Unknown	Unmodified	Potential									
Lactobacillus sunkii	DSM-19904	0												
Lactobacillus rapi	DSM-19907	0												
Lactobacillus kisonensis	DSM-19906	0												
Lactobacillus diolivorans	DSM-14421	0												
Lactobacillus hilgardii	DSM-20176	0												
Lactobacillus farraginis	DSM-18382	1	Helveticin J	Bacteriocin >10kd	Not a bacteriocin operon									
Lactobacillus parafarraginis	DSM-18390	1	Helveticin J	Bacteriocin >10kd	Potenial									
Lactobacillus senioris	DSM-24302	0												
Lactobacillus ozensis	DSM-23829	0												
Lactobacillus kunkeei	DSM-12361	0												
Lactobacillus florum	DSM-22689	0												
Lactobacillus lindneri	DSM-20690	0												
Lactobacillus sanfranciscensis	DSM-20451	0												
Lactobacillus fructivorans	ATCC-27394	0												
Lactobacillus homohiochii	DSM-20571	0												
Lactobacillus fructivorans	DSM-20350	0												
Lactobacillus fructivorans	DSM-20203	0												
Pediococcus argentinicus	DSM-23026	0												
Pediococcus claussenii	DSM-14800	1	SalivaricinA	Lactipeptide	Potential									
Pediococcus pentosaceus	DSM-20336	1	Pediocin	Unmodified	Potential									
Pediococcus stilesii	DSM-18001	2	Enterolysin_A	Bacteriocin >10kd	Not a bacteriocin operon	Carnocin_CP52	Unmodified	Yes						
Pediococcus lolii	DSM-19927	1	Enterolysin_A	Bacteriocin >10kd	Not a bacteriocin operon									
Pediococcus acidilactici	AS1-2696	0												
Pediococcus ethanolidurans	DSM-22301	2	Pediocin like	Unmodified	Yes	Carnocin_CP52	Unmodified	Not a bacteriocin operon						
Pediococcus cellicola	DSM-17757	1	Carnocin_CP52	Unmodified	Potential									
Pediococcus damnosus	DSM-20331	2	Pediocin /Sacakcin	Unmodified	Yes	SalivaricinA	Lanthipeptide_class	Not a bacteriocin operon						
Pediococcus inopinatus	DSM-20285	1	Enterolysin_A	Bacteriocin >10kd	Not a bacteriocin operon									
Pediococcus parvulus	DSM-20332	1	Enterolysin_A	Bacteriocin >10kd	Prophage									
Carnobacterium maltaromaticum	DSM-20730	3	Piscicolin_126	Unmodified	Yes	Cytolysin_ClyLs	Lanthipeptide_class	Potential	Carnobacteriocin_B	Unmodified	Potential			
Carnobacterium maltaromaticum	DSM-20342	2	Carnocin_CP52	Unmodified	Potenital	Unknown	Sactipeptides	Not a bacteriocin operon						
Carnobacterium maltaromaticum	DSM-20722	4	Carnobacteriocin B:	Unmodified	Yes	Carnobacteriocin_BM1	Unmodified	Potential	Unknown	Sactipeptides	Not a bacteriocin operc	Cytolysin_Cl	Lanthipeptic	Potential
Carnobacterium divergens	DSM-20623	0												
Lactococcus lactis	LMG-7760	0	Plantacarin A	Unmodified	Not a bacteriocin operon									
Atopobium minutum	DSM-20586	0												
Olsenella uli	DSM-7084	0												
Atopobium rimae	DSM-7090	0												

Supplementary Notes

Supplementary Note 1

Lactobacillus relatedness to other genera

We also sequenced genomes of type strains from other genera associated with lactic acid bacteria (LAB) including *Carnobacterium* and *Lactococcus* and other genera previously misidentified as LAB, namely, *Atopobium*, *Kandleria* and *Olsenella*. *Carnobacterium* and *Lactococcus* form distinct branches and are classified in the families *Carnobacteriaceae* and *Streptococcaceae,* respectively, revealing closer genetic relatedness to genera other than *Lactobacillus*. *Carnobacterium* is closely related to *Melissococcus* and *Lactococcus* to *Streptococcus* (Fig. 1). This indicates that all currently known LAB descend from the same ancestor as proposed previously[1]. The un-relatedness of *Kandleria, Atopobium* and *Olsenella* and other LAB is confirmed, with a closer relationship of *Kandleria* with *Erysipelothrix* in the family *Erysipelotrichaceae* of the *Clostridium* subphylum cluster XVII of *Firmicutes. Atopobium* and *Olsenella* revealed the greatest genetic distance from the genus *Lactobacillus*; they belong to another phylum, *Actinobacteria,* which belongs to the high-GC, Gram-positive bacteria, the most closely related genus being *Coriobacterium*.

Supplementary Note 2

Validation of the core genome phylogeny

We validated the branching order of the 73 core gene tree using alternative subsets of gene datasets. We inferred a tree of the *Lactobacillus* genus complex, rooted on *Lactococcus lactis*, comprised of 117 genes, and another tree of the *Lactobacillus* genus complex, rooted on the *Carnobacteria*, comprised of 121 genes. We found there were only 4 minor branching alterations. The robustness of the 73 core gene tree was also assessed using a range of different models. Models tested were the WAG, LG, JTT, mtREV and Dayhoff models. The resulting branching order of the strains was consistent across all the models with the exception of mtREV. With this model the positions of *L. salivarius* DSM_20555, *L. hayakitensis* DSM_18933 and *L. acetotolerans* DSM_20749 underwent minor node exchanges within the same clade.

The impact of adding partial genes to the core gene dataset was assessed by inferring a phylogeny (Supplementary Fig. 19) from the 73 complete core genes with an additional 27 partial core genes (listed in Supplementary Table 8) added to the dataset. The inferred topology was highly congruent with the 73 complete core gene tree with only three very minor branching alterations. Specifically, *L. koreensis* moved one branch within the *L. brevis*/*L. collinoides* group. Minor displacements were also observed for *L. kefiranofaciens* DSM_5016 and DSM_10550 within the L. *delbrueckii* group and *L. salivarius* and *L. hayakitensis* within the *L. salivarius* group.

Supplementary Note 3

Relatedness of *Lactobacillus* species

At the species level, the combination of ANI value and core genome phylogeny could be proposed as the basis for optimal taxonomic classification[2], and its application could shed new light on several issues including species widely used in the food and probiotic industry. A long-debated case has been that of the *L. casei* group[3]. We propose that the species *L. casei* and *L. paracasei* should be combined into a single species, *L. casei*, because the pair-wise ANI values between the type strains of *L. casei* and two *L. paracasei* are 98~99%, larger than the "un-official" species' cut-off value of 95%[4], and these three strains clustered together as a monophyletic group (Fig. 2; Supplementary Fig. 20). The designation of *L. zeae* has been controversial and there are reports suggesting its classification into the species *L. casei*[3,5]. However, the ANI value between *L. casei* and *L. zeae* is only 77%~78%, which is similar to the value between *L. casei* and another well-defined species, *L. rhamnosus*. Therefore, our genomic analysis supports retaining *L. zeae* as a single species. Remarkably, ANI values seem not to be related to total DNA-DNA hybridization data reported previously[5] for similarities of *L. casei* type strain ATCC 393, which corresponds to the strain DSM 20011 included in this study.

The genus *Lactobacillus* was recently defined as 16 phylogroups (incl. *Pediococcus*), 4 couples (groups containing only two species) and 10 single species[6]. Such delineations are generally supported by the phylogenetic relationships constructed here based on the core proteins, but two modifications are suggested (Fig. 2; Supplementary Fig. 18). Firstly, *L. camelliae* could be included in the *L. manihotivorans* group and not in the *L. delbrueckii* group, supported by a mean TNI value of <15% to the group, which is far lower than that within the other *L. delbrueckii* group species (79.5%). Moreover, *L. amylotrophicus* and *L. amylophilus* should also be classified as a single species, *L. amylophilus*, as the two type strains have an ANI value of approximately 100%, although multilocus analysis and DNA-DNA hybridization values suggested their separation.

Supplementary Note 4

Phylogenomics of glycolysis and hexose fermentation

In species characterized by the presence of Pfk, the distribution of the pyruvate dehydrogenase operon (Pdh; composed of 4 genes) reflected carbohydrate metabolism since it is absent in 57% of obligately homofermentative species while it is present in facultatively heterofermentative members. Consistency was observed between Pdh distribution and phylogenetic groupings: 90% of the species of the *L. delbrueckii* group lack *pdh*, while members of groups like *L. salivarius*, *L. plantarum*, *L. casei* or *L. alimentarius* are characterized by presence of both *pfk* and *pdh,* although they belong to different phenotypic categorizations. Additionally, 80% of the species within the *L. delbrueckii* group lack

glucokinase, the first enzyme of the glycolytic pathway, except for the monophyletic subgroup formed by the *L. delbrueckii* and *L. equicursoris* species. For growth on glucose, a PTS transport system may obviate the need for this enzyme[7] and most of these species (79%) are classified as obligately homofermentative. Thus, comparative genomic analysis of glycolysis reveals that species inside the historically defined groups have a coherent genotypic background despite metabolic heterogeneity.

Supplementary Note 5

Carbohydrate active enzymes

Some GH families are present more uniformly across the dataset, indicating the importance of the biotransformations associated with these families. Included in this are enzymes involved in the hydrolysis of peptidoglycan (GH25 and GH73[8]), which play an important role in cell division, growth and preserving cell wall integrity. Bacterial autolysis can have a positive impact in the dairy fermentation process through the enhancement of cheese flavour upon the release of enzymes and amino acids[9]. Starch degradation enzymes are also present almost uniformly across the genome set. α-amylase enzymes are catalysts in the hydrolysis of the α-1,4 glycosidic linkages of starch with GH13 being the main GH family acting on substrates with α-glucoside linkages. α-glucan metabolism is important in the breakdown of resistant starch. Another universal family of GHs is GH65; this family is mainly composed of phosphorylases, including maltose and treholase phosphorylase, which are essential for the survival of lactobacilli in sugar-rich environments such as sourdough, where maltose is the predominant sugar[10].

Six clades display unusually high GH abundance, namely, *L. (par)alimentarius*, *L. perolens*, *L. plantarum*, *L. rapi*, *L. fructivorans* and *Carnobacterium* spp. The *Weissella* spp. and *L. fructivorans* clade show an unusually low GH gene count. The most abundant GH families are GH1, GH13, GH25 and GH73. GH73, an N-acetylmuramidase, is present in all *Lactobacillus* species except *L. equi*. The four *Carnobacterium* genomes harbor several GH families that are absent in all or most of the other genomes including GH18, GH24, GH84, GH85 and GH119. GH18 enzymes are chitinases, GH24 are lysozymes while GH119 enzymes are involved in chitin binding.

Some rare GTs emerge in the dataset. *P. lolii* and *P. parvulus* are the only strains that harbour genes for GT12, a ganglioside synthase. This activity is very interesting as it has been reported in only 3 bacterial species and 28 eukaryotes. Production of GT11 may be a strain-specific trait, because a single non-type strain of each of *L. johnsonii*, *L. amylovorus* and *L. paraplantarum* also appear to encode this activity (http://www.cazy.org/GT11_bacteria.html).

A number of GT families are present almost uniformly across the dataset. These include GT51, which is involved in peptidoglycan synthesis[8] and only *L. coleohominis* lacks

GT28, a galactosyltransferase involved in cell wall metabolism, suggesting alternative cell wall structure in this vaginal isolate. Other ubiquitous families include more broad-spectrum GTs that have been termed “polyspecific” because of their diverse functionality. Examples include GT4 and GT2, which encompass at least 12 functions including cellulose synthase, chitin synthase and mannosyltransferase[11].

The abundance of genes encoding carbohydrate transporters correlates strongly with GH abundance and less strongly with GT abundance (Supplementary Fig. 21). The clade distribution of carbohydrate transporter gene counts (Supplementary Fig. 22) mirrors this correlation and highlights the relative abundance of carbohydrate management machinery in the *L. alimentarius*, *L. casei*, *L. mali*, *L. plantarum* and *L. collinoides* clades, the pediococci and carnobacteria. Normalization for genome size (Supplementary Fig. 23) reduces the apparent overabundance of GT genes in some species, but not GH genes (Supplementary Fig. 23), and it is debatable if such normalization is biologically relevant.

Supplementary Note 6
Metabolic diversity of the lactobacilli

A general representation of the genome content of the lactobacilli as Clusters of Orthologous Groups (COGs; Supplementary Fig 15) reveals unexpected diversity in categories including Transcription, Cell wall biogenesis, Energy production, Co-enzyme transport, and Inorganic ion transport. The *L. plantarum*-related species from *L. fabifermentans* through *L. paraplantum* (in Fig. 2) are particularly endowed with genes for carbohydrate and amino acid transport and metabolism. Members of the *L. salivarius* clade have the highest number of genes involved in cell motility and secretion, as expected from previous studies in our group[12]. Forty nine (23%) of the genomes screened had none of the complete pathways for production of the 20 standard amino acids (AA), while no single genome harboured the genes to produce all 20 AAs. The highest number of pathways encoded by any one species was sixteen in *L. similis* (Supplementary Table 9). Some phylogenetic clades harbored genes for the production of one or two amino acids; these include *Weissella, L. brevis, Pediococcus* and *L. sakei*. The clade containing *L. collinoides* and *L. kimchicus* as well as the *L. coryniformis* clade (with the exception of *L. rennini*) are predicted to be prototrophic for at least 14 AAs. In contrast, ten of the twelve dairy isolates have genes for five or fewer complete AA pathways, reflecting their evolution to the AA-rich dairy environment.

Supplementary Note 7
Sortase-anchored proteins

The distribution of LPXTG proteins was not clearly correlated with the evolutionary relationship of lactobacilli. Rather, it seems that the decoration of the cell wall with anchored

proteins is a common feature, indicating that most lactobacilli can establish interactions with the environment. In our dataset of 213 genomes, only twelve bacterial strains do not have LPXTG proteins (Supplementary Table 5). In addition, most lactobacilli genomes harbor at least one gene encoding sortase, presumably the housekeeping sortase. The copy number of sortase genes could be associated to some extent with the presence of pilus gene clusters (PGC) (Fig. 4). These PGCs were identified in 51 strains in total, mostly belonging to six clades. The bacterial species with the most pilus gene clusters was *Lactobacillus sharpeae,* a sewage isolate. We also observed a large diversity of PGCs in terms of gene organization and numbers, suggesting they may have distinct gene order and functions (Supplementary Fig. 10). One of the most common PGC types consists of three pilin genes and one sortase gene, which is similar to the PGC originally reported in *L. rhamnosus* GG[13].

Supplementary Note 8

The *Lactobacillus* mobilome

The mobile component of the bacterial genome can expand coding capacity as in the case of plasmids and megaplasmids[14] or may be associated with genome decay as in the case of Insertion Sequence (IS) elements[15]. Some IS elements exhibit a very limited distribution across the 213 genomes, for example, IS1 which is restricted to only *L. ingluvei* and *L. equi* (Supplementary Fig. 16). IS91 is present in the genomes of only two species, both of dairy-product origin (*L. casei* and *L. paracasei* subsp. *tolerans*), and IS481 is found in only 3 strains (*L. paracollinoides*, *L. farraginis* and *P. inopinatus*) all associated with brewing. IS3, on the other hand, exhibits a much greater distribution being present in almost all groups, with the exception of *Weissella*/*Leuconostoc*, *L. fructivorans*, some *L. delbrueckii* strains and a few singletons. Some genomes apparently harbor no IS elements, perhaps indicating a more rigid architecture and a selective pressure against the acquisition of such elements. These species include *Atopobium minutum*, *L. fructivorans*, *L. florum*, *L. senoris*, *Weissella viridescens*, *L. cacaonum*, *L. apodemi*, *L. ceti* and *L. brantae*. Of the 18 IS families in this database, the largest number of families found is 13, in the *L. parabuchneri* genome. It is clear that IS elements have played a general role in shaping the diversification and evolution of the lactobacilli.

Phages were detected in the genomes of 195 of the 213 genomes (Supplementary Fig. 13), and only the genomes of *L. floricola*, *L. ingluviei*, *L. psittaci*, *L. sakei* subsp. *sakei*, *L. sanfranciscensis*, *P. cellicola*, *P. claussenii* and *W. halotolerans* lacked homologues of phage proteins. Proteins corresponding to holins and endolysins, and fibers/fiber assembly proteins were apparently under-represented; this is likely due to the fact that holins are not well annotated, whereas fiber proteins are very divergent, and not always present in the phage. It is likely that this analysis under-reports phage genes and that fine tuning of homology cut-offs for individual genes will identify more temperate phage. We identified plasmids in 41% of the

213 genomes analysed (Supplementary. Fig. 14) with numbers ranging from zero to six. 58% of the plasmid-related genes were of unknown function, emphasizing the need for functional genomics to elucidate this gene repertoire. Given the desirability of finding new plasmid vectors for genetically manipulating lactobacilli in the laboratory and for food-grade strain construction, these genomic data represent a valuable resource. Of the 87 strains predicted to have plasmids, 75 had identifiable replication genes. The other 12 may harbor new replication types that would be compatible with existing plasmid vectors.

Supplementary Note 9
Stress resistance

The broad range of niches that lactobacilli occupy is reflected in the multiple stress resistance mechanisms their genomes encode (Supplementary Fig. 24). Knowledge of the differential abundance of these systems can be exploited for identifying species and strains that can withstand production stress, storage stress or intestinal survival[16]. Superoxide dismutase and catalase show very limited distribution, as does the gene encoding glutamate decarboxylase involved in proton scavenging and acid resistance (Supplementary Fig. 24), the last of which is concentrated in *L. helveticus* and related species. Urease genes were present in only 8 species including 3 of the 4 *Carnobacteria* species. Bile salt hydrolases contribute to bile resistance in lactobacilli[17]; the pattern of their distribution in this genome data resource, viewed in conjunction with other stress resistance genes, allows rational identification of species likely to survive intestinal transit.

Protein acetyltransferases of prokaryotes like *Escherichia coli* confer resistance to heat and oxidative stress[18], and it was recently reported that homologs of the eukaryotic sirtuin protein acetyltransferases contribute to stress resistance in *Lactobacillus paracasei*[19]. Forty of the 213 genomes analyzed here lack homologs of any of SIRT1, SIR2L1, or Sir2α (Supplementary Table 10). The remaining 173 genomes encode at least one homolog, with a single species *L. ultunensis* harbouring 4 homologs, and 3 homologs being found in a large number of food or intestinal lactobacilli. Since pre-treatment of *Lactobacillus paracasei* strains with the sirtuin activator resveratrol (found in berries and red wine) alleviated growth inhibition by cholate[19], this presents the exciting prospect that some food ingredients might promote shelf-life of lactobacilli in functional food products, or that certain prebiotics might rationally promote survival and intestinal transit of lactobacilli administered in synbiotics.

Supplementary Note 10
Niche association and genome content

A search for associations between niche and genome content for the 213 genomes revealed moderate trends. The strongest trends were detected for species isolated from animal sources (n=56), which, as noted in the main text, also had the lowest number of

predicted genes and the smallest average genome size (Supplementary Fig. 25). This is evident at a functional level where numerous functional gene groups displayed the lowest abundances in the animal niche. These groups include genes for the transport and metabolism of carbohydrates (Supplementary Fig. 25, top panel), amino acids, lipids (Supplementary Fig. 25, top panel), co-enzymes, nucleotides and inorganic acids. Normalization for genome size brought the difference above the significance threshold (Supplementary Fig. 25, bottom panels; p-values of 0.11 and 0.107 for carbohydrate transport and lipid metabolism, respectively). Genes involved in transcription, cell wall/membrane biogenesis and secondary metabolites also have the lowest average abundance in the animal niche. Gene decay is commonly associated with host-adapted microbes and this is a possible explanation for the trend observed in genomes that have been isolated from animal sources.

Across the broader range of niches/sources, the analyses failed to detect niche-specific genomic associations in our study, but rather a general pattern of gene decay in species from the animal niche. This study focuses largely on the comparison of type strains within the genus *Lactobacillus* and associated species so it is likely that the diversity among species in the dataset was too great to reveal niche-specific traits. Studies that focus on multiple strains of the same species are more suited to discovering niche-specific genes.

Supplementary Note 11
Targeting competing microbes

Bacteriocins are small, ribosomally synthesized antimicrobial peptides that can be exploited as antimicrobial-producing cultures in fermented foods, or the bacteriocins themselves added, e.g. pediocin and carnobacteriocin used as biopreservatives[20]. Bacteriocins may also contribute to probiotic properties by limiting infection[21] or signalling to the innate immune system[22]. Of the 213 genomes analysed here, 107 (50.2%; Supplementary Table 11) harbored at least one Area of Interest (AOI) relating to bacteriocin production by screening against the BAGEL database[23]. Over half of these were larger proteins (>10 kDa) of the enterolysin/helveticin class, which are no longer considered classical bacteriocins[24]. However, their widespread distribution suggests a central function for these currently cryptic antimicrobials. Manual inspection confirmed that 38 AOIs had the contiguous gene structure expected for typical bacteriocin operons. No dominant bacteriocin type was identified, although 58% of intact AOIs fell under the Unmodified Bacteriocin (Class II) category. Many of these included homologs of well-known bacteriocin operons including plantaricin, sakacin, salivaricin, subtilin, leucocin, carnobacteriocin and lacticin F. Surprisingly, AOIs were identified in many species not previously associated with bacteriocin production. For example, genes encoding pediocins were annotated in *L. kimchicus* and *L. taiwanensis* was found to harbour the machinery to produce subtilin. Predicted bacteriocin loci were found across all

clades of the phylogenetic tree with the exception of *Weissella* and the *L. brevis/L.parabrevis* clade. Overall, the data do not support strict associations between species niche, the presence of a bacteriocin or type of bacteriocin. Moreover, the overall prevalence of bacteriocins was unexpectedly low compared to the literature describing this area, suggesting that many more *Lactobacillus* species can produce bacteriocins than those represented by their type strains examined in this study (for example *L. salivarius*[25]).

Supplementary References

1 Makarova, K. *et al.* Comparative genomics of the lactic acid bacteria. *Proc. Natl. Acad. Sci. U S A* **103**, 15611-15616, (2006).

2 Chan, J. Z., Halachev, M. R., Loman, N. J., Constantinidou, C. & Pallen, M. J. Defining bacterial species in the genomic era: insights from the genus Acinetobacter. *BMC Microbiol* **12**, 302, (2012).

3 Judicial Commission of the International Committee on Systematics of Bacteria. The type strain of Lactobacillus casei is ATCC 393, ATCC 334 cannot serve as the type because it represents a different taxon, the name Lactobacillus paracasei and its subspecies names are not rejected and the revival of the name 'Lactobacillus zeae' contravenes Rules 51b (1) and (2) of the International Code of Nomenclature of Bacteria. Opinion 82. *Int J Syst Evol Microbiol* **58**, 1764-1765, (2008).

4 Goris, J. *et al.* DNA-DNA hybridization values and their relationship to whole-genome sequence similarities. *Int. J. Syst. Evol. Microbiol.* **57**, 81-91, (2007).

5 Dicks, L. M., Du Plessis, E. M., Dellaglio, F. & Lauer, E. Reclassification of Lactobacillus casei subsp. casei ATCC 393 and Lactobacillus rhamnosus ATCC 15820 as Lactobacillus zeae nom. rev., designation of ATCC 334 as the neotype of L. casei subsp. casei, and rejection of the name Lactobacillus paracasei. *Int J Syst Bacteriol* **46**, 337-340, (1996).

6 Salvetti, E., Torriani, S. & Felis, G. E. The genus *Lactobacillus*: a taxonomic update. *Probiotics Antimic. Proteins* **4**, 217-226, (2012).

7 Grossiord, B., Vaughan, E. E., Luesink, E. & de Vos, W. M. Genetics of galactose utilisation via the Leloir pathway in lactic acid bacteria. *Lait* **78**, 77-84, (1998).

8 Cayrou, C., Henrissat, B., Gouret, P., Pontarotti, P. & Drancourt, M. Peptidoglycan: a post-genomic analysis. *BMC Microbiol.* **12**, 294, (2012).

9 Huard, C. *et al.* Characterization of AcmB, an N-acetylglucosaminidase autolysin from *Lactococcus lactis*. *Microbiology* **149**, 695-705, (2003).

10 Ehrmann, M. A. & Vogel, R. F. Maltose metabolism of *Lactobacillus sanfranciscensis:* cloning and heterologous expression of the key enzymes, maltose phosphorylase and phosphoglucomutase. *FEMS Microbiol. Lett.* **169**, 81-86, (1998).

11 Breton, C., Snajdrova, L., Jeanneau, C., Koca, J. & Imberty, A. Structures and mechanisms of glycosyltransferases. *Glycobiology* **16**, 29R-37R, (2006).

12 Neville, B. A. *et al.* Characterization of Pro-Inflammatory Flagellin Proteins Produced by Lactobacillus ruminis and Related Motile Lactobacilli. *PLoS One* **7**, e40592, (2012).

13 Kankainen, M. *et al.* Comparative genomic analysis of Lactobacillus rhamnosus GG reveals pili containing a human- mucus binding protein. *Proc Natl Acad Sci U S A* **106**, 17193-17198, (2009).

14 Li, Y. *et al.* Distribution of megaplasmids in *Lactobacillus salivarius* and other lactobacilli. *J. Bacteriol.* **189**, 6128-6139, (2007).

15 Callanan, M. *et al.* Genome sequence of *Lactobacillus helveticus*, an organism distinguished by selective gene loss and insertion sequence element expansion. *J. Bacteriol.* **190**, 727-735, (2008).

16 Zhang, Y. & Li, Y. Engineering the antioxidative properties of lactic acid bacteria for improving its robustness. *Curr. Opin. Biotechnol.* **24**, 142-147, (2013).

17 Fang, F. *et al.* Allelic variation of bile salt hydrolase genes in *Lactobacillus salivarius* does not determine bile resistance levels. *J. Bact.* **191**, 5743-5757, (2009).

18 Ma, Q. & Wood, T. K. Protein acetylation in prokaryotes increases stress resistance. *Biochem. Biophys. Res. Commun.* **410**, 846-851, (2011).

19 Atarashi, H. *et al.* in *LAB11 2014, poster B014.* (Egmon aan Zee, Netherlands, 2014).

20 Cotter, P. D., Hill, C. & Ross, R. P. Bacteriocins: developing innate immunity for food. *Nat. Rev. Microbiol.* **3**, 777-788, (2005).

21 Corr, S. C. *et al.* Bacteriocin production as a mechanism for the antiinfective activity of *Lactobacillus salivarius* UCC118. *Proc. Natl. Acad. Sci. U S A* **104**, 7617-7621, (2007).

22 Meijerink, M. *et al.* Identification of genetic loci in Lactobacillus plantarum that modulate the immune response of dendritic cells using comparative genome hybridization. *PLoS One* **5**, e10632, (2010).

23 de Jong, A., van Heel, A. J., Kok, J. & Kuipers, O. P. BAGEL2: mining for bacteriocins in genomic data. *Nucleic Acids Res.* **38**, W647-651, (2010).

24 Arnison, P. G. *et al.* Ribosomally synthesized and post-translationally modified peptide natural products: overview and recommendations for a universal nomenclature. *Nat Prod Rep* **30**, 108-160, (2013).

25 O'Shea, E. F. *et al.* Production of multiple bacteriocins from a single locus by gastrointestinal strains of *Lactobacillus salivarius*. *J. Bacteriol.* **193**, 6973-6982, (2011).
